# Synthesis of oligosaccharides to identify an immunologically active epitope against *Candida auris* infection†

**DOI:** 10.1039/d3sc01242e

**Published:** 2023-06-23

**Authors:** Rajat Kumar Singh, Emelie E. Reuber, Mariolina Bruno, Mihai G. Netea, Peter H. Seeberger

**Affiliations:** a Department of Biomolecular System, Max Planck Institute of Colloids and Interfaces 14476 Potsdam Germany peter.seeberger@mpikg.mpg.de; b Institute of Chemistry and Biochemistry, Freie Universität Berlin 14195 Berlin Germany; c Department of Internal Medicine, Radboud Institute for Molecular Life Sciences, Radboud University Medical Center Nijmegen The Netherlands; d Department of Immunology and Metabolism, Life & Medical Sciences Institute (LIMES), University of Bonn Bonn Germany

## Abstract

*Candida auris* (*C. auris*) is an emerging multidrug-resistant fungal pathogen that represents a significant public health challenge as it can spread rapidly and result in high mortality rates. The mannans on the *C. auris* cell surface are potent immunogens and attractive targets for developing a glycoconjugate vaccine. We synthesized the oligosaccharides resembling cell surface mannans of *C. auris* and printed them onto microarray slides that were used to screen plasma from mice infected with *C. auris*. IgM antibodies in mouse plasma recognize the β-1,2 linkage present in *C. auris* surface mannans. Disaccharide 19 emerged from glycan array screening as a lead for developing a vaccine against *C. auris*, as the majority of patient plasma samples showed antibodies against this glycan. The synthetic oligosaccharides can be used for the early detection of *C. auris* infections.

## Abstract 2

Ten synthetic oligosaccharides resembling *Candida auris* cell surface mannans are used for plasma screening to identify a vaccine lead.

## Graphical abstract

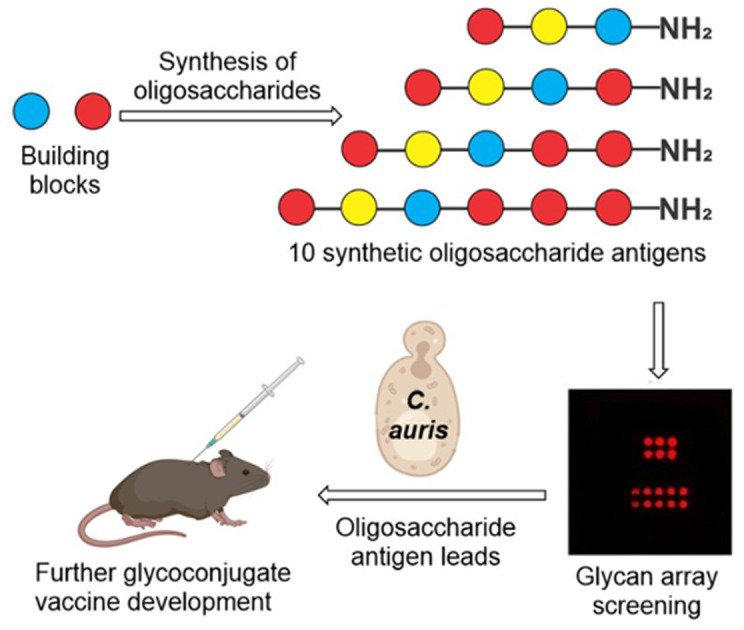

## Introduction

*Candida auris* (*C. auris*) is a species of yeast that was first reported^1^ in 2009 and is now on the World Health Organization (WHO) list as a “critical priority group” pathogen.^2^ The fungus causes multidrug-resistant and potentially life-threatening infections that can result in sepsis, meningitis, and other serious conditions if left untreated. *C. auris* infections spread most commonly in healthcare settings from person to person through contact with contaminated surfaces or medical equipment.^3^*C. auris* infections are typically diagnosed by a combination of laboratory testing and medical history.^4^ Treatment of *C. auris* infections is often difficult as resistance to anti-fungal drugs such as fluconazole, amphotericin, voriconazole and caspofungin is emerging.^5^ Anti-fungal vaccines are urgently needed.

**Fig. 1:**
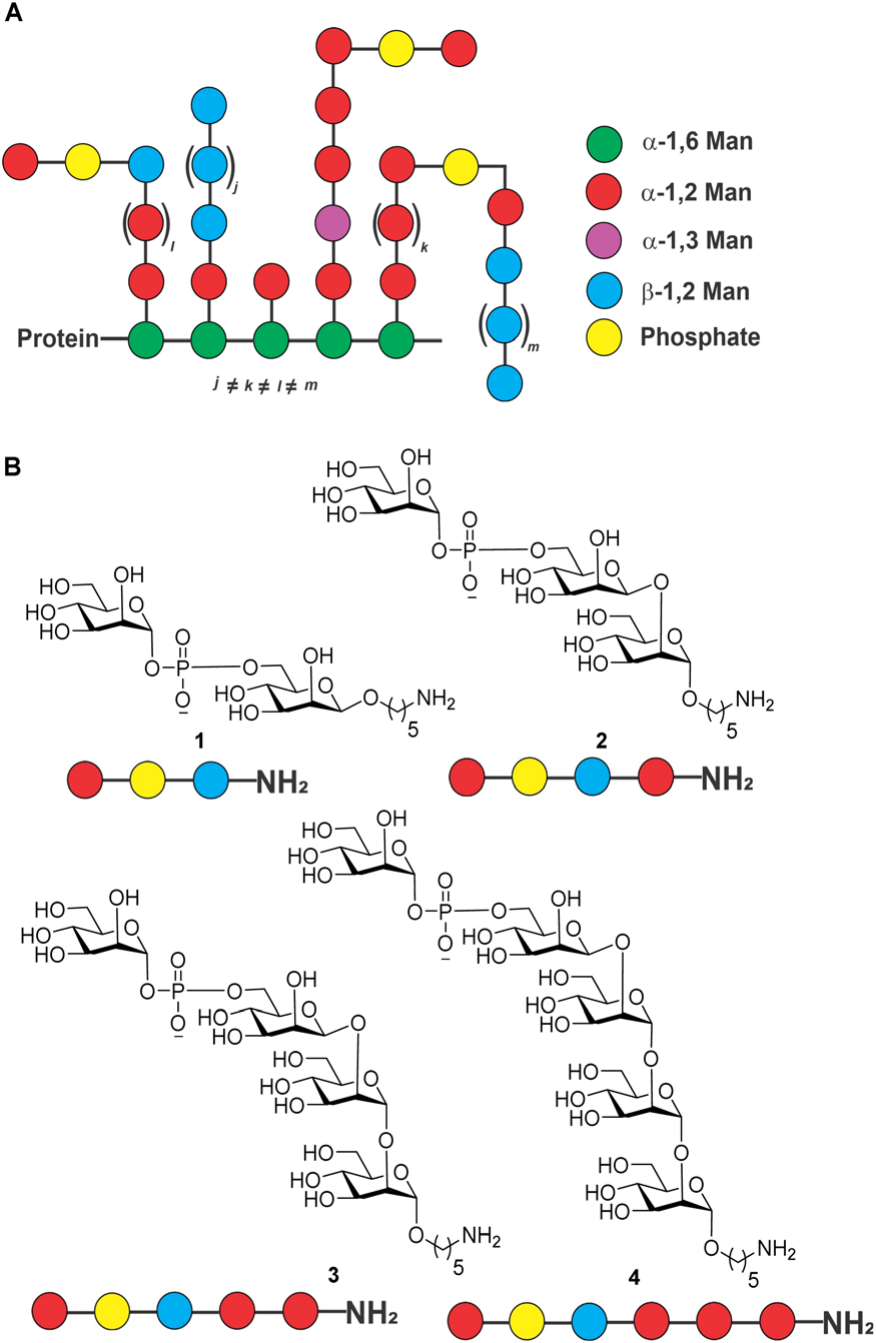
(A) Structure of *C. auris* mannans; (B) structure of synthetic mannans resembling the *C. auris* cell surface.

The outer membrane of the fungus contains a variety of proteins, including outer membrane proteins, lipoproteins, and glycoproteins. The polysaccharide-rich cell membrane is composed of chitin and β-glucans in the inner layer, and mannoprotein in the outer layer of cell wall.^6^*C. auris* mannoproteins are composed of *O*- and *N*-linked mannans with α-1,2-, α-1,3-, α-1,6- and β-1,2-linked mannopyranose units as well as phosphodiester-linked mannopyranose residues (Fig. 1).^6–8^ The cell membrane is important for protecting the fungus from environmental stress, providing a barrier to the entry of antimicrobial agents, and aiding in the adherence to surfaces. The cell membrane polysaccharides are recognized by pattern recognition receptors (PRRs) on the surface of the immune cell and in some cases may contribute to the pathogenicity of *C. auris* by allowing it to evade the host immune system.^9^

**Fig. 2:**
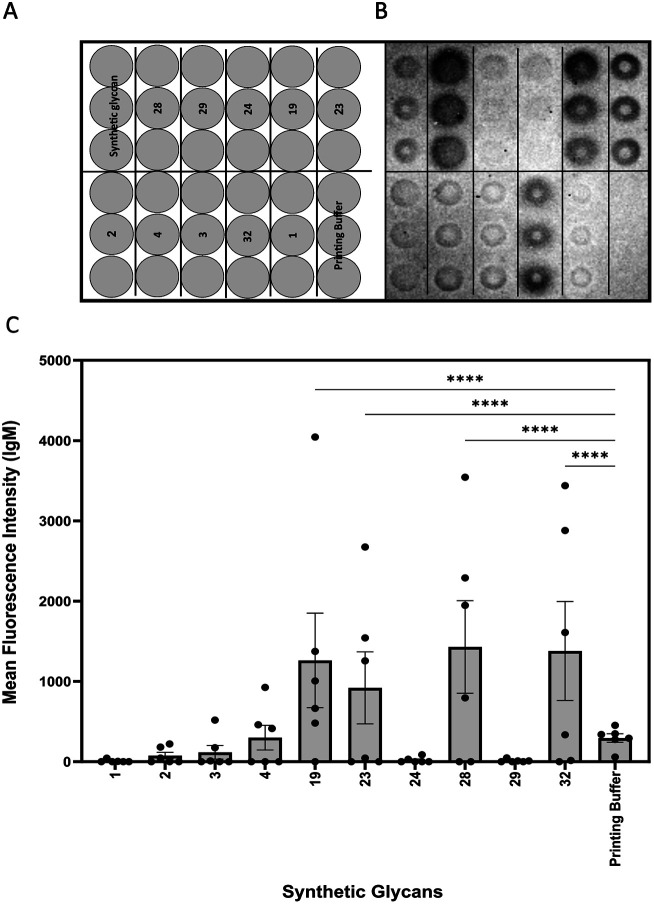
Glycan microarray analysis of *Candida auris* mannans. (A) Printing pattern of glycan microarray. (B) Exemplary binding pattern of mice plasma to immobilized synthetic glycans. (C) Mean fluorescence intensity of IgM antibody binding to synthetic glycans. A plasma dilution of 1 : 50 was used. Values represent mean ± SEM. Differences were tested for significance using one-way ANOVA followed by Tukey's post hoc test with (****) *p* < 0.0001.

Isolated mixtures of *Candida* mannans and mannoproteins were explored as potential vaccine antigens in animal models of candidiasis two decades ago without follow-up.^10,11^ Rather than using isolated mixtures of glycans, defined, synthetic antigens resembling cell surface mannans may constitute attractive vaccine candidates. As a first step in the vaccine design and development process, we report the synthesis of a series of conjugate-ready glycans related to the *C. auris* mannans (Fig. 1) that were used to screen plasma of infected mice for antibodies on glycan microarrays (Fig. 2) to identify a minimal epitope.^12^

## Results and discussion

**Scheme 1:**
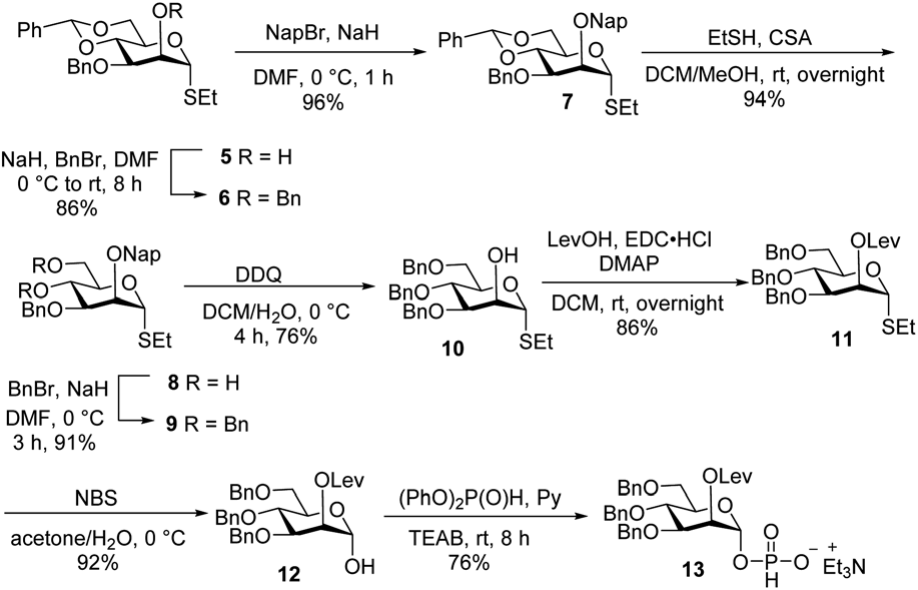
Synthesis of H-phosphonate building block 13.

Glycan assembly is based on orthogonally protected building blocks. *H*-phosphonate building block 13 (Scheme 1), was prepared by the formation of a Nap ether at the C-2 hydroxyl of commercially available thioglycoside 5 to form 7 in 96% yield.^13^ The benzylidene acetal in 7 was hydrolysed using ethanethiol and camphorsulfonic acid (CSA) to give dihydroxyl mannose thioglycoside 8. Dibenzylation of 8 afforded fully protected thioglycoside 9 in 91% yield. Oxidative removal of naphthyl ether using DDQ afforded thioglycoside^14^10 in 76% yield. Levulinoylation of the C2–OH in 10 using 1-ethyl-3-(3-dimethylaminopropyl) carbodiimide hydrochloride (EDC·HCl) and DMAP produced 11 in 86% yield.^15,16^ Hydrolysis of thioglycoside 11 using *N*-bromosuccinimide (NBS) and subsequent treatment of hemiacetal^16^11 with diphenyl phosphite provided *H*-phosphonate 13.^17^

**Scheme 2:**
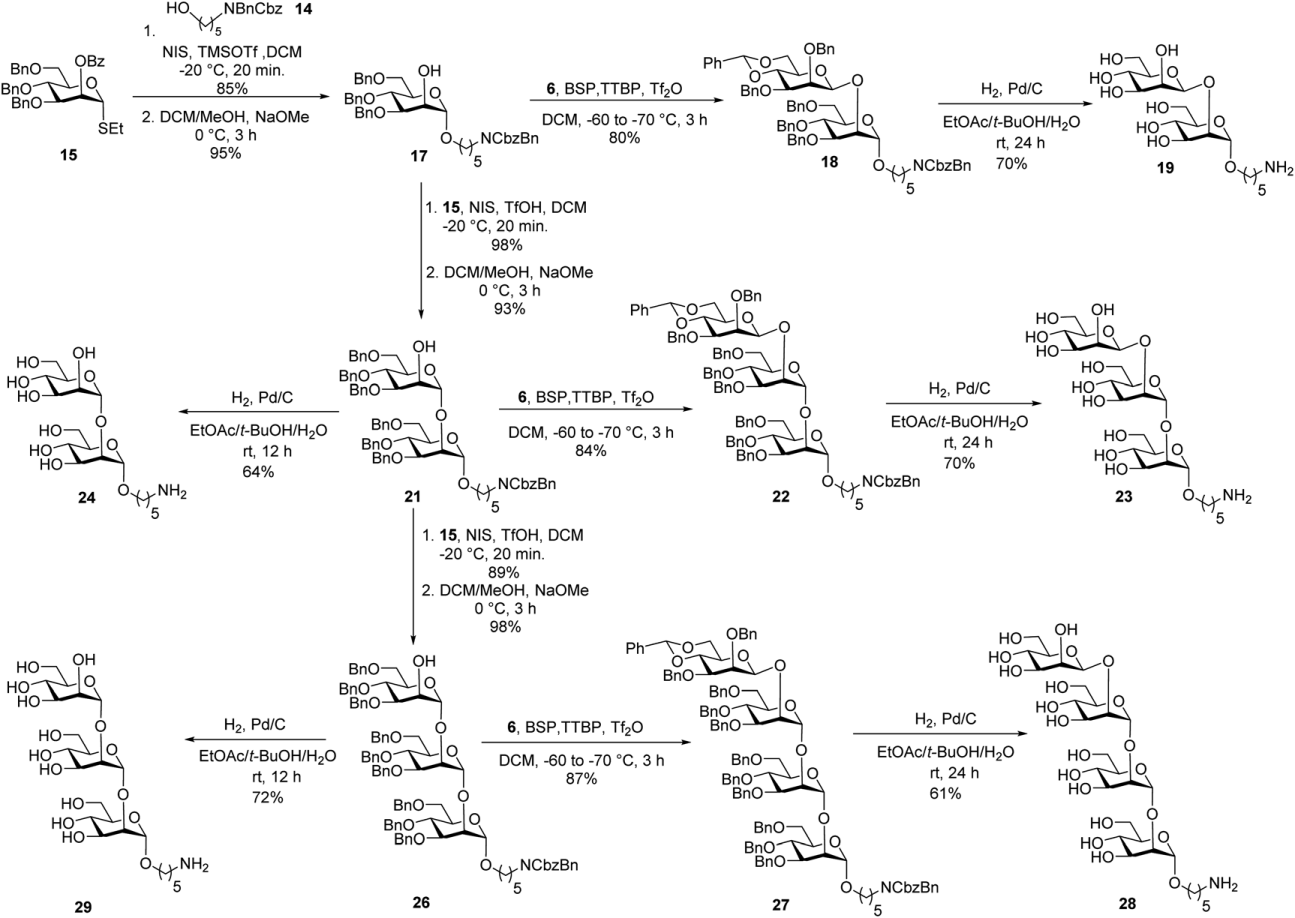
Synthesis of oligosaccharides mannans resembling the cell surface of *C. auris*.

Union of d-thiomannoside^18^15 and aminopentyl linker^19^14 promoted by NIS/TfOH afforded desired α-linked glycoside 16 in 85% yield (Scheme 2). The linker is placed in anticipation of the conjugation to a carrier protein or a microarray surface. Benzoyl ester cleavage using sodium methoxide gave differentially protected monosaccharide 17.^20^ The synthesis of β-(1→2)-linked disaccharide mannan^21^19 began with the activation of thioglycoside^22^6 by addition of 1-benzene-sulfonyl piperidine (BSP), 2,4,6-tri-*tert*-butylpyrimidine (TTBP) and triflic anhydride (Tf_2_O) at −60 °C followed by addition of acceptor 17 at −70 °C to get exclusively fully protected β-(1→2)-linked mannan 18. The newly formed β-glycosidic bond was confirmed by coupled HSQC NMR. The global deprotection^23^ of protected mannan 18 using Pd/C provided β-(1→2)-linked disaccharide 19 with 70% yield. Glycosylation of monosaccharide 17 with building block 15 using NIS/TfOH followed by cleavage of the benzoyl ester using sodium methoxide provided disaccharide 21. Hydrogenation of disaccharide 21 using Pd/C yielded 64% of deprotected disaccharide 24. For the synthesis of trisaccharide 23, disaccharide acceptor 21 was glycosylated with building block 6 using BSP, TTBP, and triflic anhydride to afford β-(1→2)-linked trisaccharide 22 in 84% yield. Hydrogenation of trisaccharide 22 furnished deprotected trisaccharide 23 in 70% yield. Synthesis of tetrasaccharide 28 began with the union of disaccharide 21 with donor 15 using NIS/TfOH, followed by debenzoylation using sodium methoxide to yield protected tetrasaccharide 26. Trisaccharide 26 was glycosylated with thioglycoside donor 6 to produce protected tetrasaccharide 27 in 87% yield. Tetrasaccharide 27 was freed from all protective groups using Pd/C to yield tetrasaccharide 28. Similarly, 26 was converted to trisaccharide 29 in 72% yield.

**Scheme 3:**
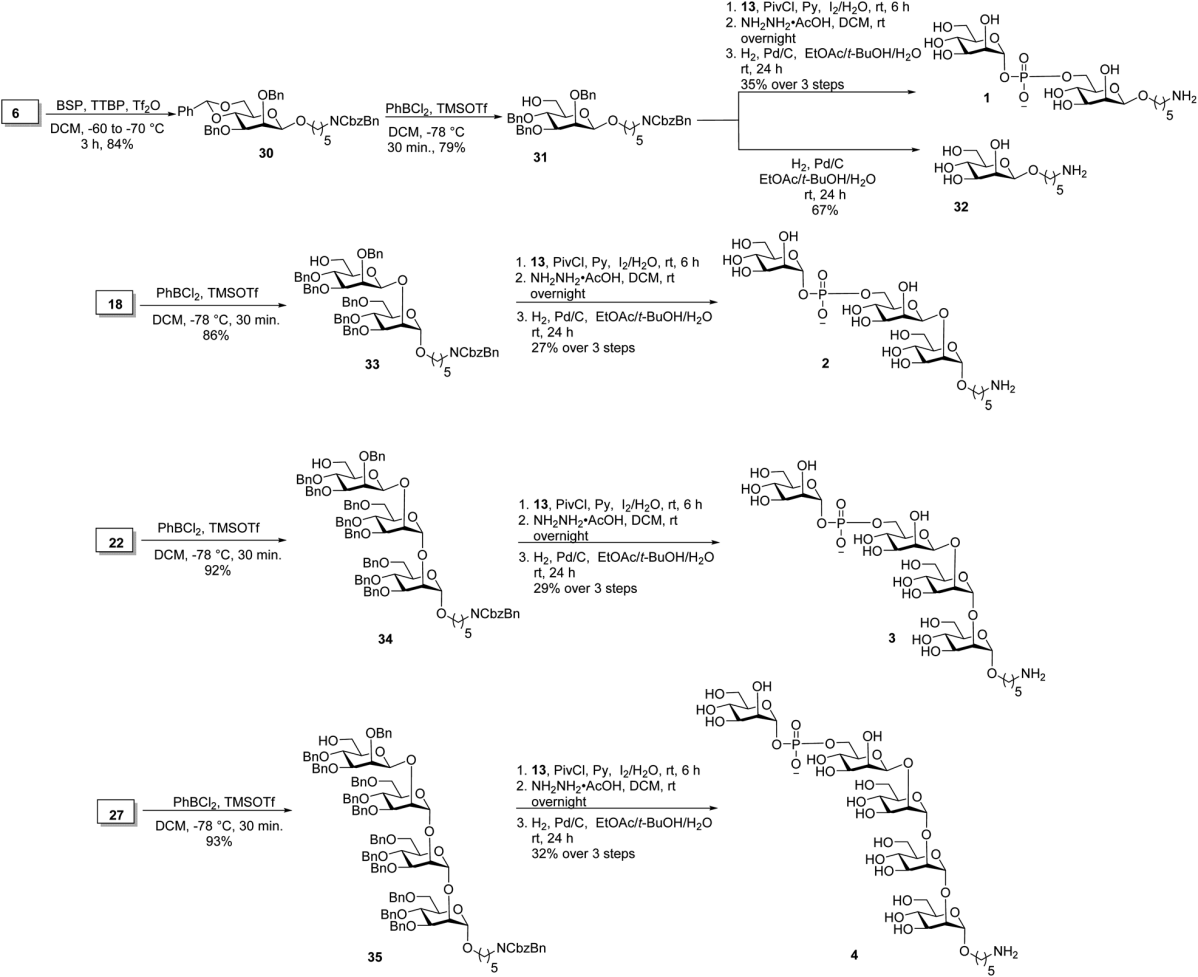
Synthesis of oligosaccharides having phosphodiester linkage.

Oligosaccharides containing a phosphodiester linkage are essential for immunological studies aimed at identifying the minimal glycan epitope (Scheme 3). Glycosylation of the aminopentyl linker with 6 using BSP, TTBP and triflic anhydride provided exclusively β-linked, fully protected monosaccharide 30 in 84% yield (confirmed by coupled HSQC NMR). The benzylidene ring in monosaccharide 30 was opened using dichlorophenyl borane and TMSOTf to yield 79% of the 6-OH containing 31. Coupling of *H*-phosphonate 13 and monosaccharide 31 using pivaloyl chloride as coupling reagent followed by iodine/H_2_O assisted oxidation of the newly formed *H*-phosphonate diester, levulinoyl (Lev) ester cleavage using hydrazine acetate and hydrogenation provided phosphate diester disaccharide 1. The stereochemistry of the newly formed linkage was confirmed by coupled HSQC NMR. For the synthesis of trisaccharide 2, the benzylidene ring in 18 was opened to obtain disaccharide 33 in 86% yield. Coupling of *H*-phosphonate 13 with disaccharide 33, followed by oxidation using iodine/H_2_O and global deprotection provided trisaccharide 2 containing a phosphodiester linkage in 27% yield over three steps. The synthesis of tetrasaccharide 3 started with benzylidene ring opening in trisaccharide 22 using dichlorophenyl borane and TMSOTf to obtain trisaccharide 34 in 92% yield. Reaction of *H*-phosphonate 13 with trisaccharide 34, oxidation of the newly formed *H*-phosphonate diester followed by global deprotection provided tetrasaccharide 3. Similarly, pentasaccharide 4, containing a phosphodiester linkage, was synthesized from tetrasaccharide 27.

Glycan microarrays are useful tools for screening plasma samples in order to identify minimal glycan epitopes.^24^ Synthetic glycans 1–4, 19, 23, 24, 28, 29 and 32 were immobilized in triplicates on glass slides to detect antibody binding to the glycans in plasma samples derived from mice three days after infection (Fig. 2).

IgM antibodies from infected mice specifically bound to structures 19, 23, 28 and 32. All these synthetic glycans contain the β-(1→2)-linked mannan. Five of six animals produced antibodies to disaccharide 19 that emerged as a vaccine lead for incorporation into a glycoconjugate. No IgM antibodies were detected against glycans 1–4 possibly be due to shielding of the phosphodiester linker. Since antibodies against the synthetic glycans were detected already after three days of infection, oligosaccharides 19, 23, 28 and 32 serve for early detection of infection.

## Conclusions

We synthesized a library of oligosaccharides resembling the cell surface mannans of *C. auris*. The challenging phosphodiester linkage was synthesized using *H*-phosphonate building block 13. Glycan array analysis of plasma from mice infected with *C. auris* for three days were screened for antibodies to the synthetic glycans. Disaccharide 19 is recognized by IgM and appears to be a promising vaccine lead for further development of a glycoconjugate vaccine candidate.

## Data availability

Data supporting this article have been uploaded as ESI.†

## Author contributions

P. H. S. conceived and supervised this project. R. K. S. synthesized all the target compounds. E. E. R. performed glycan microarray experiments. M. B. and M. G. N. provided plasma for glycan microarray experiments. R. K. S. and E. E. R. wrote the original draft of the manuscript which was edited by all authors.

## Conflicts of interest

There are no conflicts to declare.

## Supplementary Material

SC-014-D3SC01242E-s001
