# Supplementary material for: Synthesis of oligosaccharides to identify an immunologically active epitope against Candida auris infection
Source: Chem Sci. 2023 Jun 23;14(27):7559–63. doi: 10.1039/d3sc01242e (PMC10337753; doi:10.1039/d3sc01242e)
Supplement: SC-014-D3SC01242E-s001 [file SC-014-D3SC01242E-s001.pdf]

Electronic Supplementary Material (ESI) for Chemical Science.
This journal is © The Royal Society of Chemistry 2023

Synthesis of Oligosaccharides Resembling *Candida auris* Cell Surface Mannans as Basis for Anti-fungal Glycoconjugate Vaccine Development

Rajat Kumar Singh[ab], Emelie E. Reuber[ab], Mariolina Bruno[c], Mihai G. Netea[c,d] and Peter H. Seeberger*[ab]

[a.] Department of Biomolecular System, Max Planck Institute of Colloids and Interfaces, 14476 Potsdam, Germany

[b.] Institute of Chemistry and Biochemistry, Freie Universität Berlin, 14195 Berlin, Germany.

[c.] Department of Internal Medicine, Radboud Institute for Molecular Life Sciences, Radboud University Medical Center, Nijmegen, the Netherlands.

[d.] Department of Immunology and Metabolism, Life & Medical Sciences Institute (LIMES), University of Bonn, Bonn, Germany.

General Information

All the glassware were dried in the oven prior to reaction. Commercial grade solvents and reagents were used without further purification. Reactions sensitive to moisture were carried out under an atmosphere of nitrogen. NaI used in the reaction was dried at 80 °C under vacuum, sugar building blocks indicated as commercially available were purchased from GlycoUniverse GmbH. Anhydrous solvents were obtained from a solvent drying system (JCMeyer) or dried according to reported procedures. Analytical TLC was performed on Kieselgel 60 F_{254} glass (Macherey-Nagel). Spots were visualized with UV light (λ: 254 nm), sulphuric acid stain [1 mL of 3-methoxyphenol in 1 L of EtOH and 30 mL H_2SO_4] orceric ammonium molybdate stain [0.5 g $Ce(NH_4)_4(SO_4)_4{\cdot}2H_2O$, 12 g $(NH_4)_6Mo_7O_{24}{\cdot}4H_2O$ and 15 mL H_2SO_4 in 235 mL H_2O]. Flash chromatography was performed on Kieselgel 60 230-400 mesh (Sigma-Aldrich). Preparative HPLC purifications were performed with an Agilent 1200 Series or Agilent 1260 Infinity II. NMR spectra were recorded on a Varian 400 MHz spectrometer (Agilent), Ascend 400 MHz (cryoprobe, Bruker), Ascend 700 MHz (cryoprobe, Bruker) or Varian 600 MHz (Agilent) at 25 °C unless indicated otherwise. Chemical shifts (δ) are reported in parts per million (ppm) relative to the respective residual solvent peaks ($CHCl_3$: δ 7.26 in ^1H and 77.16in ^{13}C; HDO δ 4.79 in ^1H). Bidimensional and non-decoupled experiments were performed to assign identities of peaks showing relevant structural features. The following abbreviations are used to indicate peak multiplicities: s (singlet), d (doublet) dd (doublet of doublets), t (triplet), dt (doublet of triplets), td (triplet of doublets), q (quartet), p (pentet), m (multiplet). Additional descriptors b (broad signal) and app (apparent first-order multiplet) are also employed when required. Coupling constants (J) are reported in Hertz (Hz). NMR spectra were processed using MestreNova 14.1 (MestreLab Research). High-resolution mass spectra (ESI-HRMS) were recorded with a Xevo G2-XS Q-Tof (Waters).

Ethyl 3-*O*-benzyl-2-*O*-(2-napthylmethyl)-1-thio-α-D-mannopyranoside (8)

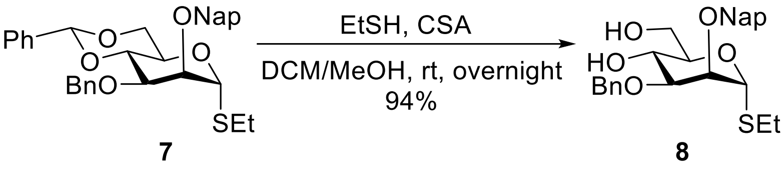

To a solution of compound **7** (2.2 g, 4.05 mmol) in DCM/MeOH (99:1, *v*/*v*) (20 mL), was added ethanethiol (1.46 mL, 20.25 mmol) and CSA (188 mg, 0.81 mmol). After stirring at rt overnight, the mixture was quenched with Et_3N and concentrated *in vacuo*. The residue was purified by silica gel column chromatography using 30% ethyl acetate in hexanes to give diol **5** (1.73 g, 94%). ^{1}H NMR (400 MHz, $CDCl_3$) δ 7.86 – 7.74 (m, 4H), 7.54 – 7.43 (m, 3H), 7.29 (dtd, *J* = 13.2, 5.3, 2.6 Hz, 5H), 5.40 (d, *J* = 1.4 Hz, 1H), 4.88 – 4.68 (m, 2H), 4.63 – 4.37 (m, 2H), 4.14 (t, *J* = 9.6 Hz, 1H), 4.05 – 3.96 (m, 1H), 3.93 – 3.83 (m, 3H), 3.68 (dd, *J* = 9.4, 3.0 Hz, 1H), 2.59 (dd, *J* = 11.2, 7.4 Hz, 2H), 1.24 (t, *J* = 7.4 Hz, 3H). ^{13}C NMR (101 MHz, $CDCl_3$) δ 137.8, 135.4, 133.3, 133.1, 128.6, 128.4, 128.1, 128.0, 127.8, 126.8, 126.3, 126.1, 126.0, 82.3, 79.9, 77.5, 77.2, 76.8, 75.8, 72.6, 72.4, 71.9, 67.5, 62.8, 25.5, 14.9. HR-ESI-MS (m/z): calculated for $C_{26}H_{30}O_5SNa$ $[M+Na]^+$: 477.1712, found: 477.1710

Ethyl 2-*O*-(2-napthylmethyl)-3,4,6-tri-*O*-benzyl-1-thio-α-D-mannopyranoside (9)

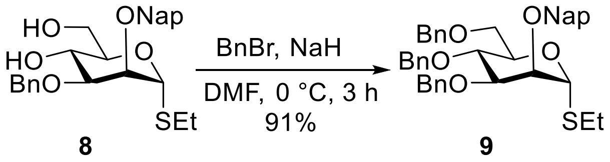

To a cooled solution of **8** (1.73 g, 3.8 mmol) in DMF (15 mL) at 0 °C were added benzyl bromide (1.35 mL, 11.4 mmol) and NaH (60% disp.) (425 mg, 10.64 mmol). After stirring for 10 h, the mixture was neutralized with H_2O and added EtOAc. The organic layer was washed five times with H_2O and brine, dried over Na_2SO_4 and concentrated *in vacuo*. The residue *was* purified by silica column chromatography using 8% ethyl acetate in hexanes to yield the desired compound **9** (2.19 g, 91%). ^{1}H NMR (400 MHz, $CDCl_3$) δ 7.90 – 7.76 (m, 4H), 7.58 (dd, *J* = 8.4, 1.6 Hz, 1H), 7.55 – 7.48 (m, 2H), 7.44 – 7.29 (m, 13H), 7.23 (dd, *J* = 7.4, 2.2 Hz, 2H), 5.51 (d, *J* = 1.5 Hz, 1H), 4.99 – 4.85 (m, 3H), 4.74 (d, *J* = 12.1 Hz, 1H), 4.68 – 4.61 (m, 2H), 4.61 – 4.55 (m, 2H), 4.24 – 4.17 (m, 1H), 4.13 (t, *J* = 9.4 Hz, 1H), 3.97 – 3.85 (m, 3H), 3.78 (dd, *J* = 10.9, 1.9 Hz, 1H), 2.75 – 2.53 (m, 2H), 1.28 (t, *J* = 7.4 Hz, 3H). ^{13}C NMR (101 MHz, $CDCl_3$) δ 138.6, 138.5, 138.4, 135.7, 133.3, 133.1, 128.5, 128.4, 128.4, 128.3, 128.1, 127.9, 127.8, 127.7, 127.7, 127.6, 126.8, 126.2, 126.1, 126.0, 82.0, 80.5, 77.5, 77.2, 76.8, 76.4, 75.2, 75.2, 73.4, 72.2, 72.2, 72.1, 69.3, 25.4, 15.1. HR-ESI-MS (m/z): calculated for $C_{40}H_{42}O_5SNa$ $[M+Na]^+$: 657.2651, found: 657.2641.

Ethyl 2-*O*-(2-napthylmethyl)-3,4,6-tri-*O*-benzyl-1-thio-α-D-mannopyranoside (10)

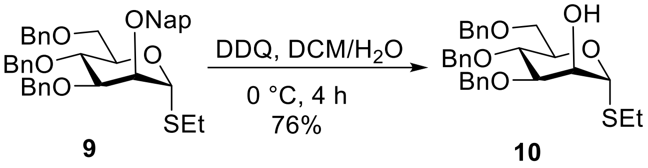

To a solution of **9** (1.6 g, 2.52 mmol) in 15 mL DCM/H_2O (7:1) at 0 °C added DDQ (743 mg, 3.28 mmol) and stirred the reaction mixture for 5 h. After completion of reaction, excess DDQ was quenched by addition of saturated sodium thiosulphate, extracted the organic phase using DCM and combined layers were dried on the Na_2SO_4. The organic phase was evaporated *in vacuo* and crude was purified by silica gel column chromatography (10% ethyl acetate in hexanes) to get the desired compound **10** (0.94 g, 76%). ^{1}H NMR (400 MHz, $CDCl_3$) δ 7.29 – 7.17 (m, 13H), 7.10 (dd, *J* = 7.3, 2.1 Hz, 2H), 5.32 (d, *J* = 1.5 Hz, 1H), 4.74 (d, *J* = 10.8 Hz, 1H), 4.60 (d, *J* = 1.0 Hz, 2H), 4.57 (s, 1H), 4.47 – 4.39 (m, 2H), 4.14 – 4.05 (m, 1H), 4.02 (dd, *J* = 3.2, 1.5 Hz, 1H), 3.87 – 3.67 (m, 3H), 3.61 (dd, *J* = 10.8, 2.0 Hz, 1H), 2.65 – 2.42 (m, 2H), 1.20 (t, *J* = 7.4 Hz, 3H). ^{13}C NMR (101 MHz, $CDCl_3$) δ 138.4, 138.3, 137.8, 128.7, 128.5, 128.4, 128.2, 128.1, 128.0, 128.0, 127.8, 127.7, 83.4, 80.6, 77.5, 77.2, 76.8, 75.3, 74.6, 73.5, 72.2, 71.5, 70.0, 68.9, 25.0, 15.0. HR-ESI-MS (m/z): calculated for $C_{29}H_{34}O_5SNa$ [M+Na]$^+$: 517.2025, found: 517.2019.

Ethyl 2-*O*-levulinoyl-3,4,6-tri-*O*-benzyl-1-thio-α-D-mannopyranoside (11)

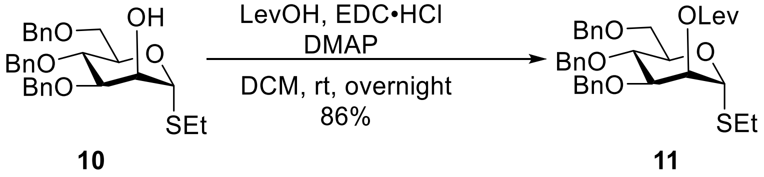

To a solution of compound **10** (2.0 g, 4.04 mmol) in DCM (20 mL) were added 4-dimethylaminopyridine (98 mg, 0.81 mmol), levulinic acid (617 µL, 6.06 mmol) and EDC•HCl (1.16 g, 6.06 mmol). The mixture was stirred overnight at rt, washed with saturated aqueous $NaHCO_3$ and brine, the organic layer was dried over Na_2SO_4, filtered and concentrated. The residue was purified by silica gel column chromatography using 30% ethyl acetate in hexanes to afford compound **11** (2.06 g, 86%) as a white solid. ^{1}H NMR (400 MHz, $CDCl_3$) δ 7.39 – 7.29 (m, 14H), 7.20 (dd, *J* = 7.3, 2.1 Hz, 2H), 5.46 (t, *J* = 2.1 Hz, 1H), 5.33 (d, *J* = 1.5 Hz, 1H), 4.88 (d, *J* = 10.8 Hz, 1H), 4.70 (dd, *J* = 11.6, 3.8 Hz, 2H), 4.57 – 4.48 (m, 3H), 4.24 – 4.15 (m, 1H), 3.98 – 3.90 (m, 2H), 3.86 (dd, *J* = 10.8, 4.2 Hz, 1H), 3.71 (dd, *J* = 10.8, 2.0 Hz, 1H), 2.81 – 2.71 (m, 4H), 2.72 – 2.53 (m, 2H), 2.17 (s, 3H), 1.30 (t, *J* = 7.4 Hz, 3H). ^{13}C NMR (101 MHz, $CDCl_3$) δ 206.5, 172.1, 138.5, 138.3, 137.9, 128.5, 128.4, 128.3, 128.0, 127.9, 127.8, 127.7, 82.4, 78.6, 77.5, 77.2, 76.8, 75.3, 74.6, 73.5, 71.9, 71.8, 70.8, 68.9, 38.1, 29.9, 28.3, 25.6, 15.0. HR-ESI-MS (m/z): calculated for $C_{34}H_{40}O_7SNa$ [M+Na]$^+$: 615.2392, found: 615.2380.

2-Levulinoyl-3,4,6-tri-*O*-benzyl-α-D-mannopyranosyl hydrogen phosphate triethylammonium salt (13)

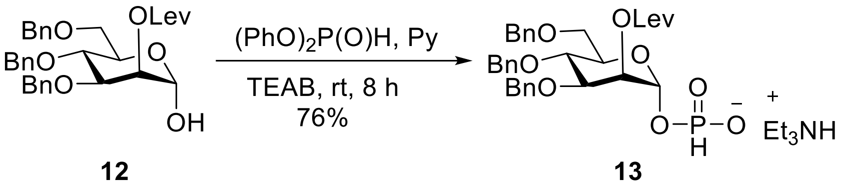

To a solution of hemiacetal **12** (150 mg, 0.273 mmol) in anhydrous pyridine (5mL), diphenyl phosphite (260 µL) was added. The mixture was stirred at rt for 2 h. TEAB buffer (20 mL) was added and reaction mixture was stirred for additional 2 h. The reaction was diluted with DCM (20 mL) and washed with TEAB buffer. The organic layer was dried over Na_2SO_4, concentrated and purified by silica gel column chromatography using 5% methanol in DCM which was neutralized with triethylamine to afford the *H*-phosphonate **13** (149 mg, 76%) as a light yellow syrup. 1H NMR (400 MHz, CDCl3) δ 11.98 (s, 1H), 7.36 – 7.21 (m, 13H), 7.20 – 7.11 (m, 2H), 5.62 – 5.53 (m, 1H), 5.40 (t, J = 3.1 Hz, 1H), 4.84 (dt, J = 11.1, 3.3 Hz, 1H), 4.63 (ddt, J = 12.5, 7.6, 3.6 Hz, 2H), 4.48 (tdd, J = 12.3, 7.4, 3.1 Hz, 3H), 4.14 – 3.99 (m, 2H), 3.87 (td, J = 9.9, 4.2 Hz, 1H), 3.78 (td, J = 7.5, 3.6 Hz, 1H), 3.71 – 3.62 (m, 1H), 2.99 (q, 7H), 2.75 – 2.58 (m, 4H), 2.08 (s, 3H), 1.27 (t, J = 3.3 Hz, 12H). ^{13}C NMR (101 MHz, $CDCl_3$) δ 206.4, 171.8, 138.6, 138.3, 138.1, 129.4, 129.2, 129.2, 128.3, 128.3, 128.2, 128.2, 128.0, 127.9, 127.8, 127.8, 127.7, 127.6, 127.6, 127.5, 115.7, 93.3, 93.2, 77.8, 77.6, 77.5, 77.3, 77.2, 77.0, 76.9, 74.9, 74.1, 73.4, 72.4, 71.7, 69.4, 69.4, 68.9, 45.6, 38.0, 29.8, 28.2, 8.6. ^{31}P NMR (162 MHz, $CDCl_3$) δ -0.2. HR-ESI-MS (m/z): calculated for $C_{38}H_{53}NO_{10}H$ $[M+H]^+$: 714.3407, found: 714.3405.

***N*-(Benzyl)benzyloxycarbonyl-5-aminopentyl 2-benzoyl-3,4,6-*O*-tri-benzyl-α-D-mannopyranoside (16)**

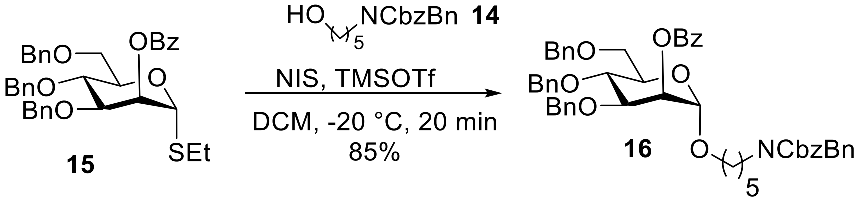

To a solution of donor **15** (1.161 g, 1.93 mmol) and *N*-benzyloxycarbonyl-*N*-benzyl-5-aminopentanol **14** (0.952 g, 2.895 mmol) in DCM (10 mL) were added 4 Å molecular sieves. After ~30 minutes, the mixture was cooled to 0 °C and *N*-iodosuccinimide (0.738 g, 3.281 mmol) and TMSOTf (85 µL, 0.965 mmol) were added. After TLC analysis indicated complete consumption of the starting material (~20 min), the reaction was quenched with Et_3N (2 mL) and the mixture was diluted with DCM. After filtration over Celite®, the mixture was washed with 10% aqueous $Na_2S_2O_3$, saturated aqueous $NaHCO_3$ and brine, dried over Na_2SO_4, filtered and concentrated *in vacuo*. The crude was purified by silica gel column chromatography using 10% ethyl acetate in hexanes to afford the title product **16** as the sole isomer (1.412 g, 85%). ^1H NMR (400 MHz, $CDCl_3$) δ 8.09 – 8.05 (m, 2H), 7.55 (t, *J* = 7.4 Hz, 1H), 7.38 (dt, *J* = 8.3, 2.1 Hz, 6H), 7.35 – 7.21 (m, 18H), 7.21 – 7.14 (m, 3H), 5.61 – 5.57 (m, 1H), 5.18 (d, *J* = 21.0 Hz, 2H), 4.92 (d, *J* = 12.0 Hz, 1H), 4.86 (d, *J* = 10.7 Hz, 1H), 4.79 (d, *J* = 11.3 Hz, 1H), 4.73 (d, *J* = 12.0 Hz, 1H), 4.59 – 4.47 (m, 5H), 4.10 – 4.06 (m, 2H), 3.91 – 3.86 (m, 1H), 3.83 (d, *J* = 10.4 Hz, 1H), 3.76 (d, *J* = 10.7 Hz, 1H), 3.71 – 3.56 (m, 1H), 3.44 – 3.31 (m, 1H), 3.23 (dt, *J* = 44.0, 7.6 Hz, 2H), 1.56 – 1.46 (m, 4H), 1.34 – 1.19 (m, 2H). ^{13}C NMR (101 MHz, $CDCl_3$) δ 165.9, 138.6, 138.4, 138.2, 138.1, 136.9, 133.3, 130.1, 130.0, 128.7, 128.6, 128.6, 128.5, 128.5, 128.4, 128.3, 128.2, 128.1, 128.0, 128.0, 127.9, 127.8, 127.7, 127.7, 127.6, 127.4, 127.3, 97.9, 78.5, 77.4, 77.2,

76.9, 75.5, 74.5, 73.6, 71.7, 69.2, 67.8, 67.3, 50.6, 50.3, 47.2, 46.2, 29.2, 28.1, 27.6, 23.5. HR-ESI-MS (m/z): calculated for $C_{54}H_{57}NO_9Na$ $[M+Na]^+$: 886.3931, found: 886.3919.

_N_-(Benzyl)benzyloxycarbonyl-5-aminopentyl 4,6-_O_-benzylidene-2,3-_O_-di-benzyl-β-D-mannopyranosyl-(1→2)-3,4,6-_O_-tri-benzyl-α-D-mannopyranoside (18)

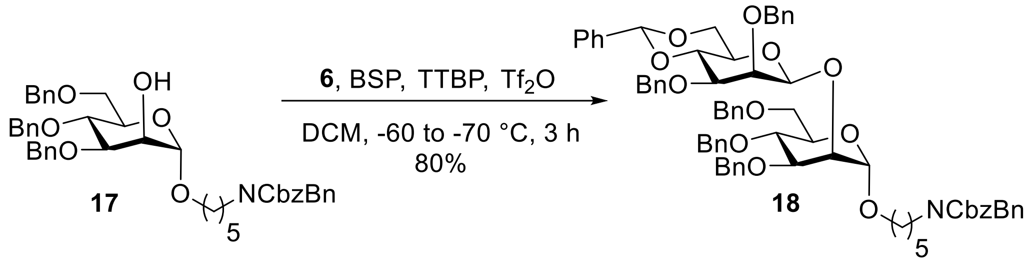

To a solution of donor **6** (778 mg, 1.58 mmol) in DCM (8 mL) was added 4 Å molecular sieves and the solution was stirred for 30 min. BSP (330 mg, 1.58 mmol) and TTBP (522 mg, 2.1 mmol) were added to the reaction mixture at -60 °C and stirred the reaction for 30 min. After stirring for 30 min, Tf_2O (441 μL, 2.63 mmol) was added and stirred the mixture for 30 min. The reaction mixture was brought to -70 °C and acceptor **17** (400 mg, 0.526 mmol) was added. After stirring for 2 h, reaction was quenched by adding Et_3N (0.5 mL) and filtered through Celite®. Organic layer was washed with saturated aqueous sodium bicarbonate, dried over Na_2SO_4 and evaporated *in vacuo*. The crude product was purified by silica gel column chromatography (10% ethyl acetate in hexanes) to get the disaccharide **18** (504 mg, 80%). ^1H NMR (400 MHz, $CDCl_3$) δ 7.56 – 7.50 (m, 4H), 7.49 – 7.45 (m, 2H), 7.43 – 7.35 (m, 5H), 7.35 – 7.29 (m, 12H), 7.28 – 7.20 (m, 14H), 7.12 – 7.06 (m, 2H), 5.61 (s, 1H), 5.23 – 5.16 (m, 2H), 5.11 (d, *J* = 11.6 Hz, 1H), 4.86 (d, *J* = 11.5 Hz, 3H), 4.79 (d, *J* = 10.8 Hz, 1H), 4.65 (d, *J* = 3.7 Hz, 3H), 4.62 – 4.54 (m, 2H), 4.53 – 4.48 (m, 2H), 4.46 (d, *J* = 12.1 Hz, 1H), 4.31 (d, *J* = 10.8 Hz, 1H), 4.28 – 4.20 (m, 3H), 4.04 (d, *J* = 3.2 Hz, 1H), 3.94 – 3.81 (m, 3H), 3.76 – 3.63 (m, 4H), 3.59 (d, *J* = 10.3 Hz, 1H), 3.41 – 3.16 (m, 5H), 1.51 – 1.42 (m,40H), 0.96 – 0.90 (m, 2H). ^{13}C NMR (101 MHz, $CDCl_3$) δ 138.8, 138.7, 138.3, 138.0, 137.6, 134.5, 129.8, 129.0, 128.9, 128.8, 128.7, 128.6, 128.5, 128.3, 128.3, 128.2, 128.2, 128.2, 128.1, 128.1, 128.0, 127.9, 127.8, 127.8, 127.7, 127.6, 127.6, 127.5, 127.5, 127.4, 127.2, 126.1, 101.4, 100.4, 97.3, 78.3, 78.2, 77.4, 77.3, 77.0, 76.7, 76.1, 75.1, 74.5, 74.2, 73.4, 73.3, 72.9, 71.8, 71.4, 70.6, 70.5, 68.9, 68.5, 67.6, 67.2, 50.2, 47.0, 46.1, 45.3, 32.0, 30.2, 30.1, 29.7, 29.4, 29.2, 28.0, 27.7, 27.5, 23.4, 22.7, 21.5, 14.2. HR-ESI-MS (m/z): calculated for $C_{74}H_{79}NO_{13}Na$ $[M+Na]^+$: 1212.5449, found: 1212.5438.

5-Aminopentyl β-D-mannopyranosyl-(1→2)-α-D-mannopyranoside (19)

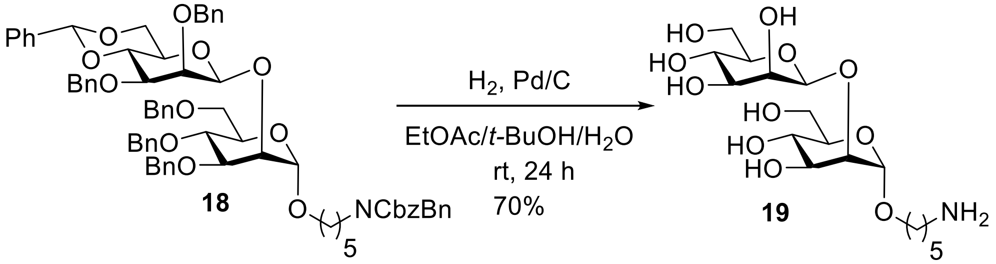

The disaccharide **18** (20 mg, 16.8 μmol) was dissolved in EtOAc/*t*-BuOH/H2O (2/1/1, *v*/*v*/*v*, 3 mL) and added Pd/C (40 mg). The reaction mixture was stirred overnight at rt under a hydrogen atmosphere (1

atm, balloon). The crude material was purified by HPLC (Hypercarb column, 150 x 10 mm, H_2O (0.1% formic acid) isocratic (5 min), linear gradient to 40% ACN (35 min), linear gradient to 100% ACN (10 min)) and lyophilized to obtain pure disaccharide **19** (5 mg, 70%) as a white solid. ^{1}H NMR (700 MHz, D_2O) δ 4.93 (d, *J* = 1.7 Hz, 1H), 4.72 (d, *J* = 1.0 Hz, 1H), 4.10 (dd, *J* = 3.4, 1.7 Hz, 1H), 4.00 (dd, *J* = 3.4, 0.9 Hz, 1H), 3.88 (dd, *J* = 12.3, 2.3 Hz, 1H), 3.83 (dd, *J* = 12.3, 2.2 Hz, 1H), 3.77 (dd, *J* = 9.7, 3.4 Hz, 1H), 3.75 – 3.63 (m, 4H), 3.62 – 3.56 (m, 2H), 3.54 – 3.48 (m, 2H), 3.35 – 3.30 (m, 1H), 2.96 (t, *J* = 7.6 Hz, 2H), 1.69 – 1.56 (m, 4H), 1.47 – 1.35 (m, 2H). ^{13}C NMR (176 MHz, D_2O) δ 170.9, 98.5, 97.4, 77.1, 76.3, 72.8, 72.7, 70.7, 69.9, 67.5, 66.9, 66.6, 61.0, 60.5, 39.3, 28.0, 26.5, 22.4. HR-ESI-MS (m/z): calculated for $C_{17}H_{33}NO_{11}H$ $[M+H]^+$: 428.2132, found: 428.2087.

_N_-(Benzyl)benzyloxycarbonyl-5-aminopentyl 2-benzoyl-3,4,6-_O_-tri-benzyl-α-D-mannopyranosyl-(1→2)-3,4,6-_O_-tri-benzyl-α-D-mannopyranoside (20)

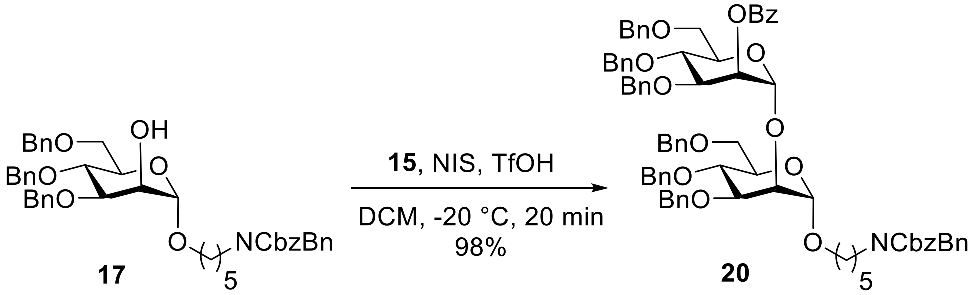

Acceptor **17** (913 mg, 1.20 mmol) and thioglycoside donor **15** (1.077 g, 1.8 mmol) were mixed, co-evaporated with toluene (3 x 10 mL) and dried under high vacuum for 2 h. The freshly activated 4 Å molecular sieves were added, dissolved in anhydrous DCM (12 mL) under a nitrogen atmosphere and stirred for 30 min at rt. The mixture was cooled to 0 °C, to this stirred suspension, NIS (0.413 g, 1.92 mmol) and TfOH (52 µL, 0.6 mmol) were slowly added. After stirring at rt for 20 min, diluted with DCM (10 mL), quenched with Et_3N, warmed to rt and filtered. The filtrate was washed with 10% aqueous $Na_2S_2O_3$, saturated aqueous $NaHCO_3$ and brine. The combined organic layer was dried over Na_2SO_4, filtered and evaporated *in vacuo*. The crude product was purified by silica column chromatography using 13% ethyl acetate in hexanes to afford the desired protected disaccharide **20** (1.541 g, 98%). ^{1}H NMR (600 MHz, $CDCl_3$) δ 8.07 (dd, *J* = 8.3, 1.4 Hz, 2H), 7.60 – 7.52 (m, 1H), 7.41 – 7.32 (m, 10H), 7.31 – 7.24 (m, 19H), 7.24 – 7.12 (m, 13H), 5.78 (dd, *J* = 3.1, 2.0 Hz, 1H), 5.21 – 5.11 (m, 3H), 4.89 – 4.83 (m, 3H), 4.76 (d, *J* = 11.1 Hz, 1H), 4.74 – 4.63 (m, 4H), 4.55 (dd, *J* = 11.5, 4.5 Hz, 2H), 4.52 – 4.49 (m, 3H), 4.48 – 4.43 (m, 2H), 4.15 – 4.07 (m, 1H), 4.06 – 4.01 (m, 2H), 3.98 (s, 1H), 3.92 – 3.80 (m, 3H), 3.80 – 3.67 (m, 4H), 3.55 (dd, *J* = 26.9, 8.5 Hz, 1H), 3.28 – 3.13 (m, 3H), 1.47 (ddd, *J* = 31.6, 15.5, 9.0 Hz, 4H), 1.26 – 1.14 (m, 2H). ^{13}C NMR (151 MHz, $CDCl_3$) δ 165.6, 156.8, 138.6, 138.5, 138.4, 138.2, 138.0, 133.2, 130.1, 130.1, 128.7, 128.6, 128.6, 128.5, 128.4, 128.4, 128.4, 128.3, 128.3, 128.0, 128.0, 127.9, 127.8, 127.6, 127.6, 127.6, 127.5, 127.4, 127.3, 99.7, 98.8, 79.9, 78.3, 77.4, 77.2, 76.9, 75.4, 75.3, 74.8, 74.5, 73.5, 73.4, 72.3, 72.1, 71.9, 71.8, 69.4, 69.4, 69.2, 67.6, 67.3, 50.6, 50.3, 47.2, 46.2, 29.3, 28.1, 27.6, 23.5. HR-ESI-MS (m/z): calculated for $C_{81}H_{85}NO_{14}Na$ $[M+Na]^+$: 1318.5868, found: 1318.5861.

_N_-(Benzyl)benzyloxycarbonyl-5-aminopentyl 3,4,6-_O_-tri-benzyl-α-D-mannopyranosyl-(1→2)-3,4,6-_O_-tri-benzyl-α-D-mannopyranoside (21)

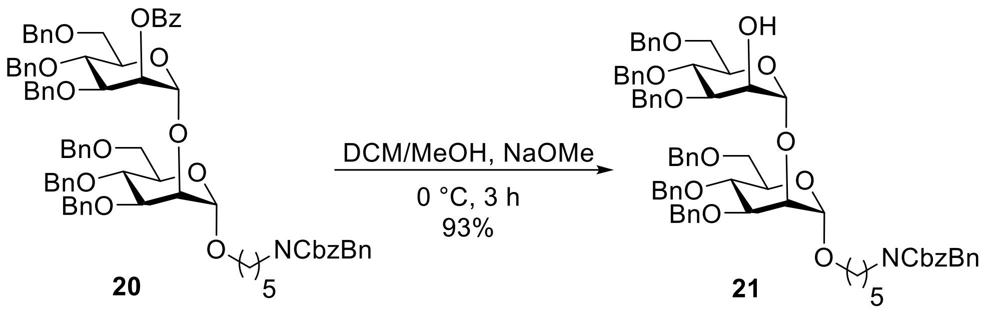

To a stirred solution of **20 (**1.52 g**,** 1.17 mmol**)** in MeOH/DCM (2:1, 30 mL) in an ice bath was added NaOMe (3.98 mL, 1 M in MeOH) and stirred the reaction mixture for 3 h at rt. After completion of the reaction, the reaction mixture was neutralized by addition of Amberlite® IR120 H^+, filtered and concentrated under reduced pressure. The crude product was purified by silica column chromatography using 15% ethyl acetate in hexanes to afford the desired disaccharide **21** (1.3 g, 93%). ^{1}H NMR (400 MHz, $CDCl_3$) δ 7.39 – 7.14 (m, 40H), 5.20 – 5.11 (m, 3H), 4.90 – 4.78 (m, 3H), 4.72 – 4.62 (m, 3H), 4.61 – 4.54 (m, 3H), 4.54 – 4.44 (m, 5H), 4.12 (t, *J* = 2.5 Hz, 1H), 4.00 (s, 1H), 3.98 – 3.93 (m, 1H), 3.92 – 3.90 (m, 1H), 3.88 (d, *J* = 3.3 Hz, 1H), 3.87 – 3.84 (m, 1H), 3.84 – 3.76 (m, 2H), 3.75 – 3.66 (m, 3H), 3.51 (d, *J* = 9.1 Hz, 1H), 3.27 – 3.11 (m, 3H), 1.53 – 1.36 (m, 4H), 1.26 – 1.10 (m, 2H). ^{13}C NMR (101 MHz, $CDCl_3$) δ 138.75, 138.50, 138.44, 138.38, 138.11, 128.68, 128.61, 128.57, 128.50, 128.46, 128.42, 128.16, 128.07, 128.01, 127.98, 127.95, 127.84, 127.82, 127.75, 127.67, 127.57, 127.51, 101.21, 98.90, 80.14, 79.97, 77.48, 77.36, 77.16, 76.84, 75.34, 75.16, 74.95, 74.55, 73.52, 73.43, 72.38, 72.27, 72.00, 71.65, 69.45, 69.33, 68.65, 67.59, 67.28, 50.30, 47.22, 46.26, 29.36, 23.53. HR-ESI-MS (m/z): calculated for $C_{74}H_{81}NO_{13}Na$ $[M+Na]^+$: 1214.5606, found: 1214.50.1.

***N*-(Benzyl)benzyloxycarbonyl-5-aminopentyl 2,3-*O*-di-benzyl-4,6-*O*-benzylidene-β-D-mannopyranosyl-(1→2)-3,4,6-*O*-tri-benzyl-α-D-mannopyranosyl-(1→2)-3,4,6-*O*-tri-benzyl-α-D-mannopyranoside (22)**

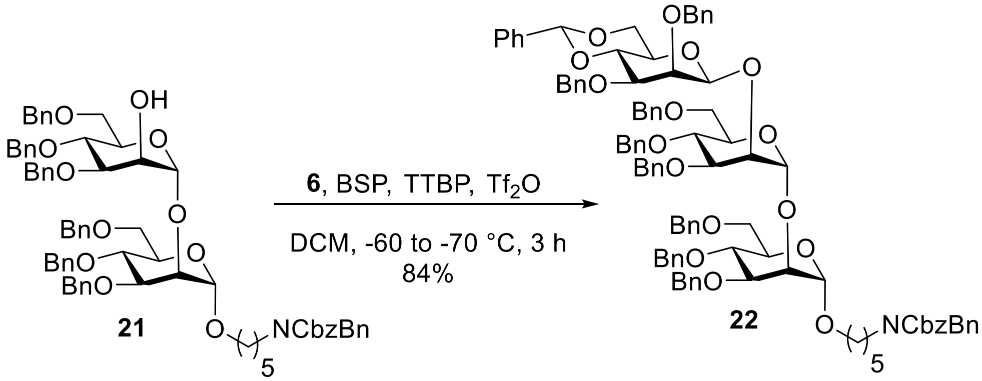

To a solution of donor **6** (248 mg, 0.503 mmol) in DCM (8 mL) was added 4 Å molecular sieves and the solution was stirred for 30 min under nitrogen atmosphere. BSP (105 mg, 0.503 mmol) and TTBP (167 mg, 0.671 mmol) were added to the reaction mixture at -60 °C and the reaction was stirred for 30 min. After stirring for 30 min, Tf_2O (140 µL, 0.839 mmol) was added and the mixture was stirred for another 30 min. The reaction mixture was brought to -70 °C and acceptor **21** (200 mg, 0.168 mmol, in 2 mL of DCM) was added. After stirring for 2 h, the reaction was quenched by adding Et_3N (0.5 mL) and filtered through Celite®. The organic layer was washed with saturated aqueous sodium bicarbonate, dried over Na_2SO_4 and

evaporated *in vacuo*. The crude product was purified by silica gel column chromatography (10% ethyl acetate in hexanes) to obtained the trisaccharide **22** (230 mg, 84%). ^{1}H NMR (600 MHz, $CDCl_3$) δ 7.55 – 7.51 (m, 4H), 7.47 – 7.43 (m, 2H), 7.43 – 7.37 (m, 8H), 7.37 – 7.19 (m, 37H), 7.15 – 7.11 (m, 4H), 7.09 (d, *J* = 7.3 Hz, 1H), 5.60 (s, 1H), 5.22 (d, *J* = 19.8 Hz, 2H), 5.14 (d, *J* = 2.8 Hz, 1H), 5.05 (d, *J* = 11.7 Hz, 1H), 4.93 – 4.87 (m, 2H), 4.83 (dd, *J* = 11.6, 6.5 Hz, 2H), 4.77 – 4.68 (m, 3H), 4.66 – 4.57 (m, 6H), 4.55 – 4.50 (m, 3H), 4.46 (d, *J* = 12.1 Hz, 1H), 4.31 – 4.20 (m, 3H), 4.14 (dd, *J* = 21.0, 11.4 Hz, 2H), 4.00 (dd, *J* = 7.5, 3.1 Hz, 1H), 3.98 – 3.89 (m, 2H), 3.88 – 3.76 (m, 5H), 3.76 – 3.66 (m, 4H), 3.64 – 3.48 (m, 1H), 3.33 (dd, *J* = 9.9, 3.2 Hz, 1H), 3.31 – 3.14 (m, 3H), 3.02 (s, 1H), 1.59 – 1.41 (m, 4H), 1.30 – 1.18 (m, 2H). ^{13}C NMR (151 MHz, $CDCl_3$) δ 156.8, 156.3, 138.9, 138.9, 138.7, 138.3, 138.1, 137.9, 128.9, 128.7, 128.7, 128.6, 128.6, 128.6, 128.5, 128.4, 128.4, 128.4, 128.4, 128.3, 128.3, 128.3, 128.2, 128.2, 128.1, 128.1, 128.0, 128.0, 127.9, 127.8, 127.8, 127.7, 127.7, 127.6, 127.6, 127.6, 127.5, 127.5, 127.4, 127.4, 127.3, 126.2, 101.5, 100.1, 99.0, 80.4, 78.5, 77.8, 77.4, 77.3, 77.2, 77.1, 76.9, 76.3, 75.4, 75.2, 75.0, 74.8, 74.3, 73.6, 73.4, 73.4, 73.3, 73.0, 71.8, 71.7, 71.6, 71.4, 69.5, 69.3, 68.6, 67.5, 67.3, 67.2, 50.6, 50.3, 47.2, 46.2, 31.6, 30.3, 29.8, 29.3, 28.0, 27.6, 23.5, 23.5. HR-ESI-MS (m/z): calculated for $C_{101}H_{107}NO_{18}Na$ $[M+Na]^+$: 1644.7386, found: 1644.7379.

5-Aminopentyl β-D-mannopyranosyl-(1→2)-α-D-mannopyranosyl-(1→2)-α-D-mannopyranoside (23)

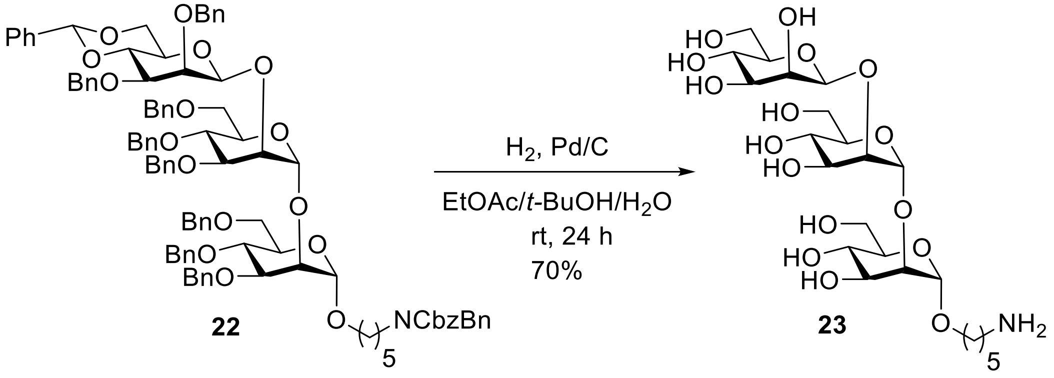

Trisaccharide **22** (12 mg, 7.3 µmol) was dissolved in a mixture of EtOAc/*t*-BuOH/H_2O (2/1/1, *v*/*v*/*v,* 2 mL) and Pd/C (60 mg) was added to the solution. After stirring for 24 h under a hydrogen atmosphere (1 atm, balloon), the mixture was filtered through a PTFE filter (0.45 µm pore size) and concentrated. The crude material was purified by HPLC (Hypercarb column, 150 x 10 mm, H_2O (0.1% formic acid) isocratic (5 min), linear gradient to 40% ACN (35 min), linear gradient to 100% ACN (10 min)) and lyophilized to obtain pure trisaccharide **23** (3 mg, 70%). ^{1}H NMR (700 MHz, D_2O) δ 5.09 (d, *J* = 1.8 Hz, 1H), 5.04 (d, *J* = 1.7 Hz, 1H), 4.75 (d, *J* = 1.0 Hz, 1H), 4.25 (dd, *J* = 3.4, 1.8 Hz, 1H), 3.99 (dd, *J* = 3.3, 0.9 Hz, 1H), 3.92 (dd, *J* = 3.5, 1.7 Hz, 1H), 3.88 (dd, *J* = 12.3, 2.3 Hz, 1H), 3.86 – 3.80 (m, 4H), 3.74 – 3.67 (m, 5H), 3.65 – 3.62 (m, 2H), 3.60 (dd, *J* = 9.6, 3.3 Hz, 1H), 3.58 – 3.54 (m, 1H), 3.54 – 3.48 (m, 2H), 3.34 (ddd, *J* = 9.5, 6.7, 2.3 Hz, 1H), 2.95 (t, *J* = 7.6 Hz, 2H), 1.69 – 1.55 (m, 4H), 1.47 – 1.34 (m, 2H). ^{13}C NMR (176 MHz, D_2O) δ 171.0, 100.1, 98.4, 98.0, 78.8, 76.9, 76.3, 73.3, 72.7, 72.7, 70.7, 70.1, 69.6, 67.4, 67.0, 66.9, 66.6, 60.9, 60.8, 60.7, 39.3, 27.9, 26.4, 22.4. HR-ESI-MS (m/z): calculated for $C_{23}H_{43}NO_{16}H$ $[M+H]^+$: 590.2660, found: 590.2660.

5-Aminopentyl α-D-mannopyranosyl-(1→2)-α-D-mannopyranoside (24)

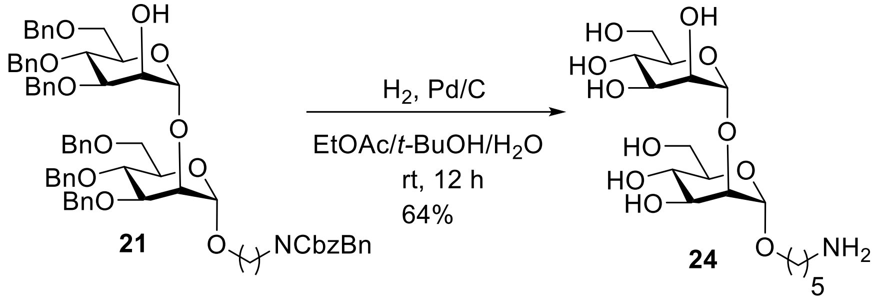

Disaccharide **21** (2.1 mg, 17.6 µmol) was dissolved in EtOAc/*t*-BuOH/H_2O (2/1/1, *v/v/v,* 3 mL) and Pd/C (40 mg) was added to the solution. After stirring overnight under a hydrogen atmosphere (1 atm, balloon), the mixture was filtered through a PTFE filter (0.45 µm pore size) and concentrated. The crude material was purified by HPLC (Hypercarb column, 150 x 10 mm, H_2O (0.1% formic acid) isocratic (5 min), linear gradient to 40% ACN (35 min), linear gradient to 100% ACN (10 min)) and lyophilized to obtain pure disaccharide **24** (4.8 mg, 64%). ^1H NMR (600 MHz, D_2O) δ 5.06 (d, *J* = 1.7 Hz, 1H), 4.97 (d, *J* = 1.8 Hz, 1H), 4.03 (dd, *J* = 3.4, 1.8 Hz, 1H), 3.91 (dd, *J* = 3.5, 1.7 Hz, 1H), 3.88 – 3.83 (m, 3H), 3.80 (dd, *J* = 9.7, 3.4 Hz, 1H), 3.76 – 3.61 (m, 5H), 3.59 – 3.54 (m, 2H), 3.52 – 3.48 (m, 1H), 2.98 – 2.93 (m, 2H), 1.70 – 1.54 (m, 4H), 1.47 – 1.34 (m, 2H). ^{13}C NMR (101 MHz, D_2O) δ 102.3, 98.0, 78.7, 73.2, 72.7, 70.2, 70.2, 69.9, 67.5, 66.9, 66.9, 61.1, 60.9, 39.3, 27.9, 26.5, 22.4. HR-ESI-MS (m/z): calculated for $C_{17}H_{33}NO_{11}H$ $[M+H]^+$: 428.2132, found: 428.2128.

***N*-(Benzyl)benzyloxycarbonyl-5-aminopentyl 2-benzoyl-3,4,6-*O*-tri-benzyl-α-D-mannopyranosyl-(1→2)-3,4,6-*O*-tri-benzyl-α-D-mannopyranosyl-(1→2)-3,4,6-*O*-tri-benzyl-α-D-mannopyranoside (25)**

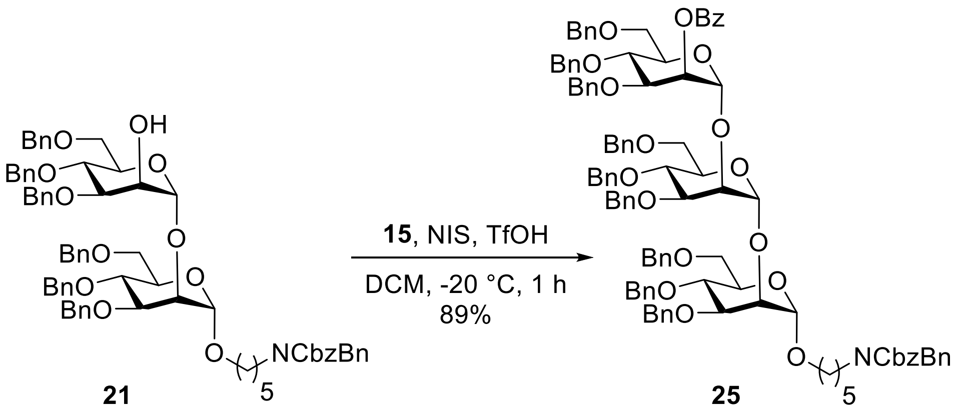

Acceptor **21** (1.2 g, 1.01 mmol) and thioglycoside donor **15** (907 mg, 1.52 mmol) were mixed, co-evaporated with toluene (3 x 10 mL) and dried under vacuum for 2 h. The mixture was dissolved in anhydrous DCM (25 mL), freshly activated 4 Å molecular sieves were added under a nitrogen atmosphere and stirred for 30 min at rt. The mixture was cooled to -20 °C, to this stirred suspension, NIS (363 mg; 1.62 mmol) and TfOH (44 µL, 0.505 mmol) were slowly added. The mixture was stirred at -20 °C for 1 h, diluted with DCM (20 mL), quenched with Et_3N, warmed to rt and 4 Å molecular sieves were filtered. The filtrate was washed with 10% aqueous $Na_2S_2O_3$, saturated aqueous $NaHCO_3$ and brine. The combined organic

layer was dried over Na_2SO_4, filtered and evaporated *in vacuo*. The crude product was purified by silica gel column chromatography using 15% ethyl acetate in hexanes to afford protected trisaccharide **25** (1.569 g, 89%). ^1H NMR (600 MHz, $CDCl_3$) δ 8.10 – 8.06 (m, 2H), 7.57 (tt, *J* = 7.4, 1.3 Hz, 1H), 7.40 – 7.36 (m, 2H), 7.36 – 7.31 (m, 6H), 7.31 – 7.27 (m, 16H), 7.26 – 7.22 (m, 10H), 7.22 – 7.19 (m, 5H), 7.19 – 7.14 (m, 11H), 7.13 – 7.07 (m, 1H), 7.06 – 7.00 (m, 1H), 5.75 (t, *J* = 2.3 Hz, 1H), 5.22 (d, *J* = 2.0 Hz, 1H), 5.16 (d, *J* = 17.3 Hz, 2H), 5.11 (d, *J* = 2.0 Hz, 1H), 4.89 – 4.78 (m, 4H), 4.76 (d, *J* = 11.2 Hz, 1H), 4.64 (dt, *J* = 24.8, 12.5 Hz, 4H), 4.56 – 4.55 (m, 2H), 4.53 (d, *J* = 4.7 Hz, 1H), 4.53 – 4.49 (m, 4H), 4.49 – 4.44 (m, 3H), 4.35 (d, *J* = 11.9 Hz, 1H), 4.14 – 4.06 (m, 3H), 3.99 – 3.87 (m, 4H), 3.81 – 3.71 (m, 6H), 3.67 (d, *J* = 10.6 Hz, 2H), 3.63 – 3.55 (m, 1H), 3.55 – 3.44 (m, 1H), 3.25 – 3.11 (m, 3H), 1.52 – 1.38 (m, 4H), 1.21 – 1.13 (m, 2H). ^{13}C NMR (151 MHz, $CDCl_3$) δ 165.5, 138.7, 138.6, 138.6, 138.5, 138.2, 133.2, 130.2, 130.1, 128.7, 128.6, 128.5, 128.5, 128.5, 128.5, 128.4, 128.4, 128.4, 128.2, 128.1, 128.1, 127.9, 127.8, 127.8, 127.7, 127.7, 127.6, 127.6, 127.5, 127.5, 127.3, 100.8, 99.6, 98.9, 79.8, 78.2, 77.4, 77.2, 76.9, 75.6, 75.4, 75.3, 75.1, 75.0, 74.9, 74.4, 73.4, 73.4, 73.3, 72.3, 72.3, 72.2, 72.2, 71.9, 71.7, 69.7, 69.4, 69.1, 67.6, 67.3, 50.6, 50.3, 47.2, 46.2, 29.4, 28.1, 27.6, 23.5. HR-ESI-MS (m/z): calculated for $C_{108}H_{113}NO_{19}Na$ $[M+Na]^+$: 1750.7805, found: 1750.7801.

***N*-(Benzyl)benzyloxycarbonyl-5-aminopentyl 3,4,6-*O*-tri-benzyl-α-D-mannopyranosyl-(1→2)-3,4,6-*O*-tri-benzyl-α-D-mannopyranosyl-(1→2)-3,4,6-*O*-tri-benzyl-α-D-mannopyranoside (26)**

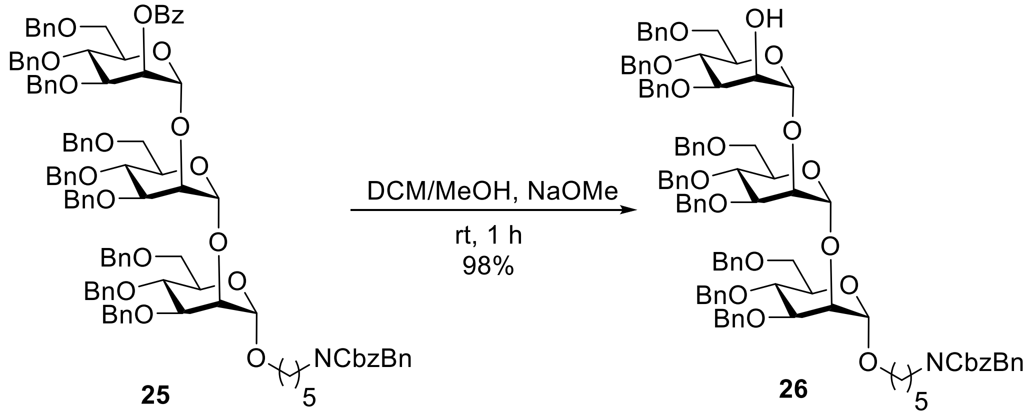

Compound **25** (1.569 g, 0.907 mmol) was dissolved in a mixture of DCM/MeOH (1:2, 15 mL), NaOMe (1.81 mL, 0.907 mmol) was added and the reaction mixture was stirred for 4 h at rt. After completion, reaction mixture was neutralized with the addition of Amberlite® IR120 H^+, filtered and concentrated. The crude product was purified by silica gel column chromatography to get trisaccharide **26** (1.441 g, 98%). ^1H NMR (600 MHz, $CDCl_3$) δ 7.37 – 7.10 (m, 55H), 5.21 (d, *J* = 2.0 Hz, 1H), 5.19 – 5.11 (m, 3H), 4.88 (s, 1H), 4.81 (t, *J* = 10.1 Hz, 3H), 4.66 (d, *J* = 12.3 Hz, 2H), 4.62 – 4.42 (m, 14H), 4.31 (d, *J* = 12.2 Hz, 1H), 4.11 (q, *J* = 2.5 Hz, 2H), 3.97 – 3.86 (m, 6H), 3.86 – 3.70 (m, 6H), 3.67 (d, *J* = 10.6 Hz, 2H), 3.62 (dd, *J* = 10.5, 3.2 Hz, 1H), 3.56 – 3.46 (m, 2H), 3.25 – 3.08 (m, 3H), 2.36 (s, 1H), 1.51 – 1.32 (m, 4H), 1.22 – 1.02 (m, 2H). ^{13}C NMR (151 MHz, $CDCl_3$) δ 138.7, 138.6, 138.5, 138.4, 138.3, 138.2, 128.7, 128.6, 128.6, 128.6, 128.5, 128.5, 128.5, 128.4, 128.4, 128.4, 128.2, 128.0, 128.0, 127.9, 127.8, 127.7, 127.7, 127.6, 127.6, 127.6, 127.5, 127.3, 101.1, 101.0, 98.9, 80.1, 79.6, 77.4, 77.2, 77.0, 75.4, 75.2, 75.1, 75.0, 75.0, 74.4, 73.4, 73.4, 73.4, 72.4, 72.3, 72.2, 71.9, 71.7, 69.7, 69.4, 69.0, 68.6, 67.6, 67.3, 50.6, 50.3, 47.2, 46.2, 29.4, 28.1, 27.6, 23.5. HR-ESI-MS (m/z): calculated for $C_{101}H_{109}NO_{18}Na$ $[M+Na]^+$: 1646.7542, found: 1646.7487.

_N_-(Benzyl)benzyloxycarbonyl-5-aminopentyl 2,3-_O_-di-benzyl-4,6-_O_-benzylidene-β-D-mannopyranosyl-(1→2)-3,4,6-_O_-tri-benzyl-α-D-mannopyranosyl-(1→2)-3,4,6-_O_-tri-benzyl-α-D-mannopyranosyl-(1→2)-3,4,6-_O_-tri-benzyl-α-D-mannopyranoside (27)

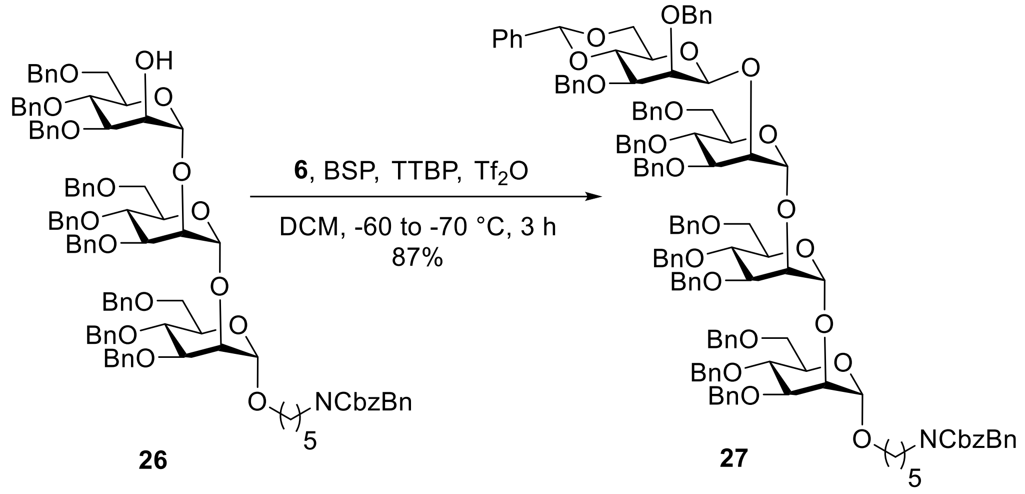

To a solution of donor **6** (181.8 mg, 0.369 mmol) in DCM (5 mL) was added 4 Å molecular sieves and the solution was stirred for 30 min. BSP (77.2 mg, 0.396 mmol) and TTBP (122.2 mg, 0.492 mmol) were added to the reaction mixture at -60 °C and stirred the reaction for 30 min. After stirring for 30 min, Tf_2O (103 µL, 0.615 mmol) was added and the mixture was stirred for 30 min. The reaction mixture was cooled to -70 °C and acceptor **26** was added. After stirring for 2 h, reaction was quenched by adding Et_3N (1 mL) and filtered through Celite®. The organic layer was washed with saturated aqueous sodium bicarbonate, dried over Na_2SO_4 and evaporated *in vacuo*. The crude was purified by silica gel column chromatography (20% ethyl acetate in hexanes) to get the tetrasaccharide **27** (220 mg, 87%). ^{1}H NMR (600 MHz, $CDCl_3$) δ 7.50 – 7.45 (m, 4H), 7.41 (d, *J* = 7.3 Hz, 2H), 7.37 (dd, *J* = 5.4, 1.9 Hz, 3H), 7.35 – 7.27 (m, 18H), 7.26 – 6.96 (m, 34H), 5.54 (s, 1H), 5.20 (s, 1H), 5.15 (d, *J* = 16.6 Hz, 2H), 5.05 (d, *J* = 2.6 Hz, 1H), 5.00 (d, *J* = 11.7 Hz, 1H), 4.88 – 4.81 (m, 3H), 4.77 (dd, *J* = 11.5, 4.2 Hz, 2H), 4.72 – 4.55 (m, 5H), 4.55 – 4.44 (m, 10H), 4.41 (d, *J* = 11.1 Hz, 1H), 4.23 – 4.12 (m, 5H), 4.08 (t, *J* = 9.6 Hz, 1H), 3.98 (s, 1H), 3.93 (dd, *J* = 7.9, 3.0 Hz, 1H), 3.91 – 3.81 (m, 5H), 3.81 – 3.77 (m, 3H), 3.77 – 3.64 (m, 6H), 3.56 – 3.44 (m, 2H), 3.41 (dd, *J* = 10.5, 2.0 Hz, 1H), 3.24 (dd, *J* = 9.9, 3.2 Hz, 1H), 3.22 – 3.09 (m, 3H), 2.89 (s, 1H), 1.52 – 1.36 (m, 4H), 1.35 – 1.23 (m, 2H). ^{13}C NMR (176 MHz, $CDCl_3$) δ 156.8, 156.2, 101.5, 100.7, 100.1, 98.8, 80.0, 79.9, 78.5, 77.8, 77.3, 77.2, 77.0, 76.4, 76.1, 75.3, 75.2, 75.2, 75.1, 75.0, 74.9, 74.4, 73.5, 73.4, 73.4, 73.3, 72.9, 72.9, 72.2, 72.0, 71.9, 71.7, 71.6, 71.3, 69.5, 69.3, 68.8, 68.6, 67.6, 67.2, 67.1, 50.5, 50.2, 47.1, 46.2, 29.8, 29.3, 28.0, 27.6, 23.4, 22.8. HR-ESI-MS (m/z): calculated for $C_{128}H_{135}NO_{23}Na$ $[M+Na]^+$: 2076.9323, found: 2076.9321.

5-Aminopentyl β-D-mannopyranosyl-(1→2)-α-D-mannopyranosyl-(1→2)-α-D-mannopyranosyl-(1→2)-α-D-mannopyranoside (28)

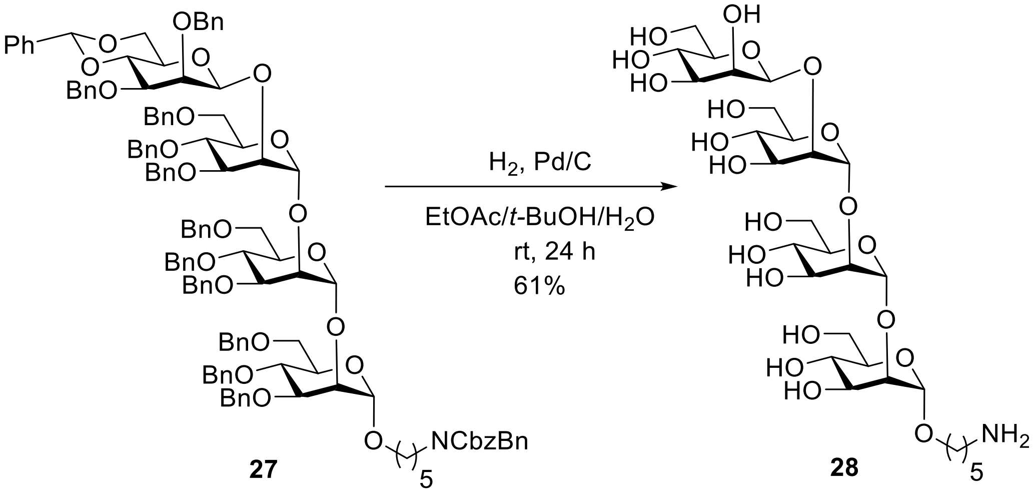

Tetrasaccharide **27** (25 mg, 12.2 µmol) was dissolved in a mixture of EtOAc/*t*-BuOH/H_2O (2/1/1, *v*/*v*/*v*, 2 mL) and Pd/C (60 mg) was added to the solution. After stirring for 24 h under a hydrogen atmosphere (1 atm, balloon), the mixture was filtered through a PTFE filter (0.45 µm pore size) and concentrated. The crude material was purified by HPLC (Hypercarb column, 150 x 10 mm, H_2O (0.1% formic acid) isocratic (5 min), linear gradient to 10% ACN (30 min), linear gradient to 100% ACN (10 min)) and lyophilized to obtain pure trisaccharide **28** (5.6 mg, 61%). ^{1}H NMR (700 MHz, D_2O) δ 5.24 (d, *J* = 1.8 Hz, 1H), 5.12 (d, *J* = 1.8 Hz, 1H), 5.04 (d, *J* = 1.7 Hz, 1H), 4.75 (s, 1H), 4.24 (dd, *J* = 3.4, 1.8 Hz, 1H), 4.08 (dd, *J* = 3.3, 1.8 Hz, 1H), 3.99 (d, *J* = 3.3 Hz, 1H), 3.92 – 3.87 (m, 3H), 3.86 – 3.80 (m, 5H), 3.73 – 3.66 (m, 7H), 3.66 – 3.58 (m, 4H), 3.56 (ddd, *J* = 9.6, 6.3, 2.3 Hz, 1H), 3.54 – 3.47 (m, 2H), 3.33 (ddd, *J* = 9.5, 6.7, 2.3 Hz, 1H), 2.95 (t, *J* = 7.6 Hz, 2H), 1.70 – 1.55 (m, 3H), 1.45 – 1.35 (m, 2H). ^{13}C NMR (176 MHz, D_2O) δ 100.6, 99.9, 98.4, 97.9, 78.9, 78.7, 76.9, 76.3, 73.3, 73.2, 72.7, 70.7, 70.1, 69.8, 69.6, 67.4, 67.0, 67.0, 66.9, 66.6, 61.1, 60.9, 60.8, 60.7, 39.3, 27.9, 26.4, 22.4. HR-ESI-MS (m/z): calculated for $C_{128}H_{135}NO_{23}Na$ $[M+Na]^+$: 2076.9323, found: 2076.9321.

5-Aminopentyl α-D-mannopyranosyl-(1→2)-α-D-mannopyranosyl-(1→2)-α-D-mannopyranoside (29)

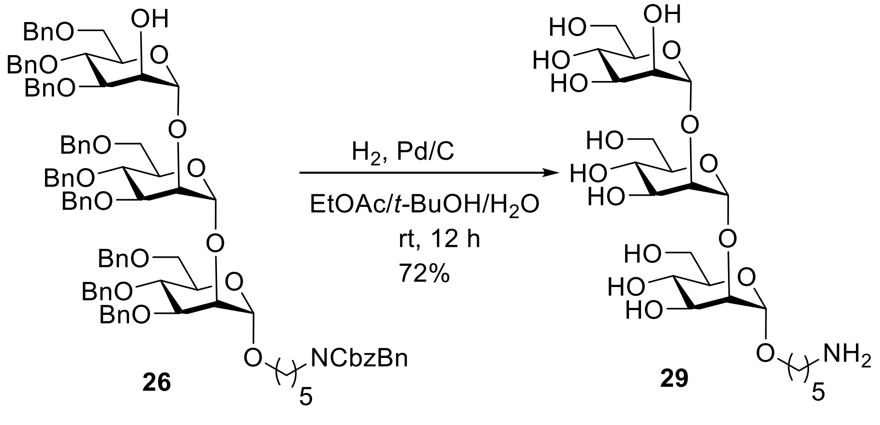

The trisaccharide **26** (20 mg, 12.3 µmol) was dissolved in the solution of EtOAc/*t*-BuOH/H_2O (2/1/1, *v*/*v*/*v*, 2 mL) and Pd/C (50 mg) was added to the solution. After stirring 12 h under a hydrogen atmosphere (1

atm, balloon), the mixture was filtered through a PTFE filter (0.45 µm pore size) and concentrated. The crude material was purified by HPLC (Hypercarb column, 150 x10 mm, H_2O (0.1% formic acid) isocratic (5 min), linear gradient to 10% ACN (30 min), linear gradient to 100% ACN (10 min)) and lyophilized to obtain pure trisaccharide **29** (5.2 mg, 72%). ^{1}H NMR (400 MHz, D_2O) δ 5.23 (s, 1H), 5.04 (d, *J* = 1.7 Hz, 1H), 4.98 (d, *J* = 1.9 Hz, 1H), 4.08 – 3.98 (m, 2H), 3.92 – 3.80 (m, 6H), 3.78 (dd, *J* = 9.7, 3.3 Hz, 1H), 3.75 – 3.64 (m, 6H), 3.62 (d, *J* = 2.8 Hz, 1H), 3.57 (dt, *J* = 14.1, 6.3 Hz, 3H), 3.51 – 3.44 (m, 1H), 2.94 (t, *J* = 7.6 Hz, 2H), 1.69 – 1.51 (m, 4H), 1.49 – 1.32 (m, 2H). ^{13}C NMR (101 MHz, D_2O) δ 102.2, 100.6, 97.9, 79.0, 78.6, 73.2, 72.7, 70.2, 70.1, 69.8, 67.4, 67.0, 66.9, 66.7, 61.1, 60.8, 39.2, 27.9, 26.4, 22.3. HR-ESI-MS (m/z): calculated for $C_{23}H_{43}NO_{16}H$ $[M+H]^+$: 590.2660, found: 590.2669.

***N*-(Benzyl)benzyloxycarbonyl-5-aminopentyl 2,3-*O*-di-benzyl-4,6-*O*-benzylidene-β-D-mannopyranoside (30)**

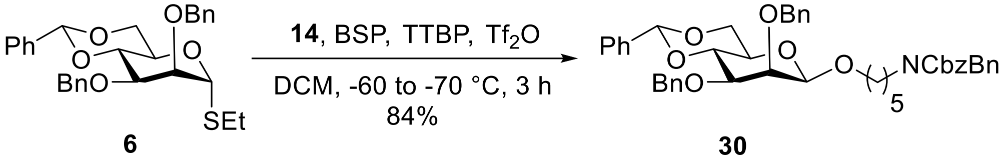

6 **30**

To a solution of donor **6** (315 mg, 0.641 mmol) in DCM (8 mL) was added 4 Å molecular sieves and the solution was stirred for 30 min. BSP (128 mg, 0.614 mmol) and TTBP (212 mg, 0.586 mmol) were added to the reaction mixture at -60 °C and stirred the reaction for 30 min. After stirring for 30 min, Tf_2O (179 µL, 1.07 mmol) was added and stirred the mixture for another 30 min. The reaction mixture was brought to -70 °C and acceptor **14** (70 mg, 0.214 mmol) was added. After stirring for 2 h, reaction was quenched by adding Et_3N (0.5 mL) and filtered through Celite®. The organic layer was washed with saturated aqueous sodium bicarbonate, dried over Na_2SO_4 and evaporated *in vacuo*. The crude product was purified by silica gel column chromatography (10% ethyl acetate in hexanes) to obtain linker attached monosaccharide **30** (136 mg, 84%). ^{1}H NMR (400 MHz, $CDCl_3$) δ 7.54 – 7.49 (m, 2H), 7.46 (d, *J* = 7.0 Hz, 2H), 7.42 – 7.35 (m, 5H), 7.34 – 7.26 (m, 15H), 7.18 (d, *J* = 7.2 Hz, 1H), 5.63 (s, 1H), 5.18 (d, *J* = 14.6 Hz, 2H), 5.01 – 4.93 (m, 1H), 4.86 (d, *J* = 12.3 Hz, 1H), 4.75 – 4.63 (m, 1H), 4.59 (d, *J* = 12.5 Hz, 1H), 4.51 (d, *J* = 9.0 Hz, 2H), 4.41 (d, *J* = 19.0 Hz, 1H), 4.31 (dd, *J* = 10.4, 4.8 Hz, 1H), 4.22 (t, *J* = 9.6 Hz, 1H), 3.99 – 3.81 (m, 3H), 3.58 (dd, *J* = 9.9, 3.2 Hz, 1H), 3.45 – 3.15 (m, 3H), 1.68 – 1.52 (m, 4H), 1.41 – 1.32 (m, 2H). ^{13}C NMR (101 MHz, $CDCl_3$) δ 156.8, 156.3, 138.5, 138.4, 138.0, 137.8, 137.7, 137.0, 136.8, 129.0, 128.8, 128.8, 128.7, 128.6, 128.6, 128.6, 128.5, 128.4, 128.4, 128.3, 128.3, 128.2, 128.1, 127.9, 127.8, 127.7, 127.4, 127.3, 126.2, 126.2, 102.4, 101.5, 94.1, 79.4, 79.1, 78.7, 78.0, 77.5, 77.4, 77.2, 76.8, 76.0, 75.7, 74.7, 73.7, 73.3, 72.4, 70.1, 69.0, 68.7, 67.7, 67.3, 50.6, 50.3, 47.2, 46.3, 32.1, 31.6, 30.3, 29.8, 29.5, 29.4, 28.0, 27.6, 23.4, 22.8, 14.3. HR-ESI-MS (m/z): calculated for $C_{47}H_{51}NO_8Na$ $[M+Na]^+$: 780.3512, found: 780.3519.

***N*-(Benzyl)benzyloxycarbonyl-5-aminopentyl 2,3,4-*O*-tri-benzyl-β-D-mannopyranoside (31)**

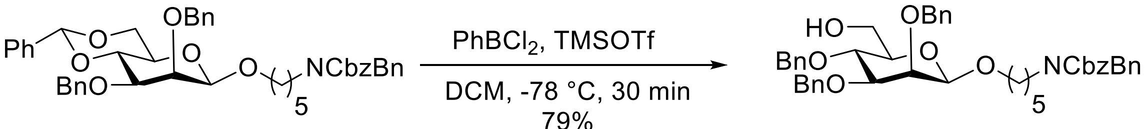

30 **31**

To a solution of **30** (65 mg, 0.085 mmol) in the anhydrous DCM (5 mL) was added 4 Å molecular sieves and stirred the mixture under argon for 30 min at rt. The mixture was cooled to the -78 °C, added

triethylsilane (40 µL, 0.255 mmol) and dichlorophenylborane (22 µL, 0.171 mmol). After stirring for 30 min, reaction was quenched by adding Et_3N (0.5 mL) and filtered through Celite®. The organic layer was washed with saturated aqueous sodium bicarbonate, dried over Na_2SO_4 and evaporated *in vacuo*. The crude was purified by silica gel column chromatography (10% ethyl acetate in hexanes) to furnish the title compound **31** (51 mg, 79%). ^1H NMR (600 MHz, $CDCl_3$) δ 7.50 – 7.42 (m, 2H), 7.40 – 7.25 (m, 22H), 7.19 (d, *J* = 7.5 Hz, 1H), 5.20 (d, *J* = 22.5 Hz, 2H), 4.97 (t, *J* = 9.8 Hz, 2H), 4.86 (d, *J* = 12.5 Hz, 1H), 4.67 (d, *J* = 10.9 Hz, 1H), 4.59 – 4.48 (m, 4H), 4.39 (d, *J* = 25.8 Hz, 1H), 3.98 – 3.87 (m, 4H), 3.80 (dd, *J* = 11.8, 5.7 Hz, 1H), 3.57 – 3.52 (m, 1H), 3.47 – 3.18 (m, 4H), 1.70 – 1.51 (m, 4H), 1.43 – 1.26 (m, 2H). ^{13}C NMR (151 MHz, $CDCl_3$) δ 156.3, 138.6, 138.3, 138.2, 138.0, 137.0, 136.8, 134.3, 134.1, 128.6, 128.6, 128.6, 128.5, 128.5, 128.4, 128.3, 128.2, 128.2, 128.1, 128.0, 127.9, 127.8, 127.7, 127.7, 127.6, 127.6, 127.4, 127.3, 101.8, 82.4, 82.0, 77.4, 77.2, 76.9, 75.9, 75.5, 75.4, 75.0, 74.0, 73.9, 71.6, 69.9, 67.3, 62.7, 50.6, 50.3, 47.2, 46.3, 29.8, 29.4, 28.0, 27.4, 23.4. HR-ESI-MS (m/z): calculated for $C_{47}H_{53}NO_8Na$ $[M+Na]^+$: 782.3669, found: 782.3661.

5-Aminopentyl β-D-mannopyranoside (32)

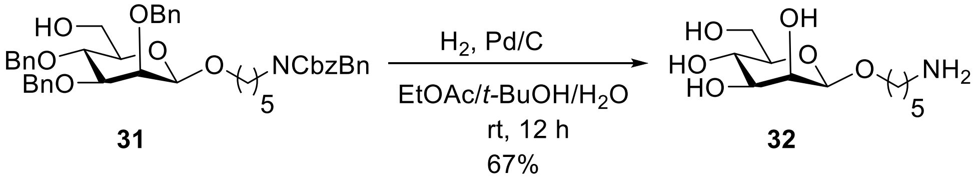

Monosaccharide **31** (24 mg, 31.58 µmol) was dissolved in a micture of EtOAc/*t*-BuOH/H_2O (2/1/1, *v/v/v,* 2 mL) and Pd/C (40 mg) was added to the solution. After stirring for 12 h under a hydrogen atmosphere (1 atm, balloon), the mixture was filtered through a PTFE filter (0.45 µm pore size) and concentrated. The crude material was purified by HPLC (Hypercarb column, 150 x10 mm, H_2O (0.1% formic acid) isocratic (5 min), linear gradient to 10% ACN (30 min), linear gradient to 100% ACN (10 min)) and lyophilized to obtain pure trisaccharide **32** (5.6 mg, 67%). ^1H NMR (600 MHz, D_2O) δ 4.65 (d, *J* = 1.0 Hz, 1H), 3.97 (dd, *J* = 3.2, 0.9 Hz, 1H), 3.94 – 3.87 (m, 2H), 3.72 (dd, *J* = 12.2, 6.5 Hz, 1H), 3.69 – 3.65 (m, 1H), 3.62 (dd, *J* = 9.6, 3.3 Hz, 1H), 3.55 (t, *J* = 9.7 Hz, 1H), 3.35 (ddd, *J* = 9.7, 6.5, 2.3 Hz, 1H), 3.02 – 2.97 (m, 2H), 1.77 – 1.60 (m, 4H), 1.44 (h, *J* = 7.1 Hz, 2H). ^{13}C NMR (151 MHz, D_2O) δ 99.8, 76.2, 73.0, 70.5, 69.4, 66.8, 61.0, 39.3, 28.1, 26.4, 22.1. HR-ESI-MS (m/z): calculated for $C_{11}H_{24}NO_6$ $[M+H]^+$: 266.1604, found: 266.1594.

5-Aminopentyl-[α-D-mannopyranosyl]-6-phosphate-(1→2)-β-D-mannopyranoside (1)

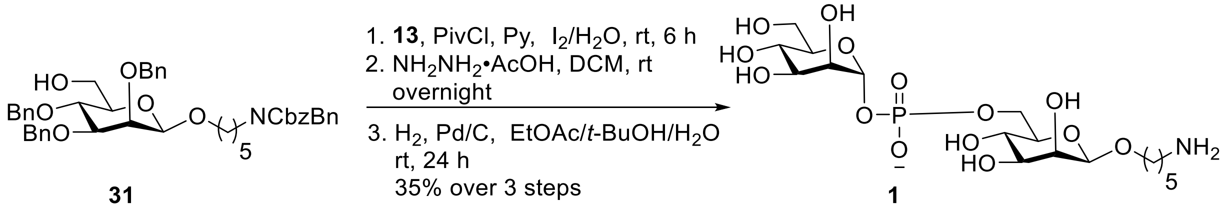

Monoaccharide alcohol **31** (20 mg, 26.3 µmol) and *H*-phosphonate **13** (37 mg, 52.6 µmol) were co-evaporated three times with pyridine and the resulting mixture was dried under vacuum overnight before dissolving it in anhydrous pyridine (3 mL). Pivaloyl chloride (16 µL, 131.5 µmol) was then added. After 5 h of stirring at rt, a freshly prepared solution of iodine (33 mg, 131.5 µmol) in pyridine/water (10/1, 0.3 mL) was added. After 3 h, the reaction mixture was diluted with DCM, washed with saturated $Na_2S_2O_3$ solution and TEAB buffer, dried over Na_2SO_4 and evaporated under reduced

pressure. The crude was dissolved in DCM (3 mL), added hydrazine acetate (12 mg, 131.5 µmol) and stirred the reaction mixture overnight at rt. After completion of reaction, quenched by acetone and evaporated *in vacuo*. The crude was dissolved in the solution of EtOAc/*t*-BuOH/H_2O (2/1/1, *v*/*v*/*v,* 2 mL) and Pd/C (60 mg) was added to the solution. After stirring for 24 h under a hydrogen atmosphere (1 atm, balloon), the mixture was filtered through a PTFE filter (0.45 µm pore size) and concentrated. The crude material was purified by HPLC (Hypercarb column, 150 x10 mm, H_2O (0.1% formic acid) isocratic (5 min), linear gradient to 40% ACN (35 min), linear gradient to 100% ACN (10 min)) and lyophilized to obtain pure trisaccharide **2** (7 mg, 35%). ^{1}H NMR (400 MHz, D_2O) δ 5.30 (dd, *J* = 7.8, 2.0 Hz, 1H), 4.55 (d, *J* = 1.0 Hz, 1H), 4.12 – 4.03 (m, 1H), 3.95 – 3.89 (m, 1H), 3.87 (dd, *J* = 3.4, 2.0 Hz, 1H), 3.86 – 3.83 (m, 1H), 3.80 – 3.73 (m, 3H), 3.73 – 3.61 (m, 2H), 3.60 – 3.53 (m, 2H), 3.51 – 3.48 (m, 2H), 3.40 – 3.33 (m, 1H), 2.87 (t, *J* = 7.5 Hz, 2H), 1.64 – 1.45 (m, 4H), 1.40 – 1.24 (m, 2H). ^{13}C NMR (151 MHz, D_2O) δ 99.9, 96.1, 74.9, 74.8, 73.8, 72.9, 70.5, 70.4, 70.4, 69.8, 69.5, 66.5, 66.3, 65.0, 64.9, 60.8, 39.4, 28.0, 26.3, 22.0. ^{31}P NMR (162 MHz, D_2O) δ -2.0. HR-ESI-MS (m/z): calculated for $C_{17}H_{33}NO_{14}P$ $[M]^-$: 506.1644, found: 506.1650

N-(Benzyl)benzyloxycarbonyl-5-aminopentyl 2,3,4-O-tri-benzyl-β-D-mannopyranosyl-(1→2)-3,4,6-O-tri-benzyl-α-D-mannopyranoside (33)

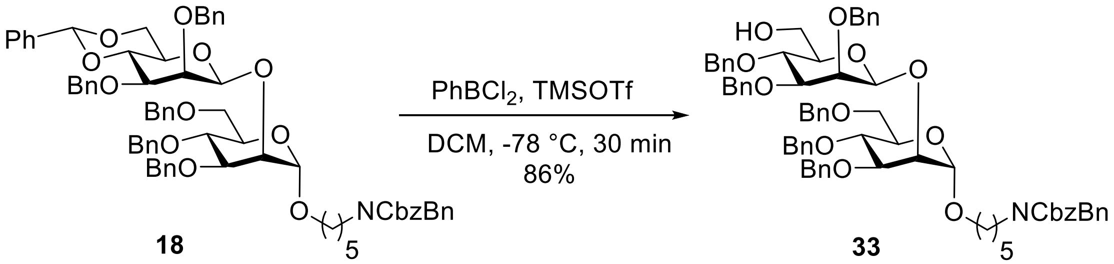

18 **33**

To a solution of disaccharide **18** (200 mg, 0.168 mmol) in anhydrous DCM (6 mL) was added 4 Å molecular sieves and the mixture was stirred under argon for 30 min at rt. The mixture was cooled to -78 °C, added triethylsilane (80 µL, 0.504 mmol) and dichlorophenylborane (43 µL, 0.336 mmol). After stirring for 30 min, reaction was quenched by adding Et_3N (1 mL) and filtered through Celite®. The organic layer was washed with saturated aqueous sodium bicarbonate, dried over Na_2SO_4 and evaporated *in vacuo*. The crude product was purified by silica gel column chromatography (15% ethyl acetate in hexanes) to get the disaccharide **33** (173 mg, 86%).^{1}H NMR (600 MHz, $CDCl_3$) δ 7.54 – 7.48 (m, 2H), 7.47 – 7.42 (m, 2H), 7.29 (tdt, *J* = 8.1, 5.6, 3.0 Hz, 17H), 7.26 – 7.19 (m, 16H), 7.15 (d, *J* = 7.4 Hz, 1H), 7.07 (dt, *J* = 4.5, 3.2 Hz, 2H), 5.16 (d, *J* = 22.4 Hz, 2H), 5.08 (d, *J* = 11.7 Hz, 1H), 4.94 (d, *J* = 10.8 Hz, 1H), 4.85 (dd, *J* = 11.7, 8.5 Hz, 3H), 4.78 (d, *J* = 10.7 Hz, 1H), 4.63 – 4.52 (m, 5H), 4.51 – 4.41 (m, 4H), 4.32 (d, *J* = 10.8 Hz, 1H), 4.24 – 4.16 (m, 1H), 4.02 – 3.95 (m, 2H), 3.91 (d, *J* = 8.5 Hz, 2H), 3.80 – 3.67 (m, 4H), 3.68 – 3.58 (m, 2H), 3.50 (d, *J* = 9.1 Hz, 1H), 3.37 – 3.13 (m, 4H), 1.53 – 1.39 (m, 4H), 1.23 – 1.13 (m, 2H). ^{13}C NMR (176 MHz, $CDCl_3$) δ 156.9, 156.3, 138.8, 138.6, 138.5, 138.4, 138.3, 138.2, 138.0, 137.0, 136.8, 129.2, 128.8, 128.7, 128.6, 128.6, 128.6, 128.5, 128.5, 128.4, 128.4, 128.3, 128.3, 128.3, 128.2, 128.2, 128.1, 128.0, 127.9, 127.9, 127.9, 127.8, 127.7, 127.7, 127.7, 127.6, 127.5, 127.4, 127.3, 99.8, 97.5, 81.4, 78.5, 77.3, 77.2, 77.0, 76.0, 75.4, 75.3, 75.3, 74.6, 74.4, 74.3, 74.3, 74.1, 73.5, 73.0, 71.5, 71.1, 70.9, 70.8, 70.0, 69.0, 67.7, 67.3, 62.3, 50.6, 50.3, 47.2, 46.2, 45.4, 32.1, 30.3, 29.9, 29.5, 29.3, 28.1, 27.6, 23.6, 22.8, 21.6, 14.3, 1.2. HR-ESI-MS (m/z): calculated for $C_{74}H_{81}NO_{13}Na$ $[M+Na]^+$: 1214.5606, found: 1214.55991.

5-Aminopentyl-β-D-mannopyranosyl [α-D-mannopyranosyl]-6-phosphate-(1→2)-α-D-mannopyranoside (2)

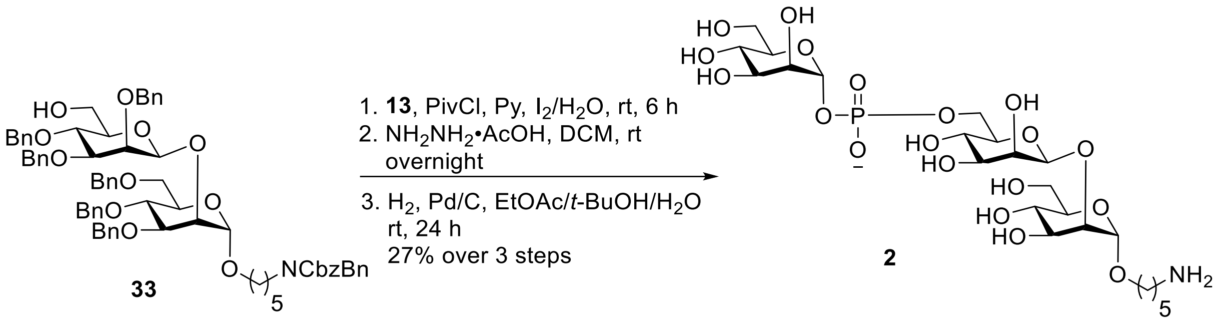

33 **2**

Disaccharide alcohol **33** (30 mg, 25.15 µmol) and *H*-phosphonate **13** (35.9 mg, 50.3 µmol) were co-evaporated three times with pyridine and the resulting mixture was dried under vacuum overnight before dissolving it in anhydrous pyridine (3 mL). Pivaloyl chloride (15.3 µL, 125.7 µmol) was then added. After 5 h of stirring at rt, a freshly prepared solution of iodine (31.9 mg, 125.7 µmol) in pyridine/water (10/1, 0.3 mL) was added. After 3 h, the reaction was diluted with DCM, washed with saturated $Na_2S_2O_3$ solution and TEAB buffer, dried over Na_2SO_4 and evaporated under reduced pressure. The crude product was dissolved in DCM (3 mL), hydrazine acetate (11.5 mg, 125.7 µmol) was added and stirred the reaction mixture overnight at rt. After completion of the reaction, it was quenched by adding acetone and evaporated *in vacuo*. The crude product was dissolved in the solution of EtOAc/*t*-BuOH/H_2O (2/1/1, *v*/*v*/*v*, 2 mL) and Pd/C (60 mg) was added to the solution. After stirring for 24 h under a hydrogen atmosphere (1 atm, balloon), the mixture was filtered through a PTFE filter (0.45 µm pore size) and concentrated. The crude material was purified by HPLC (Hypercarb column, 150 x10 mm, H_2O (0.1% formic acid) isocratic (5 min), linear gradient to 40% ACN (35 min), linear gradient to 100% ACN (10 min)) and lyophilized to obtain pure trisaccharide **2** (4.6 mg, 27%). ^1H NMR (600 MHz, D_2O) δ 5.33 (dd, *J* = 7.9, 2.0 Hz, 1H), 4.88 (s, 1H), 4.12 (ddd, *J* = 11.4, 5.6, 2.0 Hz, 1H), 4.05 (dd, *J* = 3.3, 1.8 Hz, 1H), 3.98 – 3.92 (m, 2H), 3.91 (dd, *J* = 3.4, 2.0 Hz, 1H), 3.84 – 3.75 (m, 3H), 3.75 – 3.63 (m, 5H), 3.62 – 3.51 (m, 5H), 3.49 – 3.38 (m, 2H), 2.92 – 2.87 (m, 2H), 1.62 – 1.49 (m, 4H), 1.40 – 1.31 (m, 2H). ^{13}C NMR (151 MHz, D_2O) δ 98.7, 97.5, 96.1, 96.1, 77.6, 75.1, 75.0, 73.8, 72.8, 72.6, 70.7, 70.4, 70.0, 69.8, 67.6, 67.1, 66.4, 66.3, 65.0, 64.9, 60.8, 60.6, 39.4, 28.0, 26.5, 22.5. ^{31}P NMR (162 MHz, D_2O) δ -2.0. HR-ESI-MS (m/z): calculated for $C_{23}H_{43}NO_{19}P$ $[M]^-$: 668.2172, found: 668.2153.

***N*-(Benzyl)benzyloxycarbonyl-5-aminopentyl 2,3,4-*O*-tri-benzyl-β-D-mannopyranosyl-(1→2)-3,4,6-*O*-tri-benzyl-α-D-mannopyranosyl-(1→2)-3,4,6-*O*-tri-benzyl-α-D-mannopyranoside (34)**

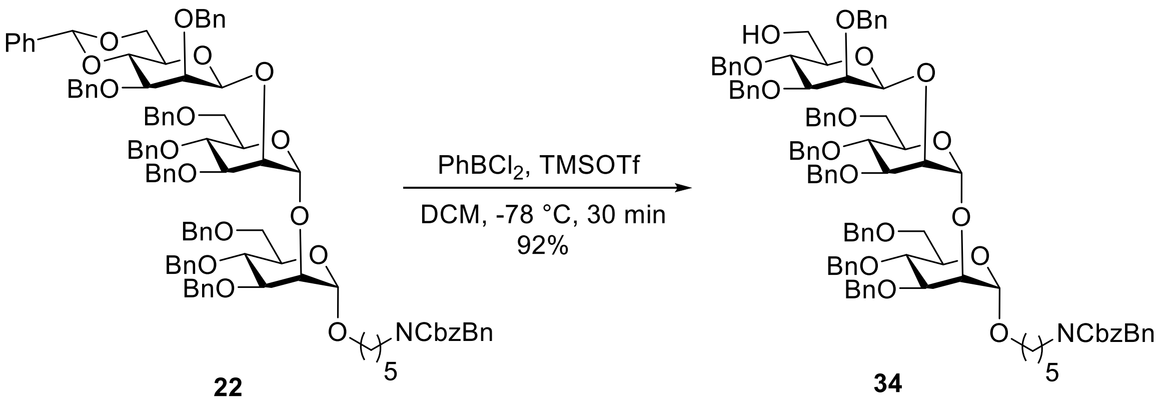

22 **34**

To a solution of **22** (140 mg, 0.086 mmol) in anhydrous DCM (7 mL) 4 Å molecular sieves were added and stirred the mixture under argon for 30 min at rt. The mixture was cooled to -78 °C, triethylsilane (41 µL, 0.258 mmol) and dichlorophenylborane (22 µL, 0.172 mmol) were added. After stirring for 30 min, reaction was quenched by adding Et_3N (1 mL) and filtered through Celite®. The organic layer was washed with saturated aqueous sodium bicarbonate, dried over Na_2SO_4 and evaporated *in vacuo*. The crude was purified by silica gel column chromatography (17% ethyl acetate in hexanes) to get the trisaccharide **34** (128 mg, 92%). ^{1}H NMR (400 MHz, $CDCl_3$) δ 7.50 (dd, *J* = 6.6, 2.9 Hz, 2H), 7.45 – 7.41 (m, 2H), 7.38 – 7.26 (m, 35H), 7.25 – 7.18 (m, 11H), 7.14 (d, *J* = 7.6 Hz, 2H), 7.13 – 7.09 (m, 3H), 5.20 (d, *J* = 12.1 Hz, 2H), 5.12 (d, *J* = 2.3 Hz, 1H), 5.00 (dd, *J* = 28.0, 11.5 Hz, 2H), 4.91 – 4.85 (m, 2H), 4.81 (dd, *J* = 11.7, 5.2 Hz, 2H), 4.78 – 4.71 (m, 2H), 4.69 (d, *J* = 4.8 Hz, 1H), 4.67 – 4.57 (m, 5H), 4.57 – 4.42 (m, 6H), 4.39 – 4.27 (m, 3H), 4.24 (t, *J* = 2.7 Hz, 1H), 4.08 (t, *J* = 2.4 Hz, 1H), 3.98 (dd, *J* = 8.2, 3.0 Hz, 1H), 3.92 (t, *J* = 9.4 Hz, 3H), 3.86 – 3.77 (m, 4H), 3.76 – 3.64 (m, 6H), 3.56 (d, *J* = 8.2 Hz, 1H), 3.31 – 3.16 (m, 4H), 3.09 – 2.99 (m, 1H), 1.55 – 1.44 (m, 4H), 0.94 – 0.82 (m, 2H). ^{13}C NMR (101 MHz, $CDCl_3$) δ 156.8, 156.3, 138.9, 138.8, 138.7, 138.5, 138.4, 138.4, 138.1, 138.0, 137.0, 136.9, 128.7, 128.7, 128.6, 128.6, 128.5, 128.5, 128.5, 128.5, 128.4, 128.4, 128.4, 128.3, 128.2, 128.1, 128.1, 128.0, 128.0, 127.9, 127.9, 127.7, 127.7, 127.7, 127.7, 127.6, 127.6, 127.5, 127.5, 127.4, 127.3, 99.8, 99.7, 99.0, 81.3, 80.4, 78.1, 77.5, 77.4, 77.2, 76.8, 75.8, 75.4, 75.2, 75.1, 74.8, 74.5, 74.4, 74.3, 73.6, 73.4, 73.4, 72.8, 71.9, 71.8, 71.2, 70.9, 69.3, 67.6, 67.3, 62.3, 50.6, 50.3, 47.2, 46.2, 29.8, 29.3, 28.1, 27.6, 23.5, 14.3. HR-ESI-MS (m/z): calculated for $C_{101}H_{110}NO_{18}H$ $[M+H]^+$: 1624.7723, found: 1624.7772

5-Aminopentyl-β-D-mannopyranosyl [α-D-mannopyranosyl]-6-phosphate-(1→2)-α-D-mannopyranosyl-(1→2)-α-D-mannopyranoside (3)

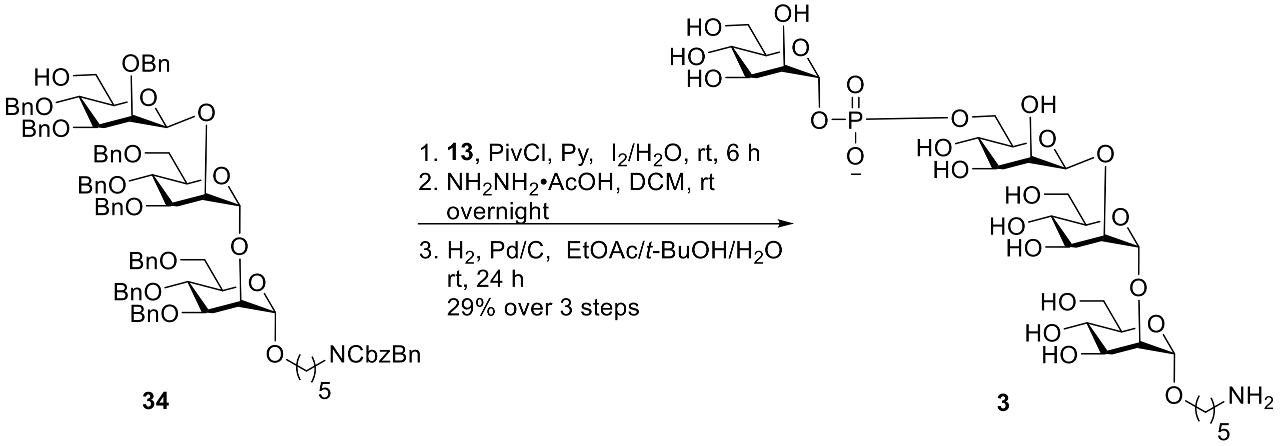

Trisaccharide alcohol **34** (28 mg, 17.2 µmol) and *H*-phosphonate **13** (31 mg, 43.7 µmol) were coevaporated three times with pyridine and the resulting mixture was dried under vacuum overnight before dissolving in anhydrous pyridine (3 mL). Pivaloyl chloride (10.5 µL, 85.2 µmol) was then added. After 5 h of stirring at rt, a freshly prepared solution of iodine (21.8 mg, 86.2 µmol) in pyridine/water (10/1, 0.3 mL) was added. After 3 h, the reaction mixture was diluted with DCM, washed with saturated $Na_2S_2O_3$ solution and TEAB buffer, dried over Na_2SO_4 and evaporated under reduced pressure. The crude product was dissolved in DCM (3 mL), added hydrazine acetate (7.9 mg, 86.2 µmol) and the reaction mixture was stirred overnight at rt. After completion of the reaction, it was quenched by adding acetone and evaporated *in vacuo*. The crude product was dissolved in a mixture of EtOAc/*t*-BuOH/H_2O (2/1/1, *v/v/v,* 2 mL) and Pd/C (60 mg) was added to the solution. After stirring for 24 h under

a hydrogen atmosphere (1 atm, balloon), the mixture was filtered through a PTFE filter (0.45 µm pore size) and concentrated. The crude material was purified by HPLC (Hypercarb column, 150 x 10 mm, H_2O (0.1% formic acid) isocratic (5 min), linear gradient to 40% ACN (35 min), linear gradient to 100% ACN (10 min)) and lyophilized to obtain pure tetrasaccharide **3** (4.1 mg, 29%). ^{1}H NMR (700 MHz, D_2O) δ 5.39 (dd, *J* = 7.9, 2.0 Hz, 1H), 5.10 (d, *J* = 1.8 Hz, 1H), 5.05 (d, *J* = 1.8 Hz, 1H), 4.81 (d, *J* = 0.9 Hz, 1H), 4.26 (dd, *J* = 3.3, 1.8 Hz, 1H), 4.18 (ddd, *J* = 11.6, 5.8, 2.1 Hz, 1H), 4.06 – 3.98 (m, 2H), 3.97 (dd, *J* = 3.4, 2.0 Hz, 1H), 3.92 (dd, *J* = 3.4, 1.8 Hz, 1H), 3.90 – 3.86 (m, 1H), 3.86 – 3.77 (m, 6H), 3.75 – 3.67 (m, 5H), 3.66 – 3.60 (m, 5H), 3.57 (ddd, *J* = 9.7, 8.0, 4.5 Hz, 1H), 3.54 – 3.46 (m, 2H), 2.96 (t, *J* = 7.6 Hz, 2H), 1.66 – 1.55 (m, 4H), 1.46 – 1.35 (m, 2H). ^{13}C NMR (176 MHz, D_2O) δ 100.1, 98.7, 97.9, 96.1, 96.1, 78.8, 77.4, 75.0, 74.9, 73.7, 73.4, 72.7, 72.5, 70.6, 70.4, 70.3, 70.2, 69.7, 69.6, 67.4, 67.1, 66.9, 66.3, 66.2, 64.8, 60.9, 60.7, 39.3, 27.9, 26.4, 22.4. ^{31}P NMR (243 MHz, D_2O) δ -1.9. HR-ESI-MS (m/z): calculated for $C_{29}H_{53}NO_{24}P$ $[M]^-$: 830.2701, found: 830.2687

***N*-(Benzyl)benzyloxycarbonyl-5-aminopentyl 2,3,4-*O*-di-benzyl-β-D-mannopyranosyl-(1→2)-3,4,6-*O*-tri-benzyl-α-D-mannopyranosyl-(1→2)-3,4,6-*O*-tri-benzyl-α-D-mannopyranosyl-(1→2)-3,4,6-*O*-tri-benzyl-α-D-mannopyranoside (35)**

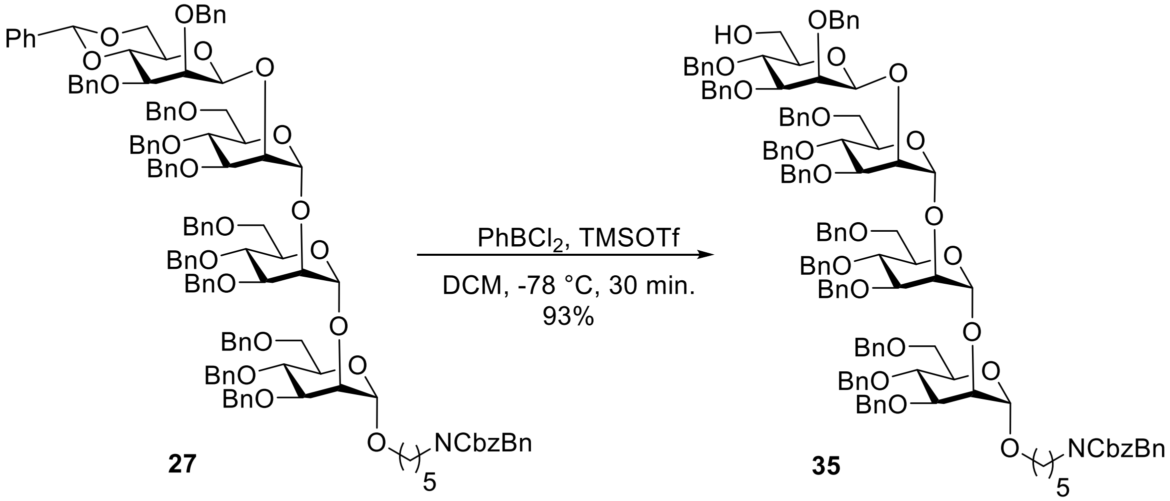

To a solution of tetrasaccharide **27** (183 mg, 0.089 mol) in DCM (5 mL) 4 Å molecular sieves were added and the reaction mixture was stirred for 30 min. After 30 min of stirring, the reaction mixture was cooled to -78 °C and triethylsilane (0.042 mL, 0.267 mmol) and dichlorophenyl borane (0.023 mL, 0.178 mmol) were added and the reaction mixture was stirred for another 30 min. The reaction was quenched by adding 1 mL of triethylamine, filtered through Celite®, washed the organic layer with saturated aqueous $NaHCO_3$, and brine, dried over Na_2SO_4 and evaporated *in vacuo*. The crude product was purified by silica gel column chromatography to obtain tetrasaccharide **34** (170 mg, 93%). ^{1}H NMR (700 MHz, $CDCl_3$) δ 7.48 (dd, *J* = 6.8, 2.9 Hz, 2H), 7.41 (d, *J* = 7.5 Hz, 2H), 7.35 – 7.26 (m, 31H), 7.25 – 7.18 (m, 20H), 7.17 – 7.12 (m, 9H), 7.11 – 7.03 (m, 6H), 5.22 – 5.12 (m, 3H), 5.07 (d, *J* = 2.3 Hz, 1H), 4.98 (dd, *J* = 54.3, 11.4 Hz, 2H), 4.90 – 4.82 (m, 3H), 4.79 (dd, *J* = 11.7, 3.4 Hz, 2H), 4.75 – 4.64 (m, 3H), 4.64 – 4.56 (m, 2H), 4.56 – 4.40 (m, 12H), 4.32 (d, *J* = 12.1 Hz, 1H), 4.26 – 4.17 (m, 5H), 4.01 – 3.94 (m, 2H), 3.93 – 3.74 (m, 11H), 3.73 – 3.65 (m, 5H), 3.47 (ddd, *J* = 44.6, 10.5, 2.8 Hz, 3H), 3.25 – 3.11 (m, 4H), 1.24 – 1.10 (m, 4H), 0.90 – 0.85 (m, 2H). ^{13}C NMR (176 MHz, $CDCl_3$) δ 138.9, 138.8, 138.6, 138.5, 138.5, 138.4, 138.4, 138.0, 137.0, 136.9, 128.7, 128.6, 128.6, 128.6, 128.5, 128.5, 128.5, 128.5, 128.4, 128.4, 128.3, 128.2, 128.2, 128.1, 128.0, 128.0, 127.9, 127.9, 127.9, 127.8, 127.8, 127.7, 127.7, 127.7, 127.6, 127.6, 127.6, 127.5, 127.5, 100.7, 99.7, 98.9, 81.2,

80.0, 79.9, 78.1, 77.3, 77.2, 77.0, 75.7, 75.4, 75.3, 75.2, 75.0, 74.9, 74.8, 74.6, 74.5, 74.5, 74.4, 74.2, 73.5, 73.4, 73.4, 73.4, 73.2, 72.8, 72.3, 72.0, 71.9, 71.9, 71.0, 70.8, 69.6, 69.3, 68.8, 67.6, 67.3, 67.3, 62.3, 50.6, 50.2, 47.2, 46.2, 32.1, 31.6, 31.6, 30.4, 30.3, 30.3, 30.2, 29.8, 29.8, 29.5, 29.4, 28.1, 27.6, 23.5, 22.8, 19.9, 14.3, 7.0, 6.7, 6.5, 5.9, 1.2. HR-ESI-MS (m/z): calculated for $C_{128}H_{138}NO_{23}H$ $[M+H]^+$: 2056.9660, found: 2056.9558

5-Aminopentyl-β-D-mannopyranosyl [α-D-mannopyranosyl]-6-phosphate-(1→2)-α-D-mannopyranosyl-(1→2)-α-D-mannopyranosyl-(1→2)-α-D-mannopyranoside (4)

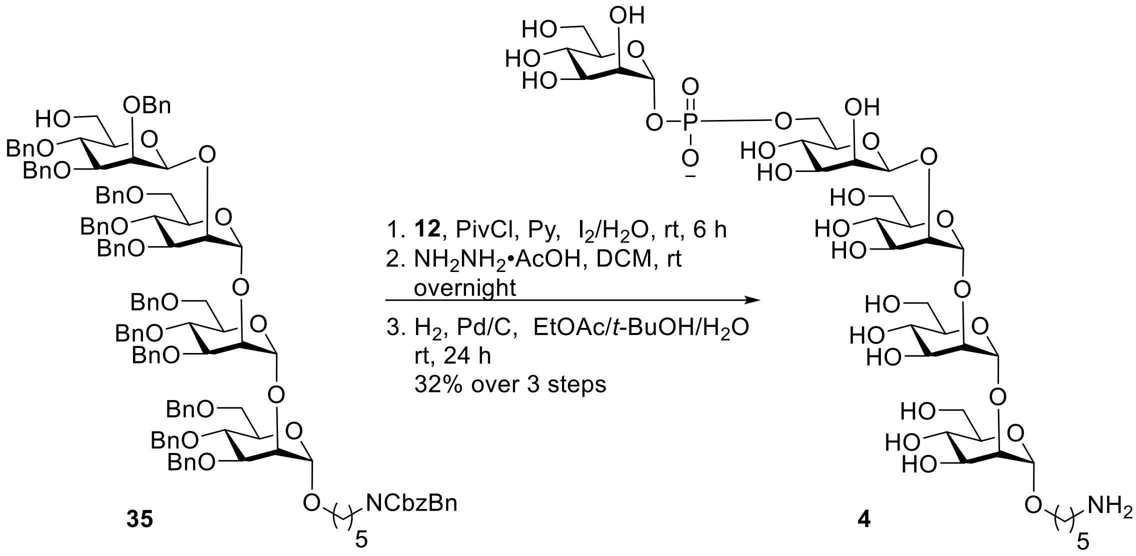

Tetrasaccharide alcohol **35** (25 mg, 12.15 µmol) and *H*-phosphonate **13** (17.3 mg, 24.3 µmol) were co-evaporated three times with pyridine and the resulting mixture was dried under vacuum overnight before dissolving it in anhydrous pyridine (2 mL). Pivaloyl chloride (7.4 µL, 60.7 µmol) was then added. After 5 h of stirring at rt, a freshly prepared solution of iodine (6.7 mg, 72.5 µmol) in pyridine/water (10/1, 0.3 mL) was added. After 2 h, the reaction was diluted with DCM, washed with saturated $Na_2S_2O_3$ solution and TEAB buffer, dried over Na_2SO_4 and evaporated under reduced pressure. The crude was dissolved in DCM (3 mL), hydrazine acetate (7.4 mg, 60.7 µmol) was added and the reaction mixture was stirred overnight at rt. After completion of reaction, quenched by acetone and evaporated *in vacuo*. The crude product was dissolved in the solution of EtOAc/*t*-BuOH/H_2O (2/1/1, *v*/*v*/*v,* 2 mL) and Pd/C (50 mg) was added to the solution. After stirring for 24 h under a hydrogen atmosphere (1 atm, balloon), the mixture was filtered through a PTFE filter (0.45 µm pore size) and concentrated. The crude material was purified by HPLC (Hypercarb column, 150 x10 mm, H_2O (0.1% formic acid) isocratic (5 min), linear gradient to 40% ACN (35 min), linear gradient to 100% ACN (10 min)) and lyophilized to obtain pure pentasaccharide **4** (3.9 mg, 32%). ^{1}H NMR (700 MHz, D_2O) δ 5.39 (dd, *J* = 7.9, 2.0 Hz, 1H), 5.23 (d, *J* = 1.8 Hz, 1H), 5.13 (d, *J* = 1.8 Hz, 1H), 5.05 (d, *J* = 1.7 Hz, 1H), 4.77 (d, *J* = 9.5 Hz, 1H), 4.25 (dd, *J* = 3.3, 1.8 Hz, 1H), 4.17 (ddd, *J* = 7.5, 5.5, 2.7 Hz, 1H), 4.08 (dd, *J* = 3.4, 1.8 Hz, 1H), 4.04 – 3.99 (m, 2H), 3.96 (dd, *J* = 3.4, 2.1 Hz, 1H), 3.91 – 3.86 (m, 4H), 3.85 – 3.77 (m, 6H), 3.75 – 3.67 (m, 7H), 3.66 – 3.58 (m, 6H), 3.58 – 3.55 (m, 1H), 3.52 – 3.46 (m, 2H), 2.98 – 2.93 (m, 2H), 1.68 – 1.55 (m, 4H), 1.46 – 1.34 (m, 2H). ^{13}C NMR (176 MHz, D_2O) δ 100.6, 100.0, 98.7, 97.9, 96.1, 96.1, 79.0, 78.7, 77.5, 74.9, 73.7, 73.3, 73.2, 72.7, 72.5, 70.7,

70.4, 70.1, 69.9, 69.7, 69.6, 67.4, 67.0, 67.0, 66.9, 66.3, 66.2, 64.8, 61.1, 60.9, 60.7, 60.7, 39.3, 27.8, 26.4, 22.3. HR-ESI-MS (m/z): calculated for $C_{35}H_{63}NO_{29}P$ $[M]^-$: 992.3229, found: 992.3167.

Glycan Microarray Screening

The *in vivo* experiments to obtain the mouse plasma used in this study were published previously.[1] Briefly, healthy C57Bl6 male mice (aged 8-9 weeks) were i.v. challenged through the tail vein with 1×10^7 c.f.u. per mouse log-phase inoculum *C. auris* 10051895 $n = 6$ and three days after infection plasma was collected and stored at -80°C.

The glycans were dissolved at 0.1 mM in 50 mM sodium phosphate buffer pH 8.5 and printed in 64 identical fields to NHS activated hydrogel glass slides (CodeLink slides, Surmodics) using a non-contact S3 microarray spotter (Scienion, Berlin, Germany). After incubation overnight in a humidified box, the remaining NHS groups of the slides were quenched with ethanolamine. The slides were blocked with 1% (w/v) bovine serum albumin (BSA) in phosphate buffered saline (PBS) and a 64 well incubation gasket (FlexWell Grid, Grace Bio Labs) was attached. The slides were incubated with mice plasma diluted 1:50 in 1% BSA-PBS for 1 h at 37°. After three washes with PBS containing 0.1% (v/v) Tween-20 (PBS-T) the slides were incubated with goat anti-mouse IgM (heavy chain) secondary antibody, Alexa Fluor™ 647 (Invitrogen, Cat. A21238) diluted 1:400 for 1 h at 37°C. The slides were washed twice with PBS-T. After removing the gasket, the slides were washed once with PBS and once with water. The dried slides were scanned with a GenePix 4300A microarray scanner (Molecular Devices). Intensities were evaluated with GenePix Pro 7 (Molecular Devices). The statistical analysis was performed with the software GraphPad Prism 9.3.1 (GraphPad Software, Inc.).

^{1}H NMR (400 MHz, CDCl$_3$)

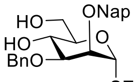

8

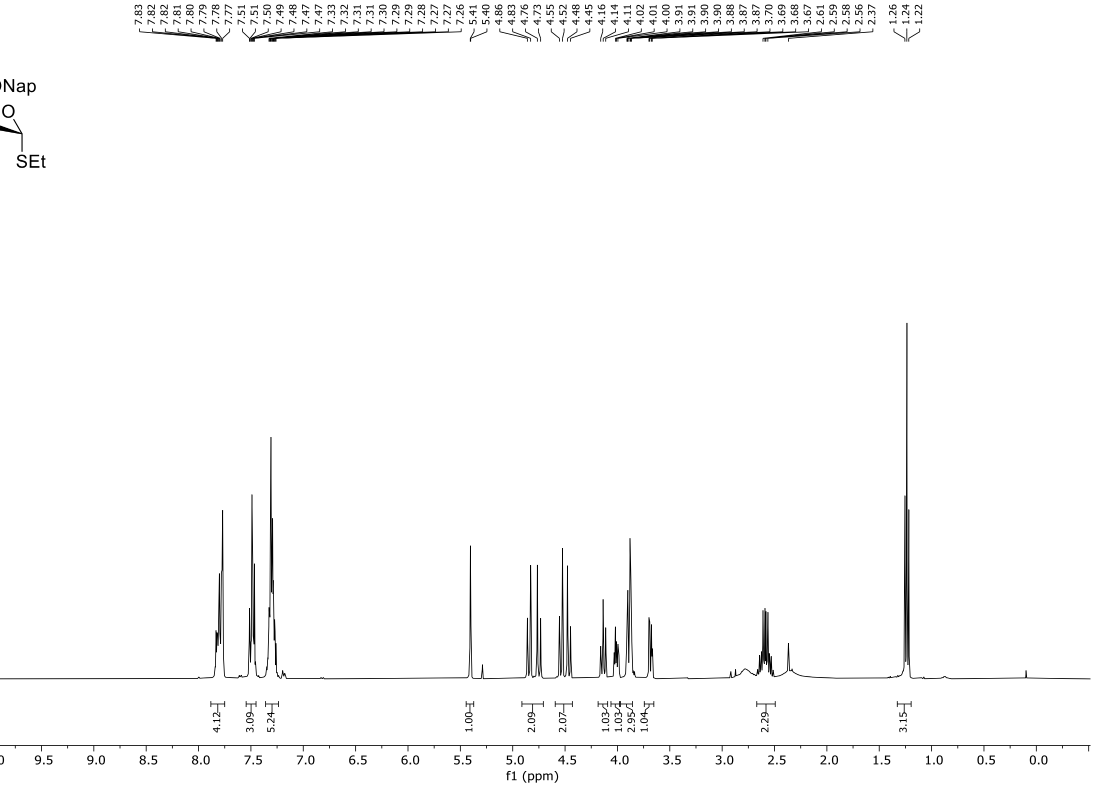

^{13}C NMR (101 MHz, CDCl$_3$)

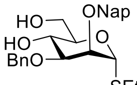

8

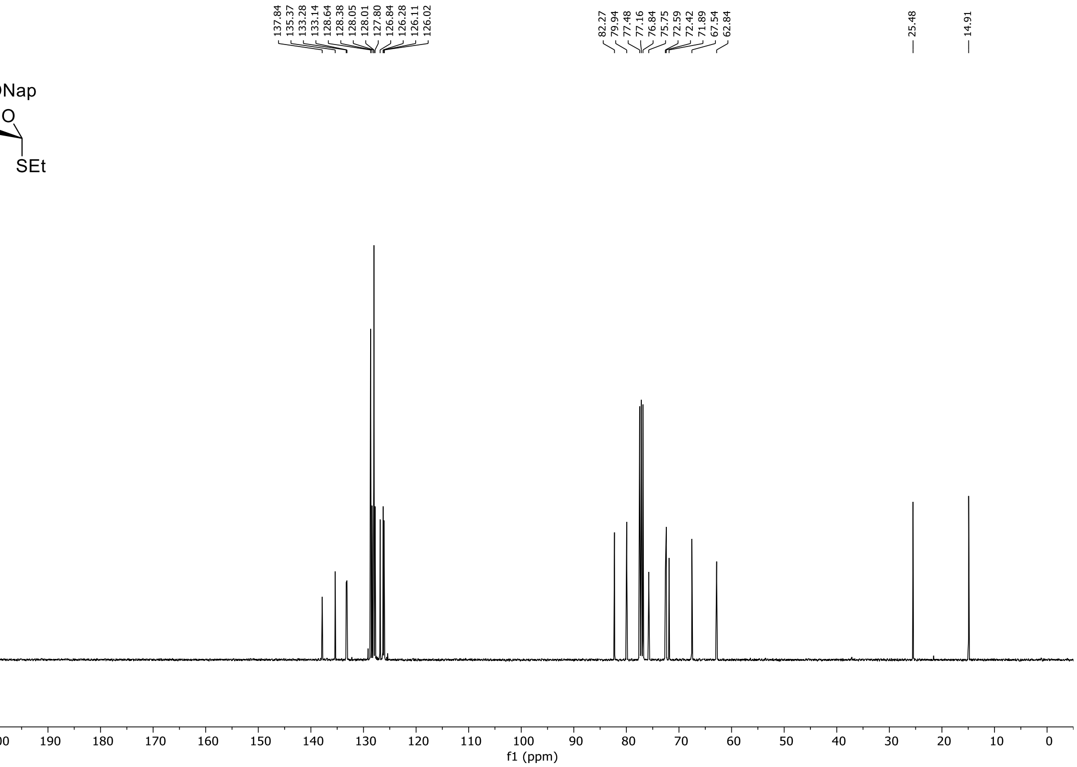

^1H-^1H COSY NMR (400 MHz, $CDCl_3$)

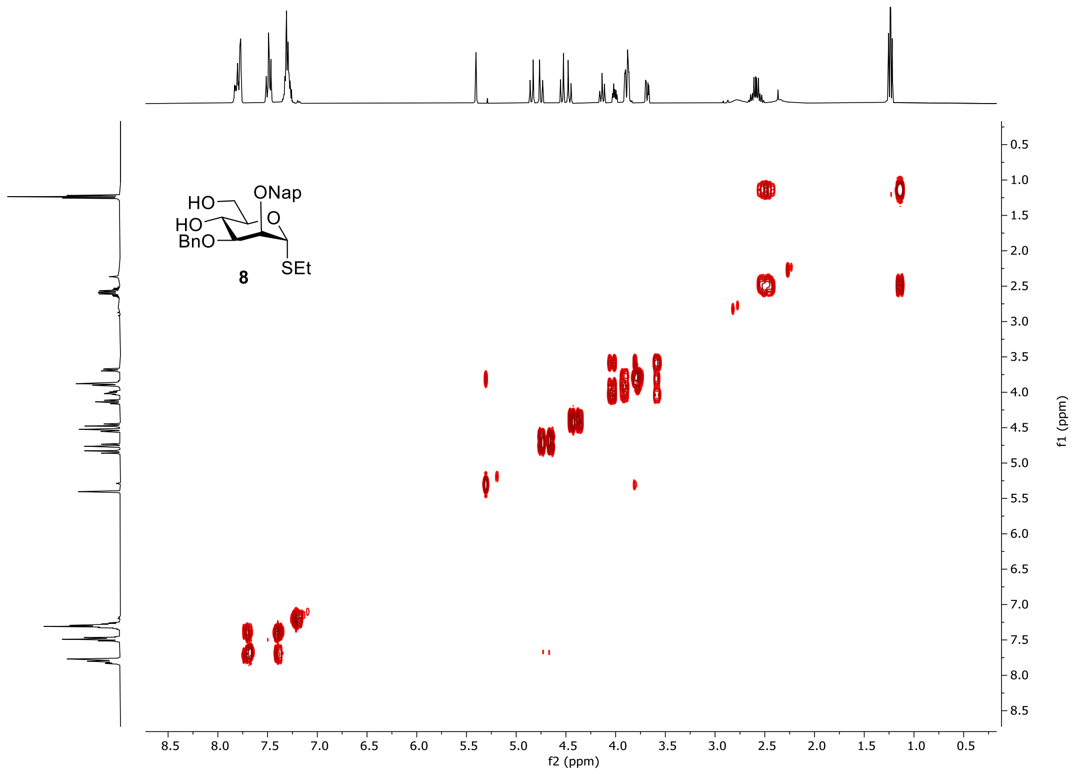

^1H-^{13}C HSQC NMR (400 MHz, $CDCl_3$)

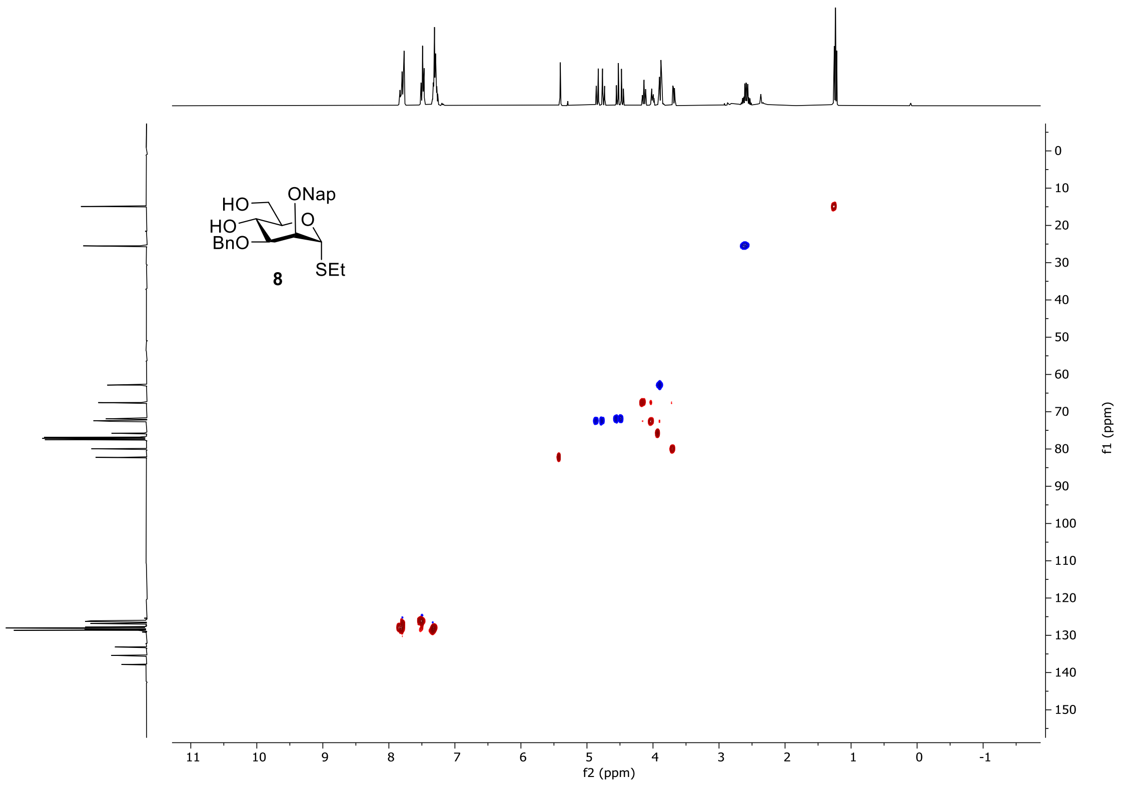

^{1}H NMR (400 MHz, $CDCl_3$)

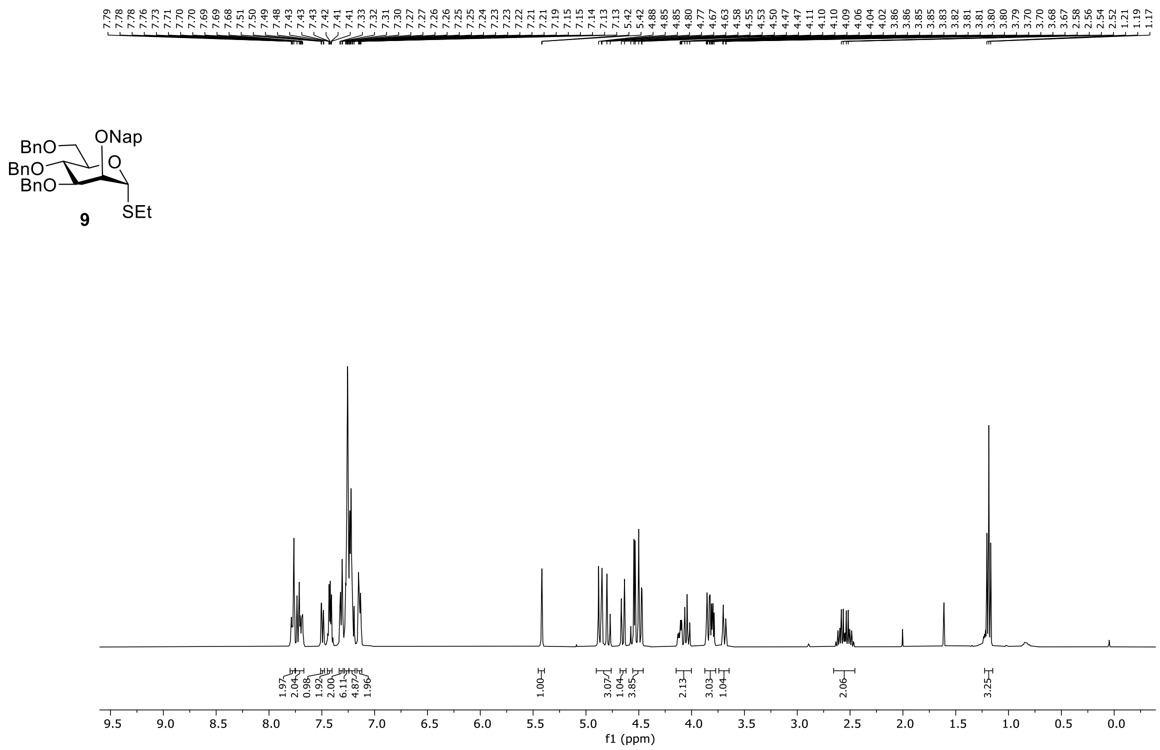

^{13}C NMR (101 MHz, $CDCl_3$)

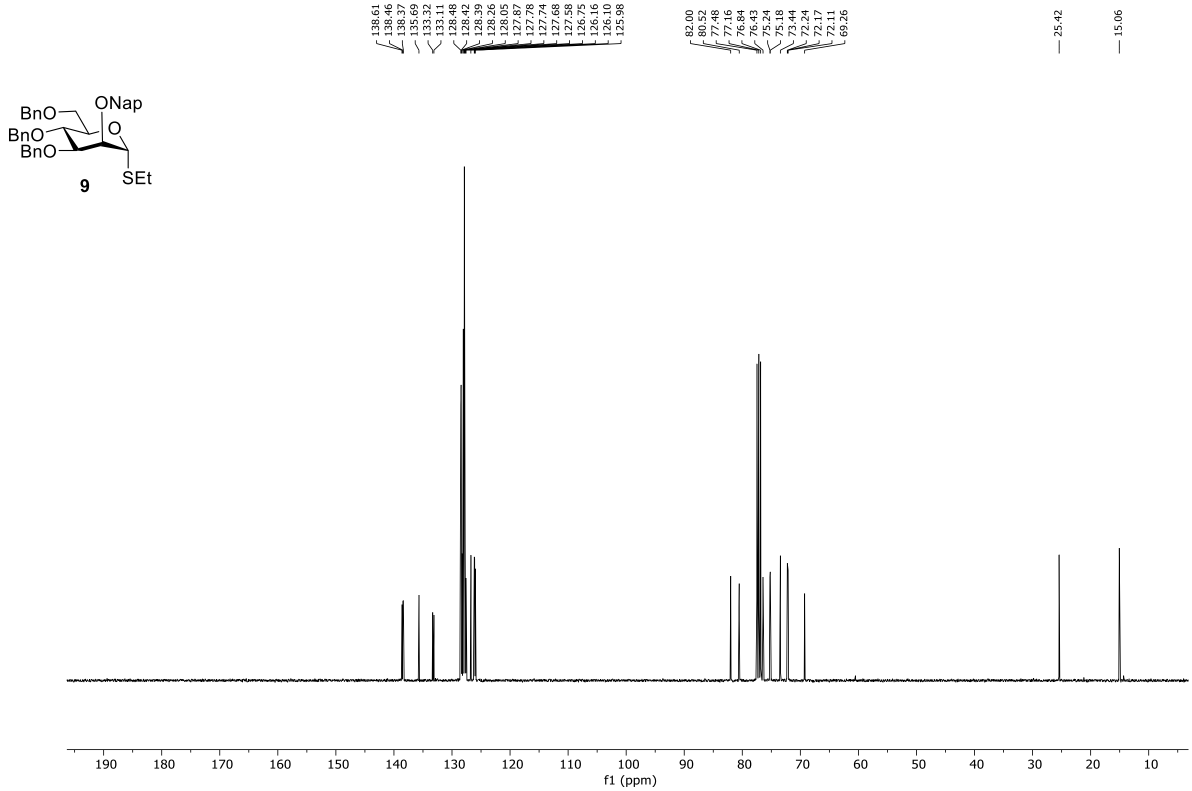

^1H-^1H COSY NMR (400 MHz, $CDCl_3$)

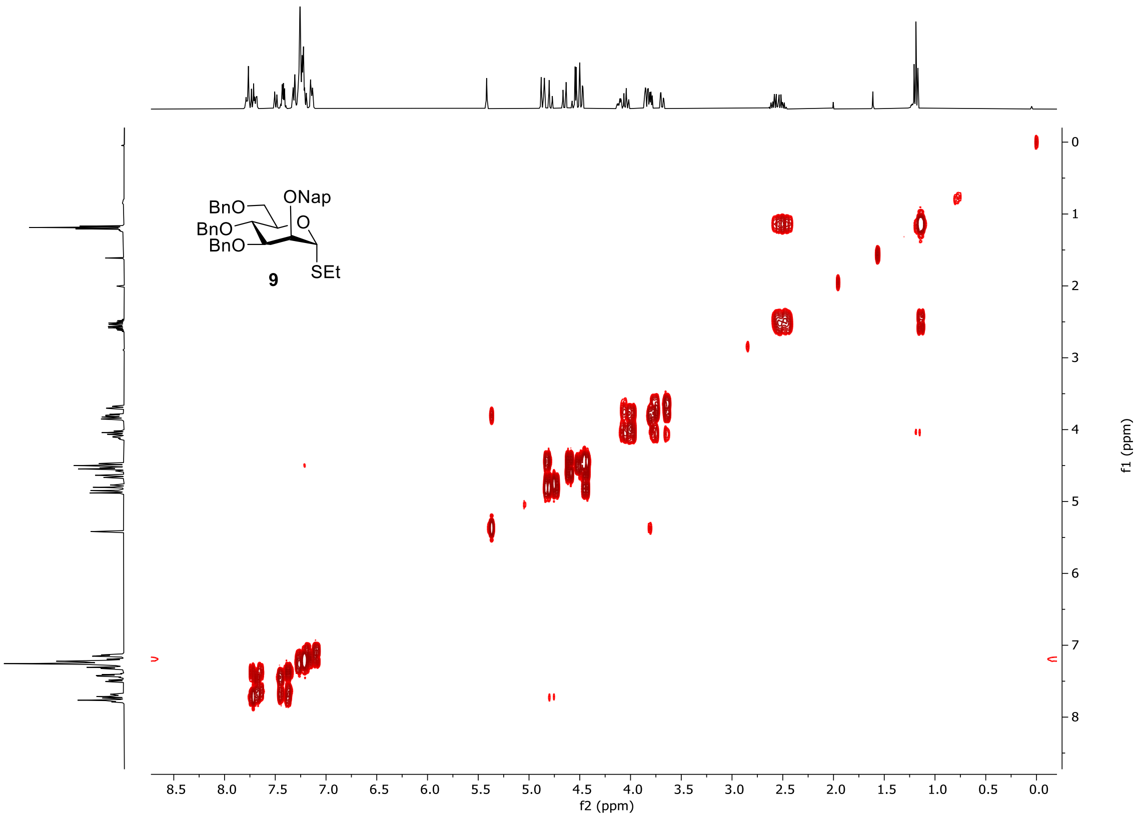

^1H-^{13}C HSQC NMR (400 MHz, $CDCl_3$)

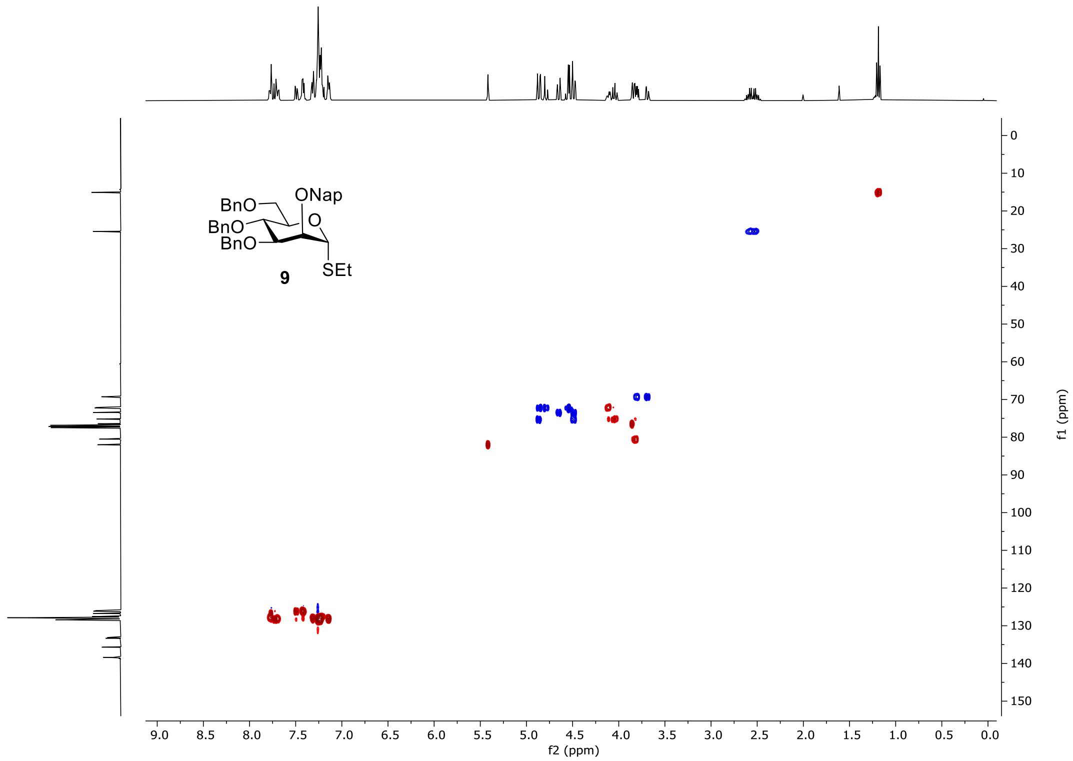

^{1}H NMR (400 MHz, $CDCl_3$)

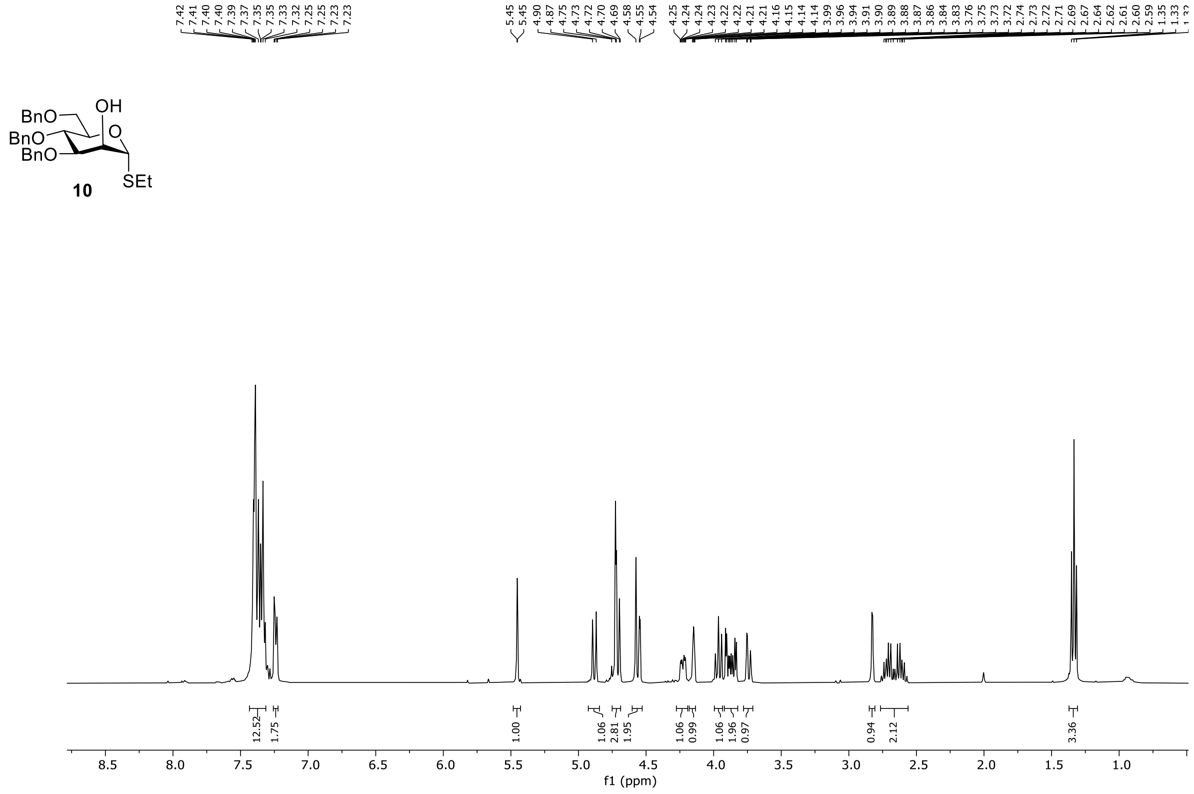

^{13}C NMR (101 MHz, $CDCl_3$)

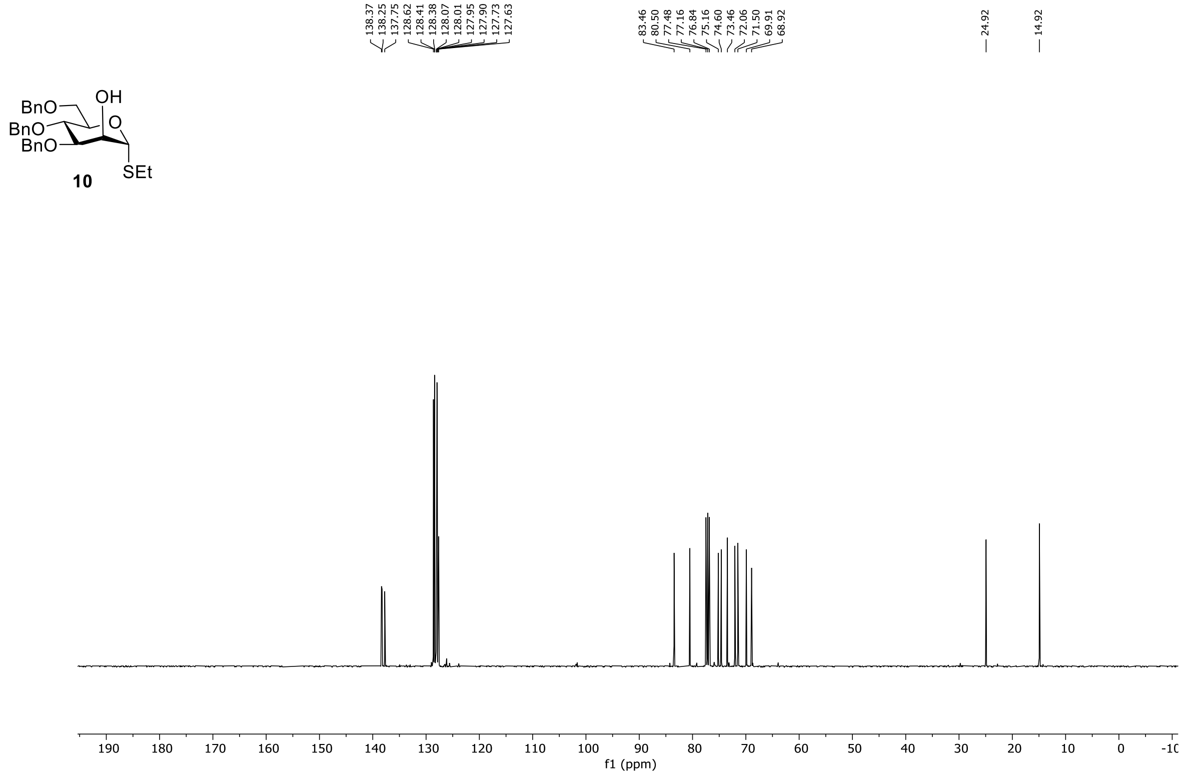

^{1}H-^{1}H COSY NMR (400 MHz, $CDCl_3$)

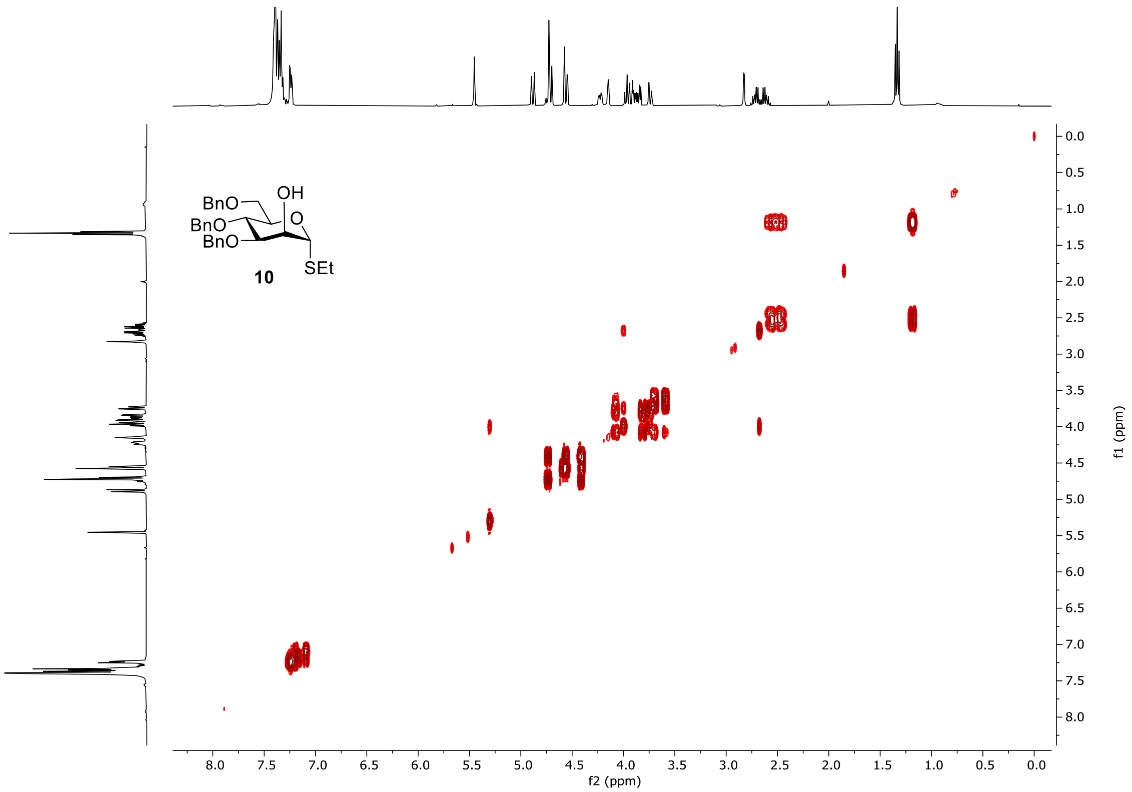

^{1}H-^{13}C HSQC NMR (400 MHz, $CDCl_3$)

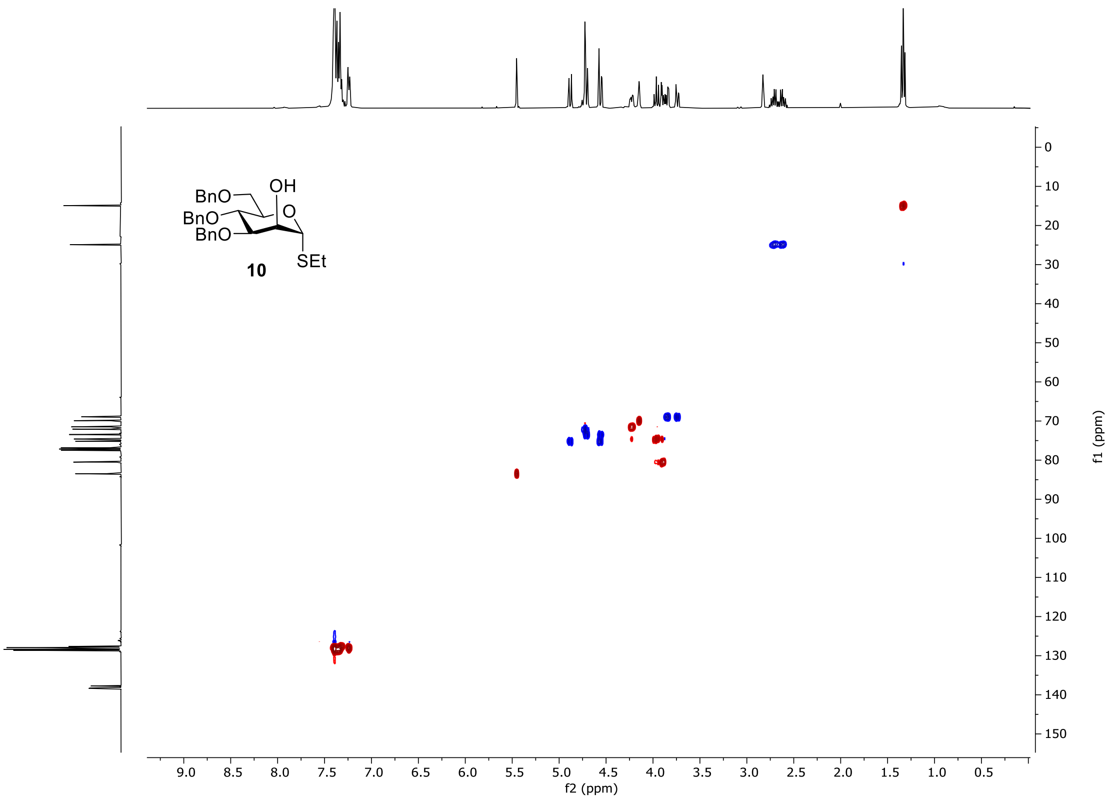

^{1}H NMR (400 MHz, CDCl$_3$)

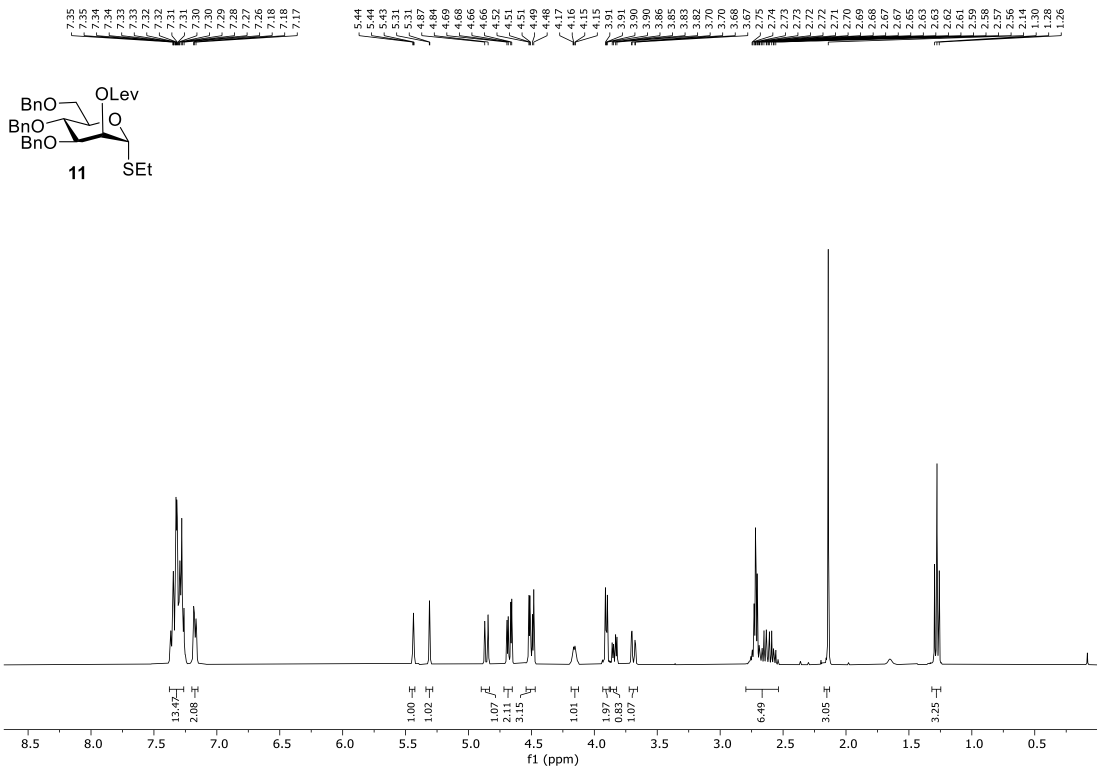

^{13}C NMR (101 MHz, CDCl$_3$)

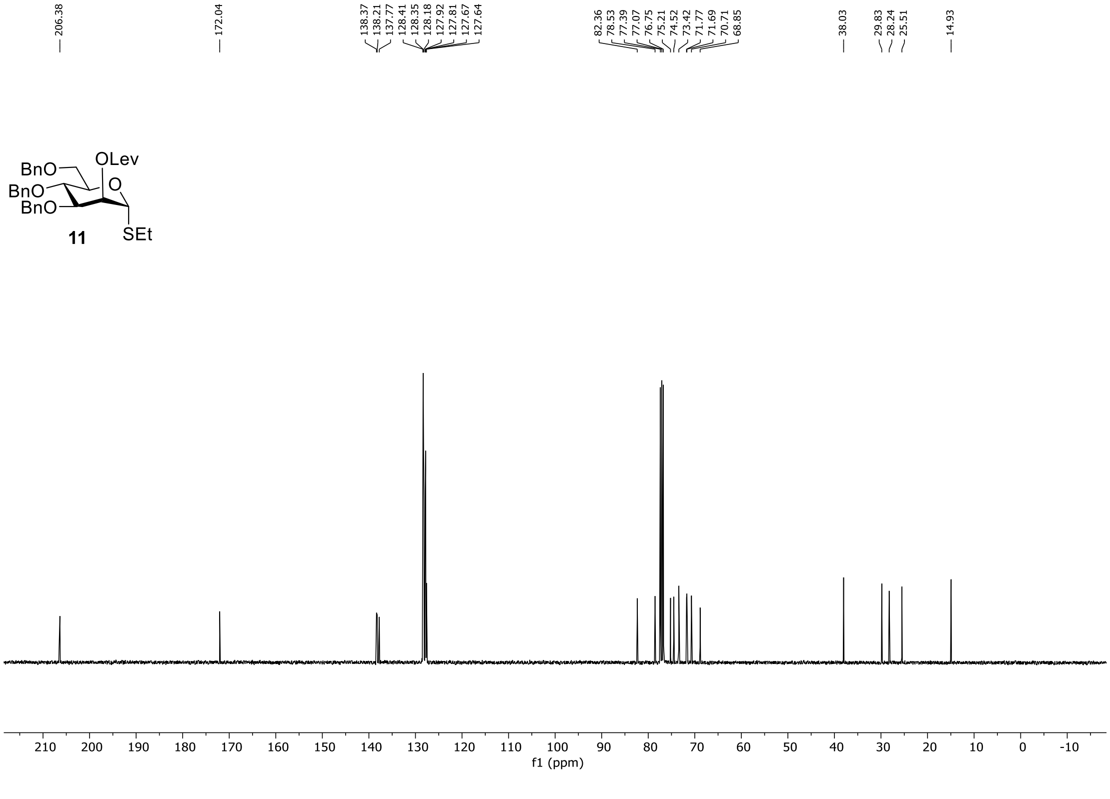

^{1}H NMR (400 MHz, $CDCl_3$)

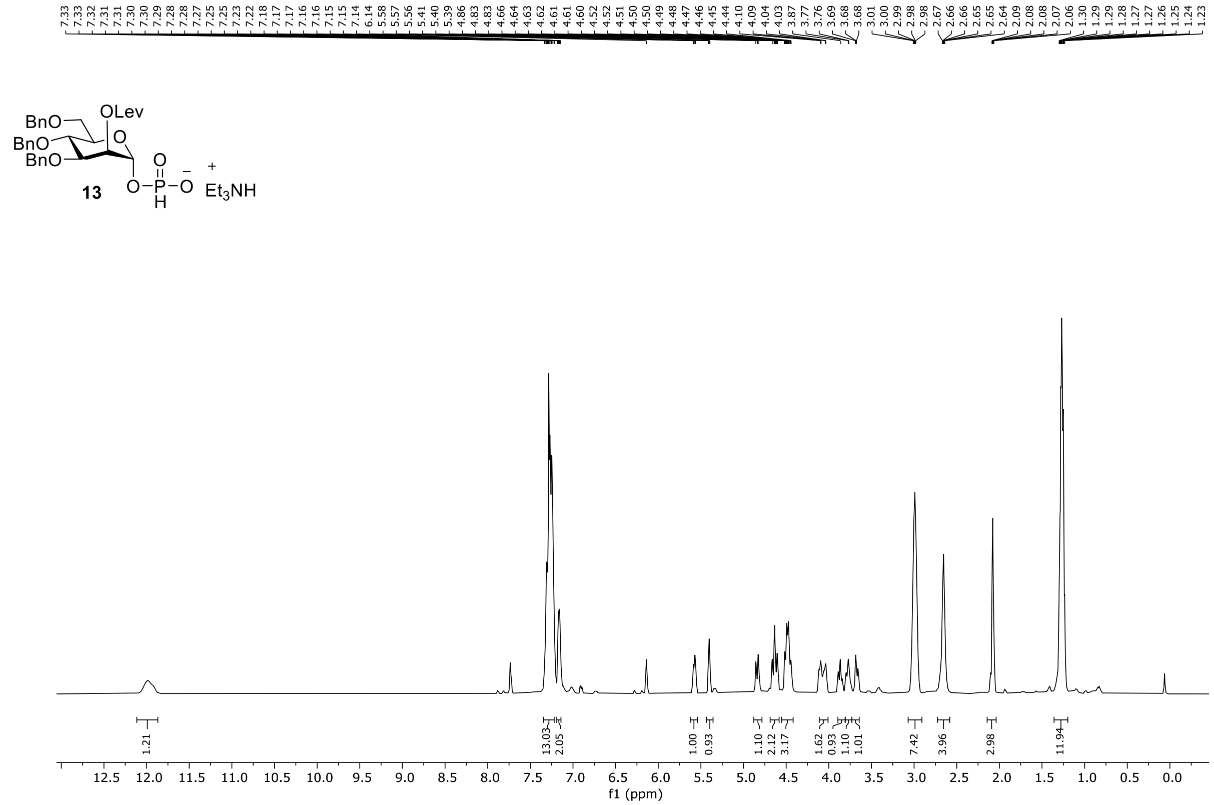

^{13}C NMR (101 MHz, $CDCl_3$)

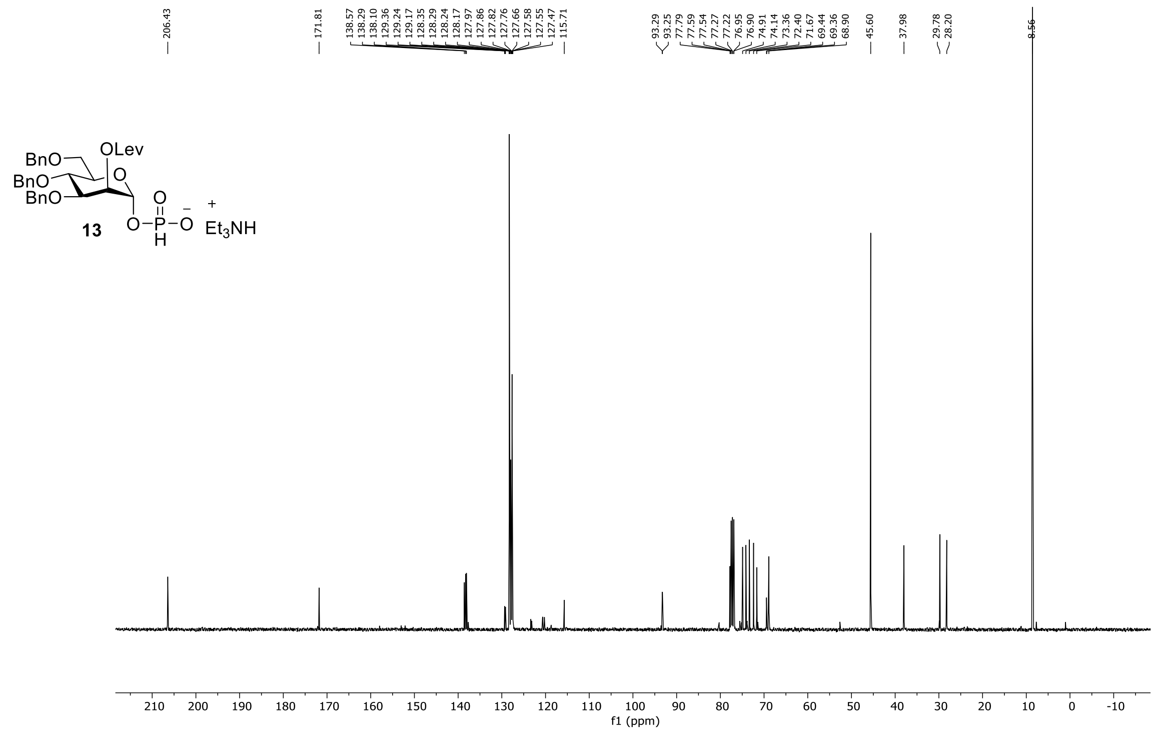

^{1}H-^{13}C HSQC NMR (400 MHz, $CDCl_3$)

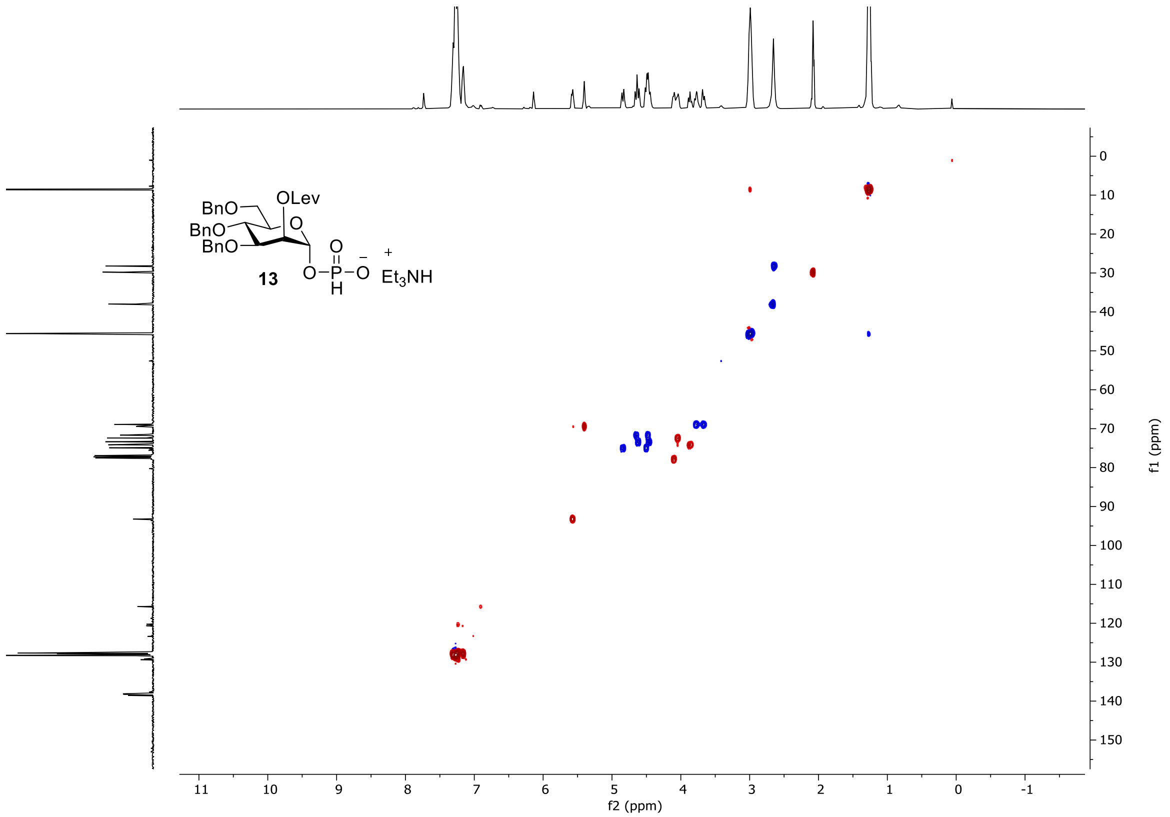

^{31}P NMR (162 MHz, $CDCl_3$)

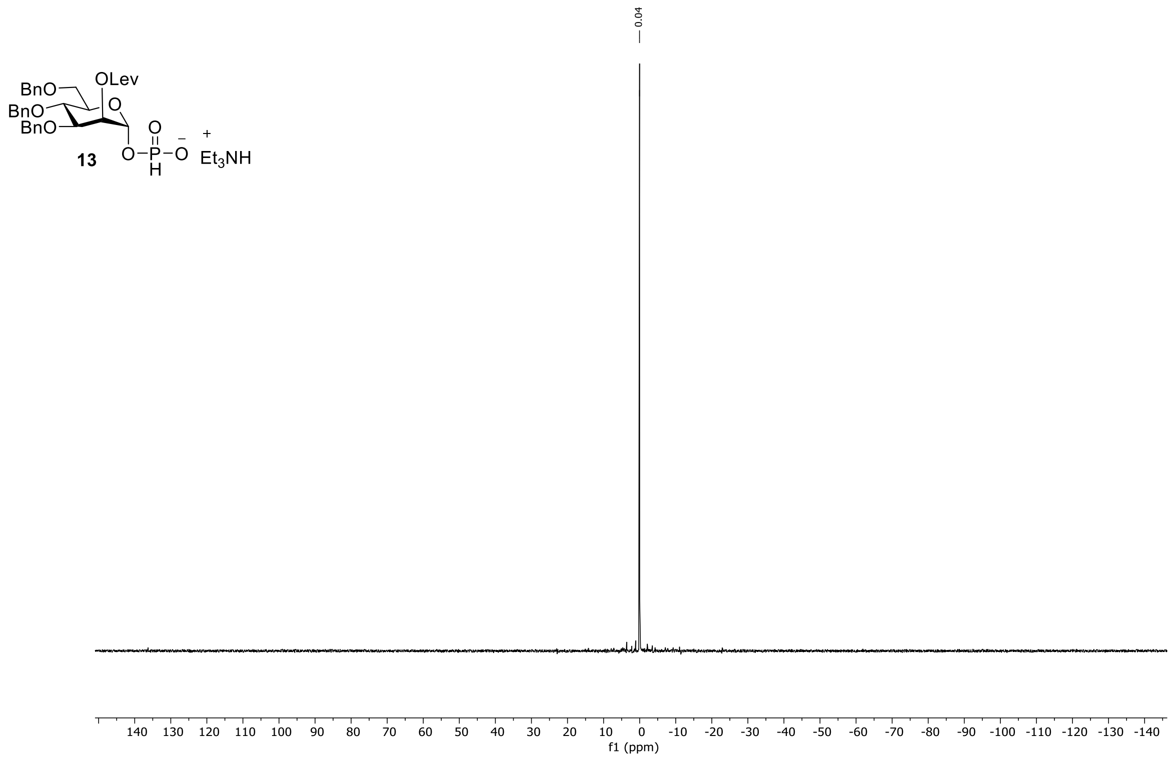

^{1}H NMR (400 MHz, $CDCl_3$)

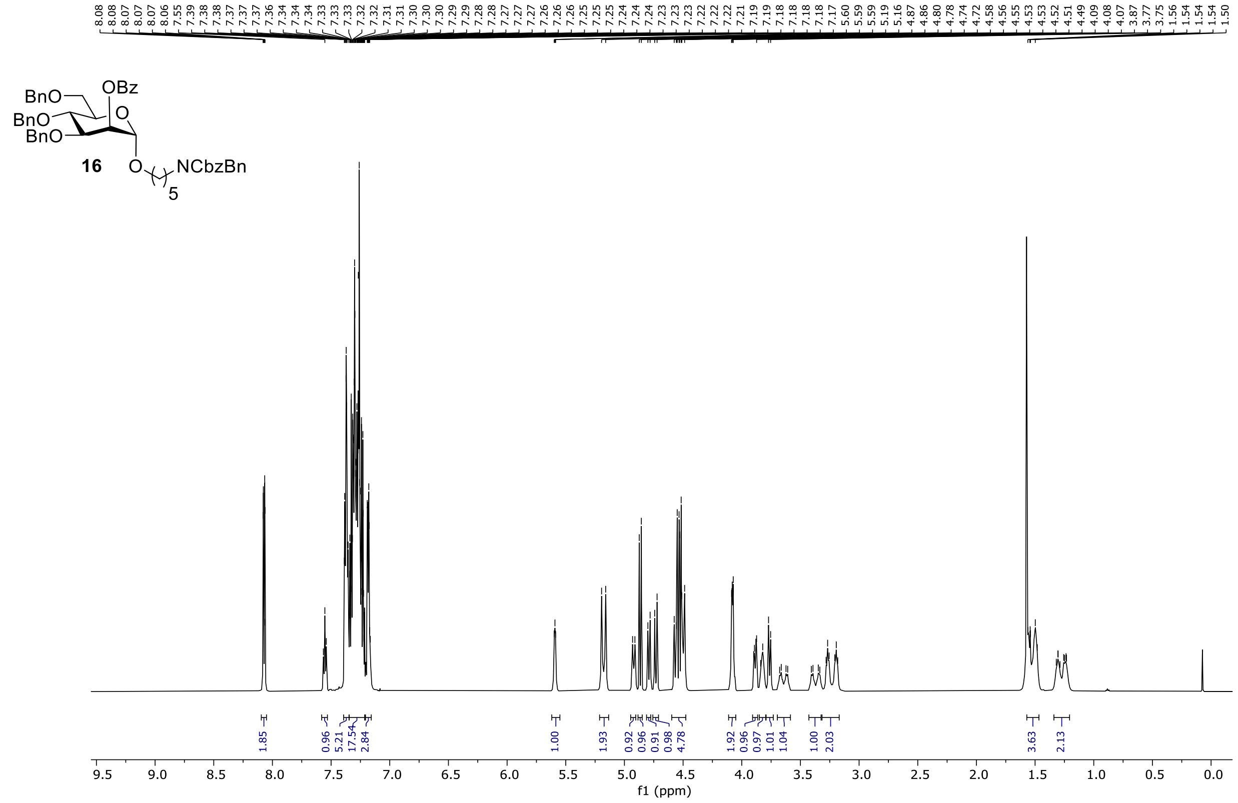

^{13}C NMR (101 MHz, $CDCl_3$)

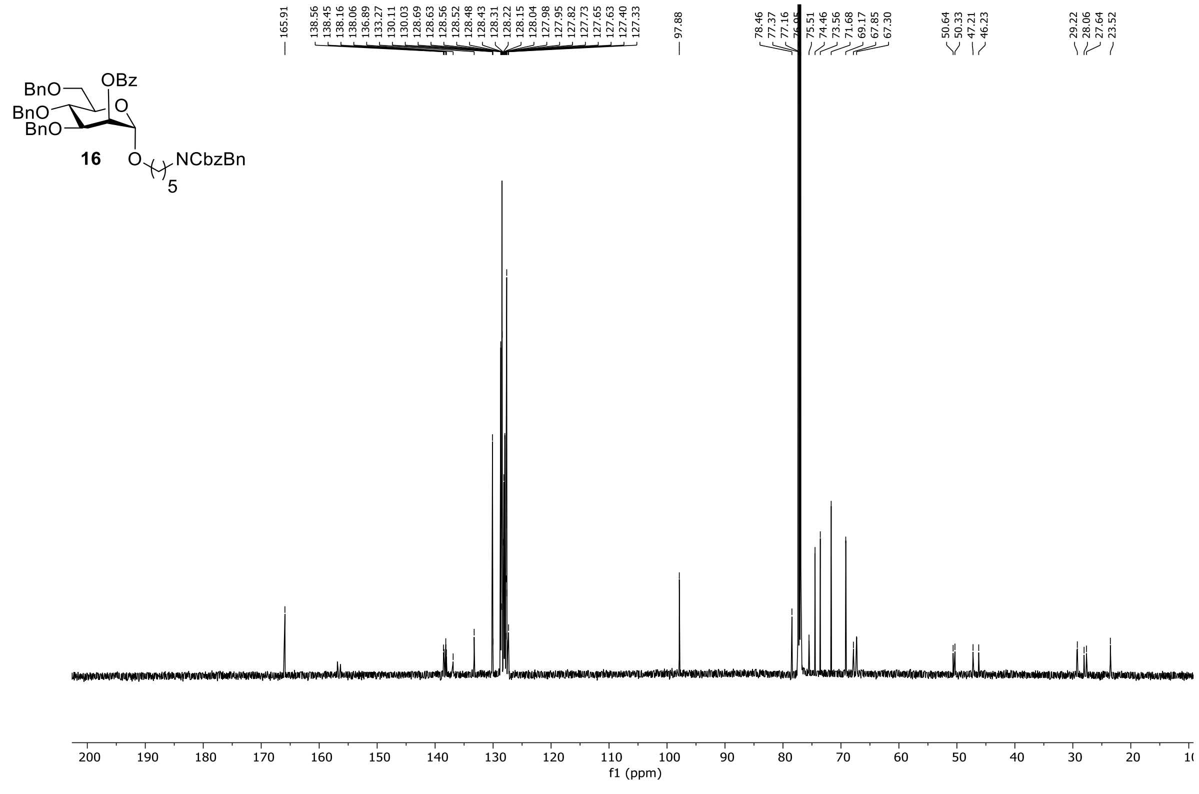

^1H-^1H COSY NMR (400 MHz, $CDCl_3$)

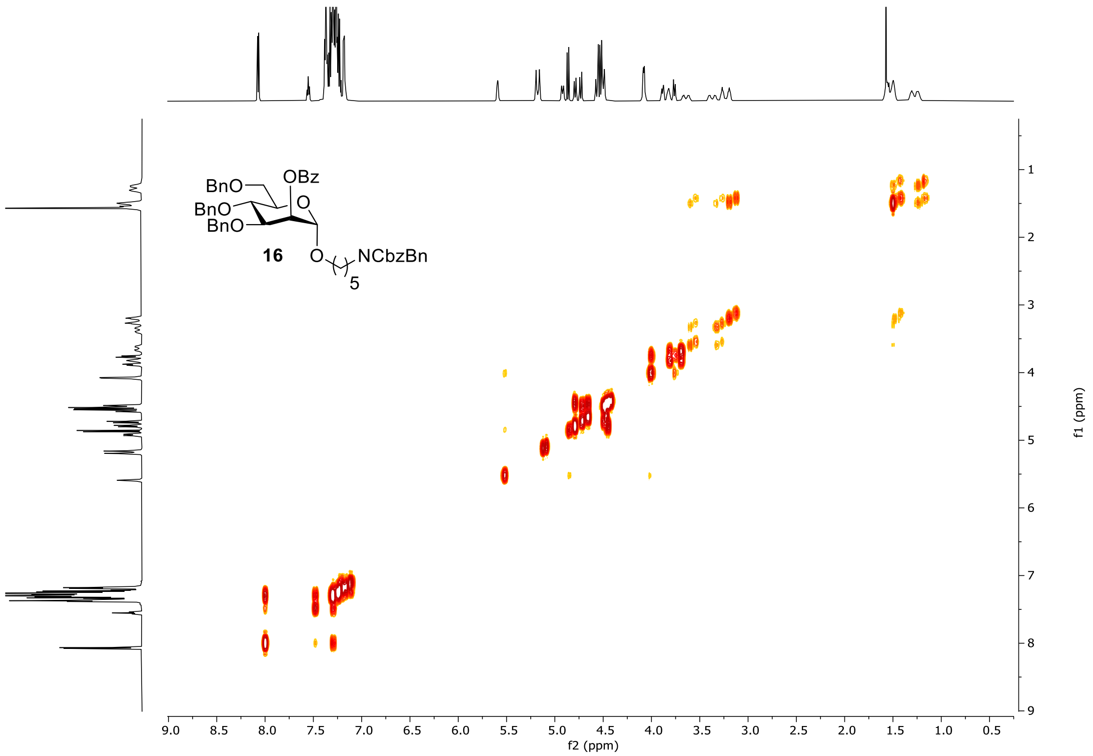

^1H-^{13}C HSQC NMR (400 MHz, $CDCl_3$)

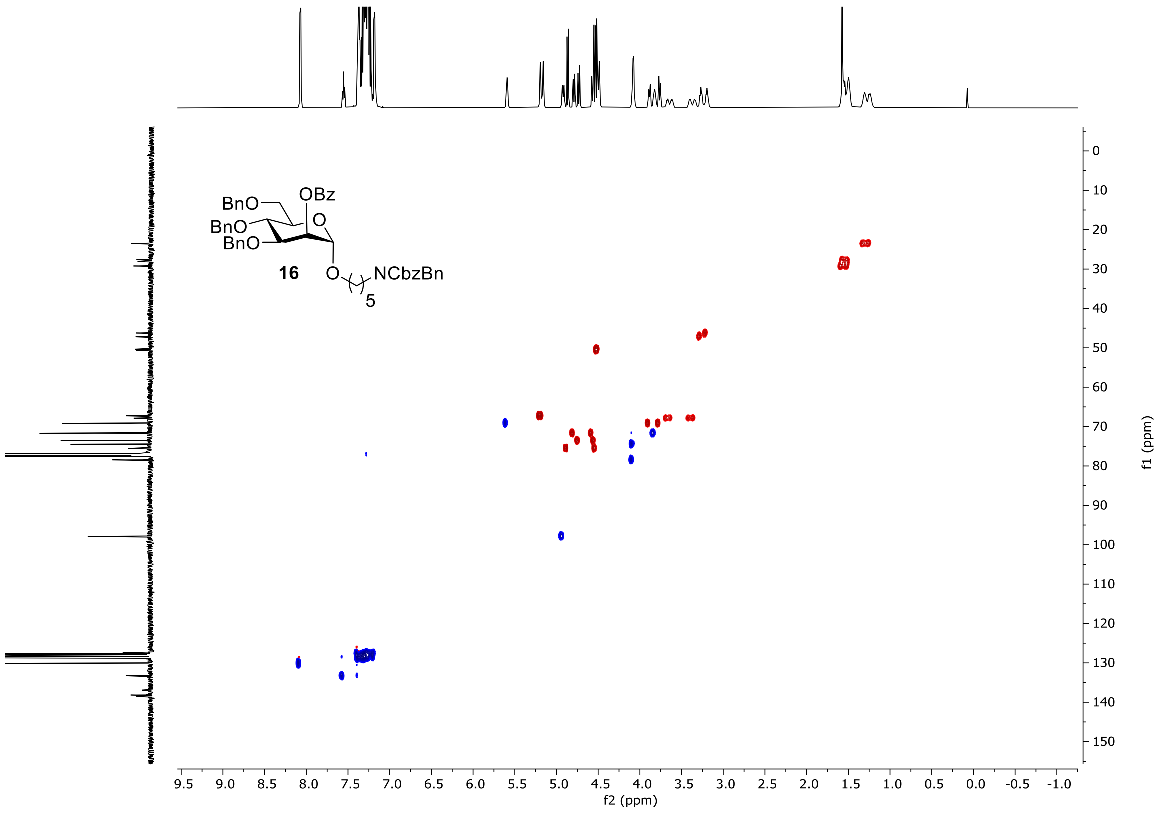

^{1}H-^{13}C Coupled HSQC NMR (400 MHz, $CDCl_3$)

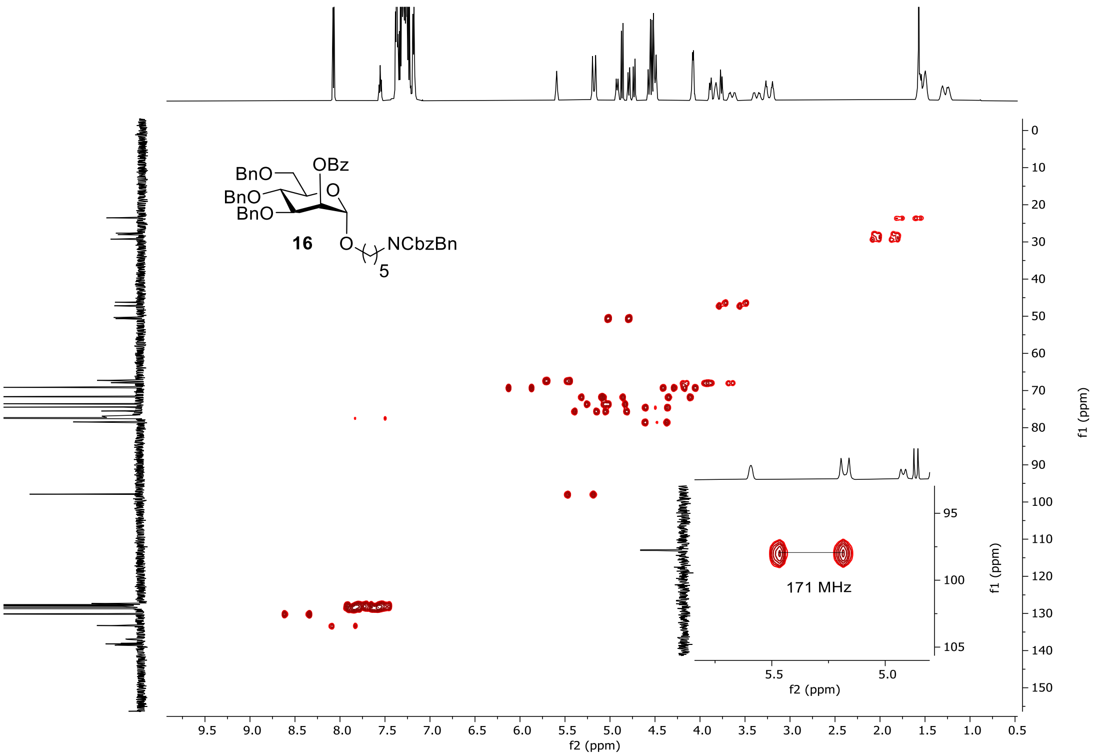

^{1}H NMR (400 MHz, $CDCl_3$)

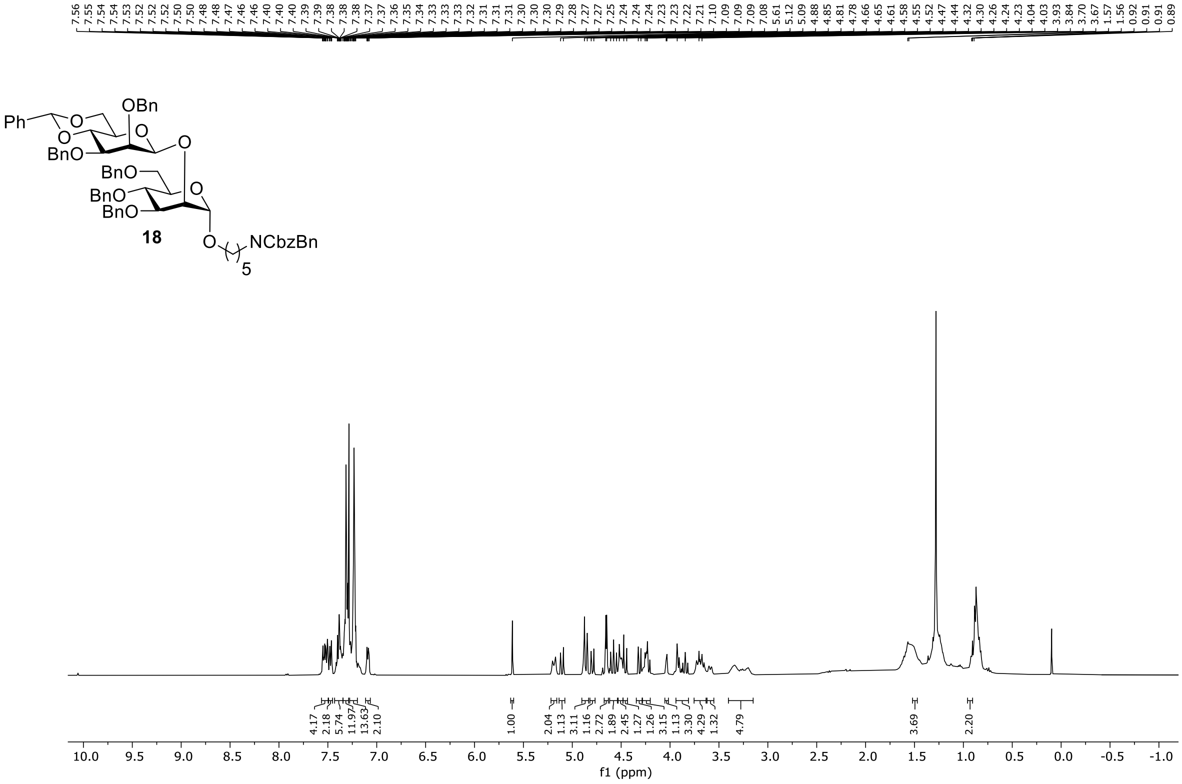

^{13}C NMR (101 MHz, $CDCl_3$)

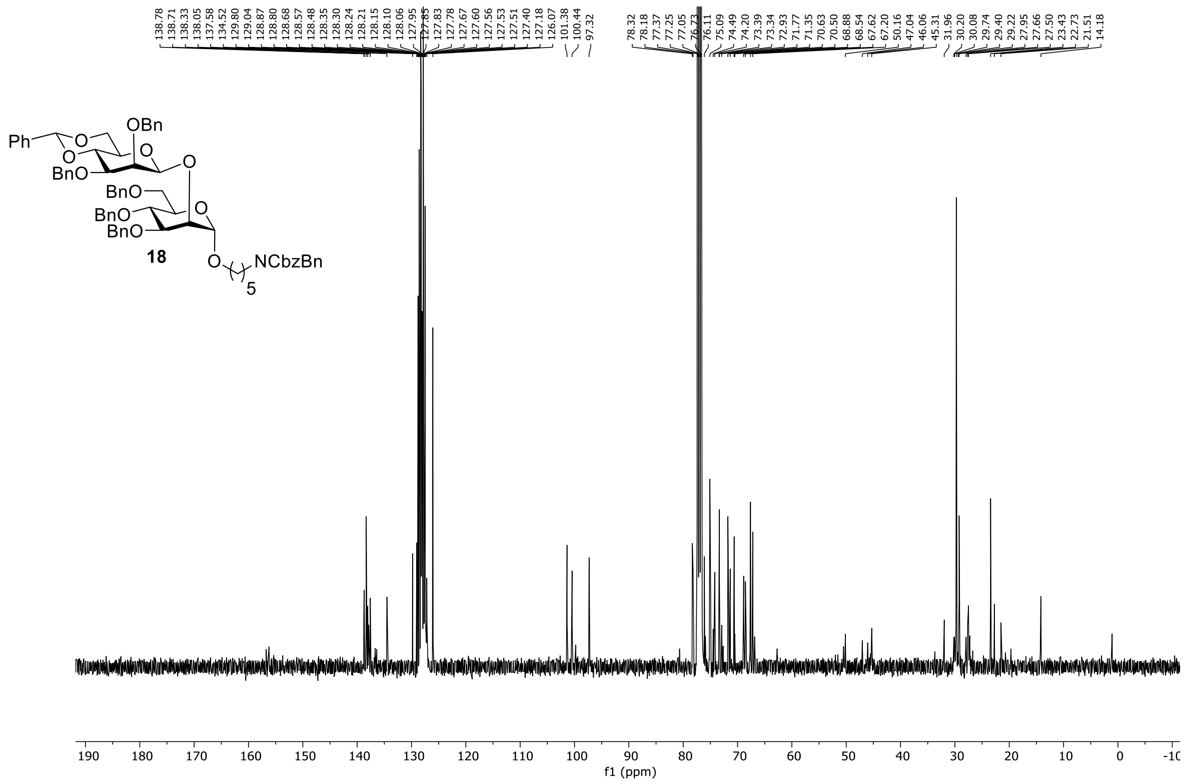

^{1}H-^{1}H COSY NMR (400 MHz, $CDCl_3$)

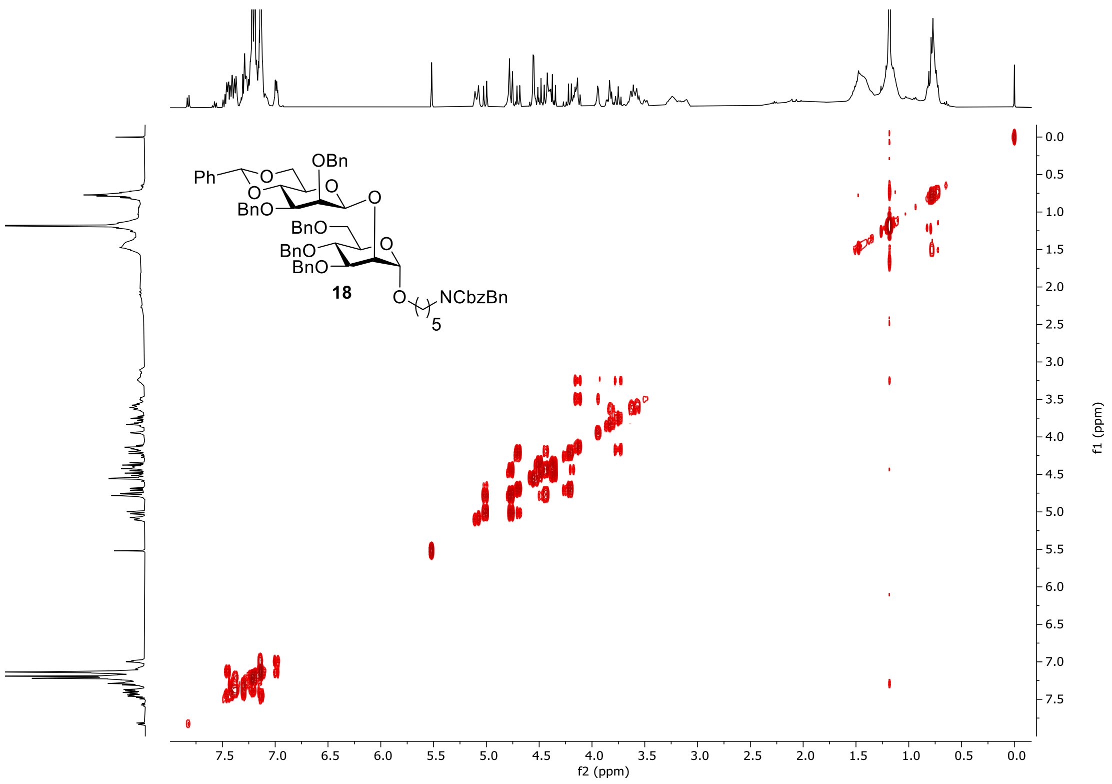

^{1}H-^{13}C HSQC NMR (400 MHz, $CDCl_3$)

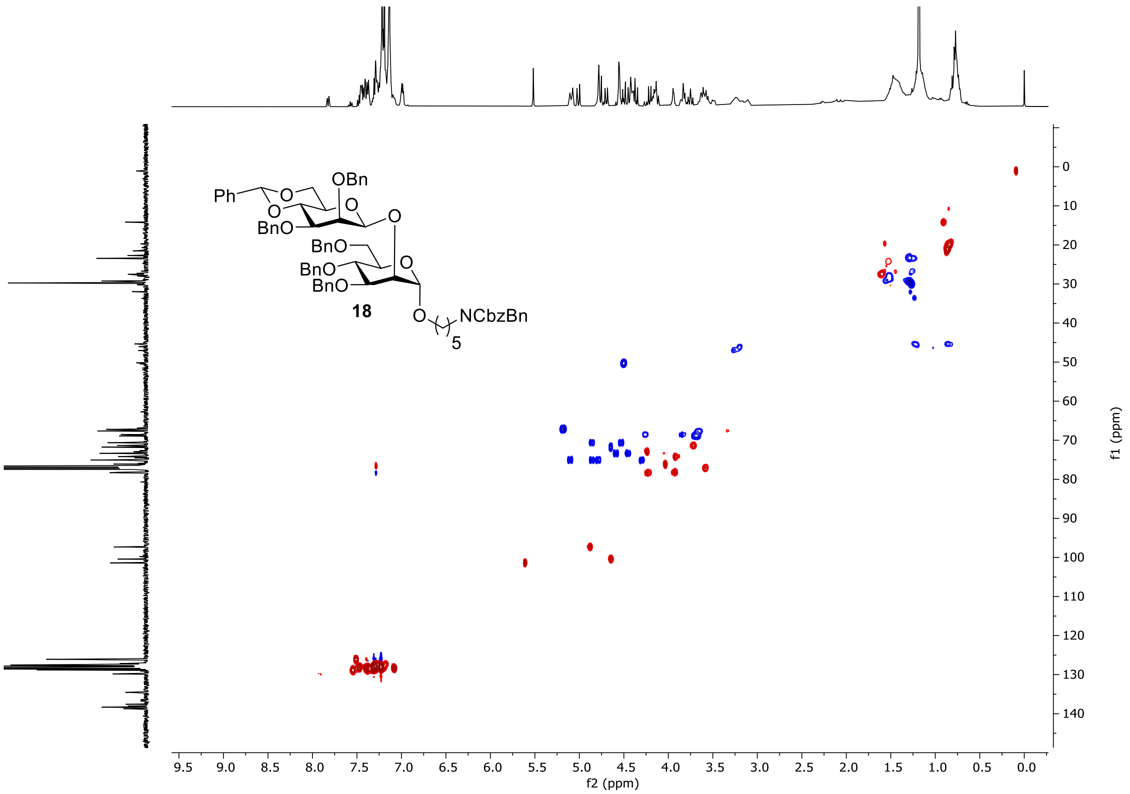

^{1}H-^{13}C Coupled HSQC NMR (400 MHz, $CDCl_3$)

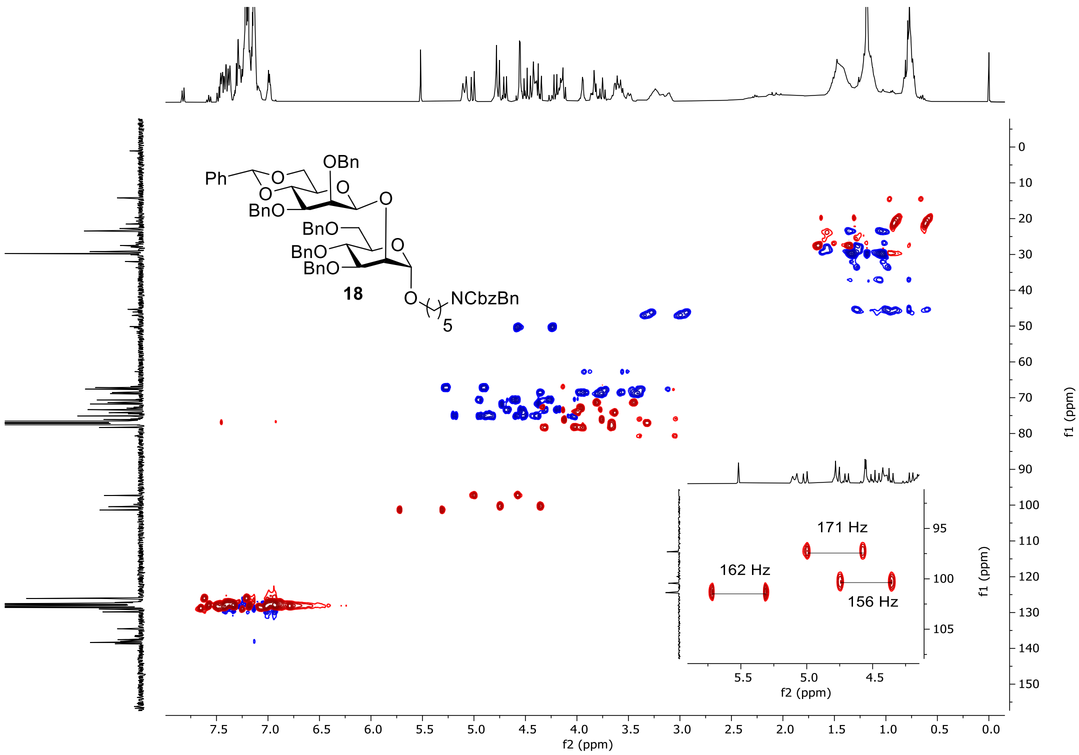

^{1}H NMR (700 MHz, D_2O)

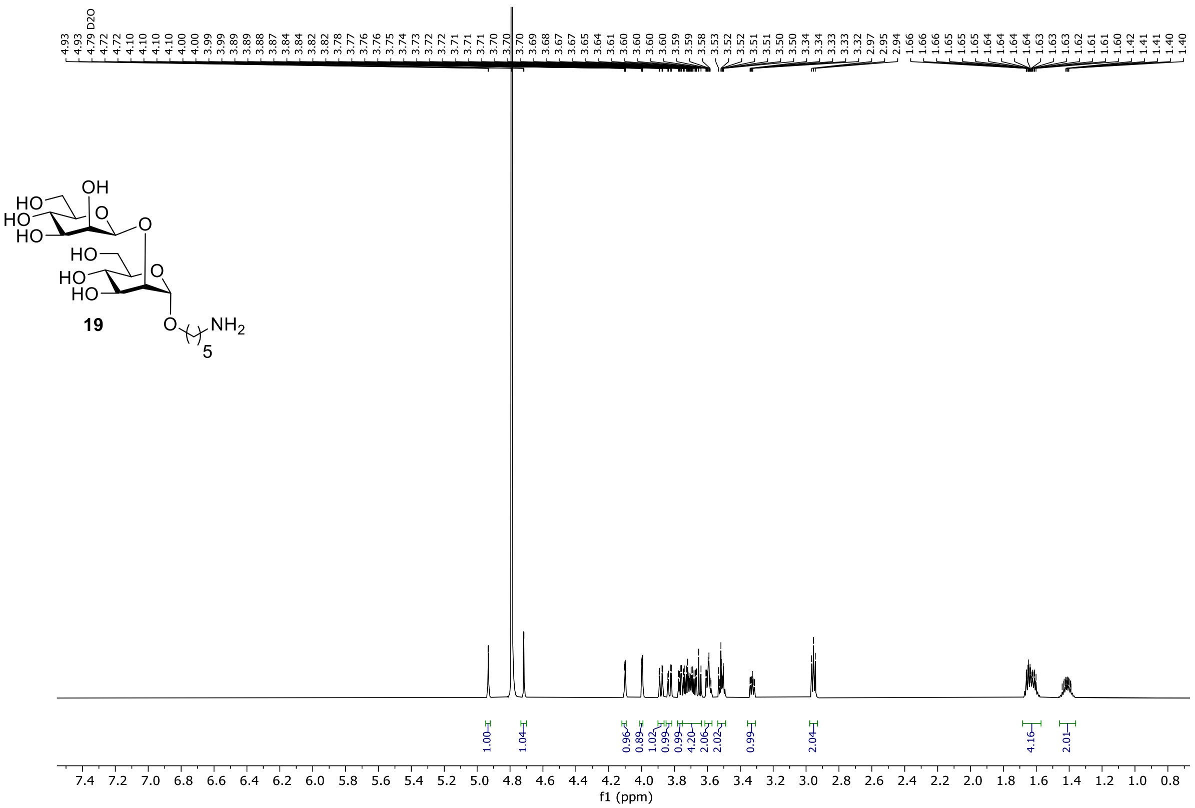

19

^{13}C NMR (176 MHz, D_2O)

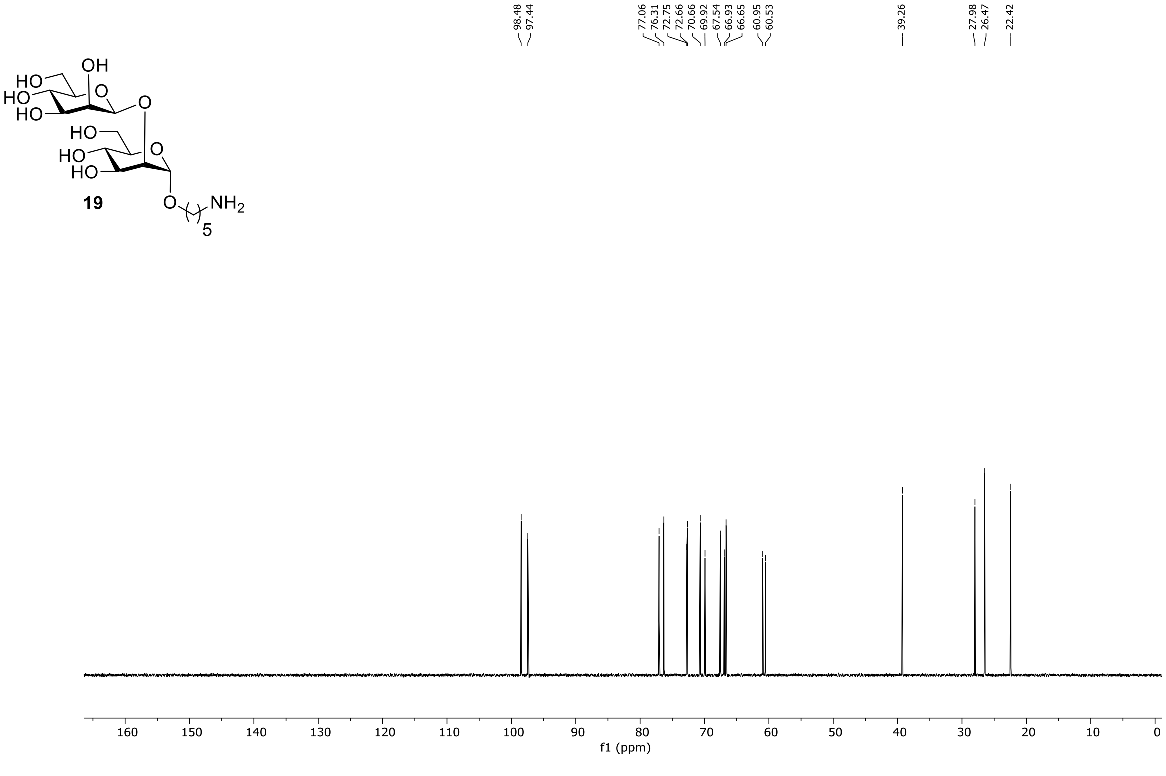

19

^{1}H-^{1}H COSY NMR (700 MHz, D_2O)

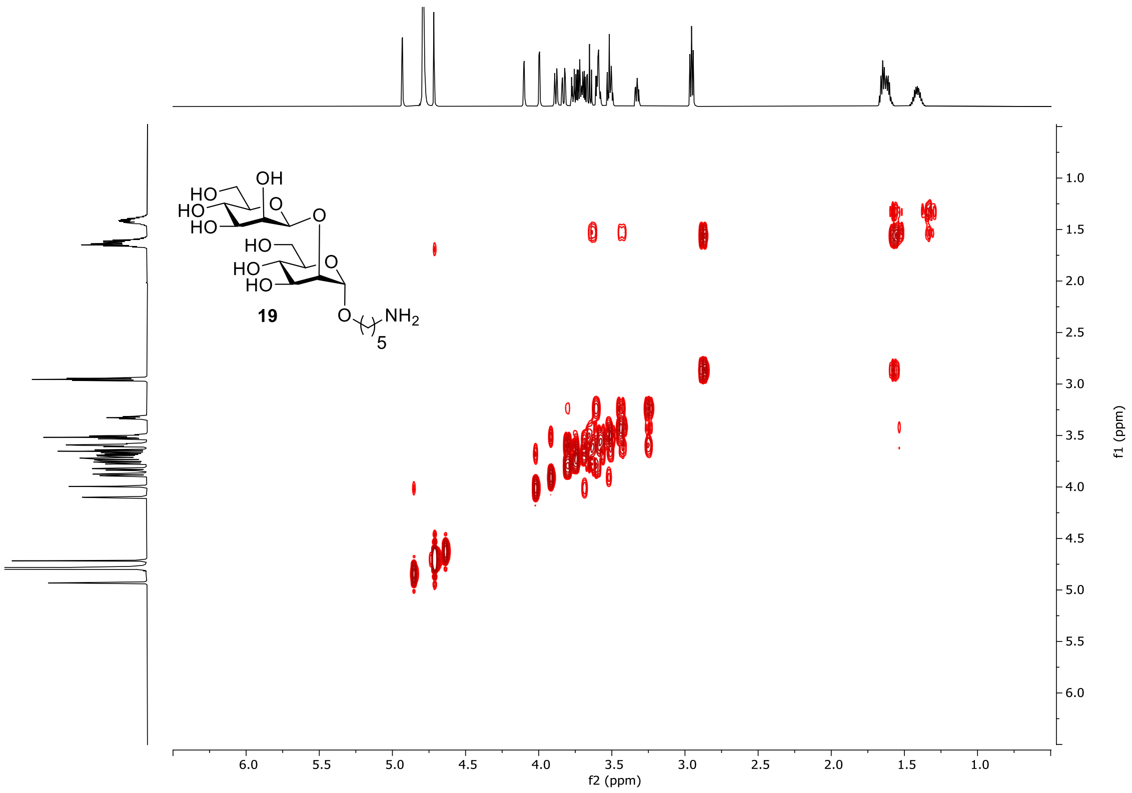

^{1}H-^{13}C HSQC NMR (700 MHz, D_2O)

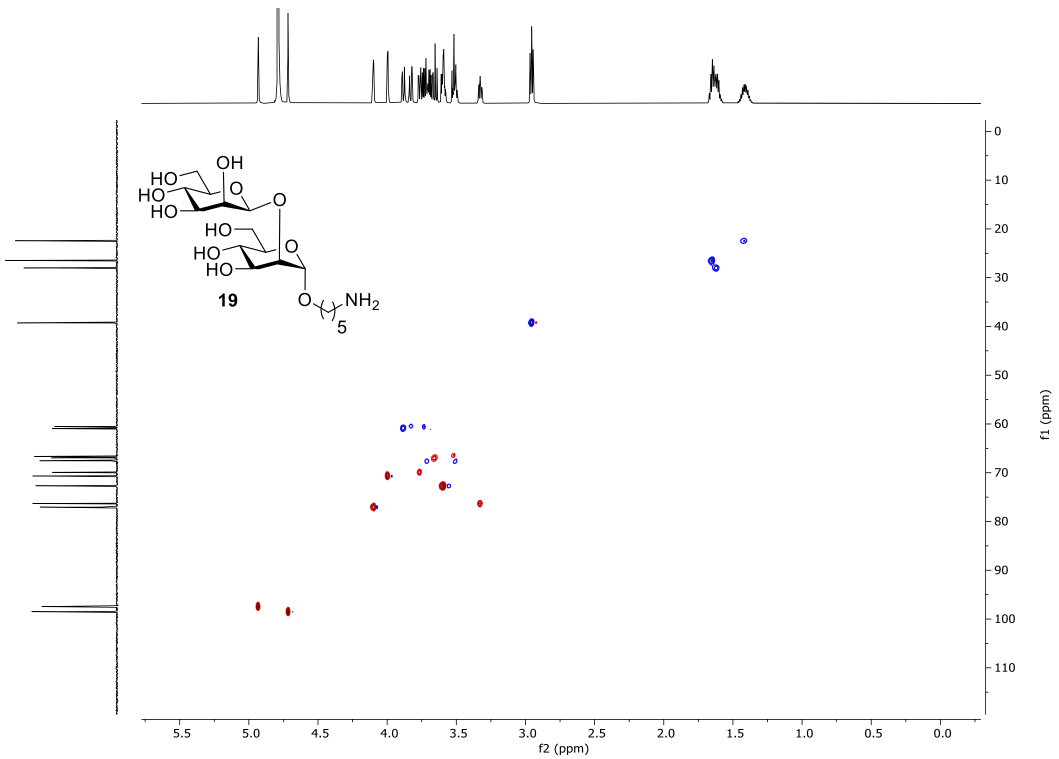

^{1}H-^{13}C Coupled HSQC NMR (700 MHz, D_2O)

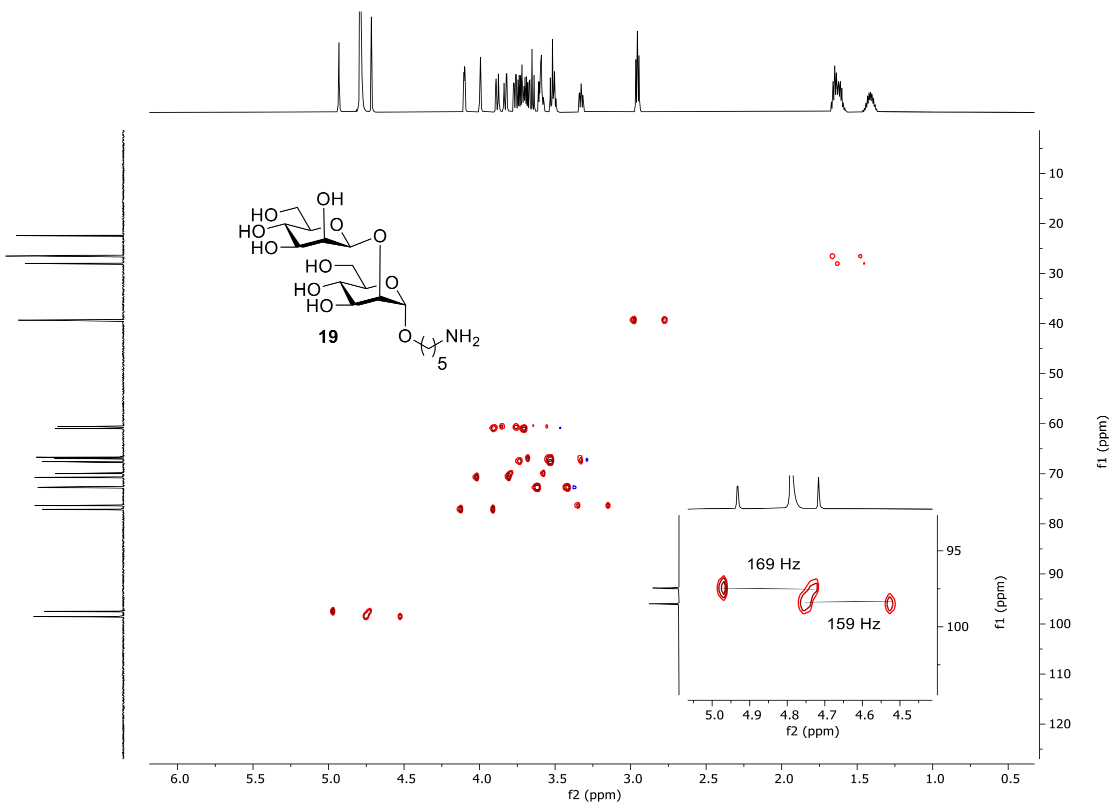

^{1}H NMR (400 MHz, $CDCl_3$)

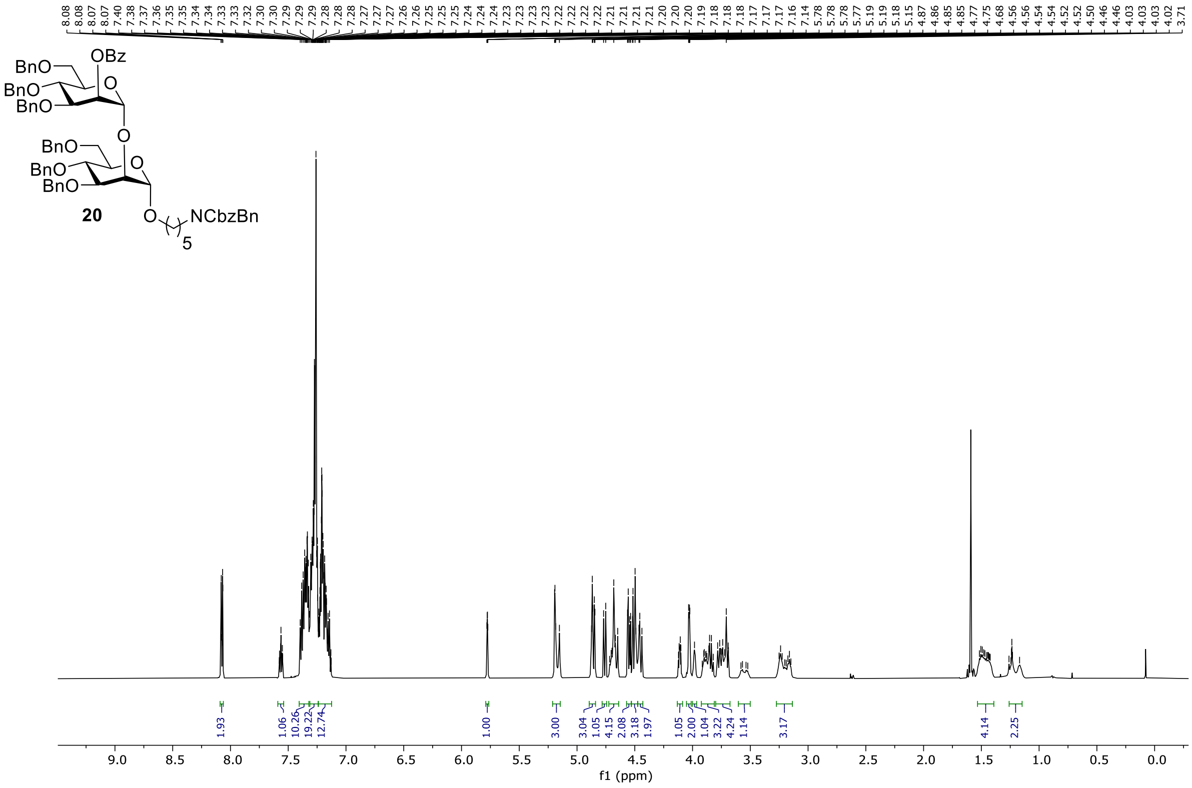

^{13}C NMR (101 MHz, $CDCl_3$)

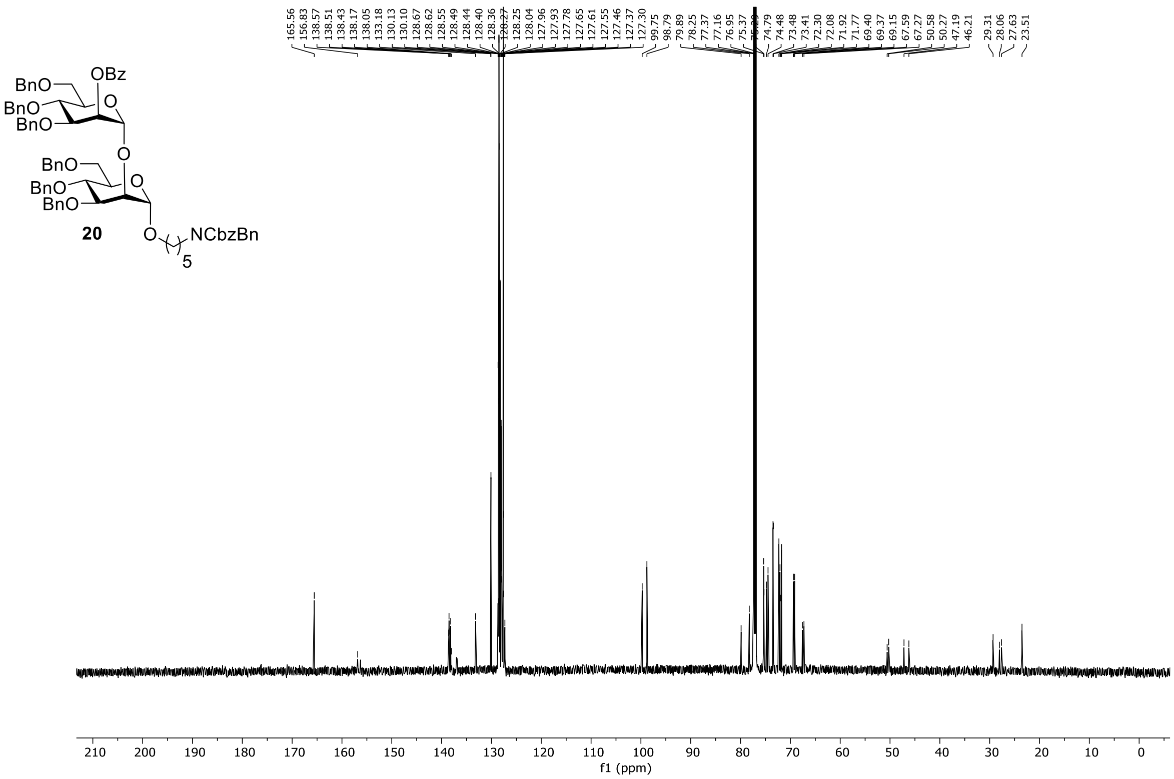

^{1}H-^{1}H HSQC NMR (400 MHz, $CDCl_3$)

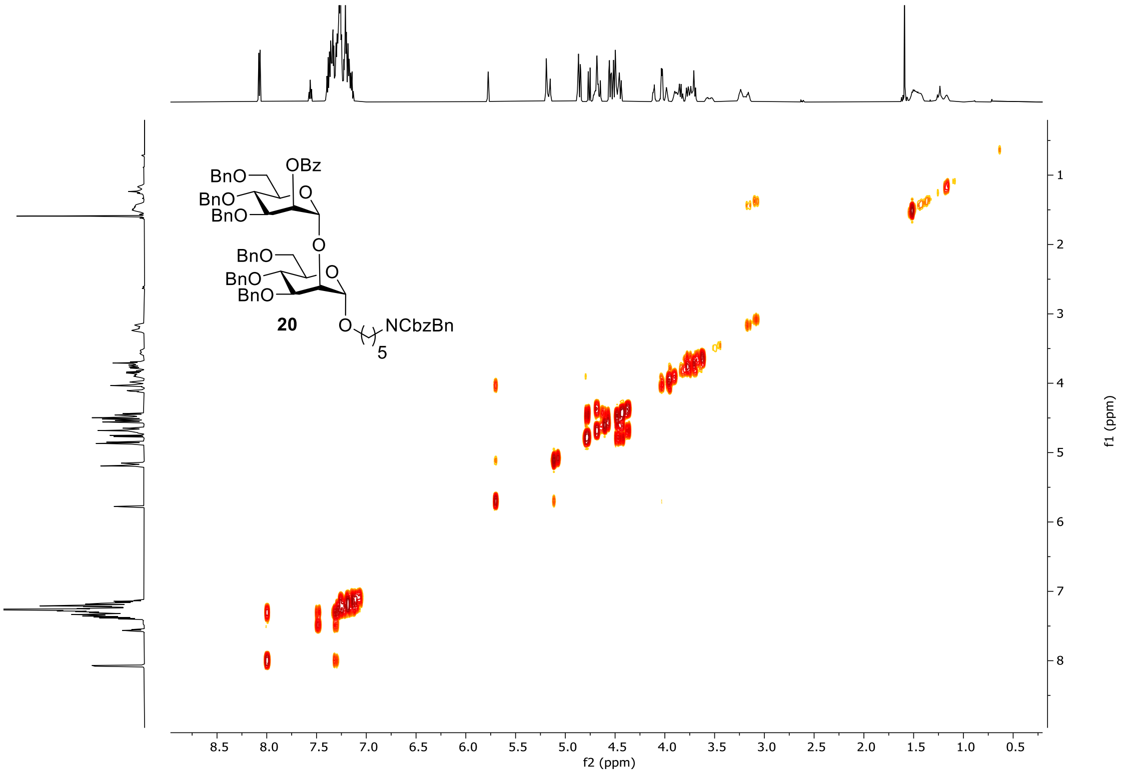

^{1}H-^{13}C HSQC NMR (400 MHz, $CDCl_3$)

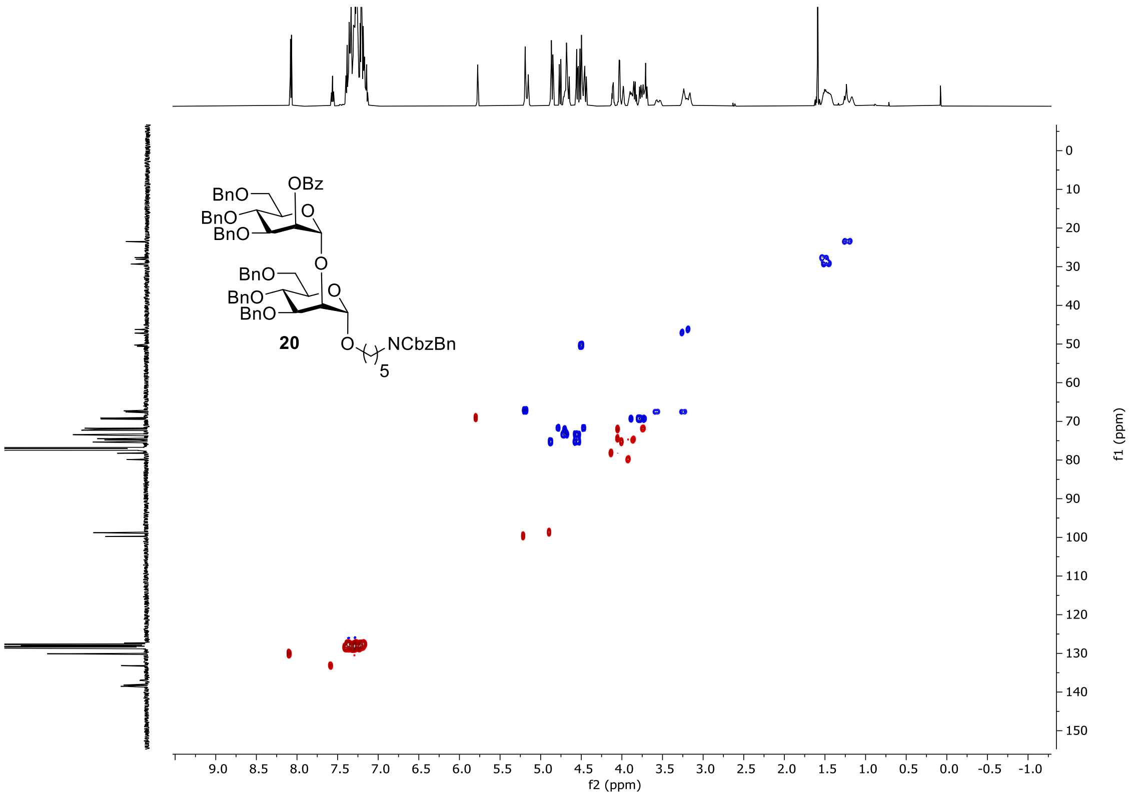

^{1}H-^{13}C Coupled HSQC NMR (400 MHz, $CDCl_3$)

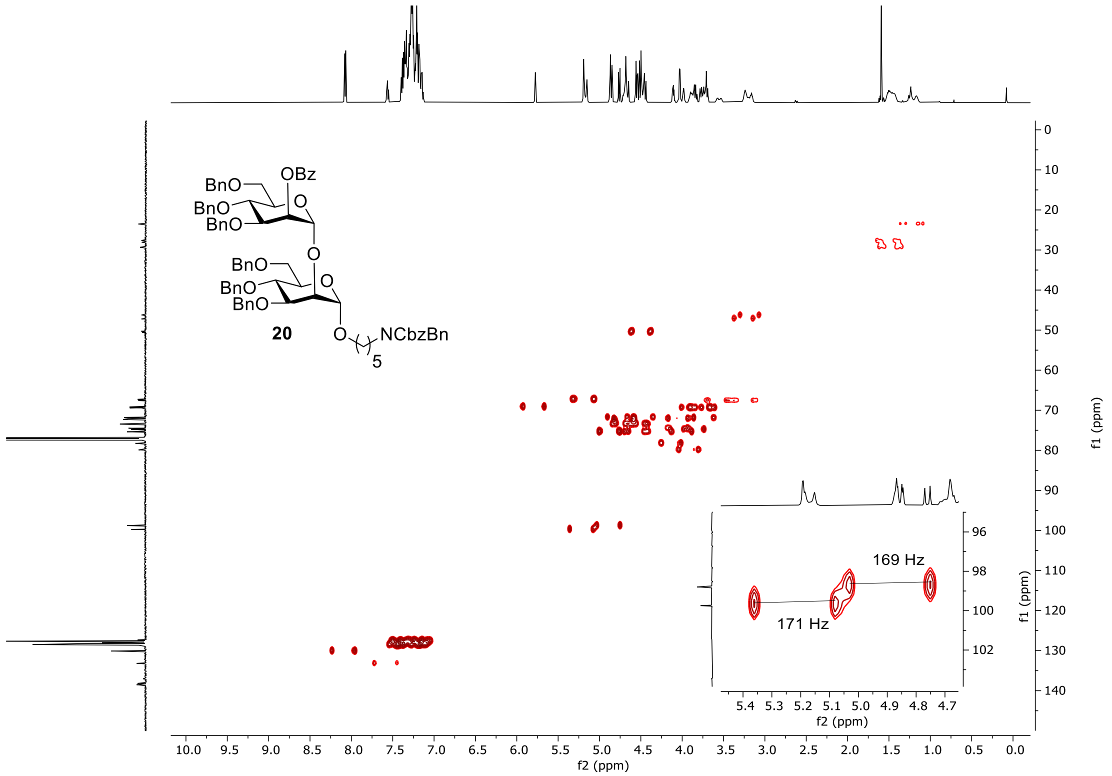

^{1}H NMR (400 MHz, $CDCl_3$)

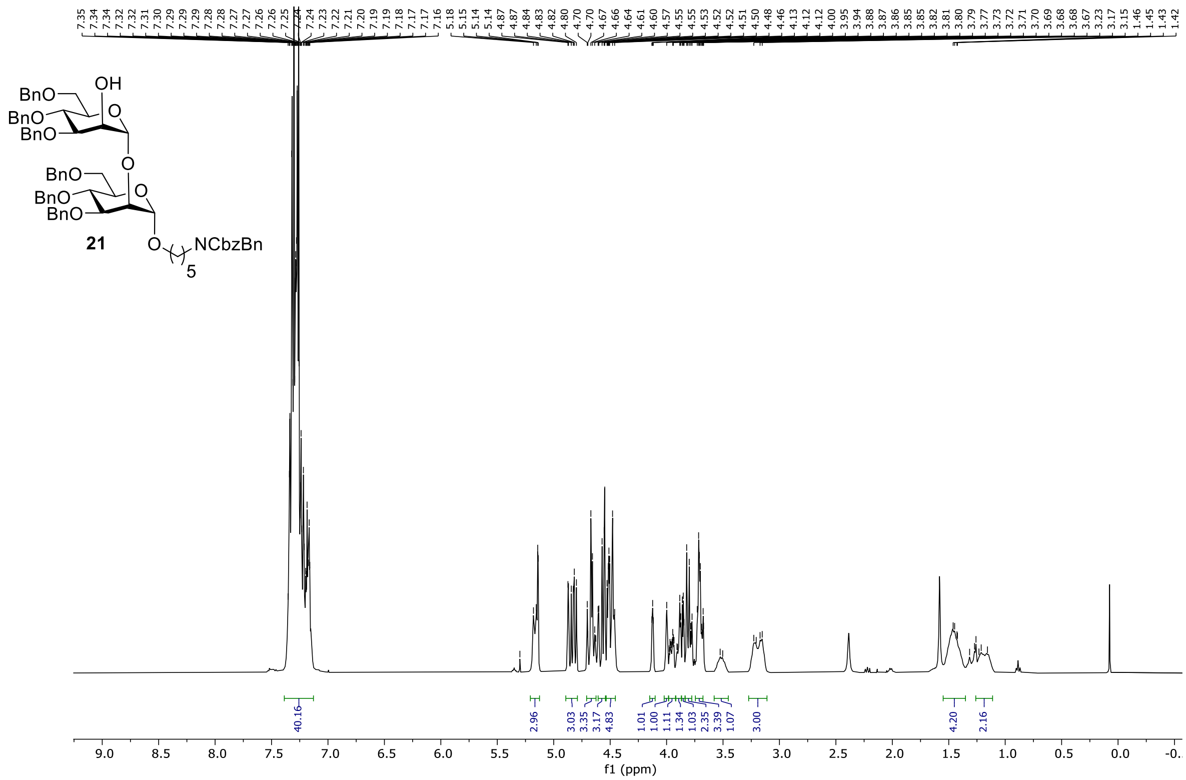

^{13}C NMR (101 MHz, $CDCl_3$)

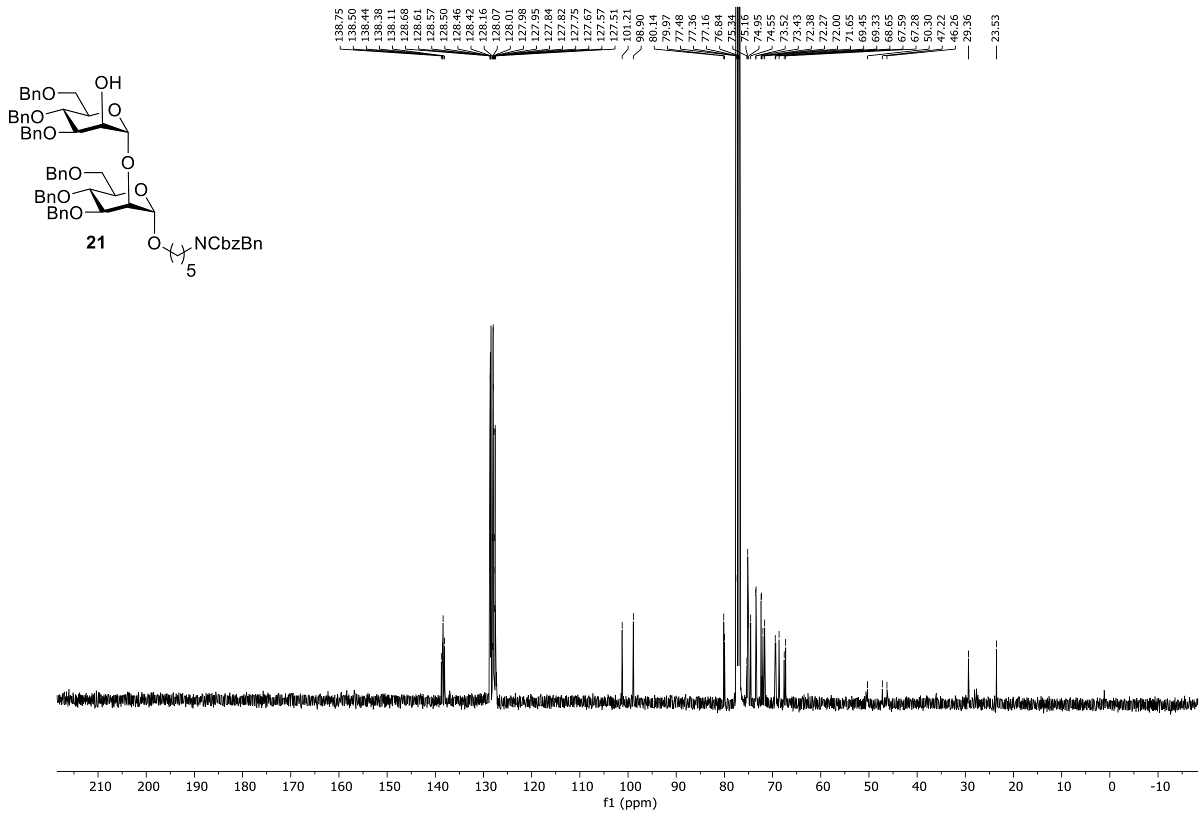

^{1}H-^{1}H COSY NMR (400 MHz, $CDCl_3$)

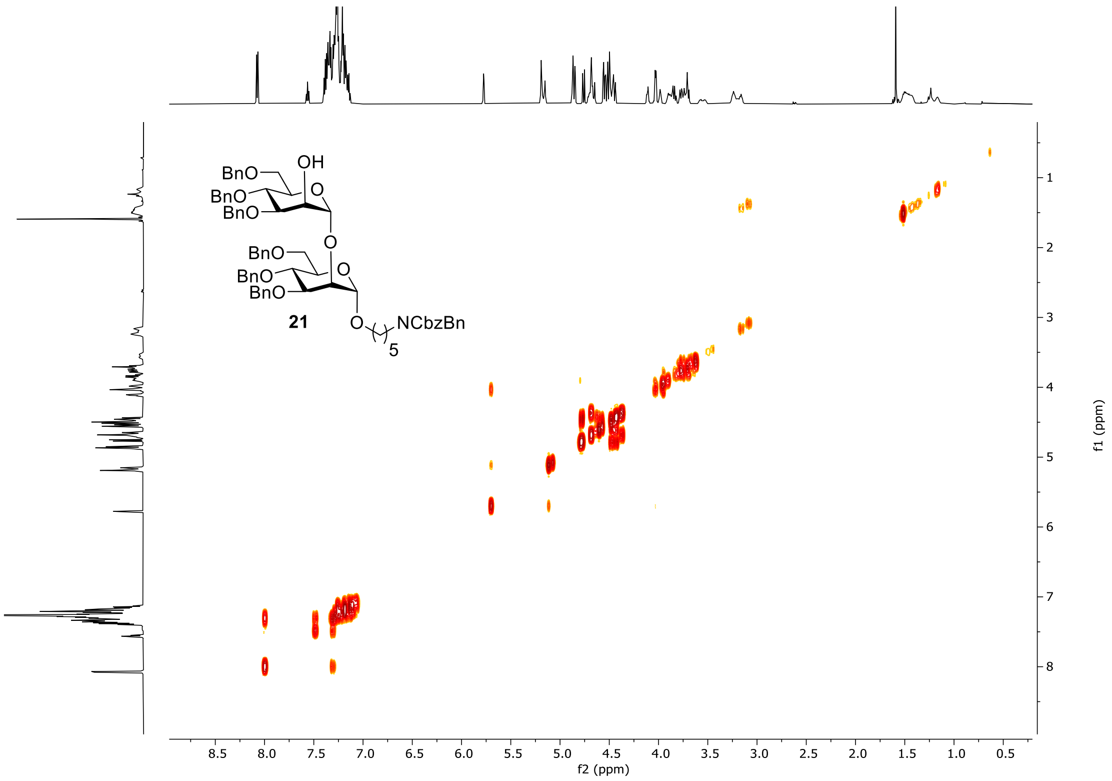

^{1}H-^{13}C HSQC NMR (400 MHz, $CDCl_3$)

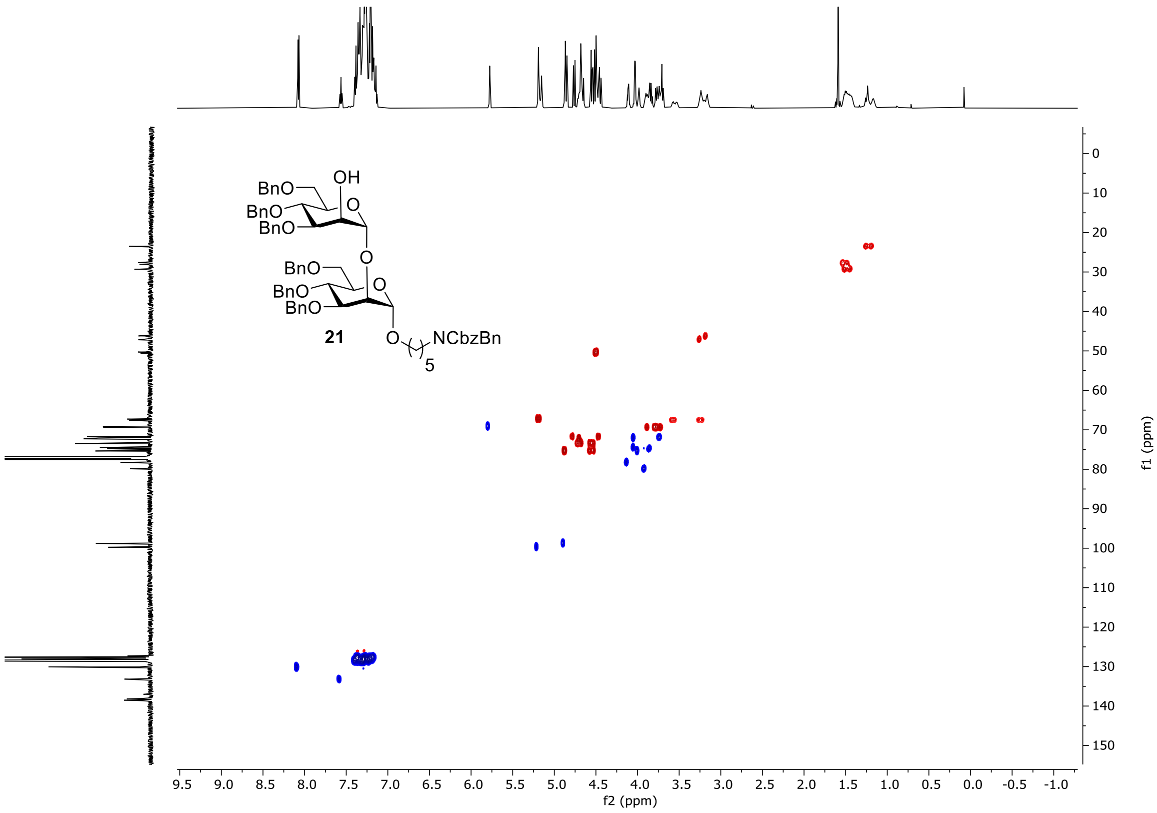

^{1}H NMR (600 MHz, $CDCl_3$)

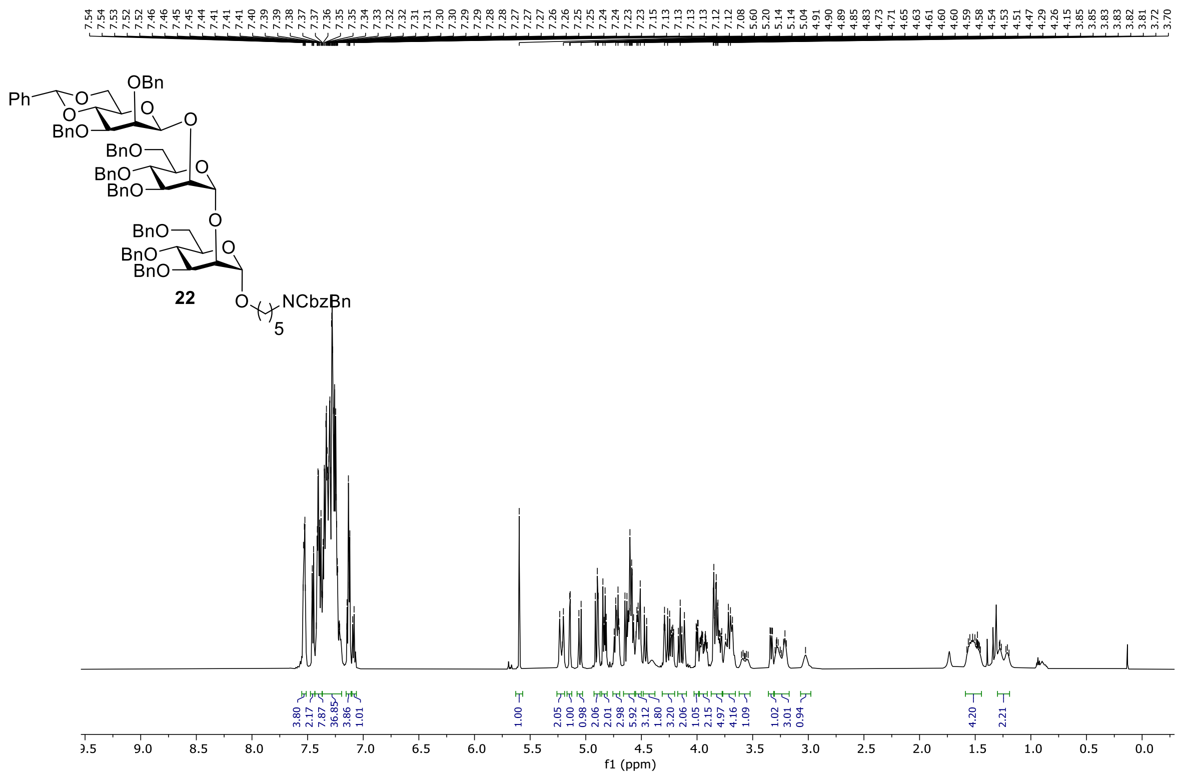

^{13}C NMR (151 MHz, $CDCl_3$)

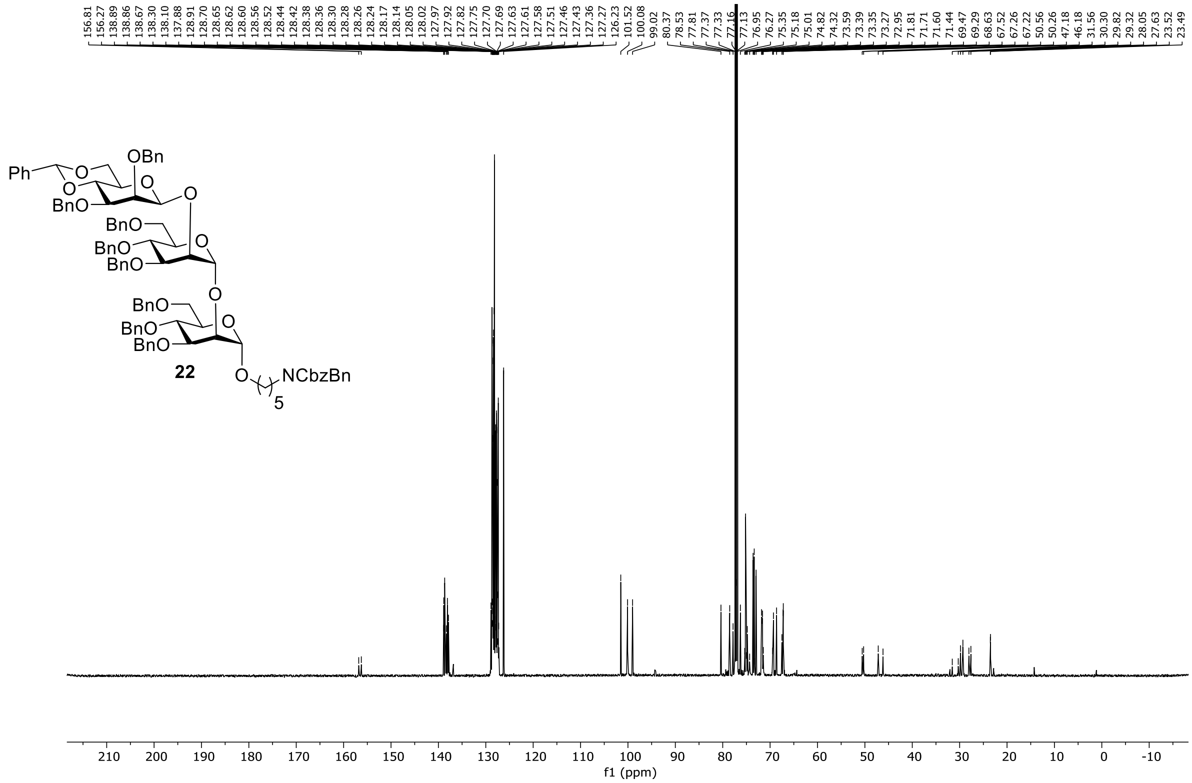

^{1}H-^{1}H COSY NMR (600 MHz, $CDCl_3$)

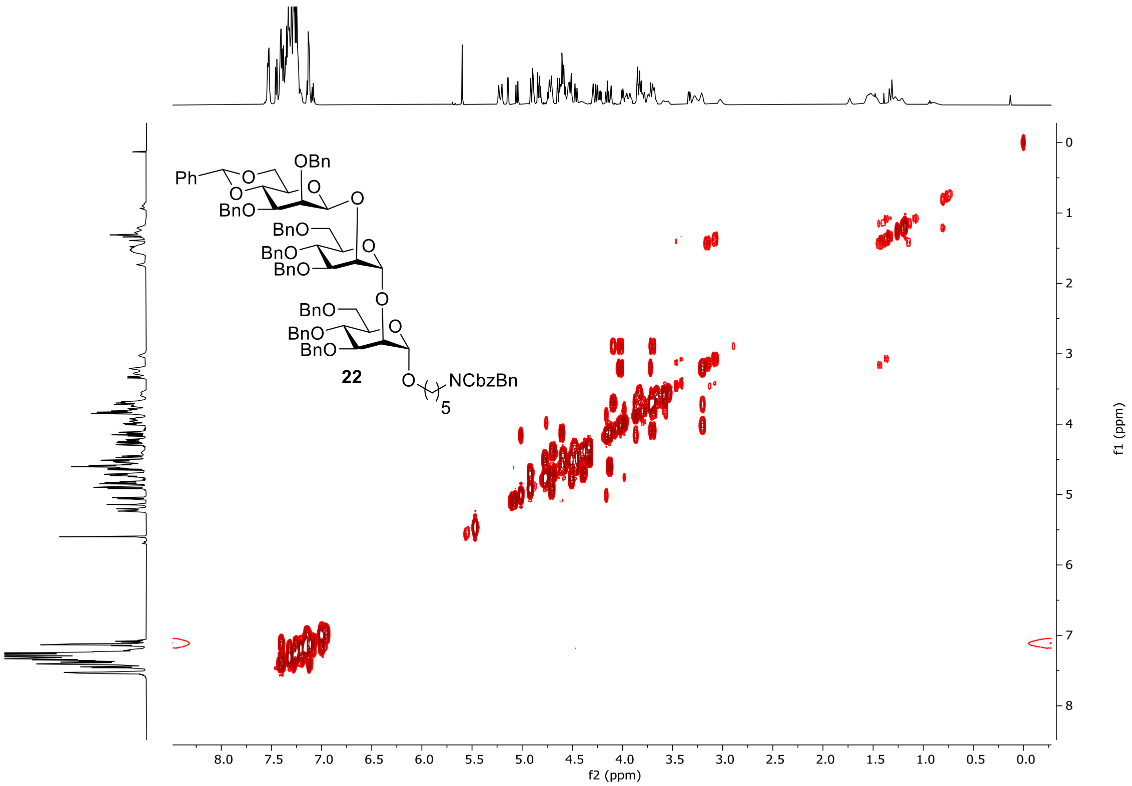

^{1}H-^{13}C HSQC NMR (600 MHz, $CDCl_3$)

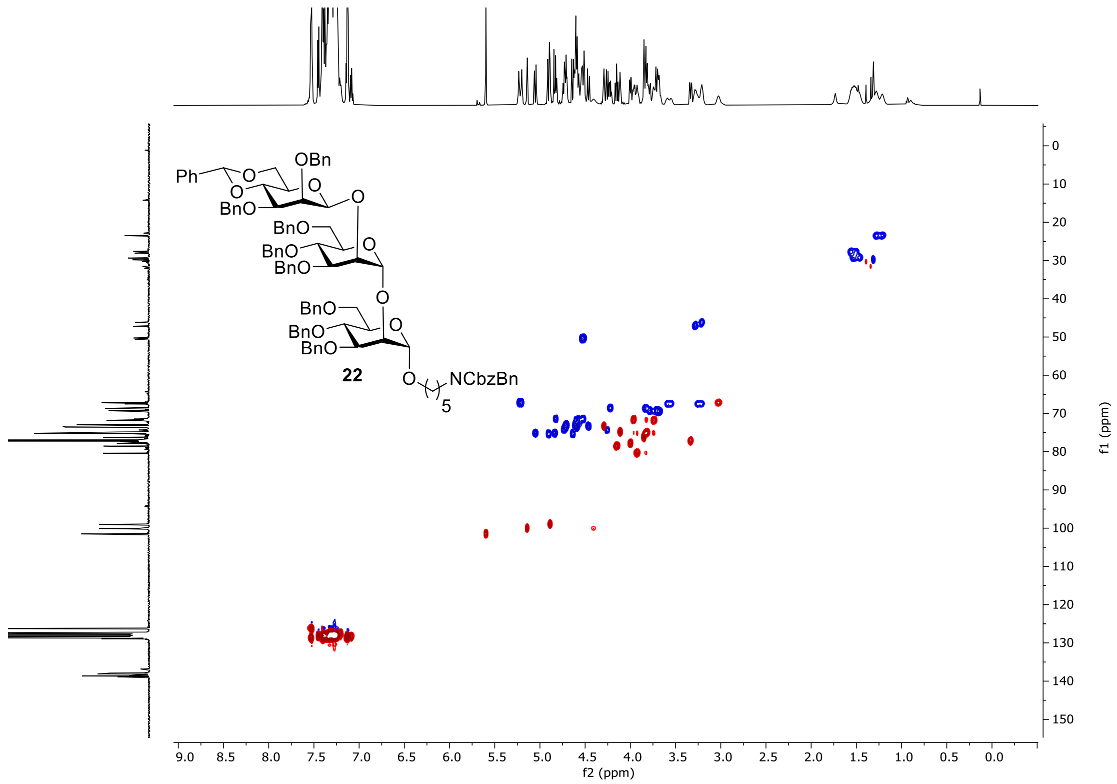

^{1}H-^{13}C HSQC NMR (600 MHz, $CDCl_3$)

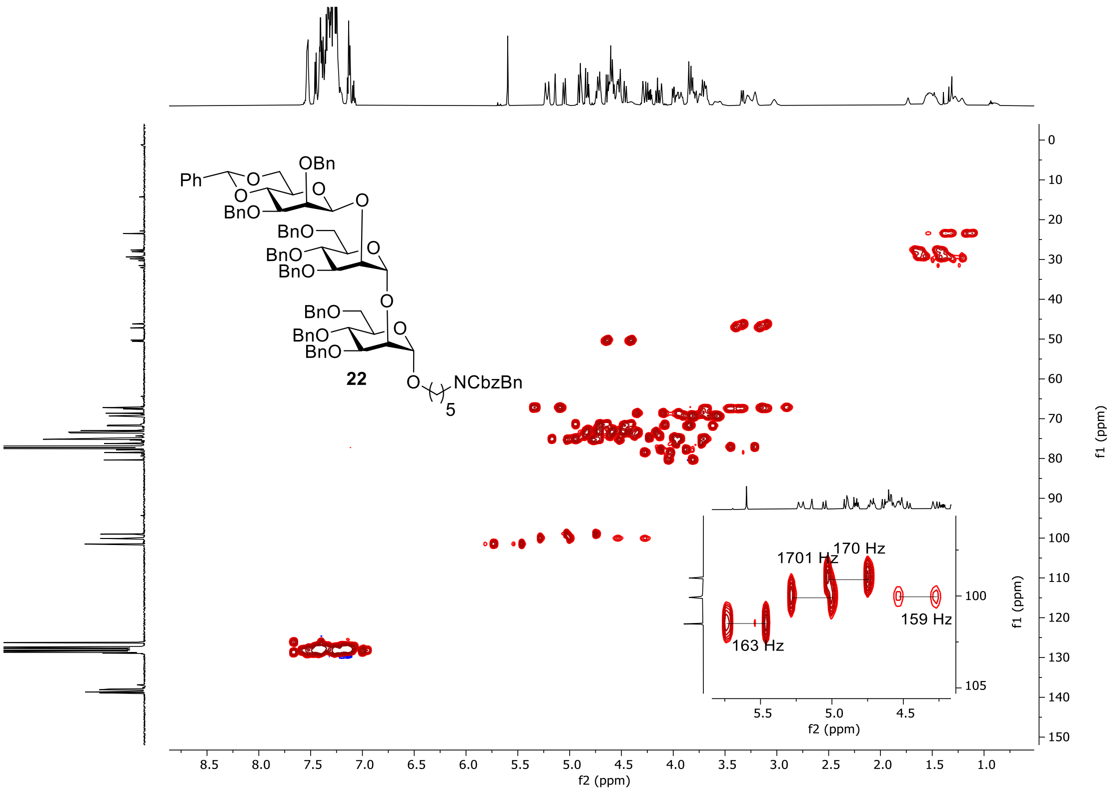

^{1}H NMR (700 MHz, D_2O)

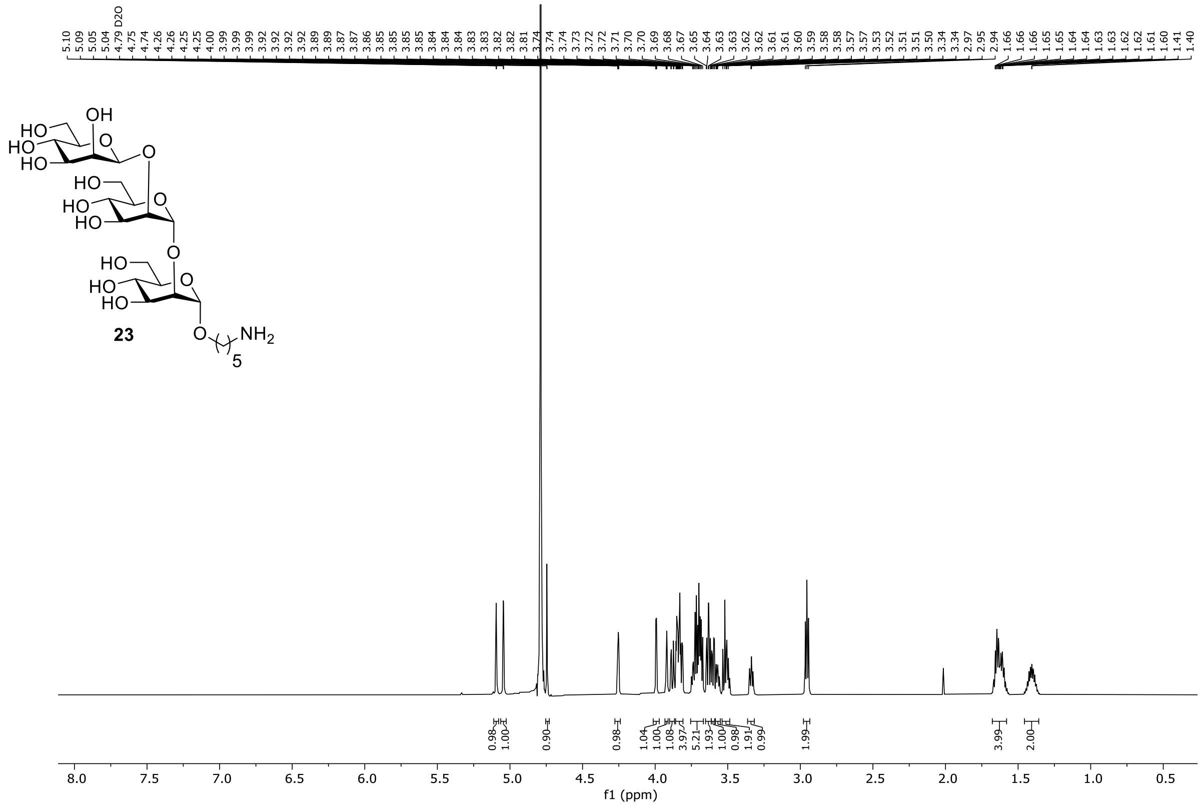

^{13}C NMR (700 MHz, D_2O)

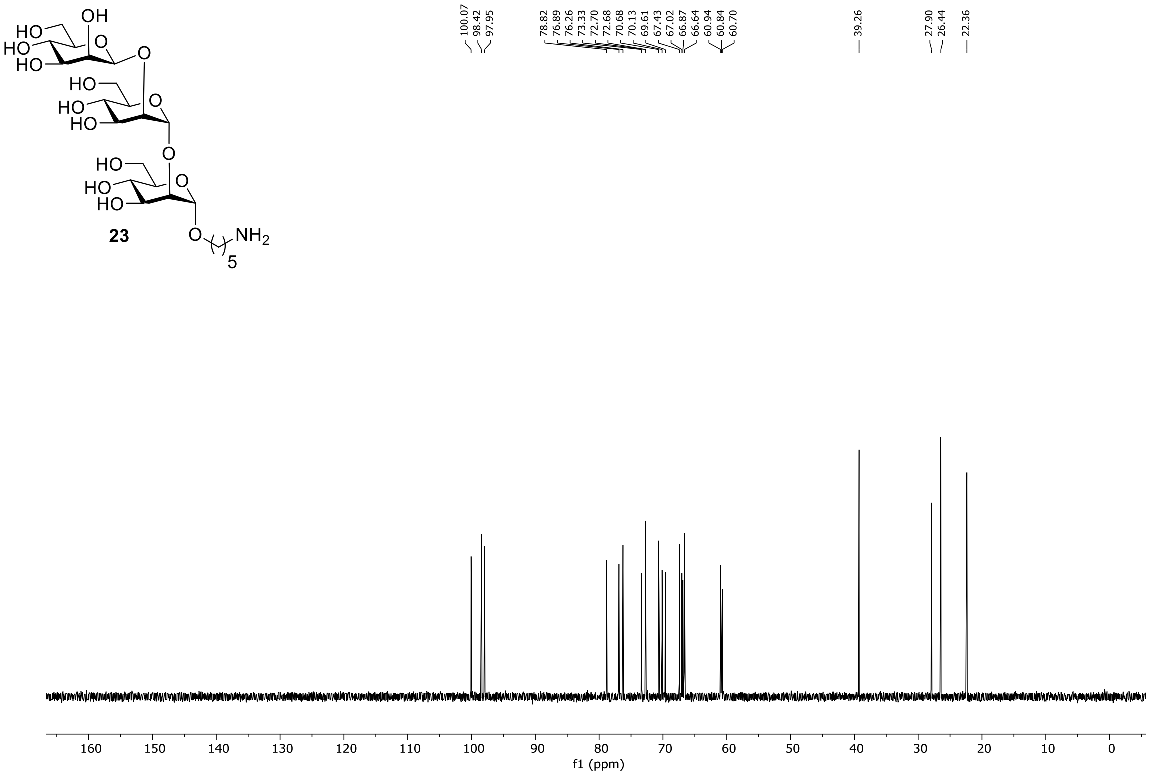

^1H-^1H COSY NMR (700 MHz, D_2O)

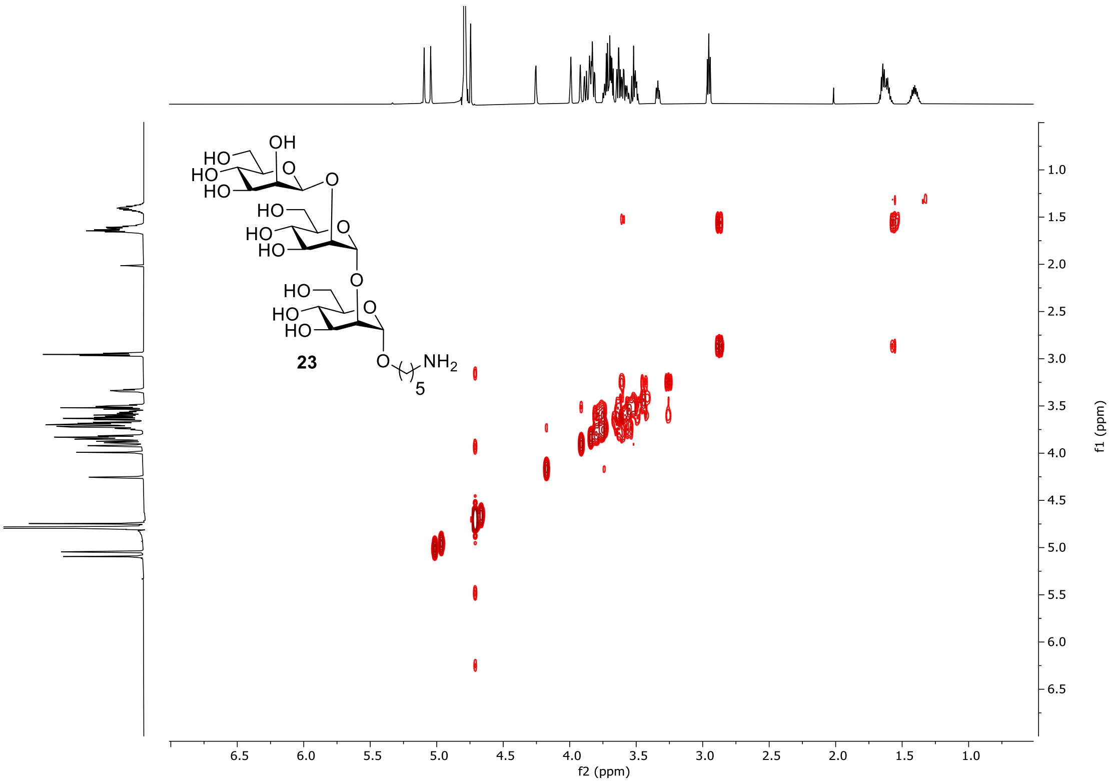

^{1}H-^{13}C HSQC NMR (700 MHz, D_2O)

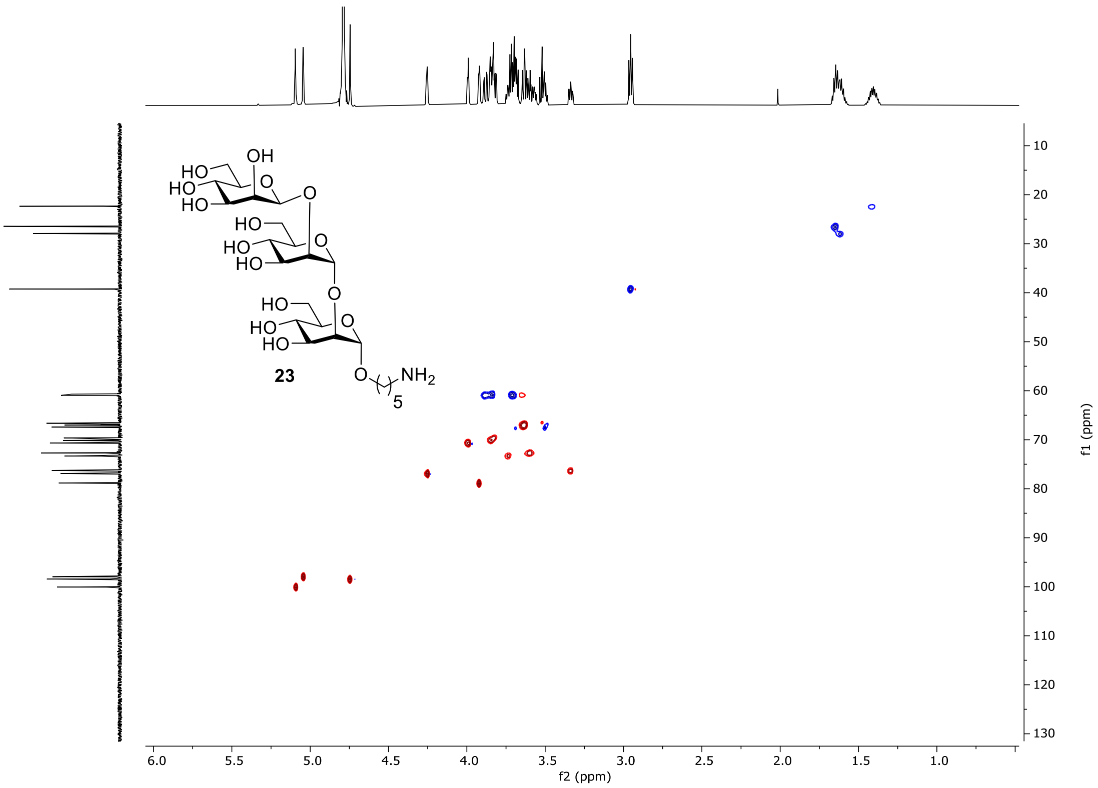

^{1}H-^{13}C Coupled HSQC NMR (700 MHz, D_2O)

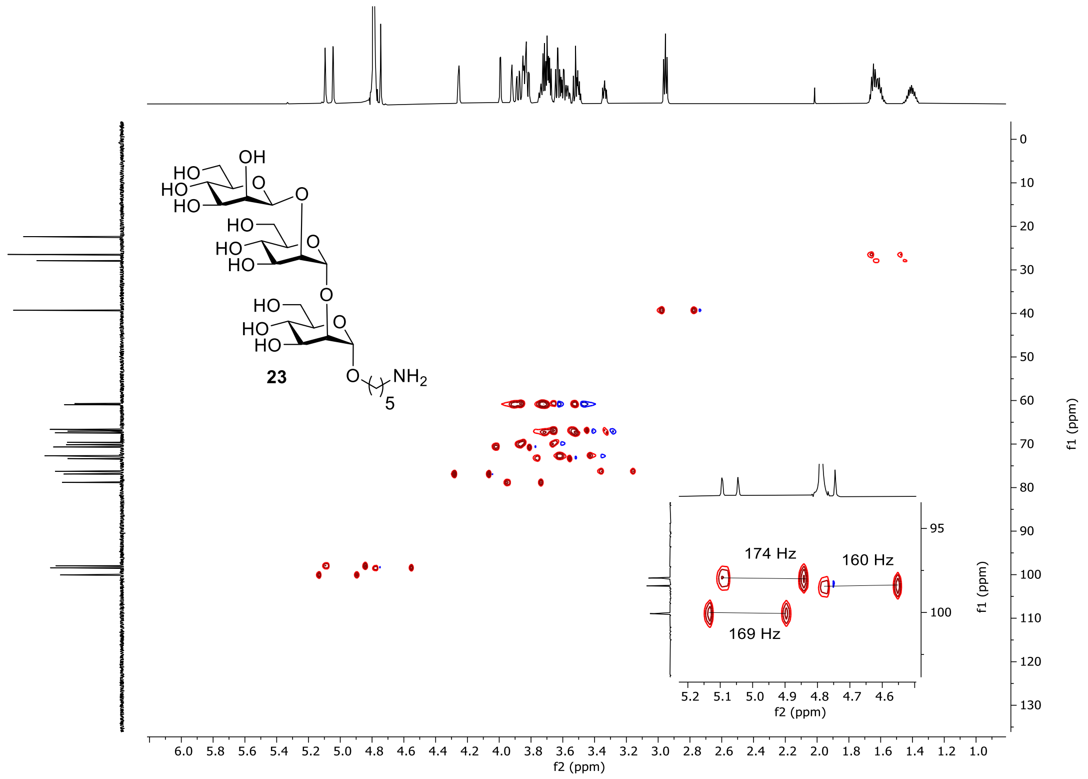

^{1}H NMR (600 MHz, D_2O)

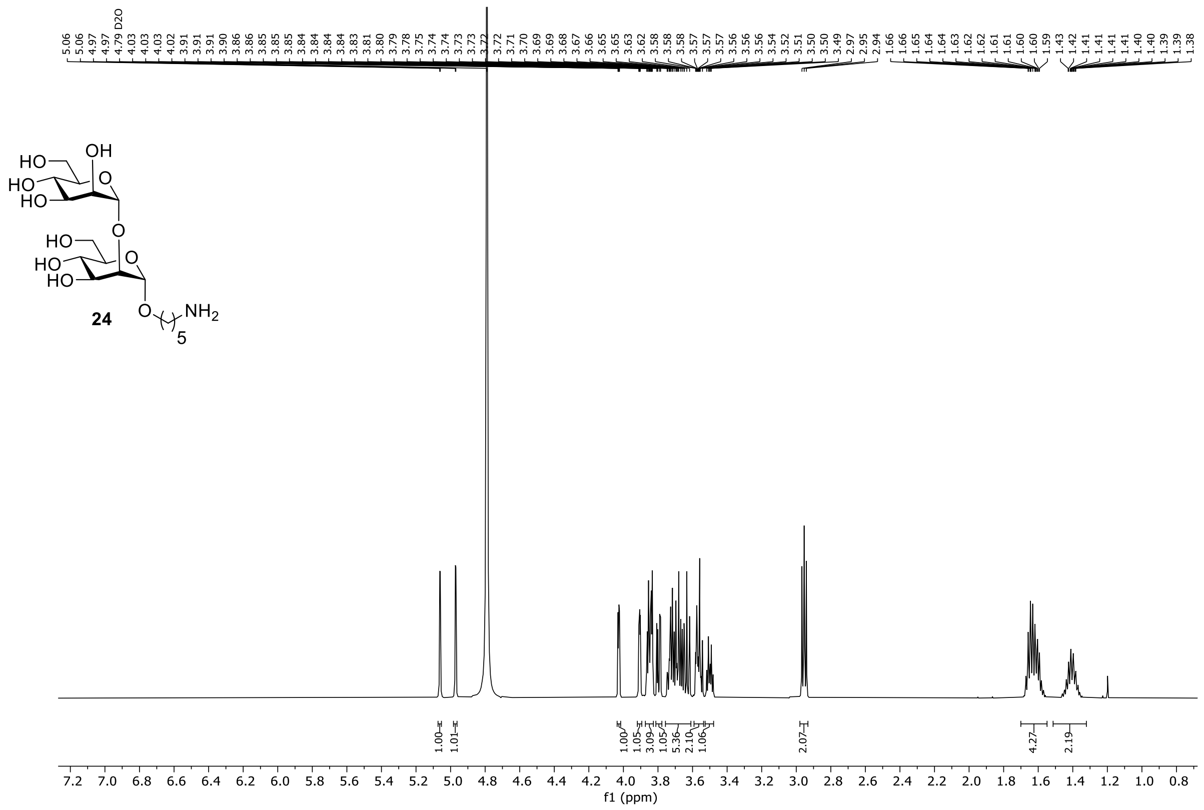

^{13}C NMR (151 MHz, D_2O)

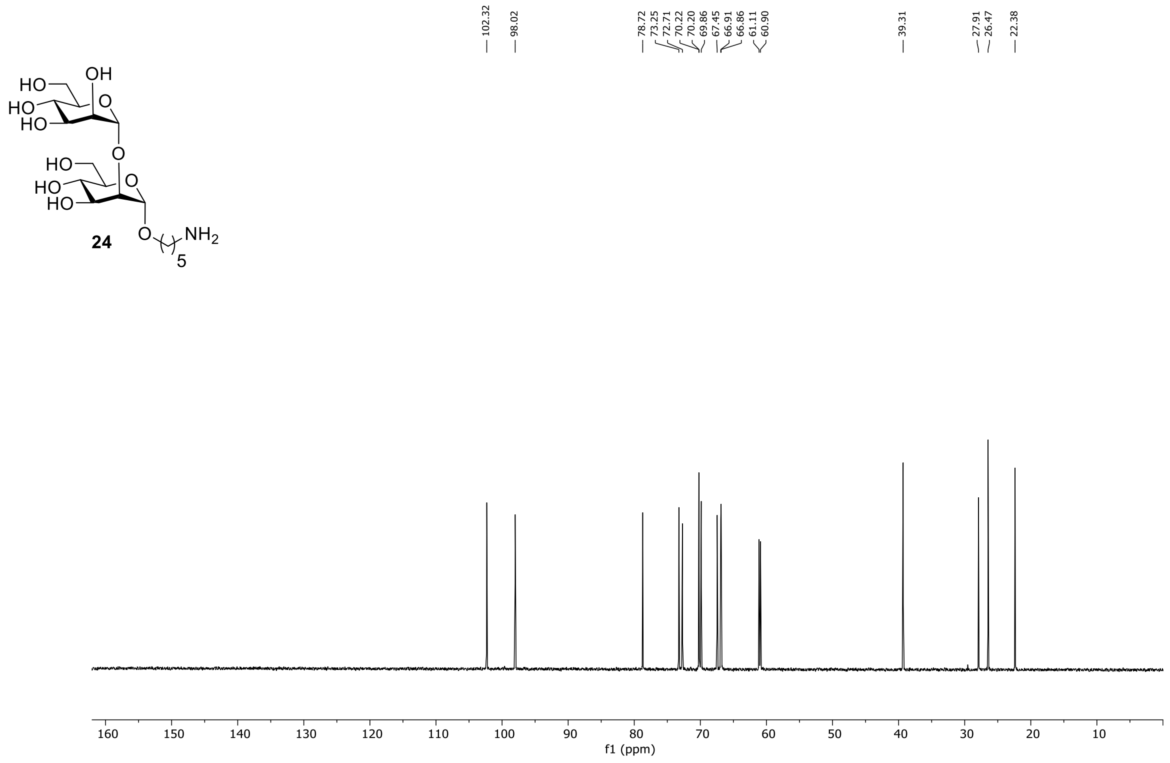

^{1}H-^{1}H COSY NMR (600 MHz, D_2O)

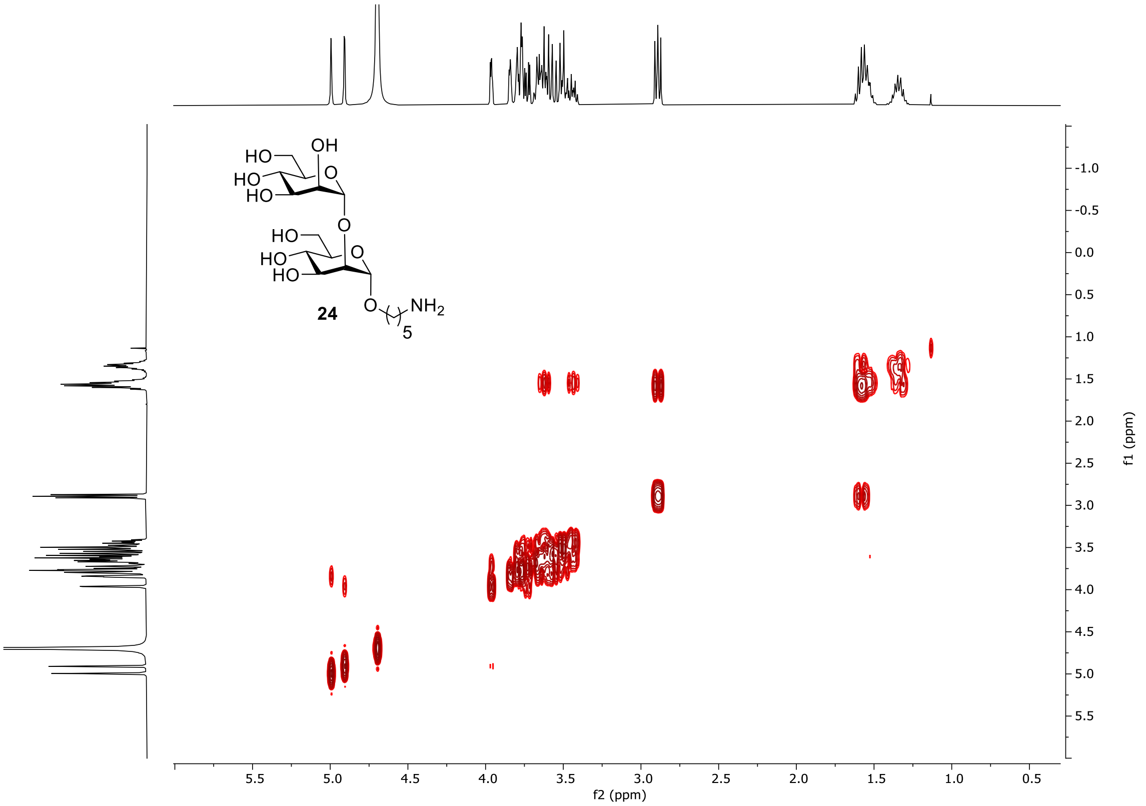

^{1}H-^{13}C HSQC NMR (600 MHz, D_2O)

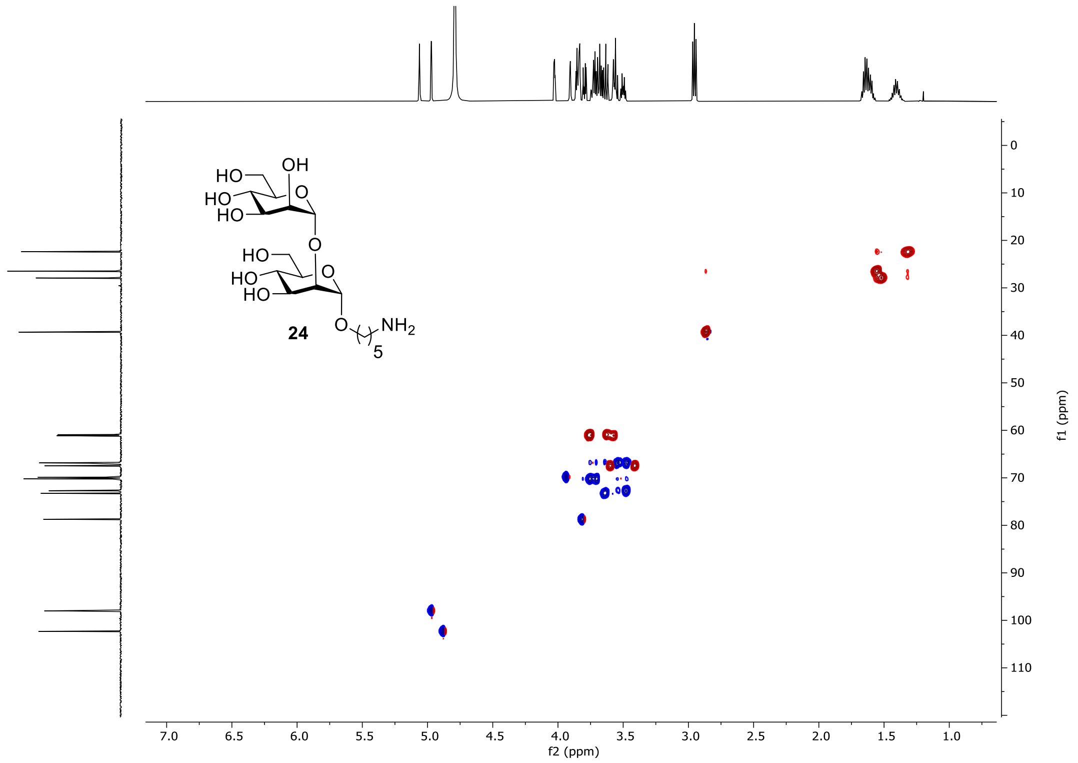

^{1}H-^{13}C Coupled HSQC NMR (600 MHz, D_2O)

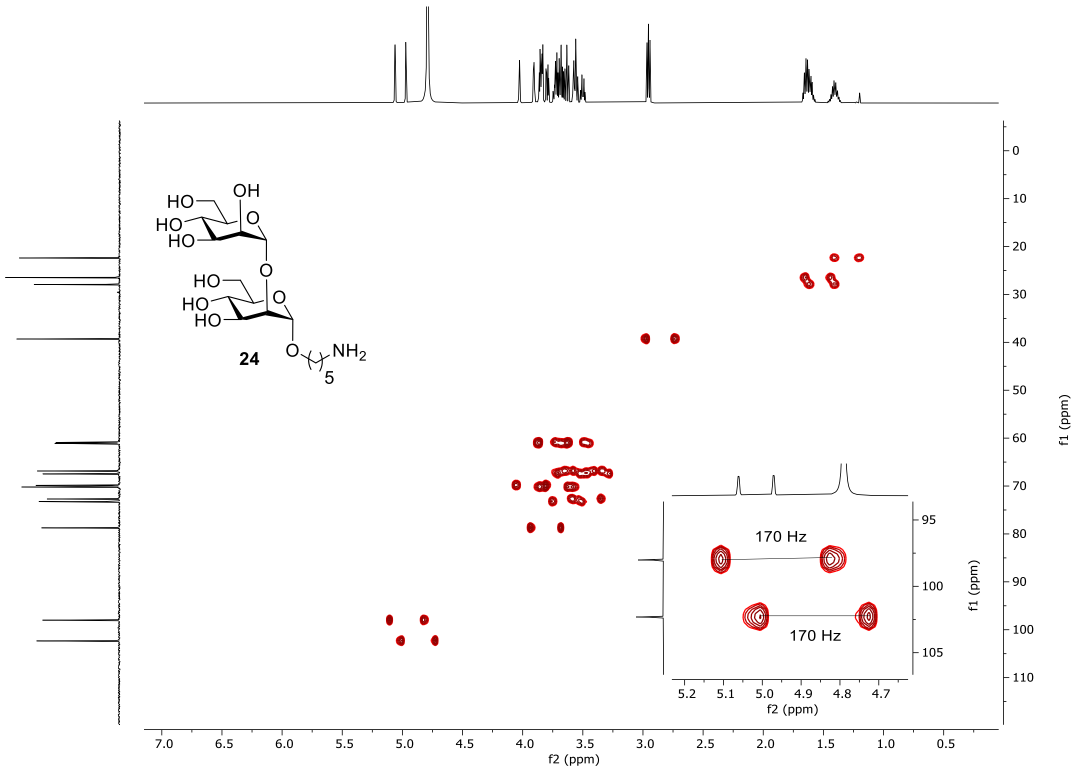

^{1}H NMR (400 MHz, $CDCl_3$)

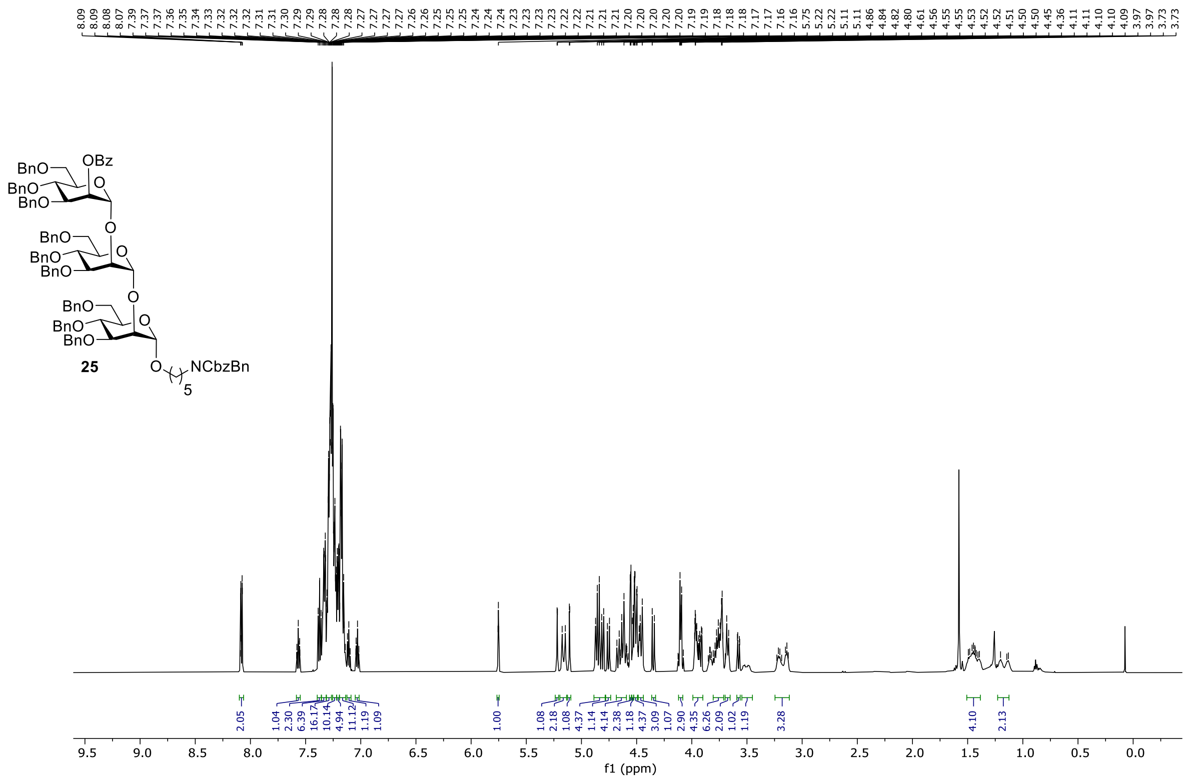

^{13}C NMR (101 MHz, $CDCl_3$)

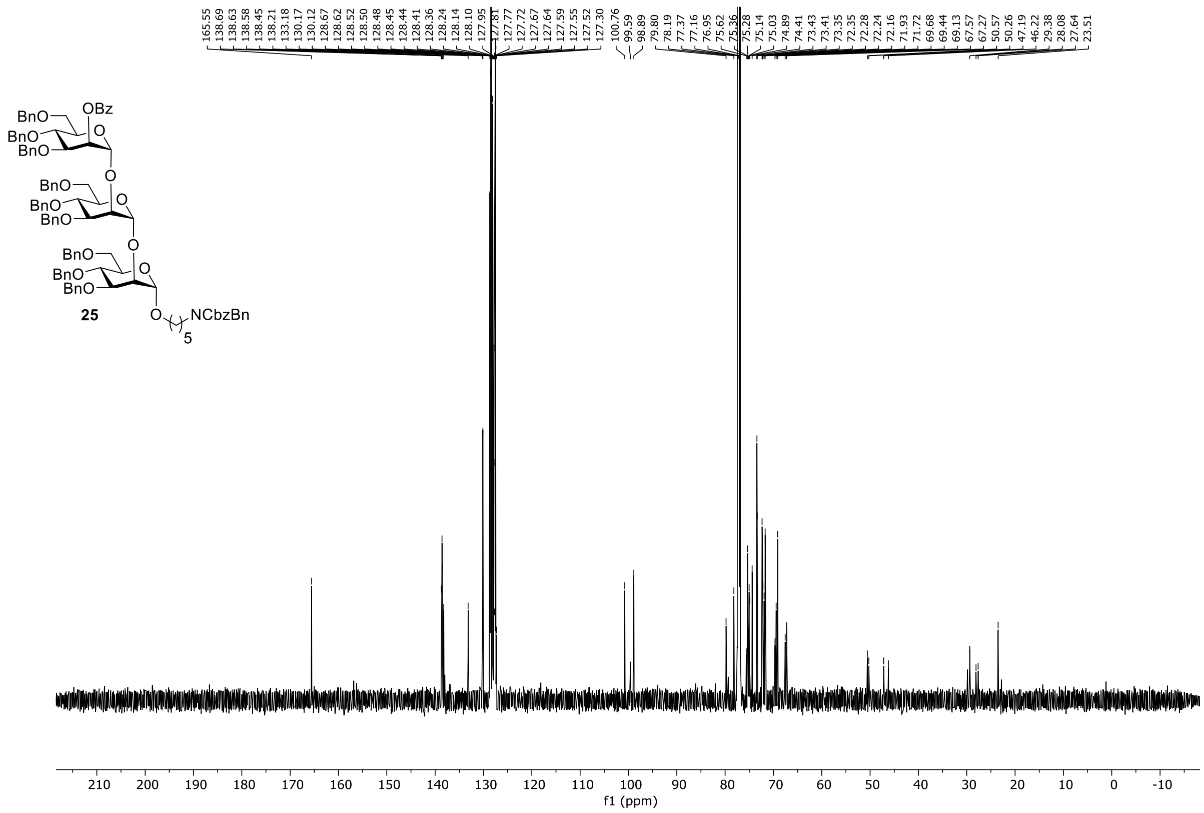

^1H-^1H COSY NMR (400 MHz, $CDCl_3$)

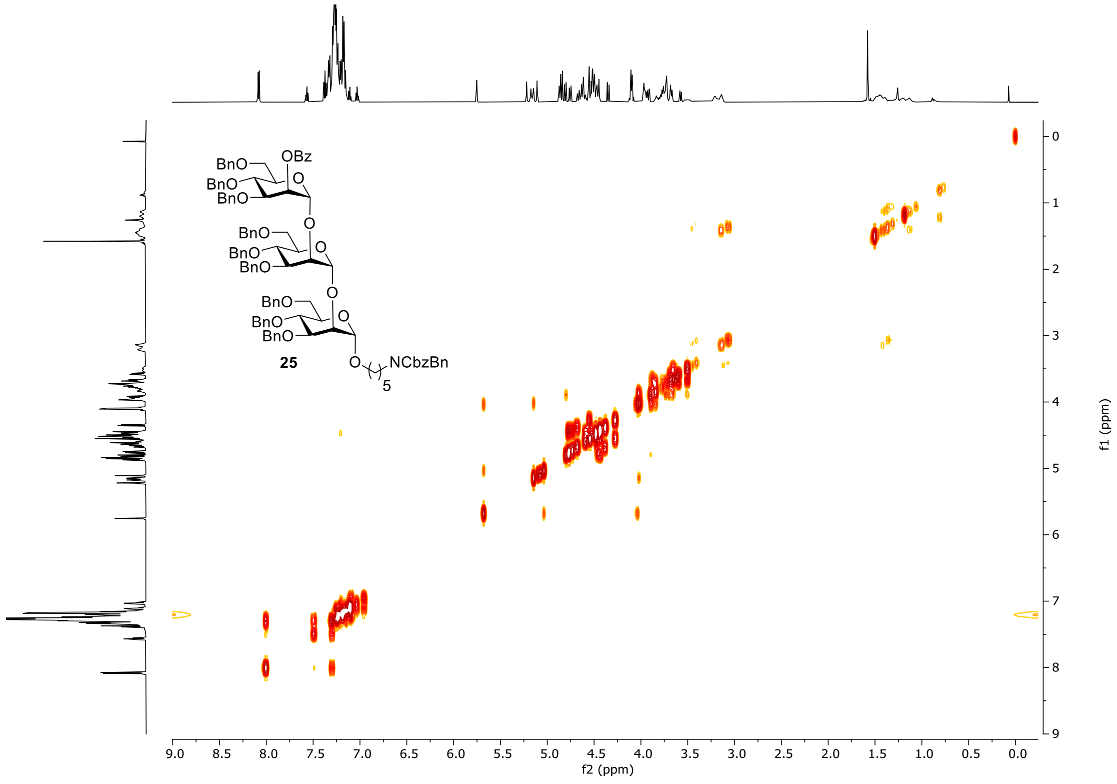

^{1}H-^{13}C NMR (400 MHz, $CDCl_3$)

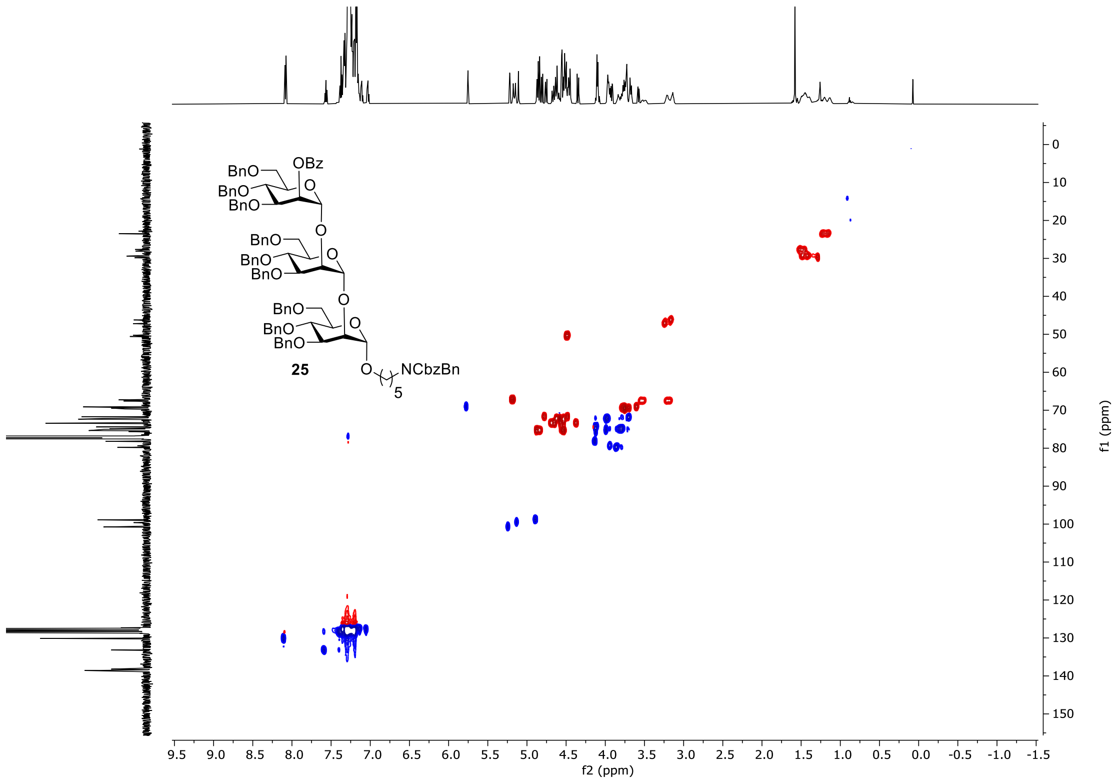

^{1}H-^{13}C Coupled HSQC NMR (400 MHz, $CDCl_3$)

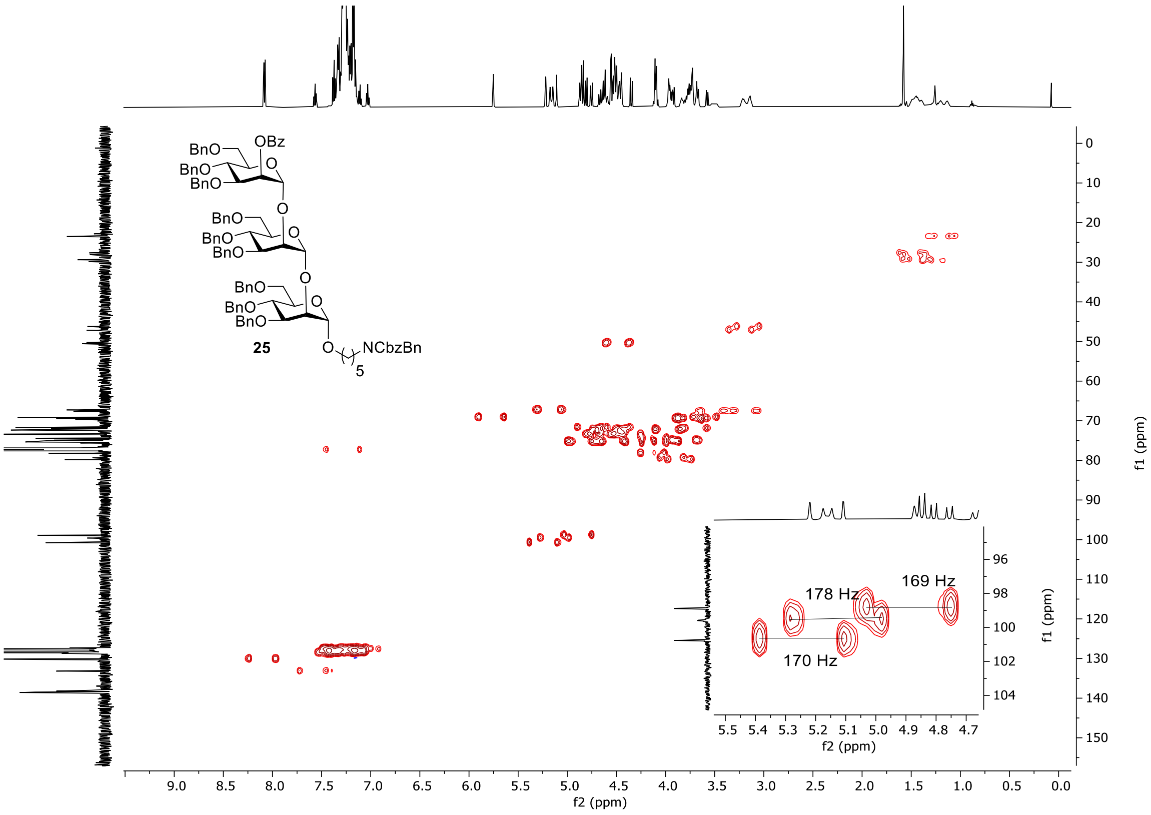

^{1}H NMR (400 MHz, $CDCl_3$)

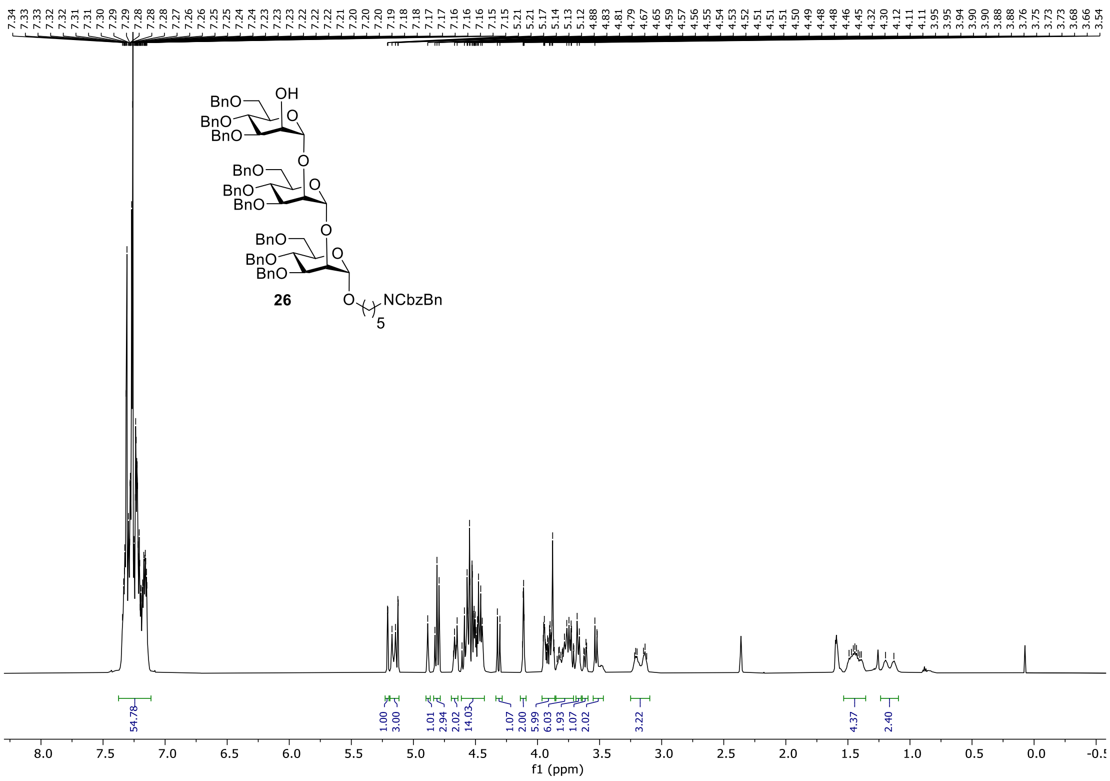

^{13}C NMR (101 MHz, $CDCl_3$)

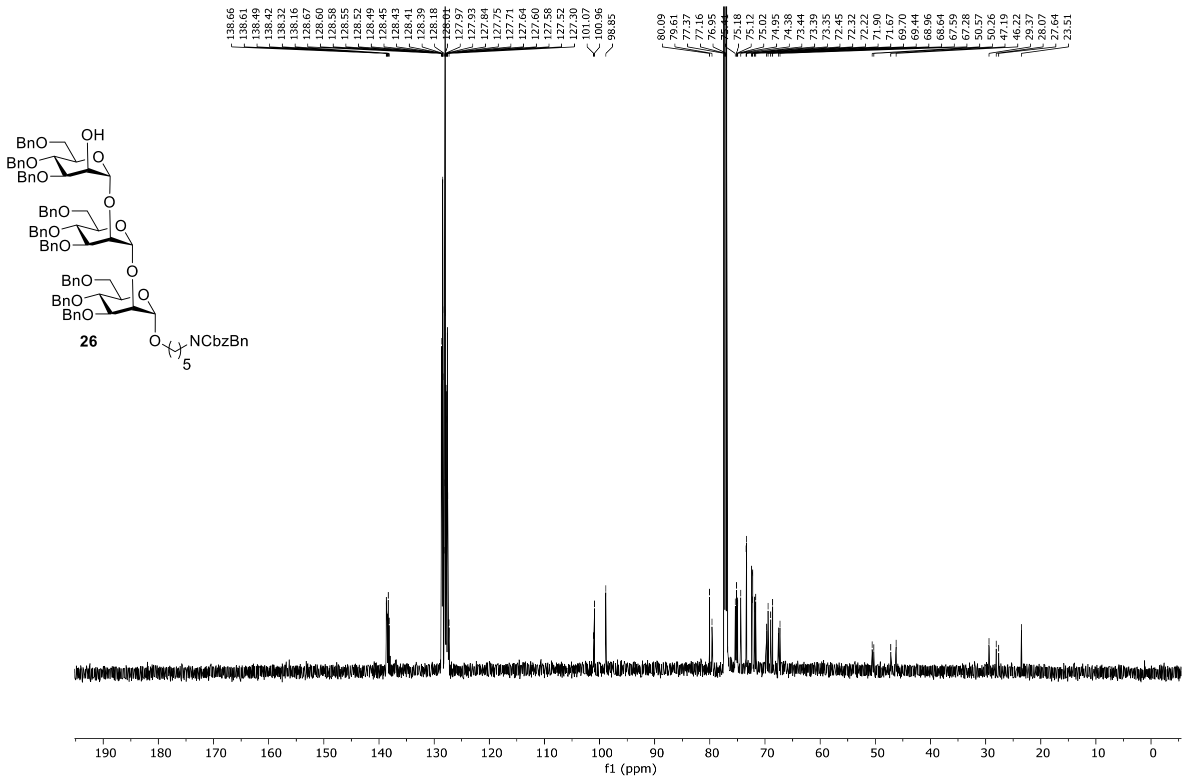

^{1}H-^{1}H COSY HSQC NMR (400 MHz, $CDCl_3$)

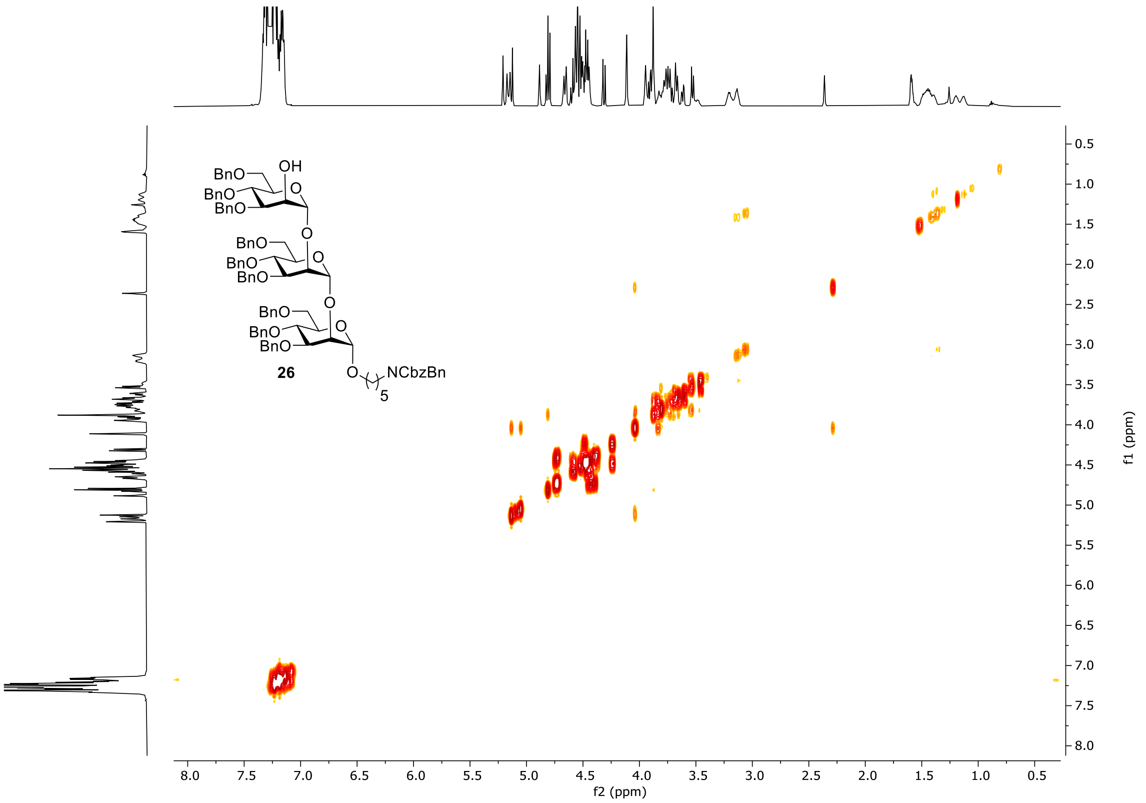

^{1}H-^{13}C HSQC NMR (400 MHz, $CDCl_3$)

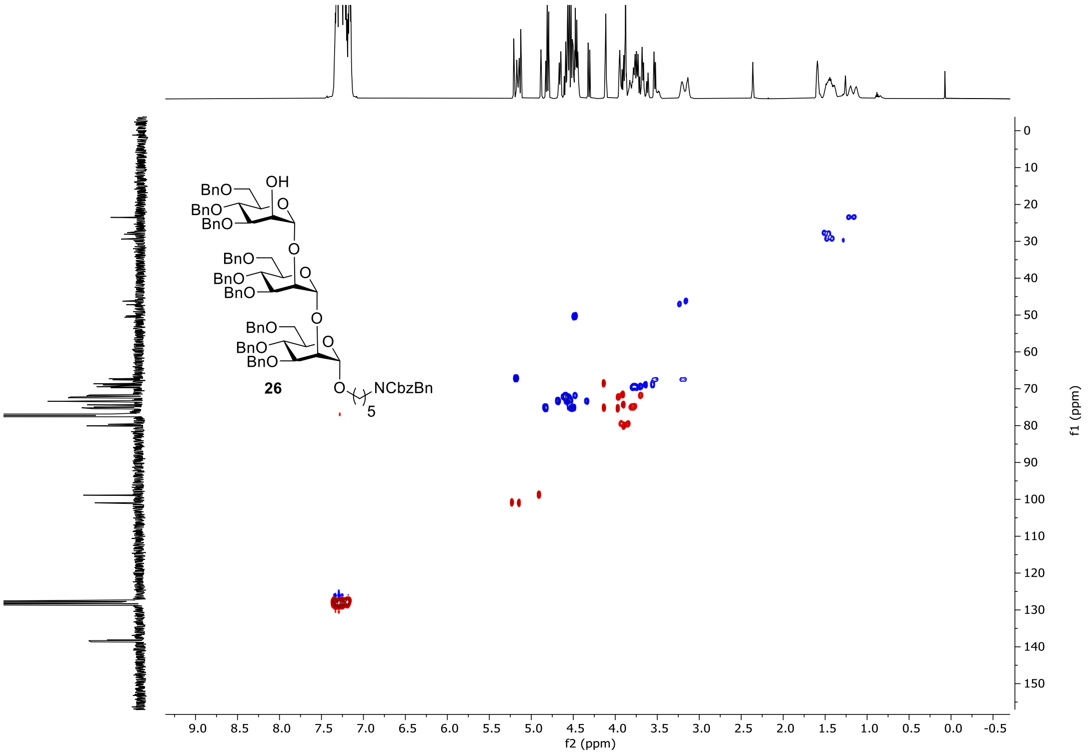

^{1}H-^{13}C Coupled HSQC NMR (400 MHz, $CDCl_3$)

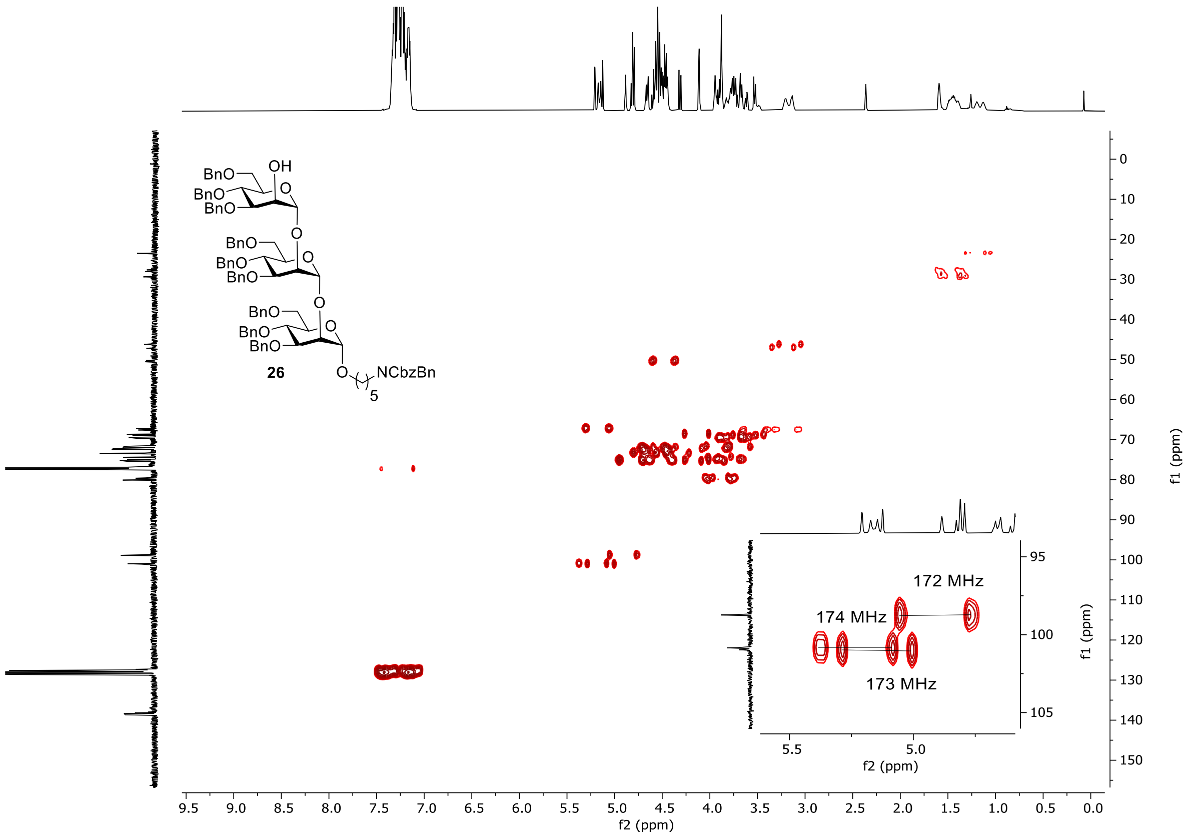

^{1}H NMR (600 MHz, $CDCl_3$)

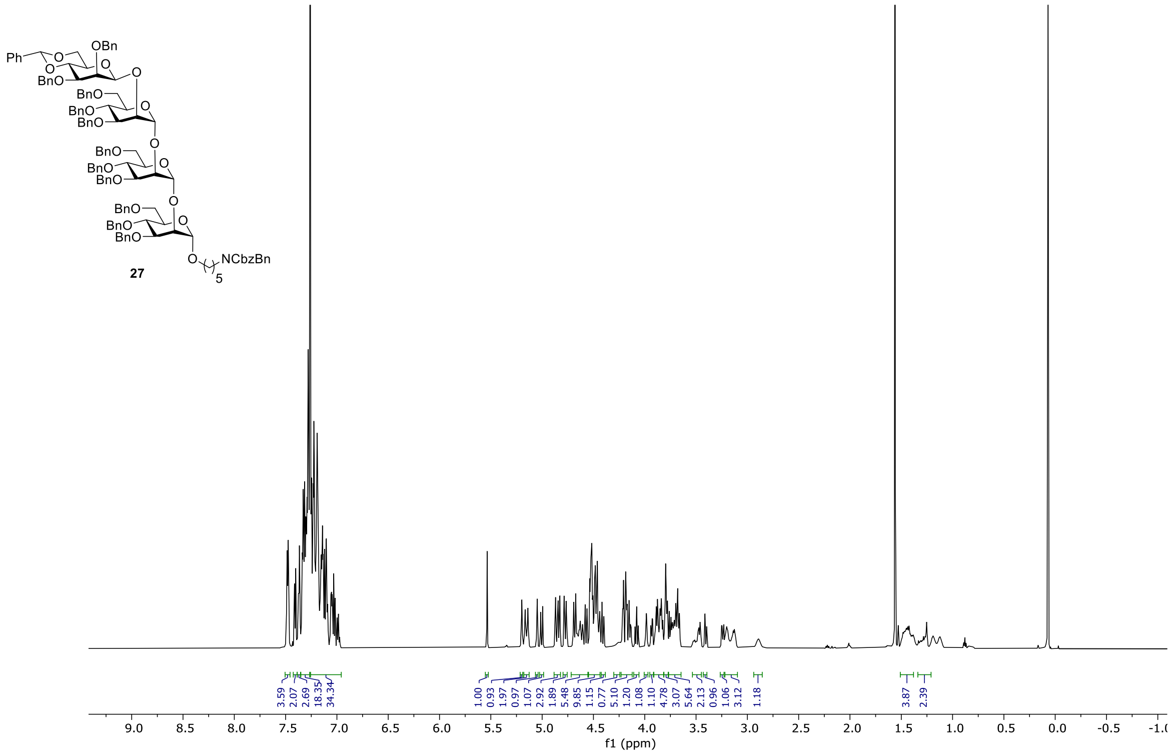

^{13}C NMR (600 MHz, CDCl$_3$)

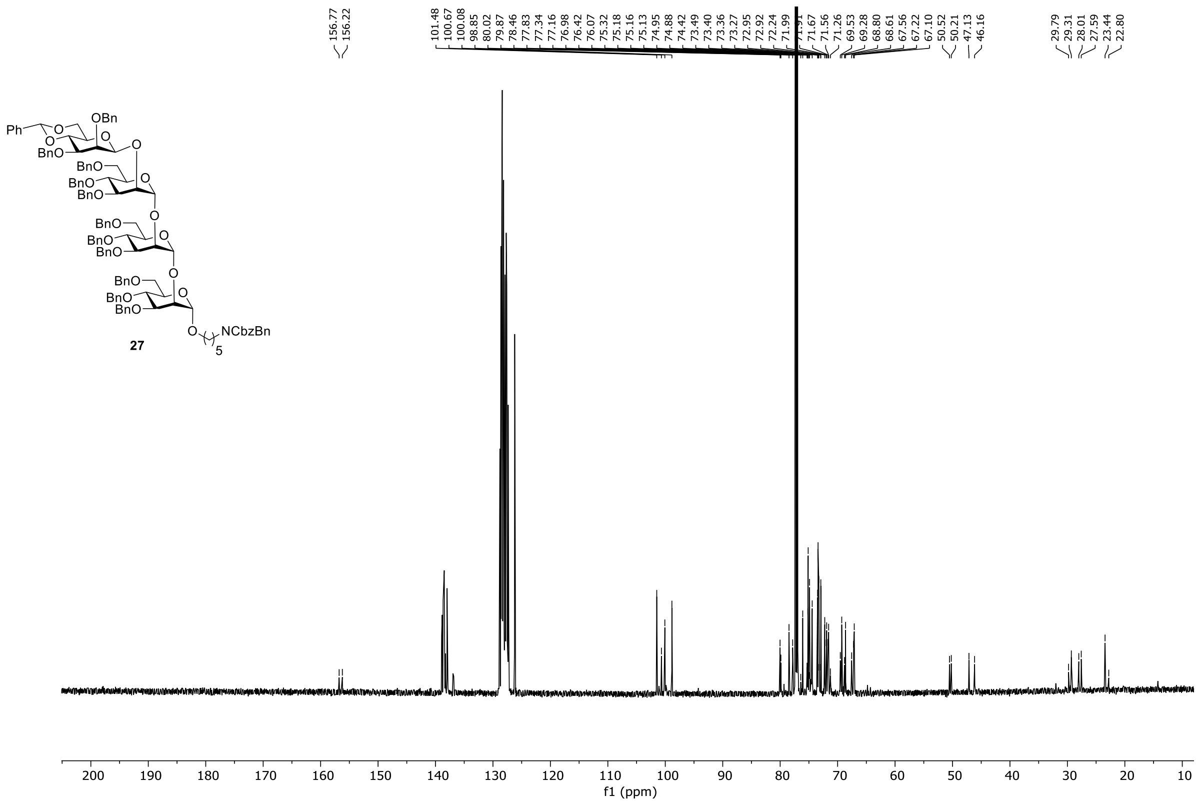

^{1}H-^{1}H COSY NMR (600 MHz, CDCl$_3$)

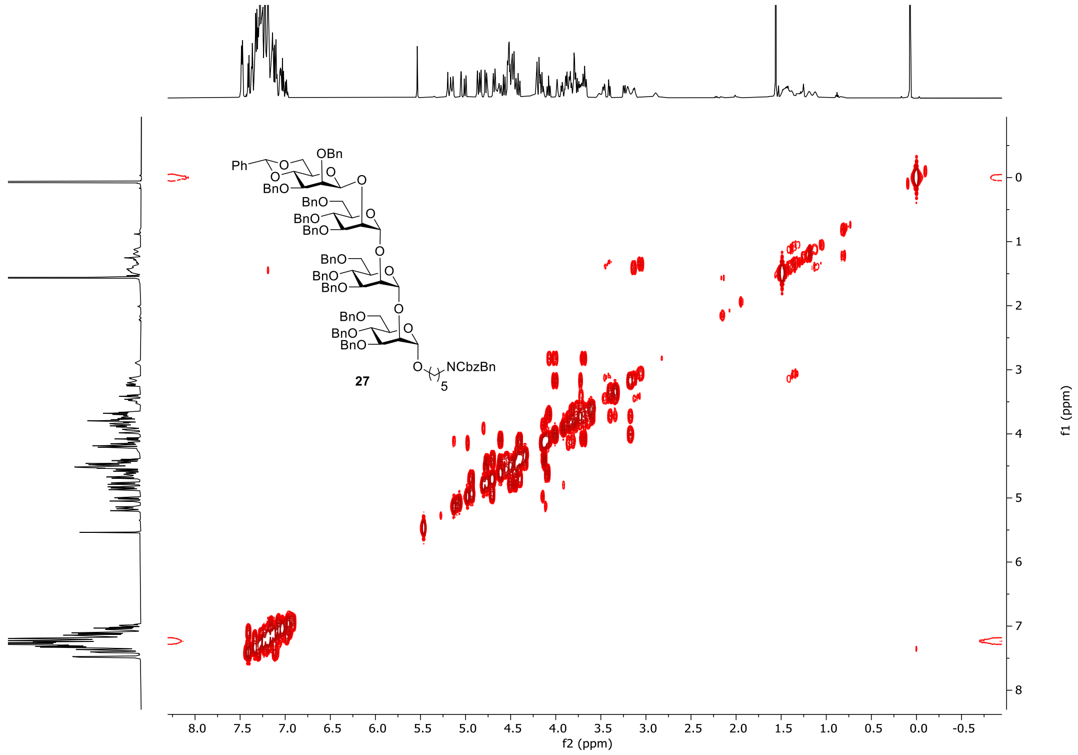

^{1}H-^{13}C HSQC NMR (600 MHz, $CDCl_3$)

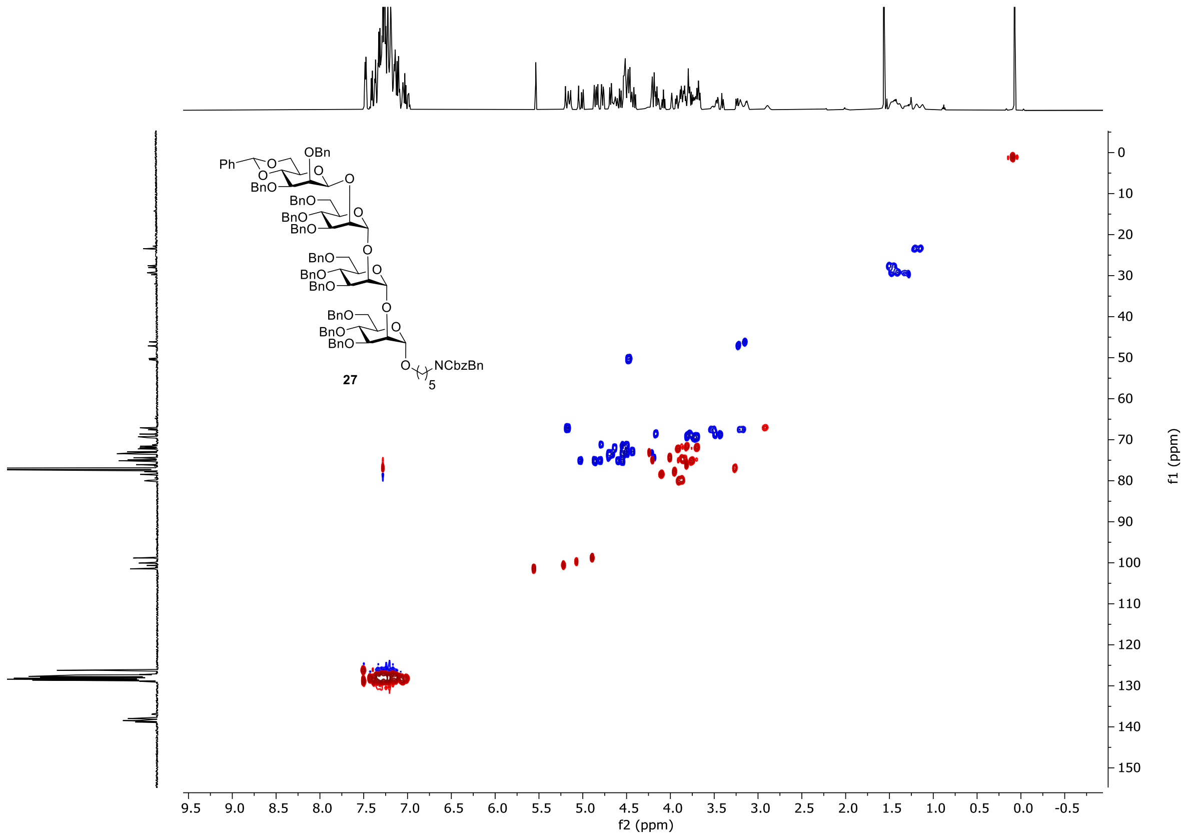

^{1}H-^{13}C Coupled HSQC NMR (600 MHz, $CDCl_3$)

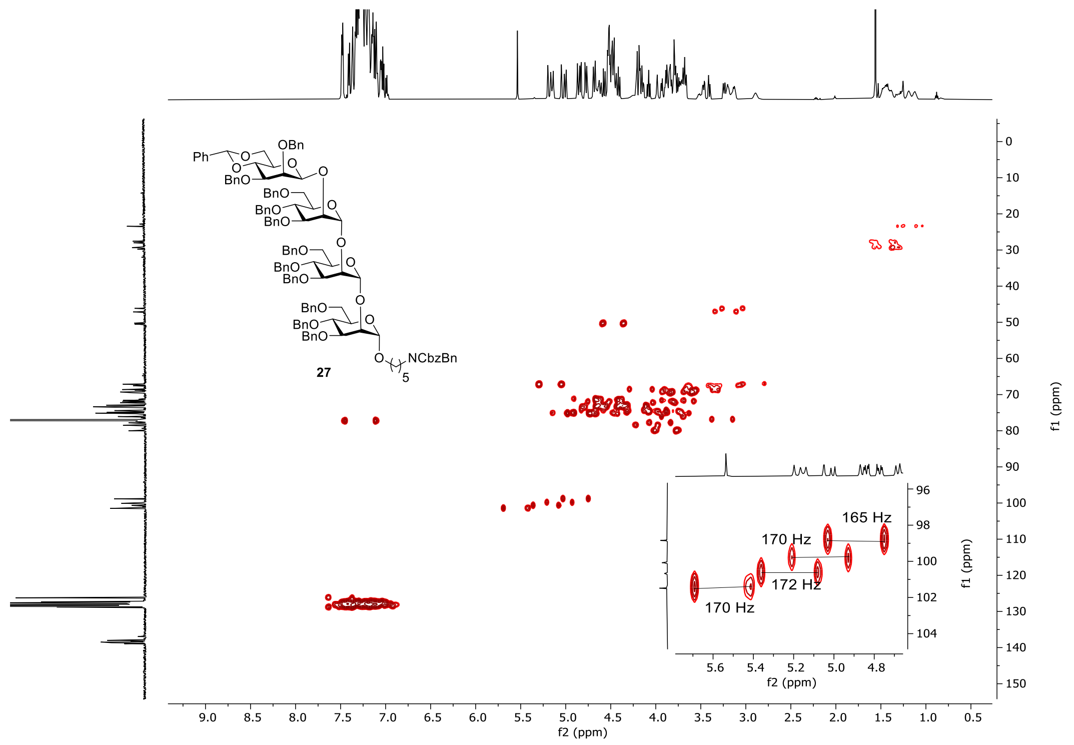

^{1}H NMR (700 MHz, D_2O)

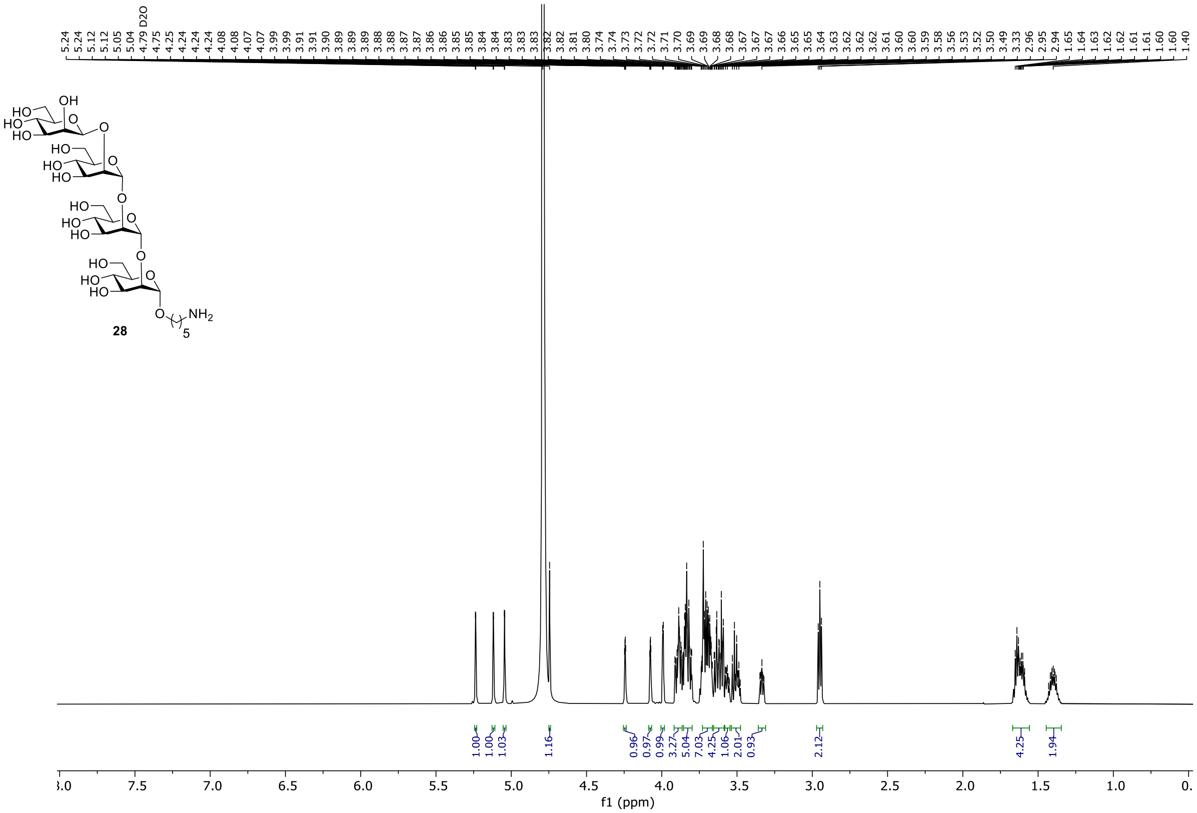

^{113}C NMR (176 MHz, D_2O)

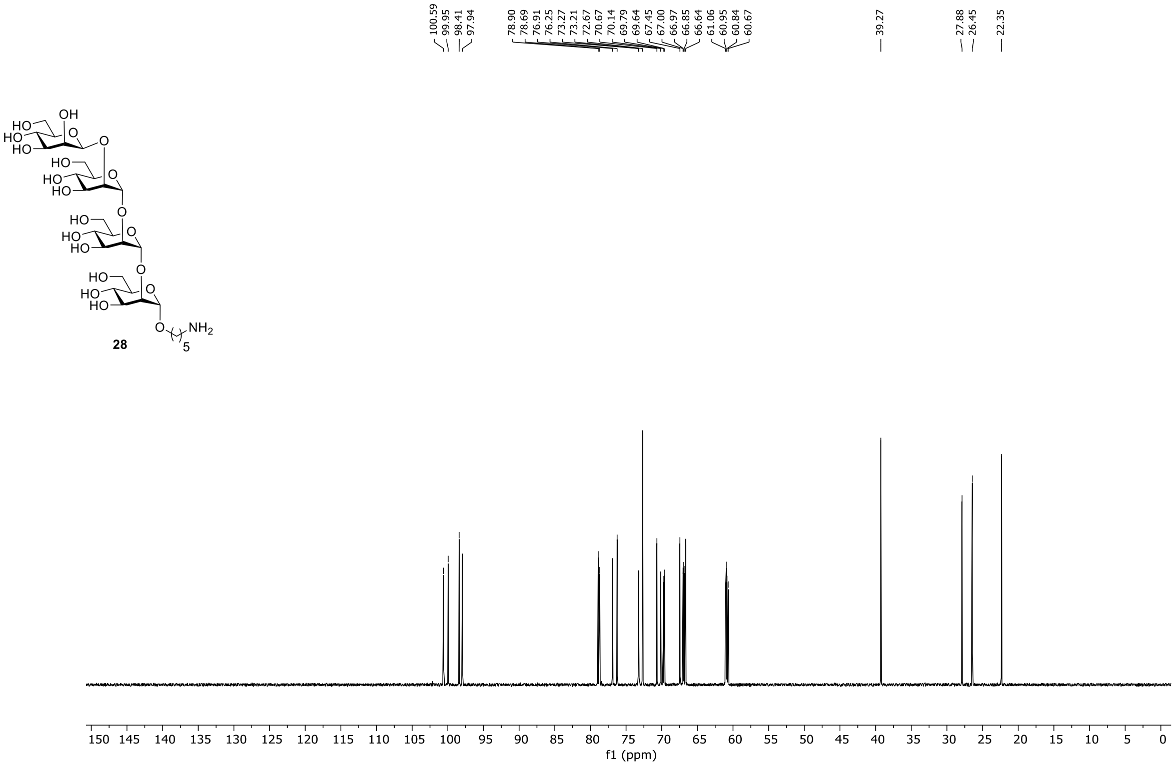

^{1}H-^{1}H COSY NMR (700 MHz, D_2O)

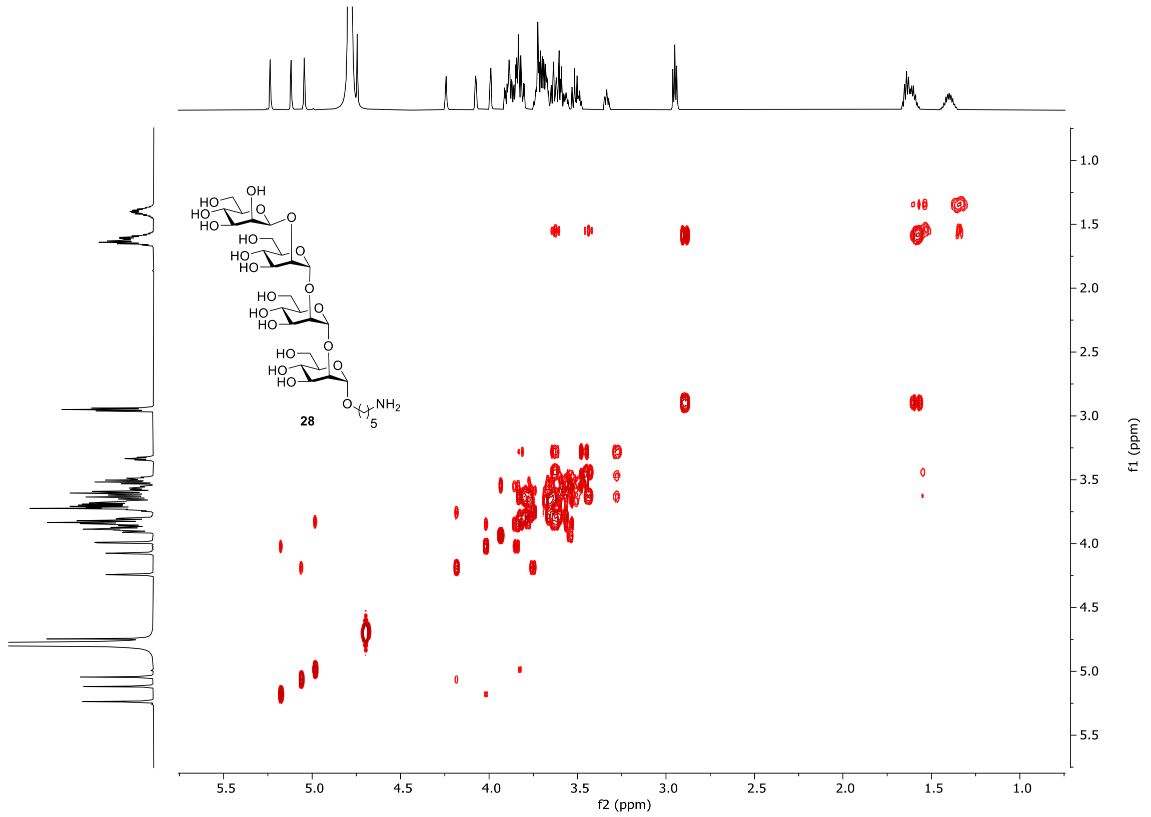

^{1}H-^{13}C HSQC NMR (700 MHz, D_2O)

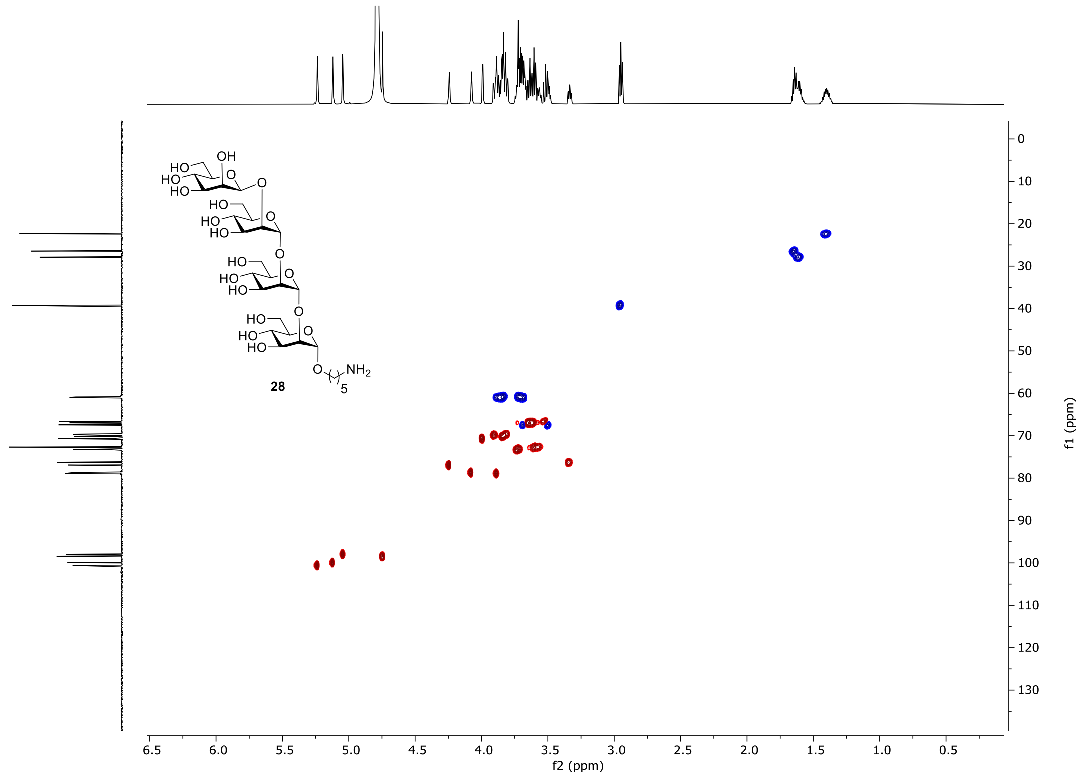

^{1}H-^{13}C Coupled HSQC NMR (700 MHz, D_2O)

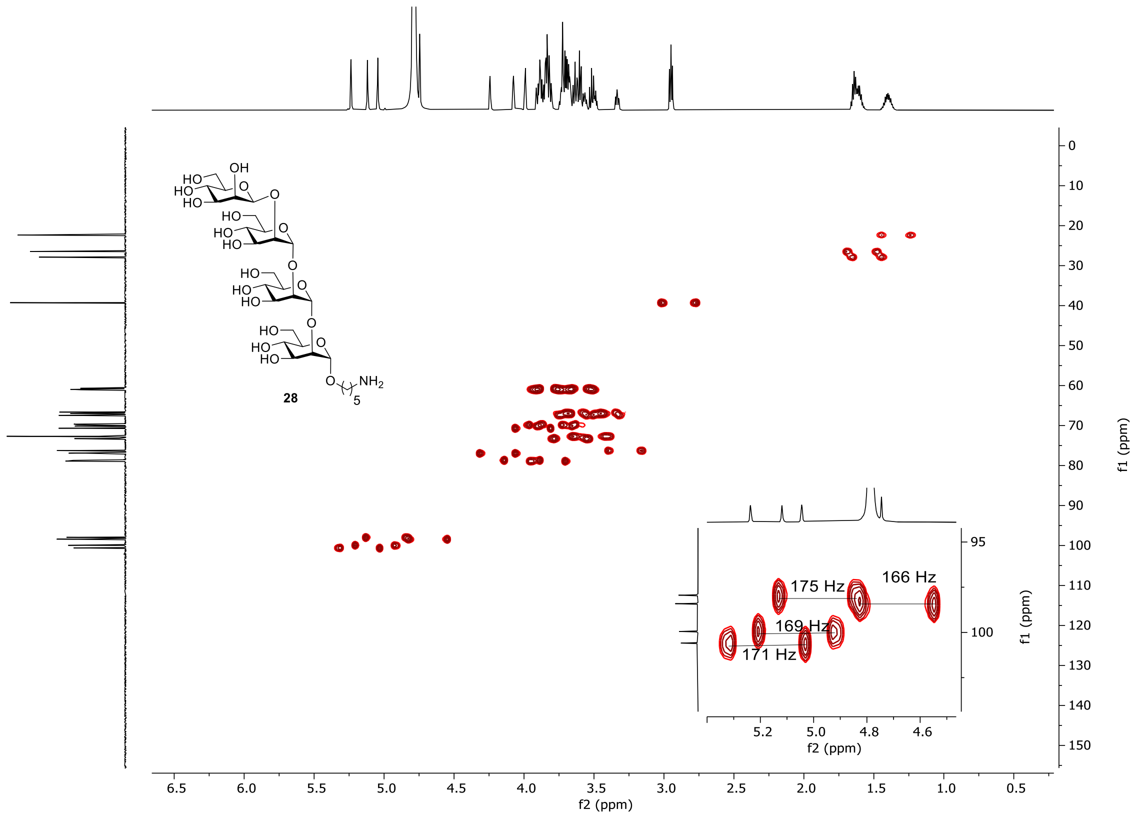

^{1}H NMR (400 MHz, D_2O)

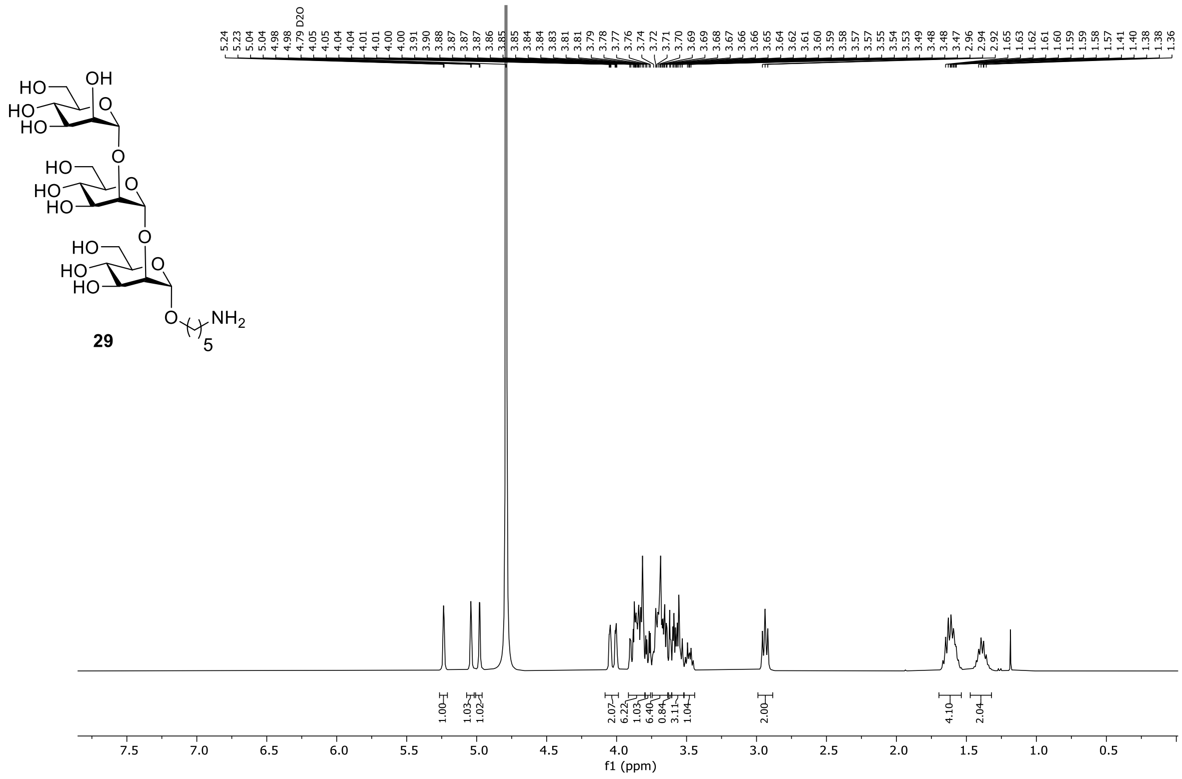

^{13}C NMR (400 MHz, D_2O)

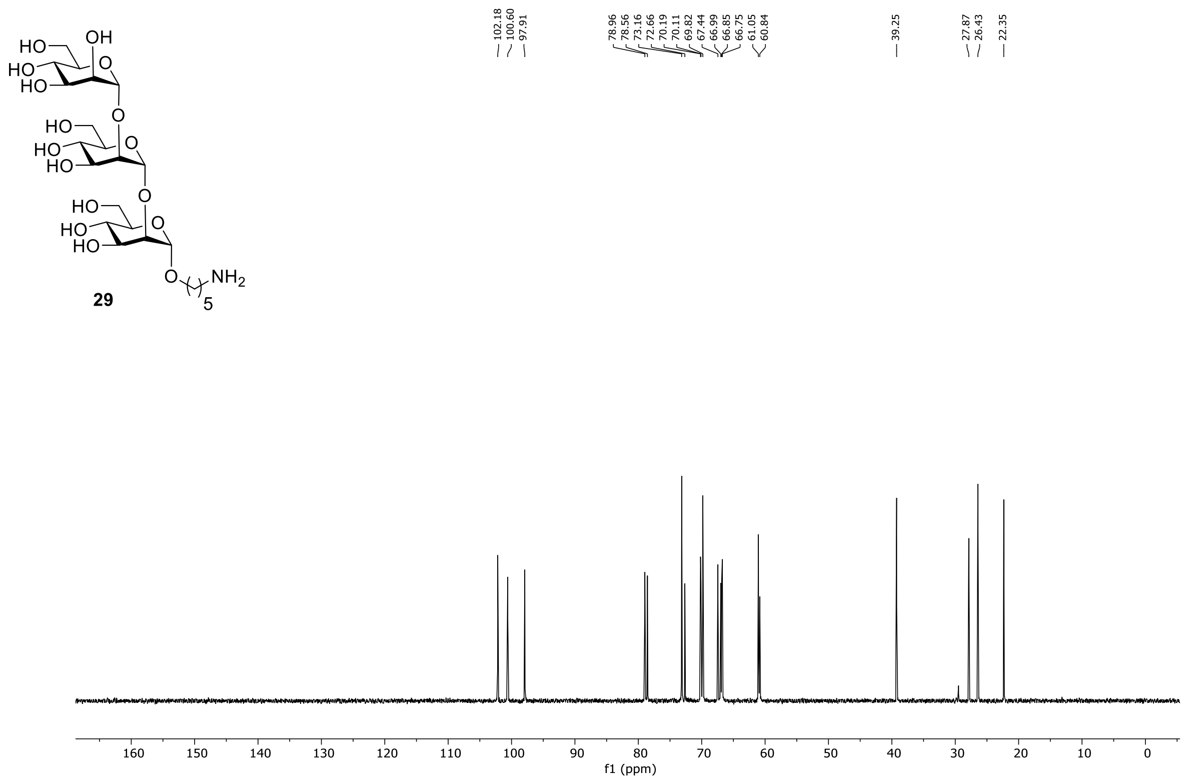

^1H-^1H COSY NMR (400 MHz, D_2O)

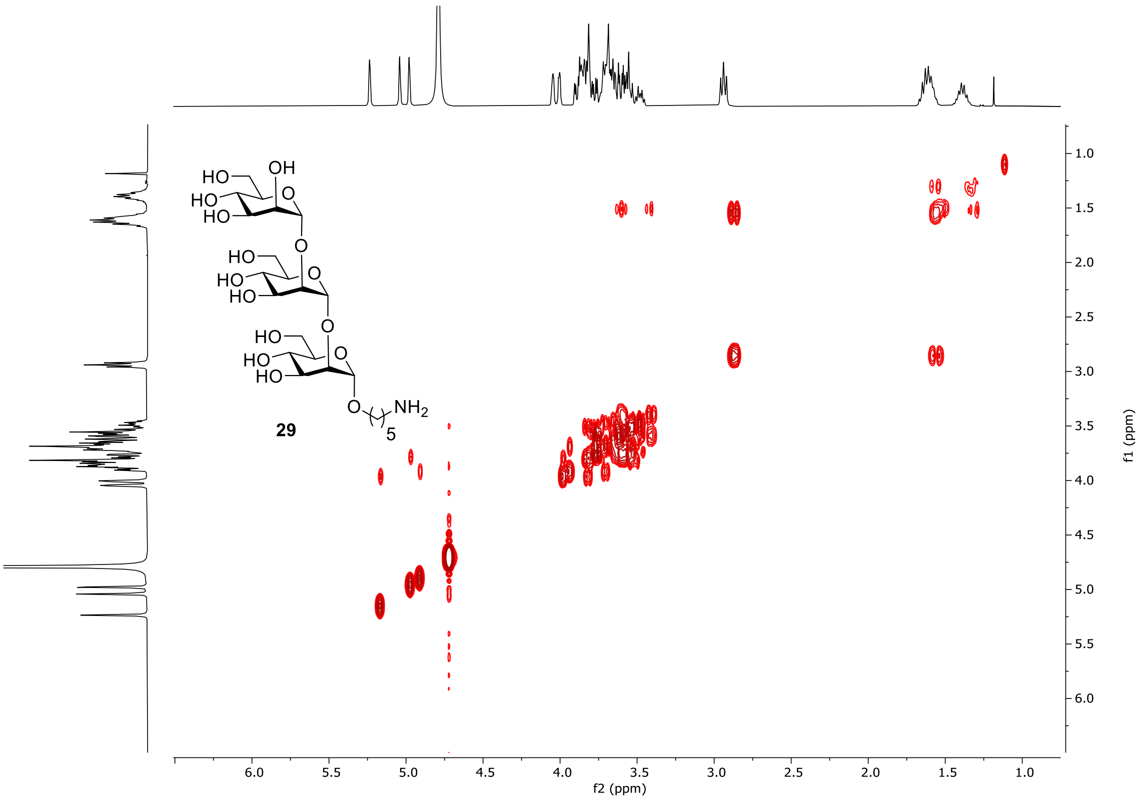

^{1}H-^{13}C HSQC NMR (400 MHz, D_2O)

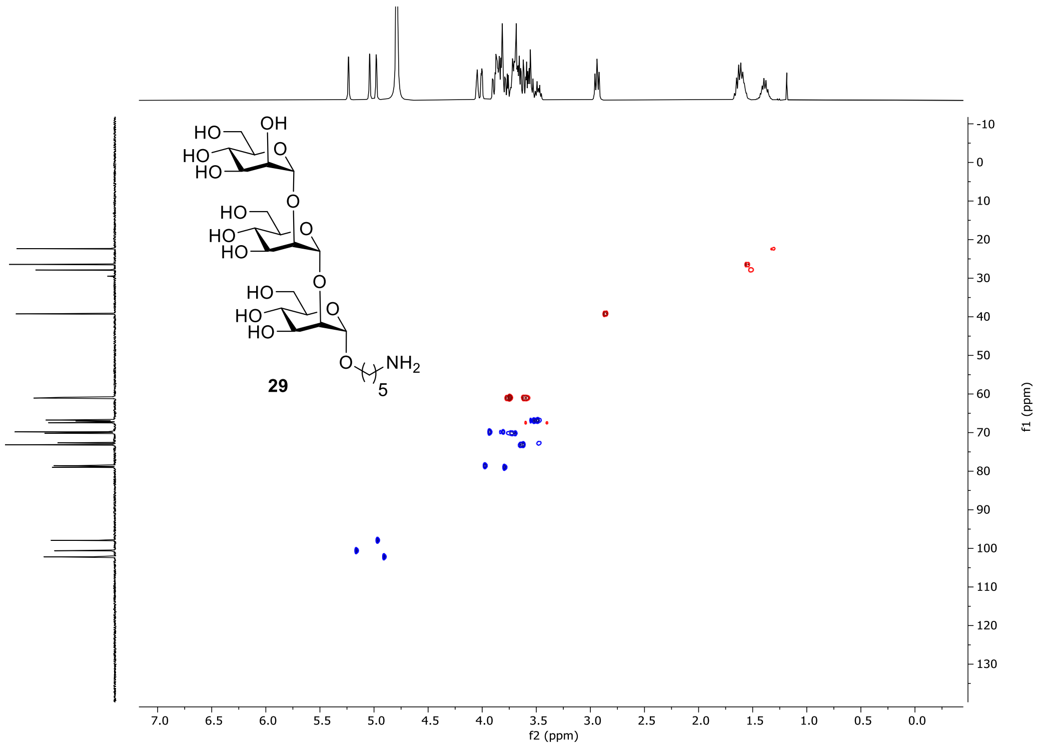

^{1}H-^{13}C Coupled HSQC NMR (400 MHz, D_2O)

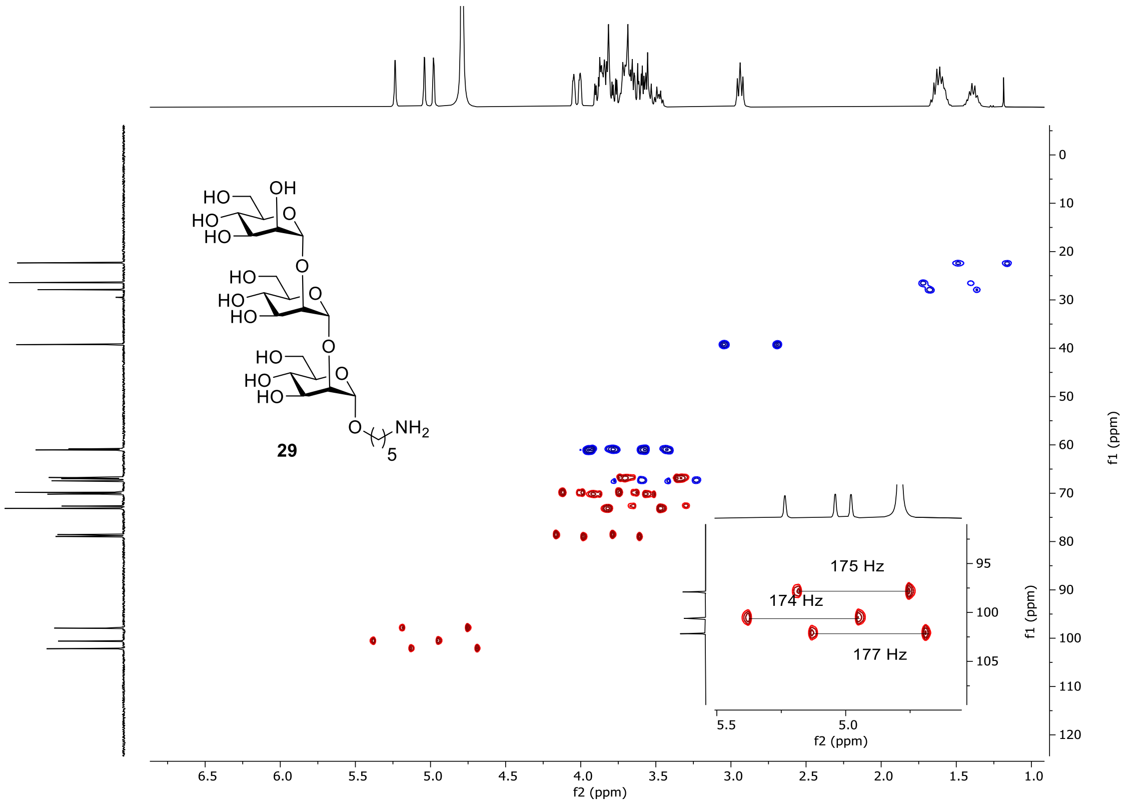

^{1}H NMR (400 MHz, $CDCl_3$)

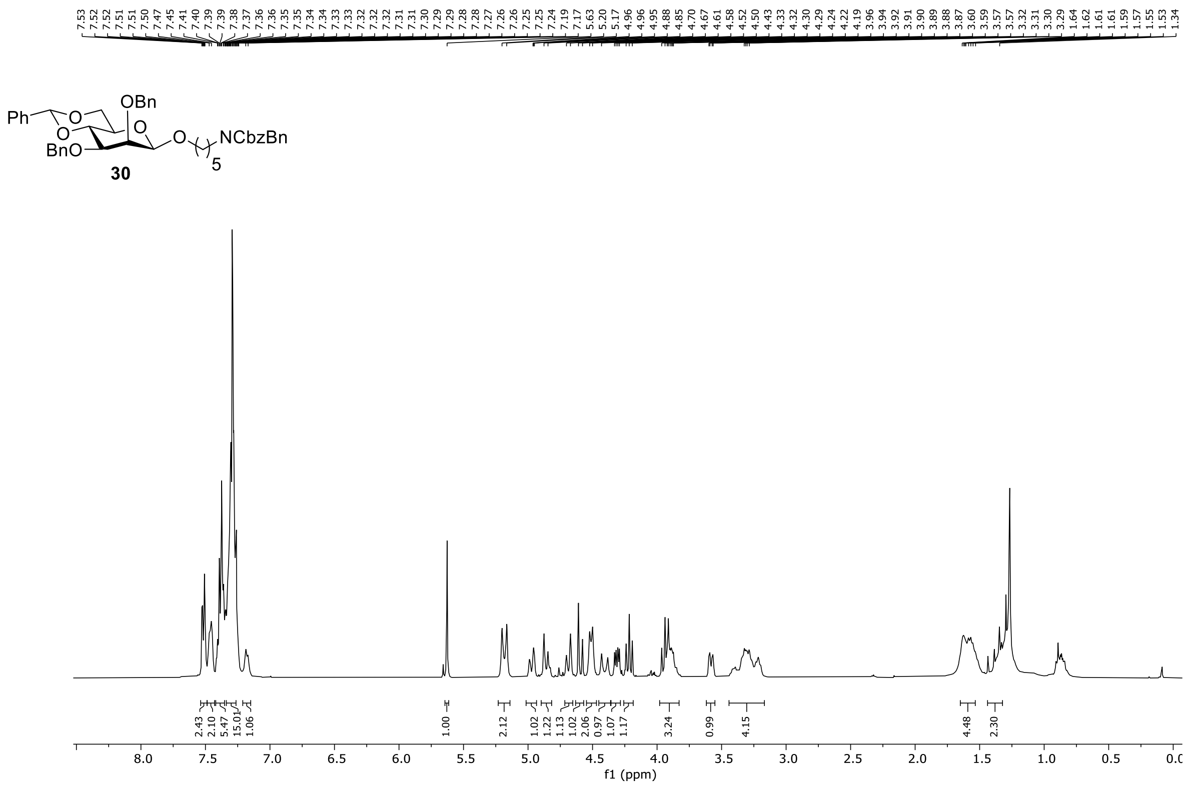

^{13}C NMR (101 MHz, $CDCl_3$)

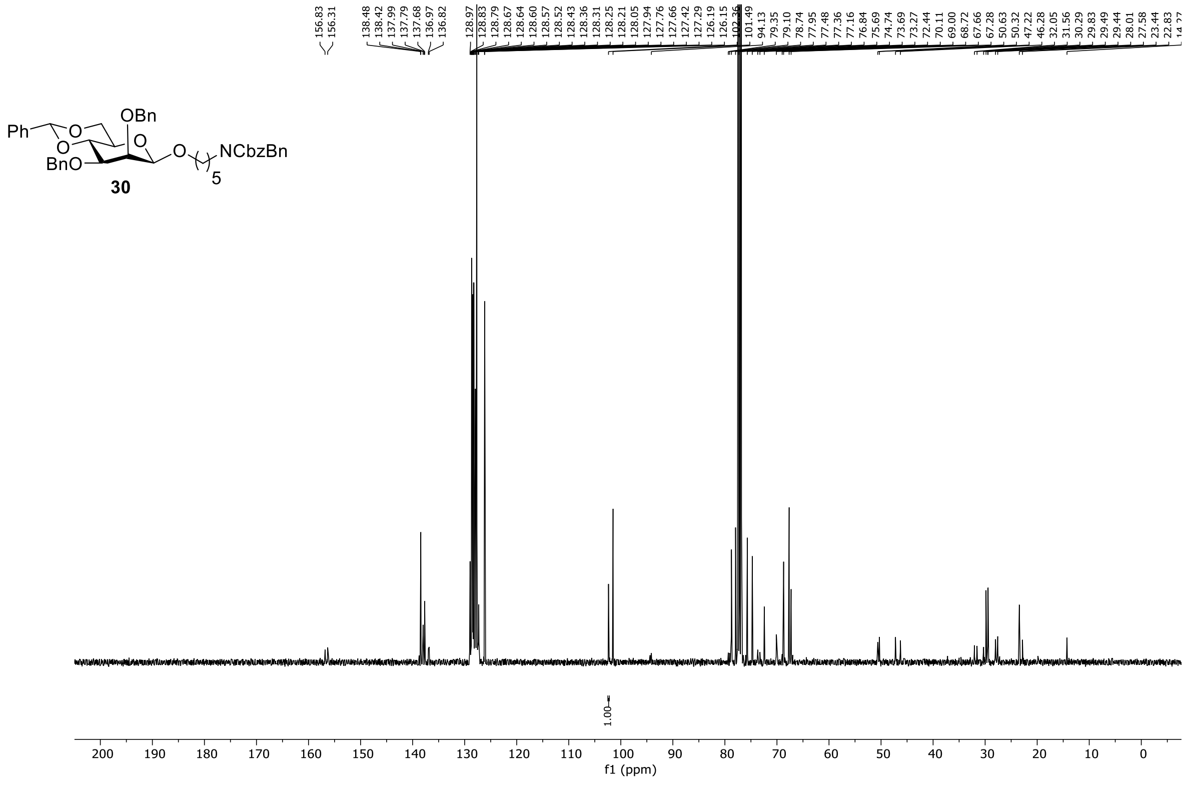

^{1}H-^{1}H COSY NMR (400 MHz, CDCl$_3$)

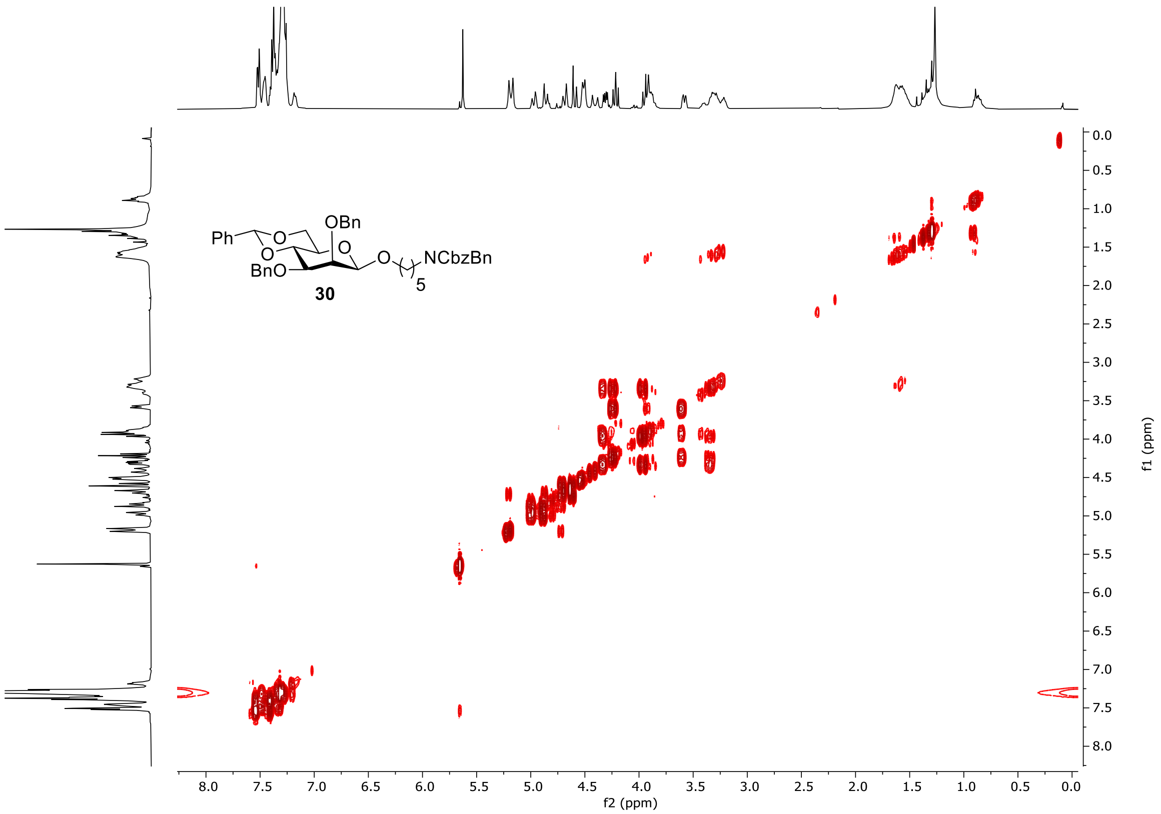

^{1}H-^{13}C HSQC NMR (400 MHz, CDCl$_3$)

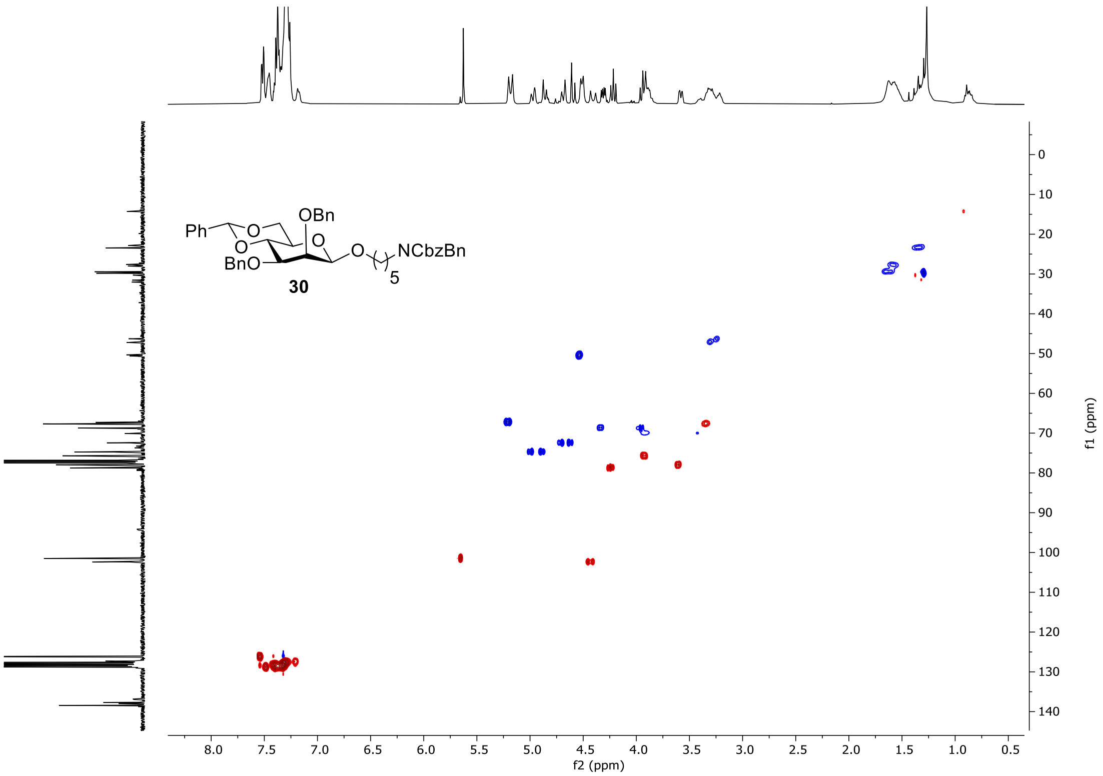

^{1}H-^{13}C Coupled HSQC NMR (400 MHz, $CDCl_3$)

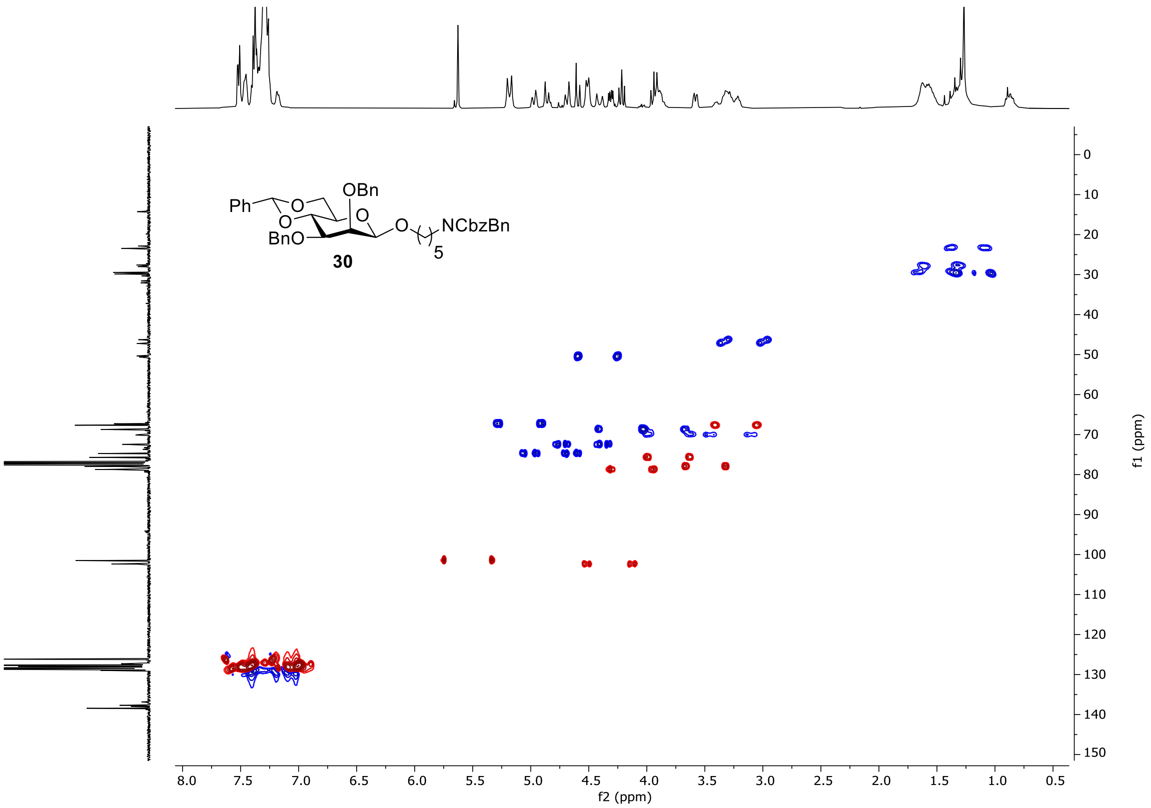

^{1}H NMR (600 MHz, $CDCl_3$)

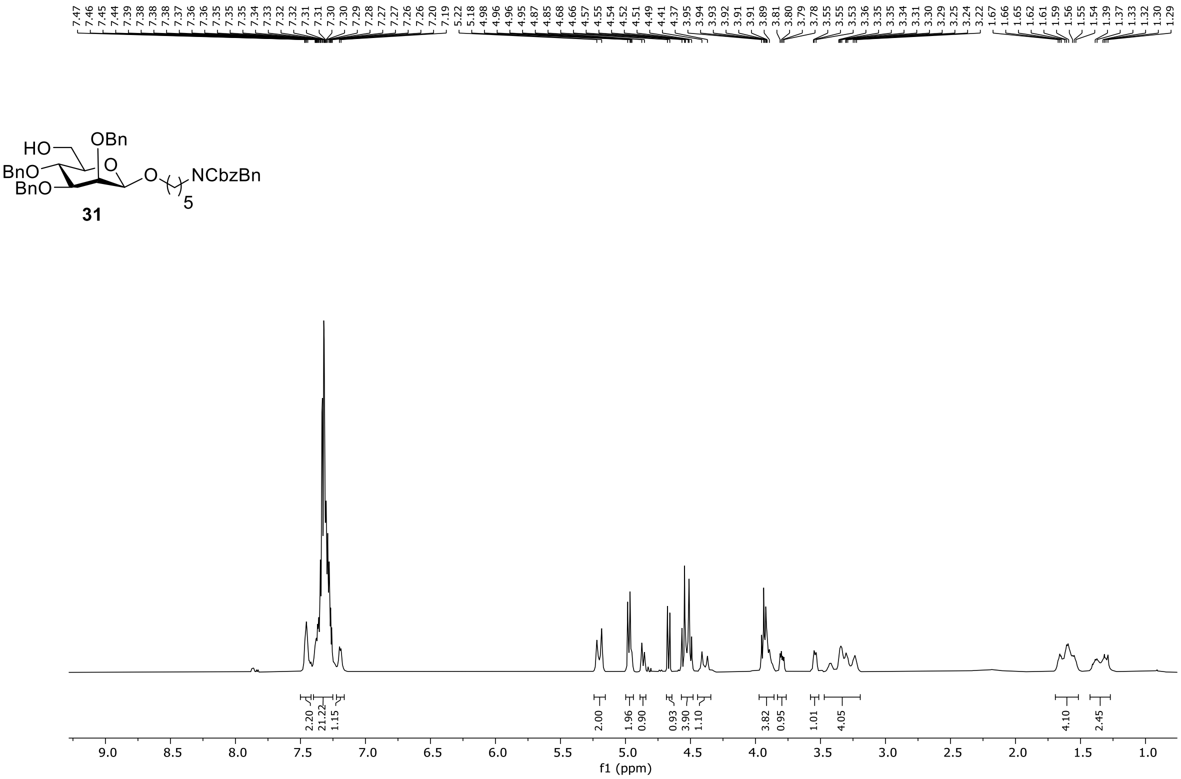

^{13}C NMR (151 MHz, $CDCl_3$)

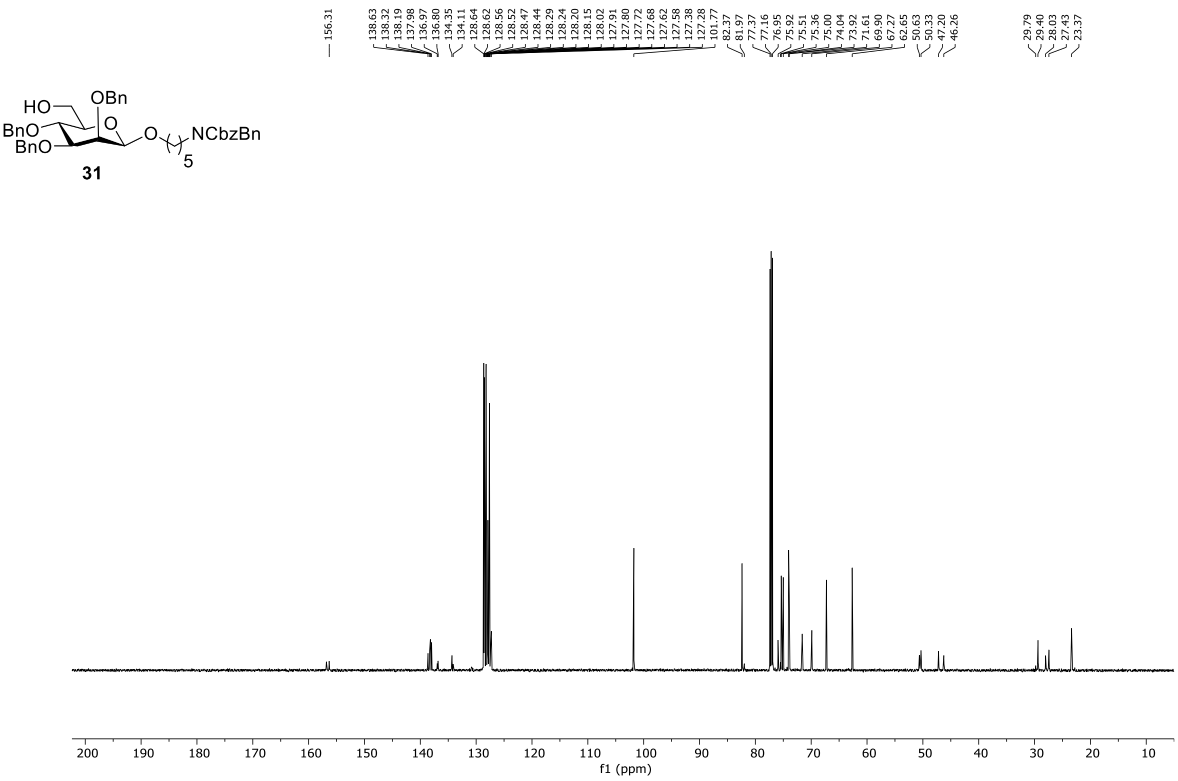

^{1}H-^{1}H COSY NMR (600 MHz, $CDCl_3$)

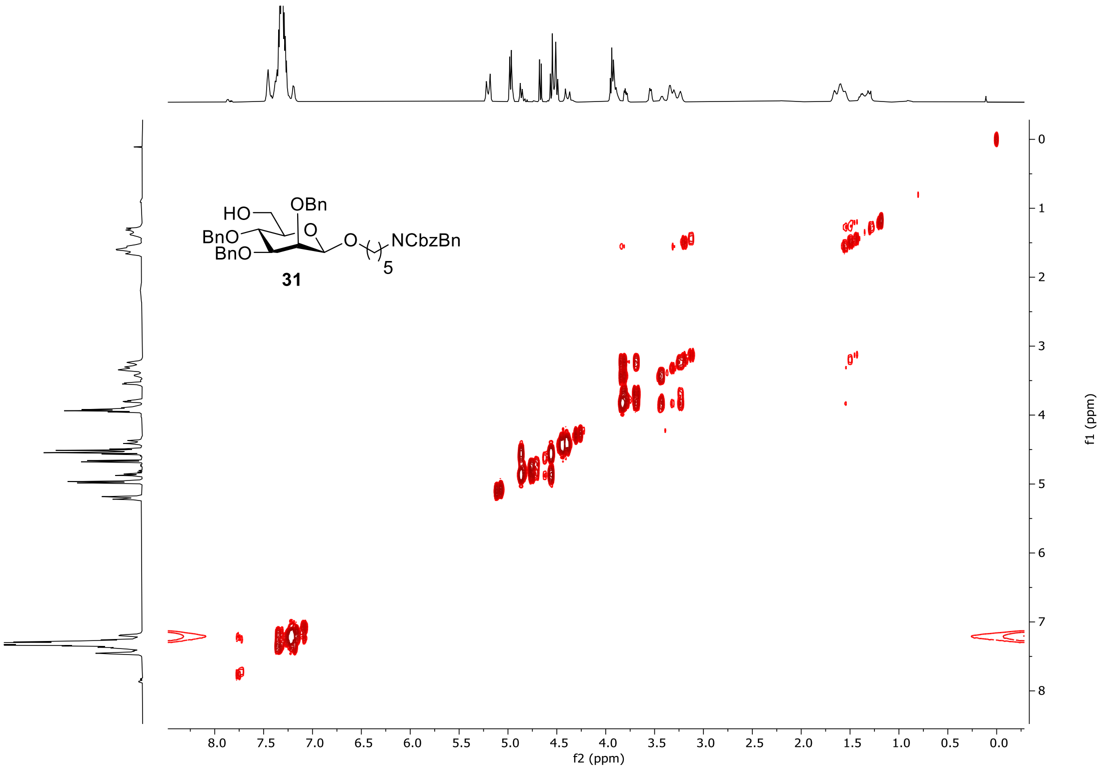

^{1}H-^{13}C HSQC NMR (600 MHz, $CDCl_3$)

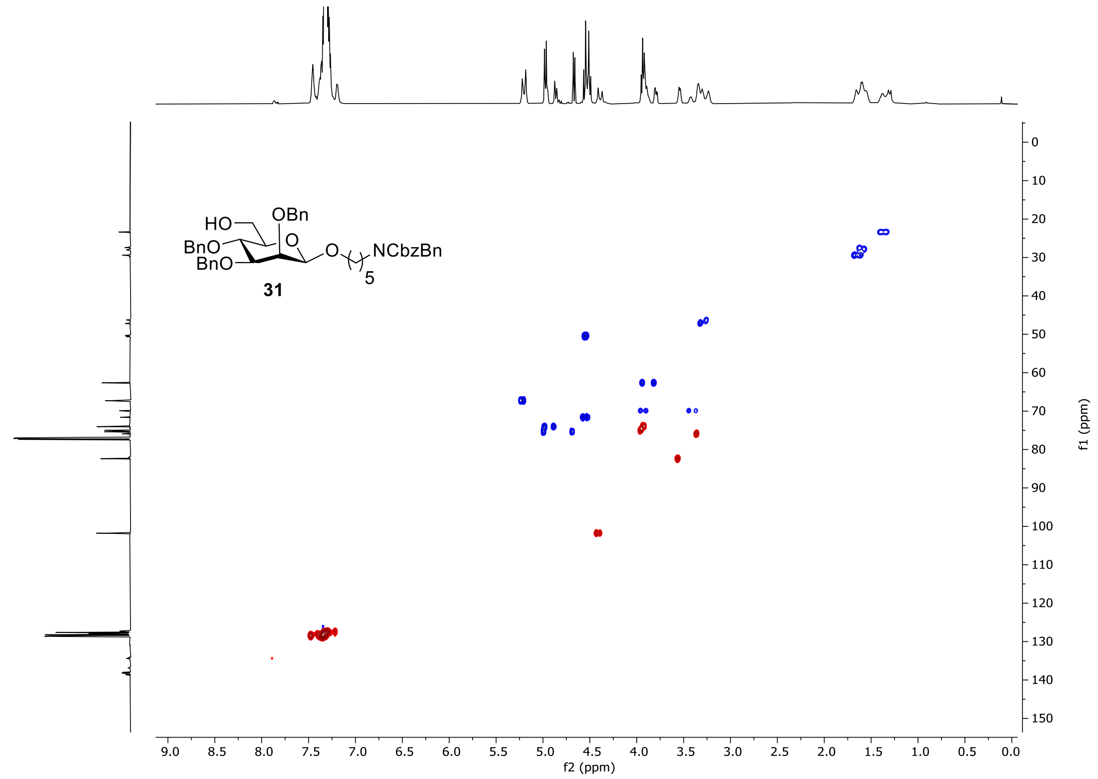

^{1}H-^{13}C Coupled HSQC NMR (600 MHz, $CDCl_3$)

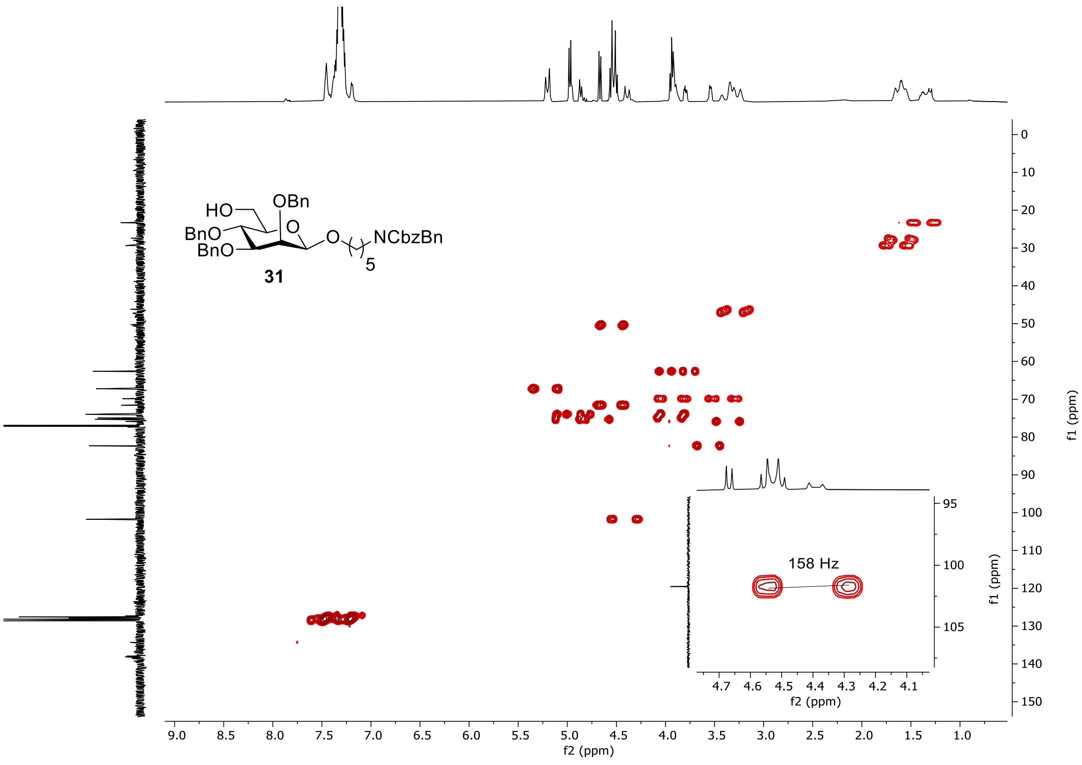

^{1}H NMR (600 MHz, $CDCl_3$)

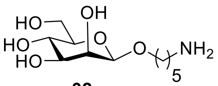

32

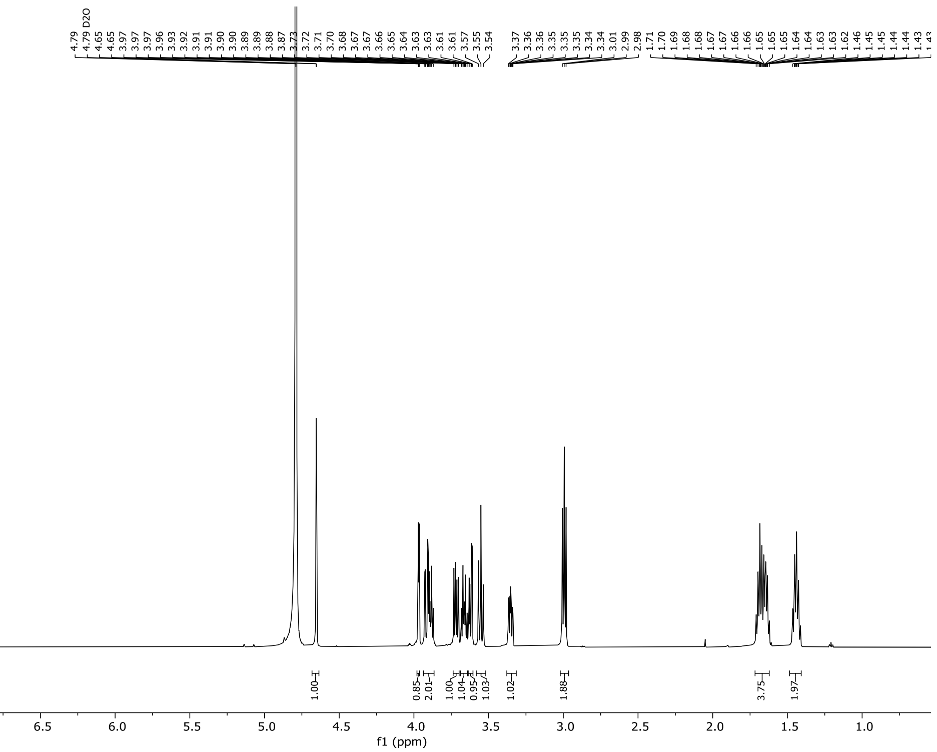

^{13}C NMR (151 MHz, $CDCl_3$)

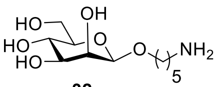

32

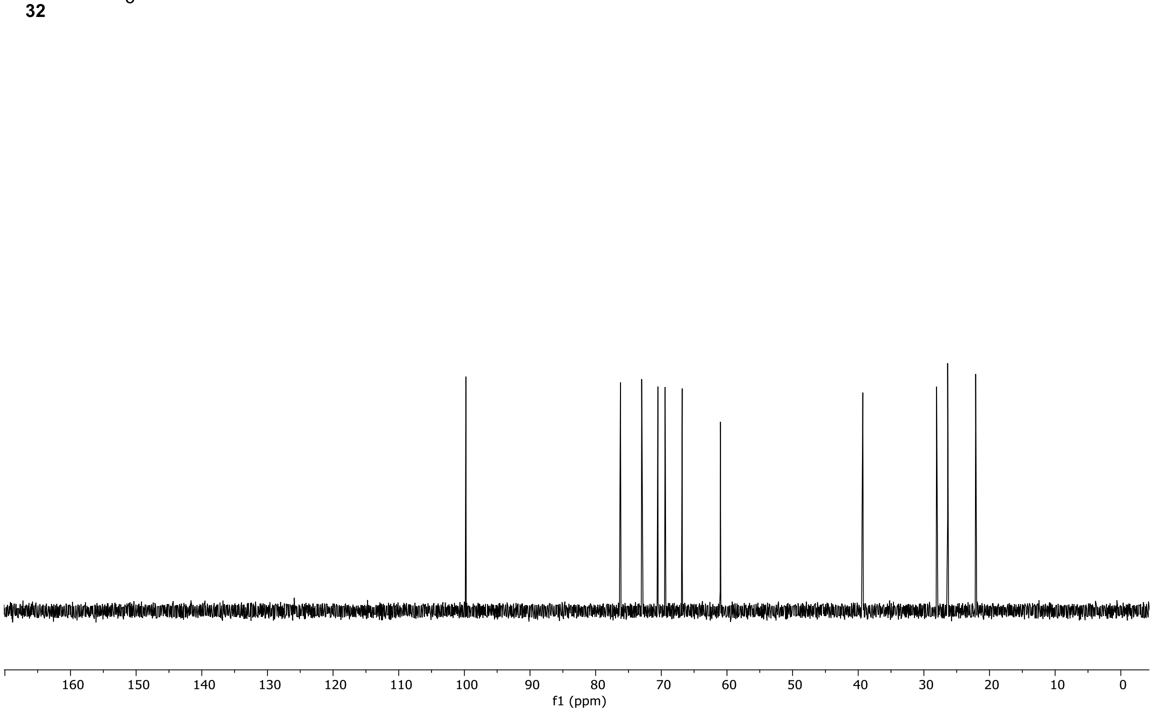

^{1}H-^{1}H COSY NMR (600 MHz, $CDCl_3$)

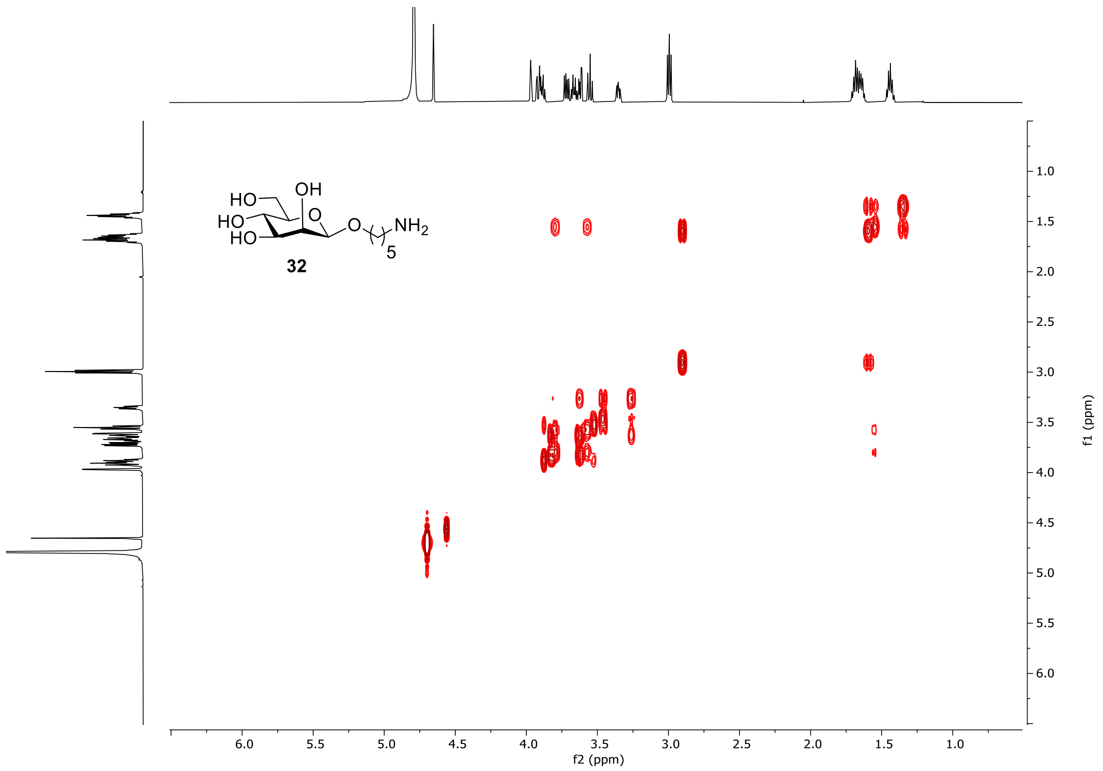

^{1}H-^{13}C HSQC NMR (600 MHz, $CDCl_3$)

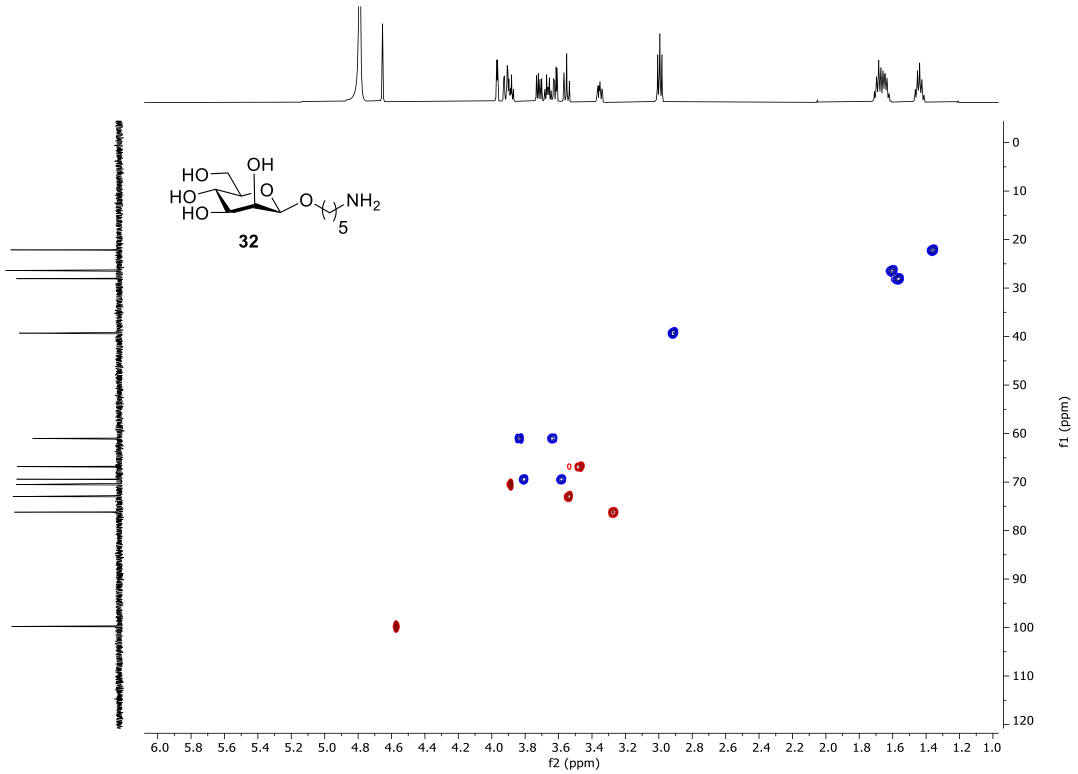

^{1}H-^{13}C Coupled HSQC NMR (600 MHz, $CDCl_3$)

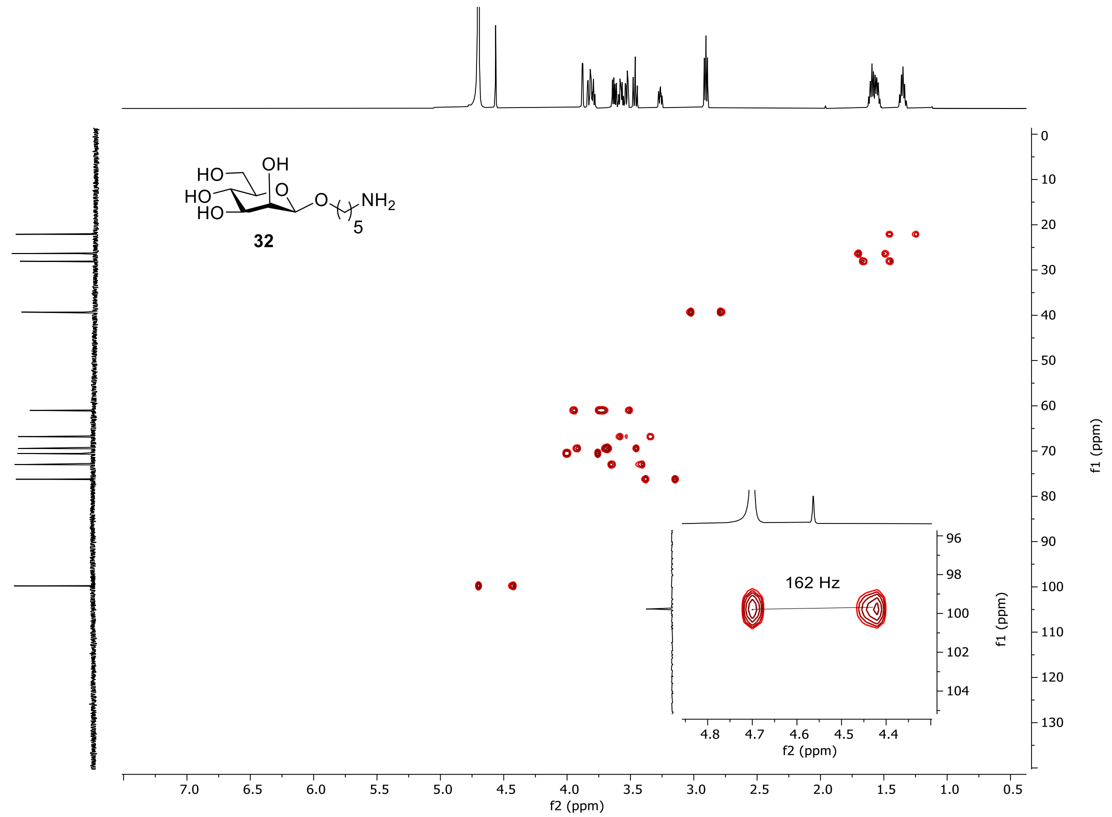

^{1}H NMR (600 MHz, D_2O)

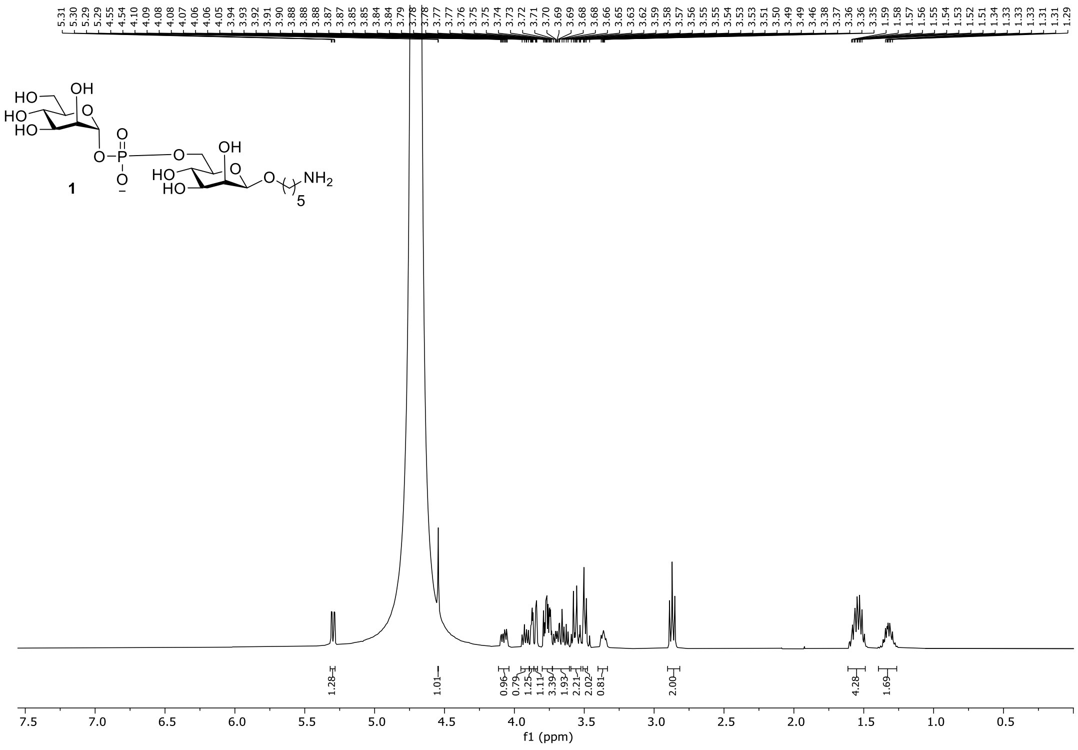

^{13}C NMR (151 MHz, D_2O)

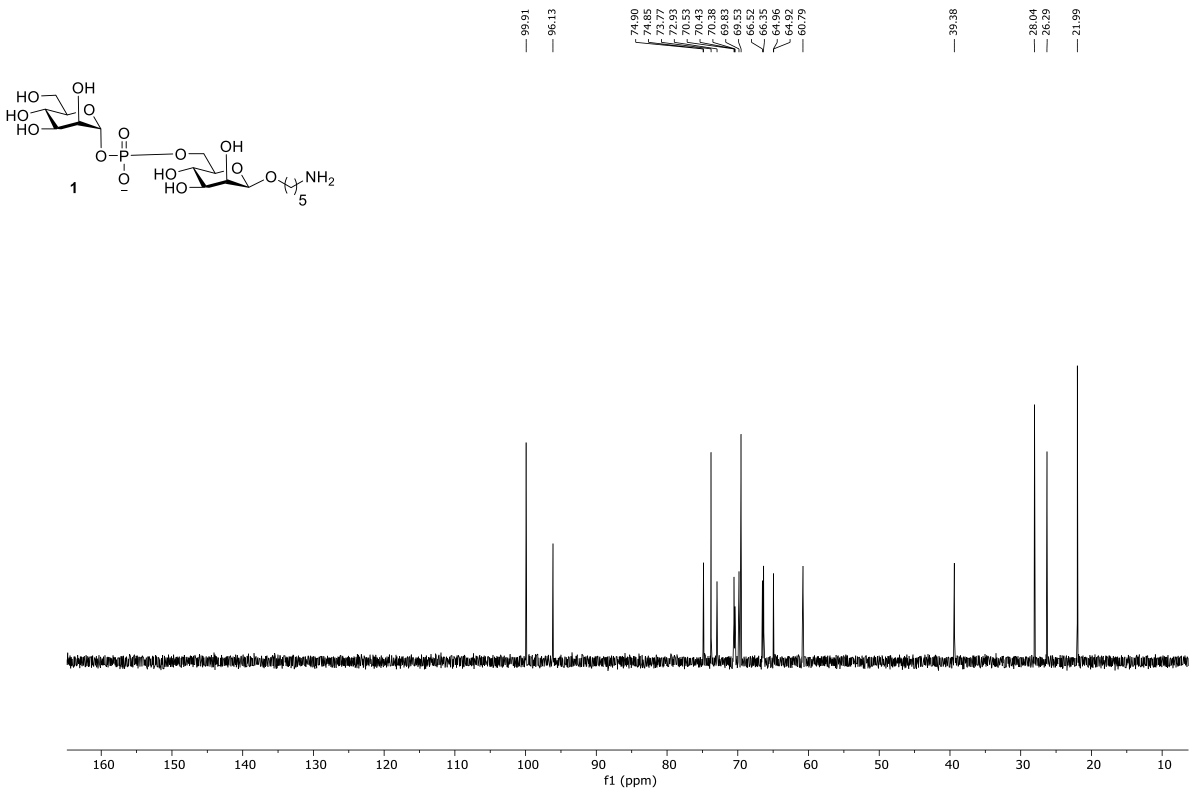

^{1}H-^{13}C HSQC NMR (600 MHz, D_2O)

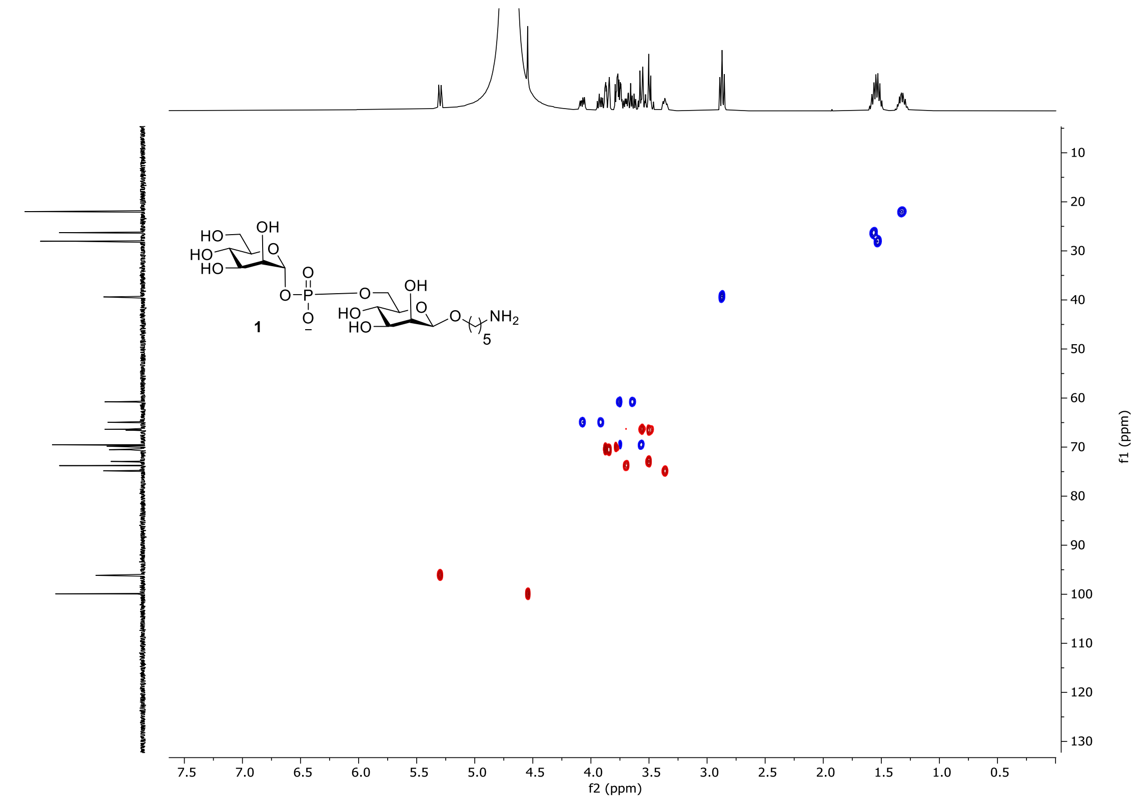

^{1}H-^{13}C Coupled HSQC NMR (600 MHz, D_2O)

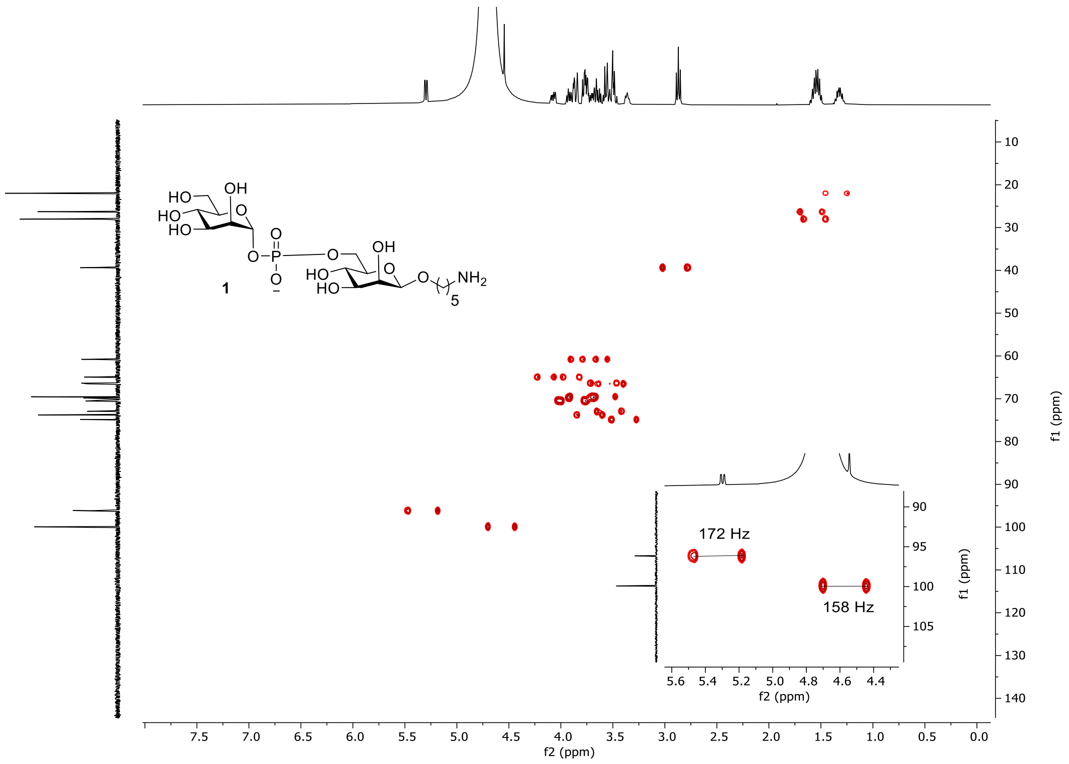

^{31}P NMR (162 MHz, D_2O)

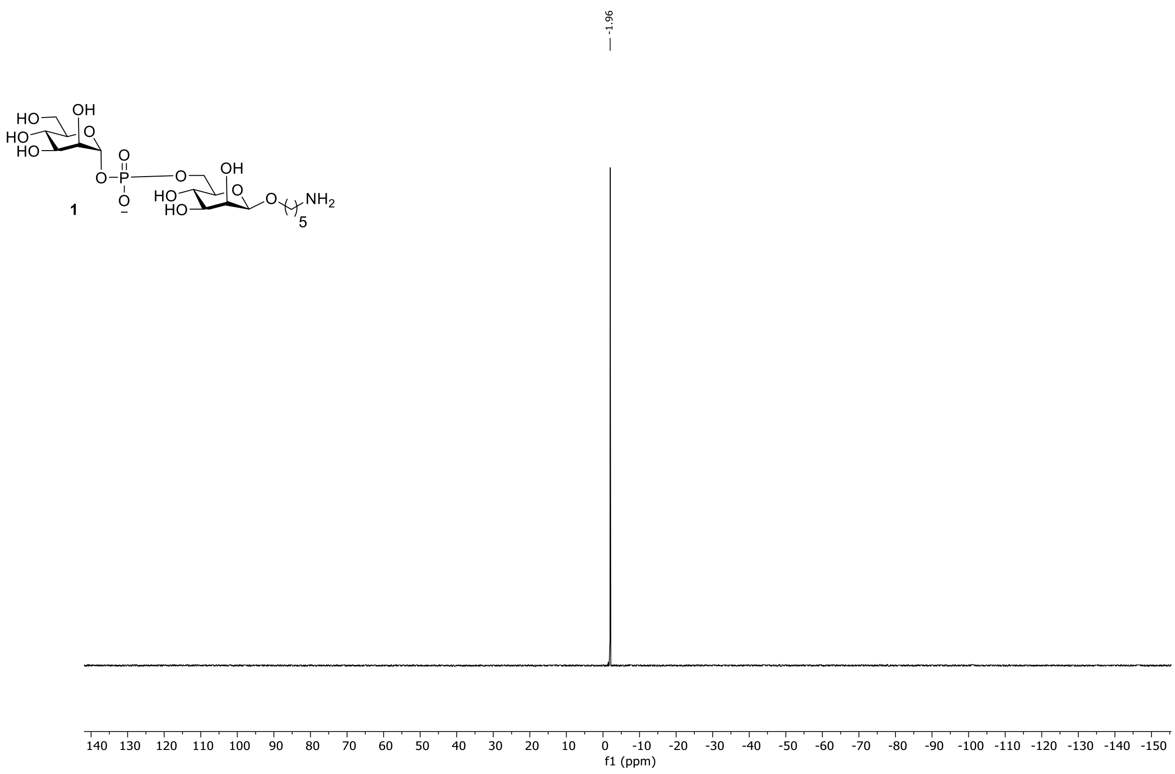

^{1}H NMR (600 MHz, CDCl$_3$)

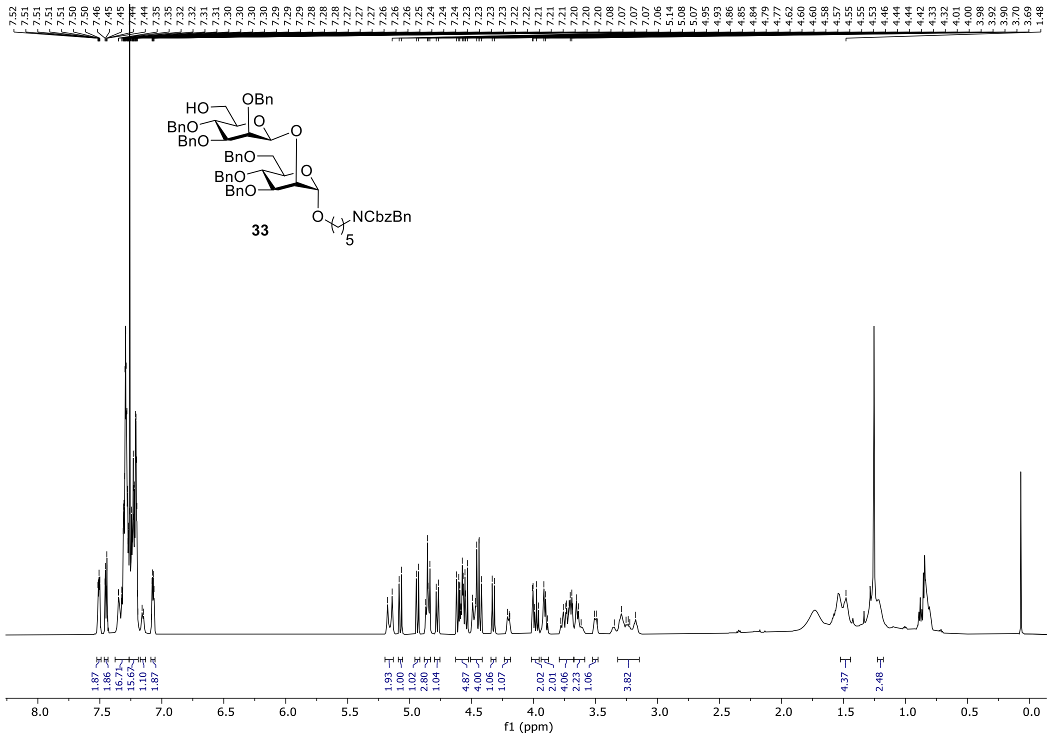

^{13}C NMR (151 MHz, CDCl$_3$)

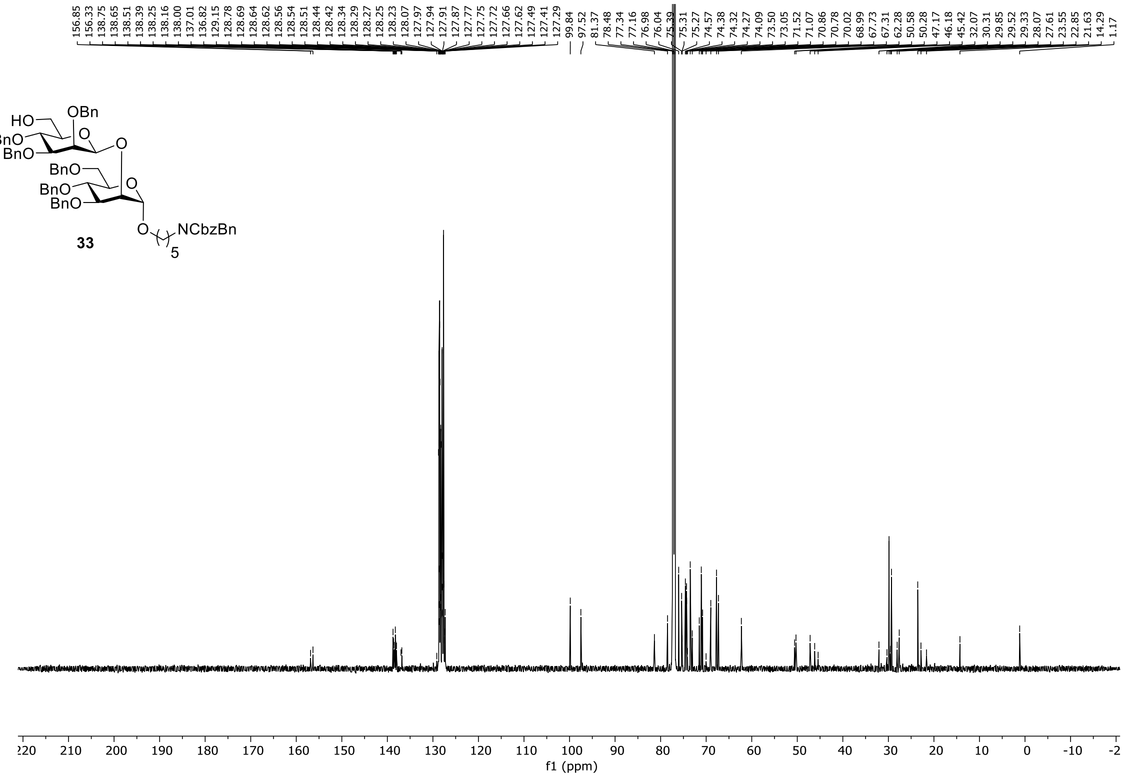

^{1}H-^{1}H COSY NMR (600 MHz, $CDCl_3$)

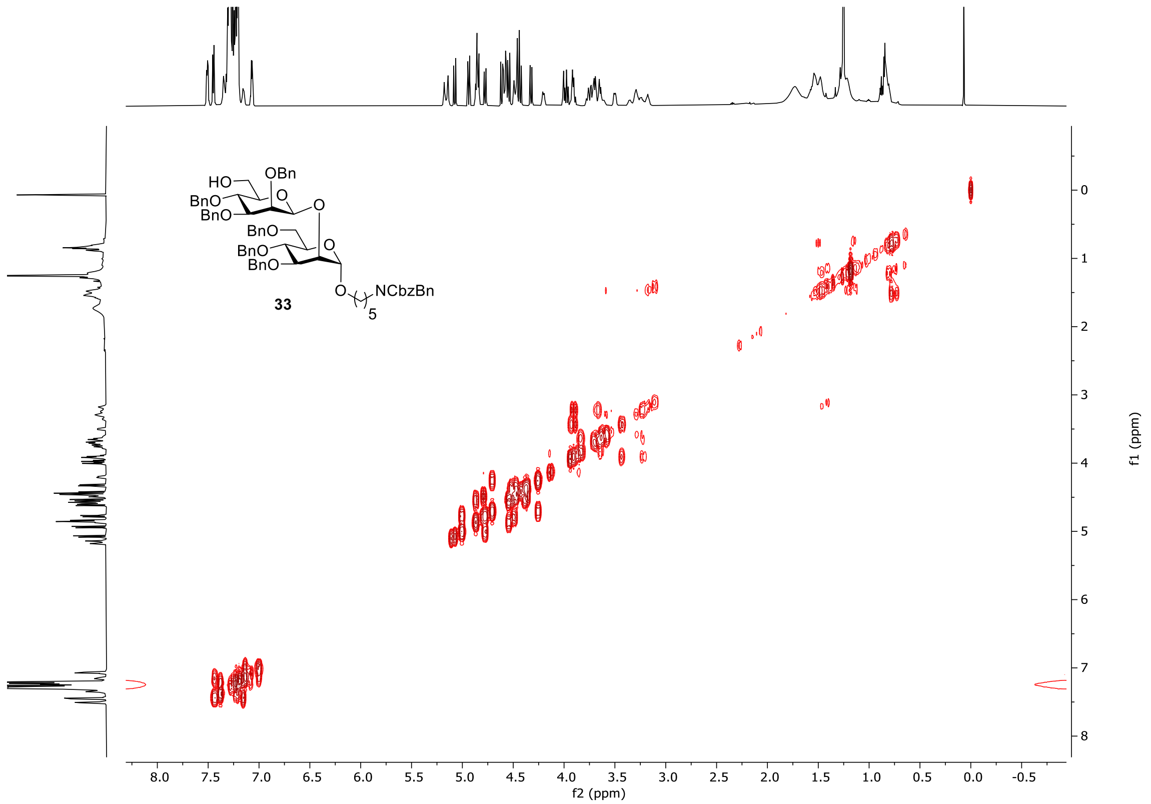

^{1}H-^{13}C HSQC NMR (600 MHz, $CDCl_3$)

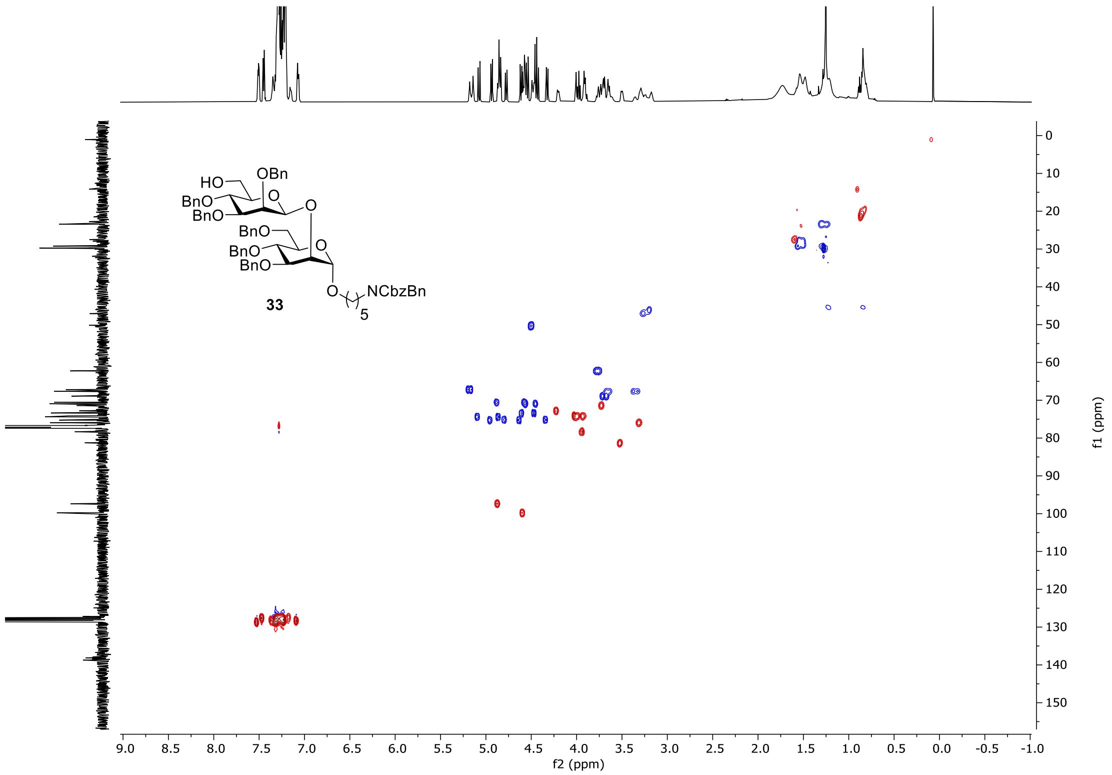

^1H-^{13}C Coupled HSQC NMR (600 MHz, $CDCl_3$)

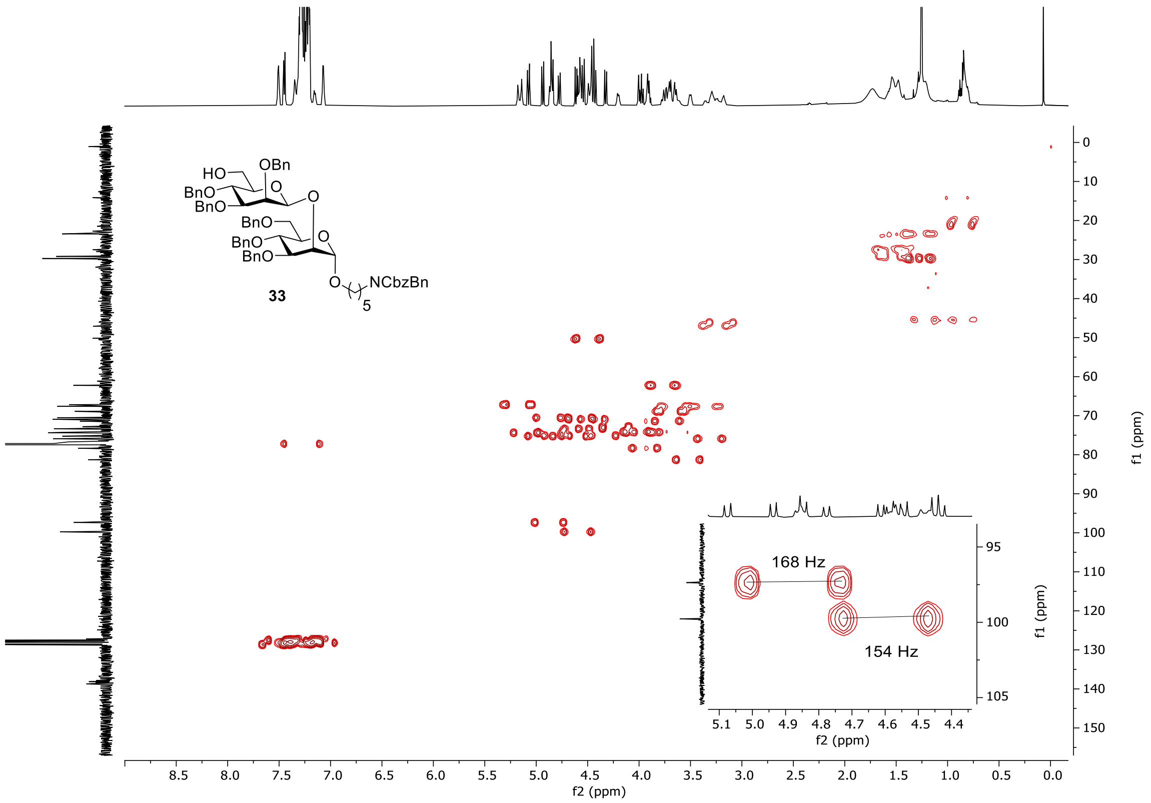

^1H NMR (600 MHz, D_2O)

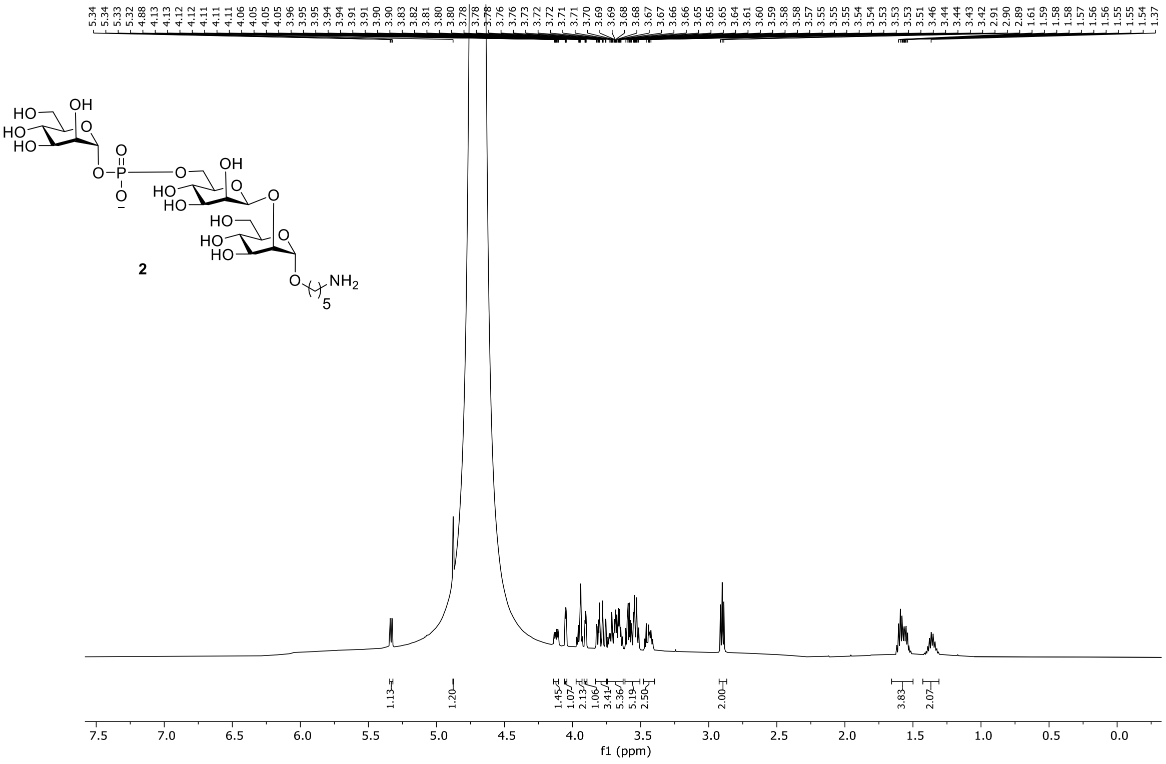

^{13}C NMR (151 MHz, D_2O)

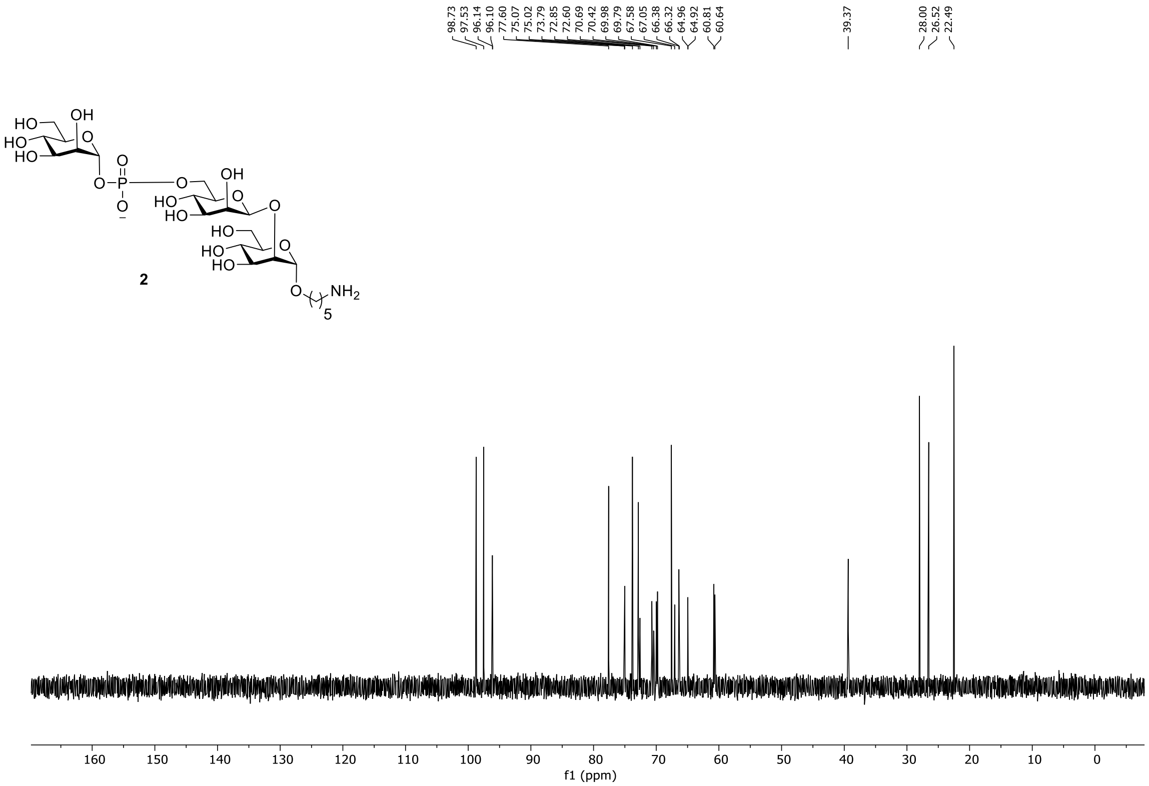

^{1}H-^{1}H COSY NMR (600 MHz, D_2O)

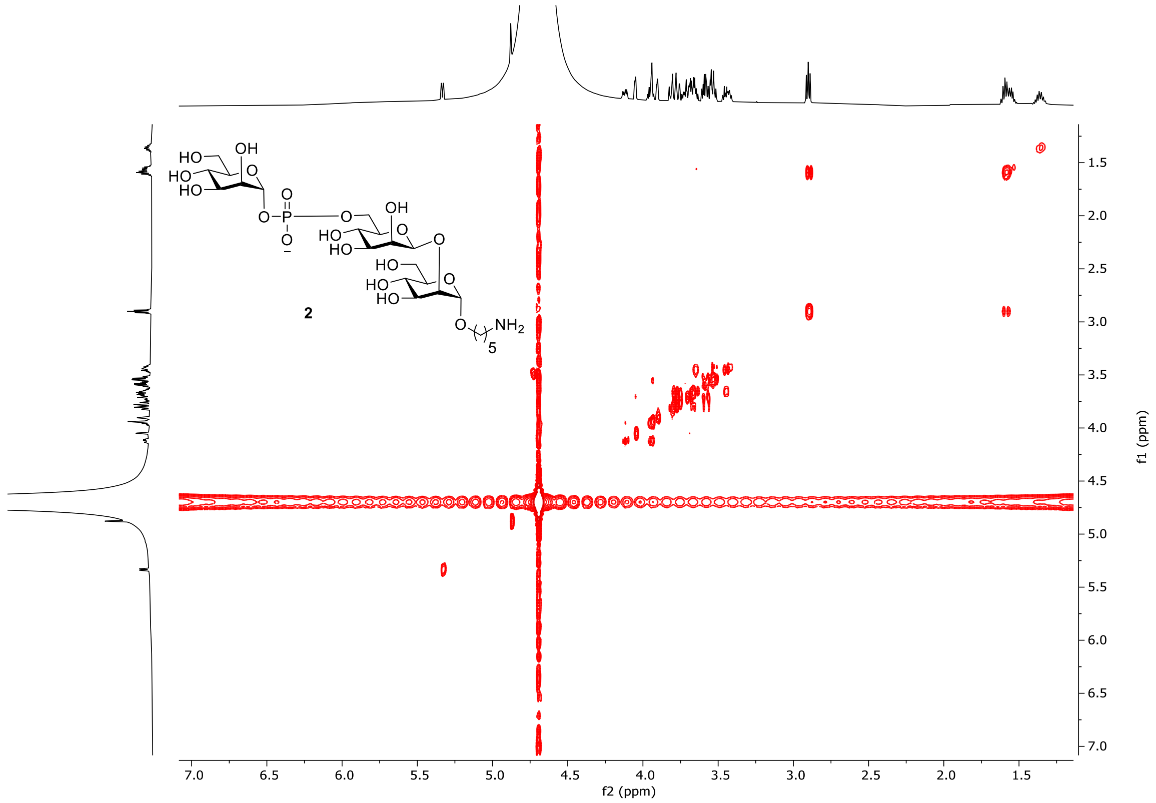

^1H-^{13}C HSQC NMR (600 MHz, D_2O)

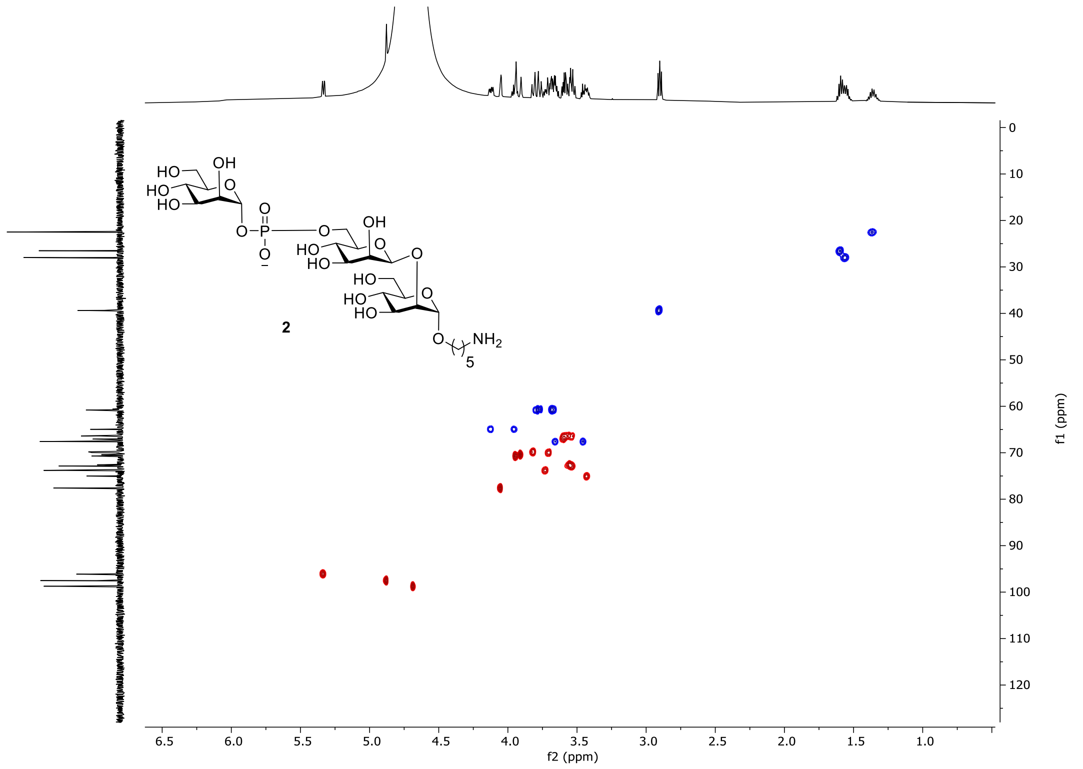

^1H-^{13}C Coupled HSQC NMR (600 MHz, D_2O)

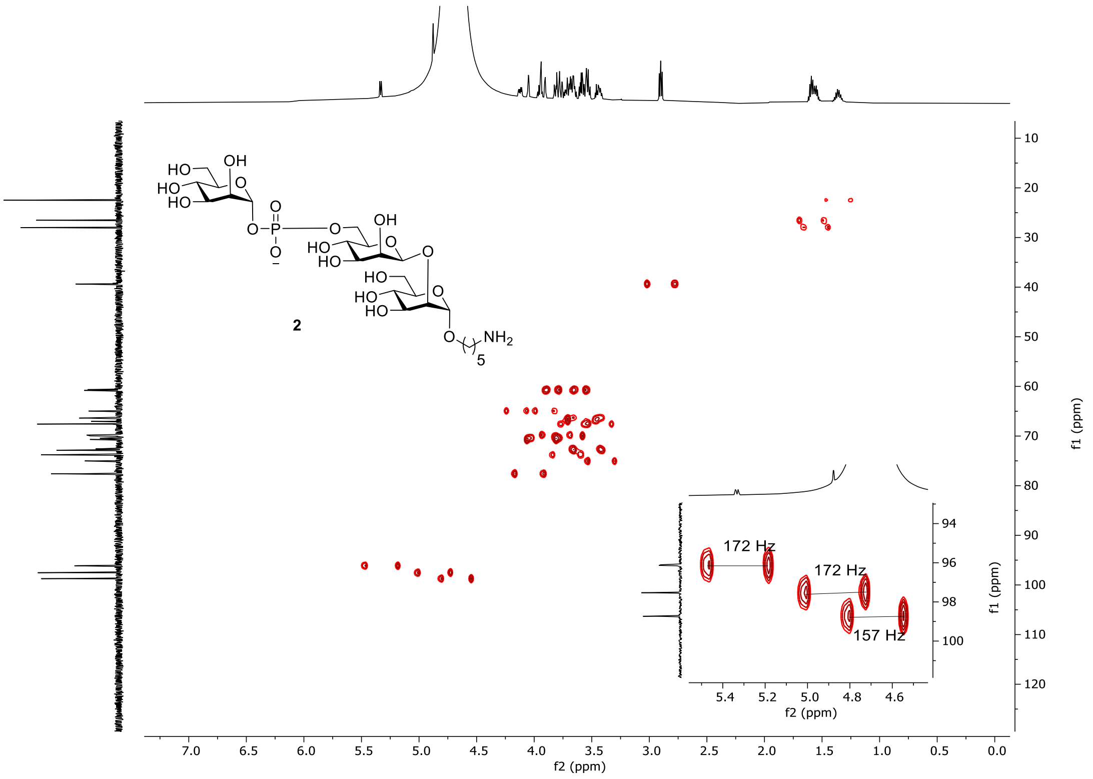

^{31}P NMR (162 MHz, D_2O)

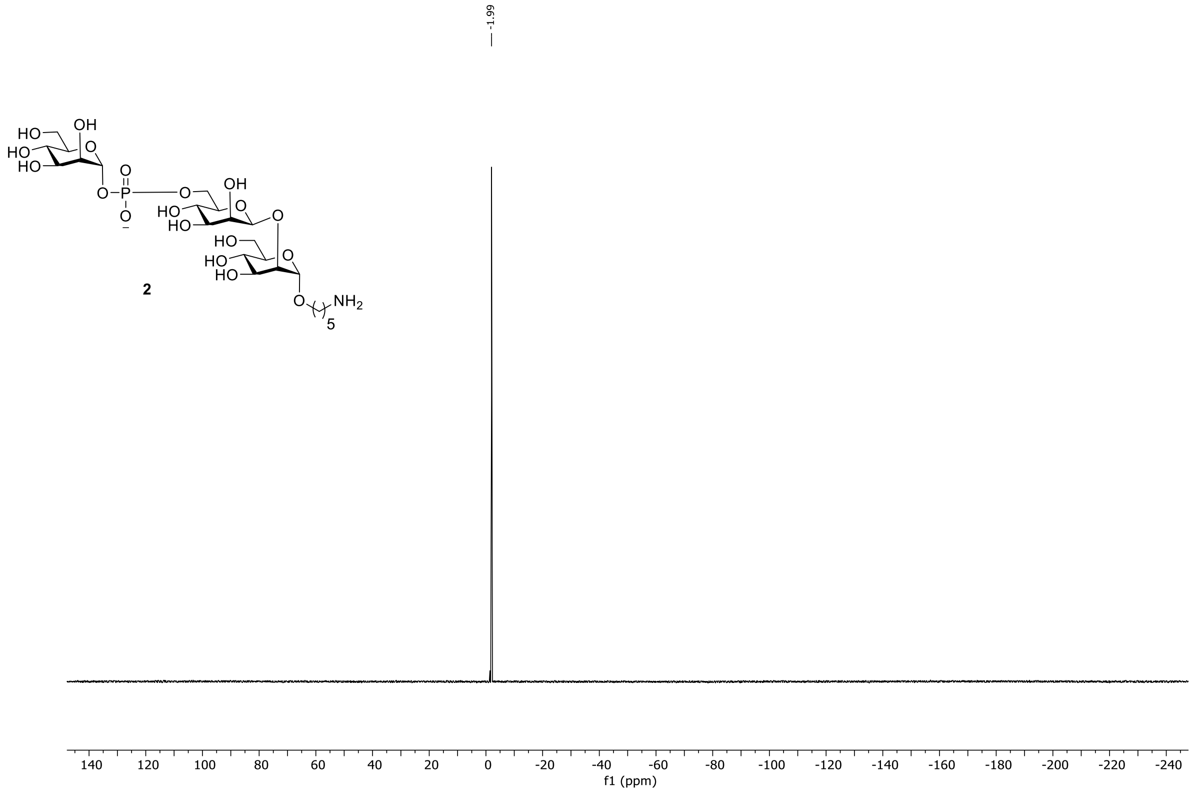

^{1}H-^{13}C HSQC NMR (400 MHz, $CDCl_3$)

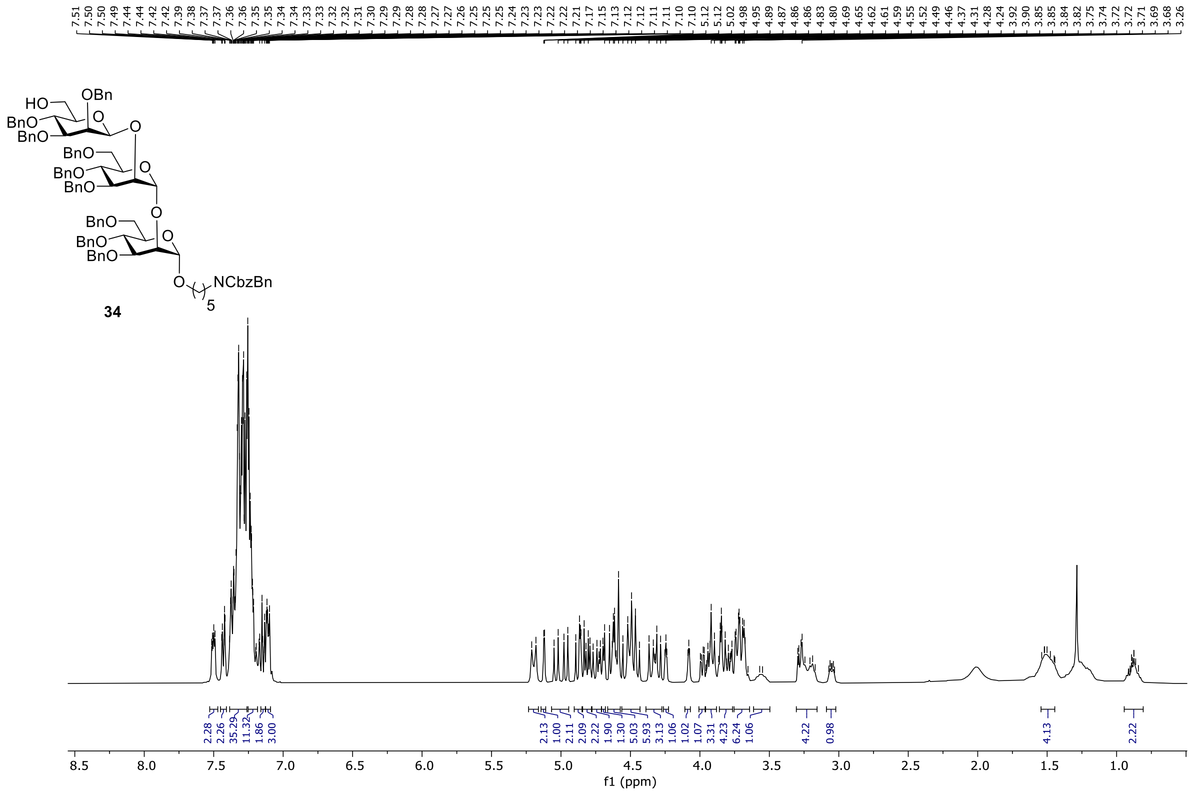

^{13}C NMR (400 MHz, $CDCl_3$)

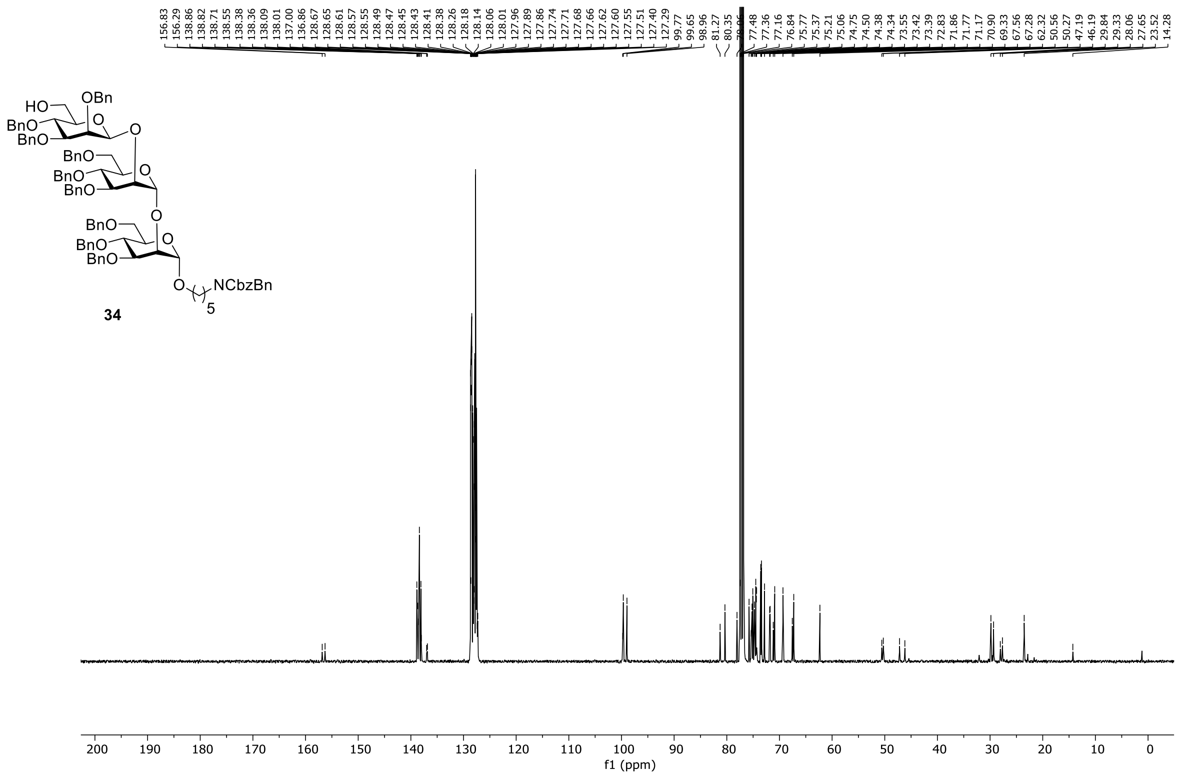

^{1}H-^{1}H COSY NMR (400 MHz, $CDCl_3$)

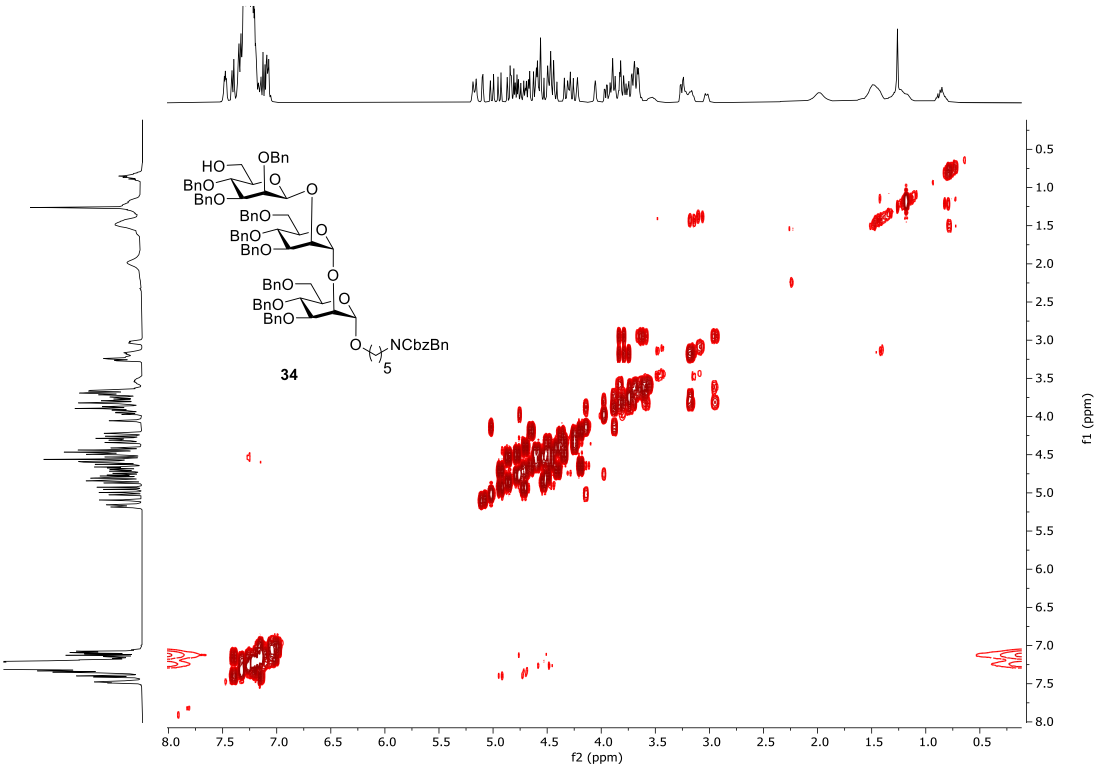

^{1}H-^{13}C HSQC NMR (400 MHz, $CDCl_3$)

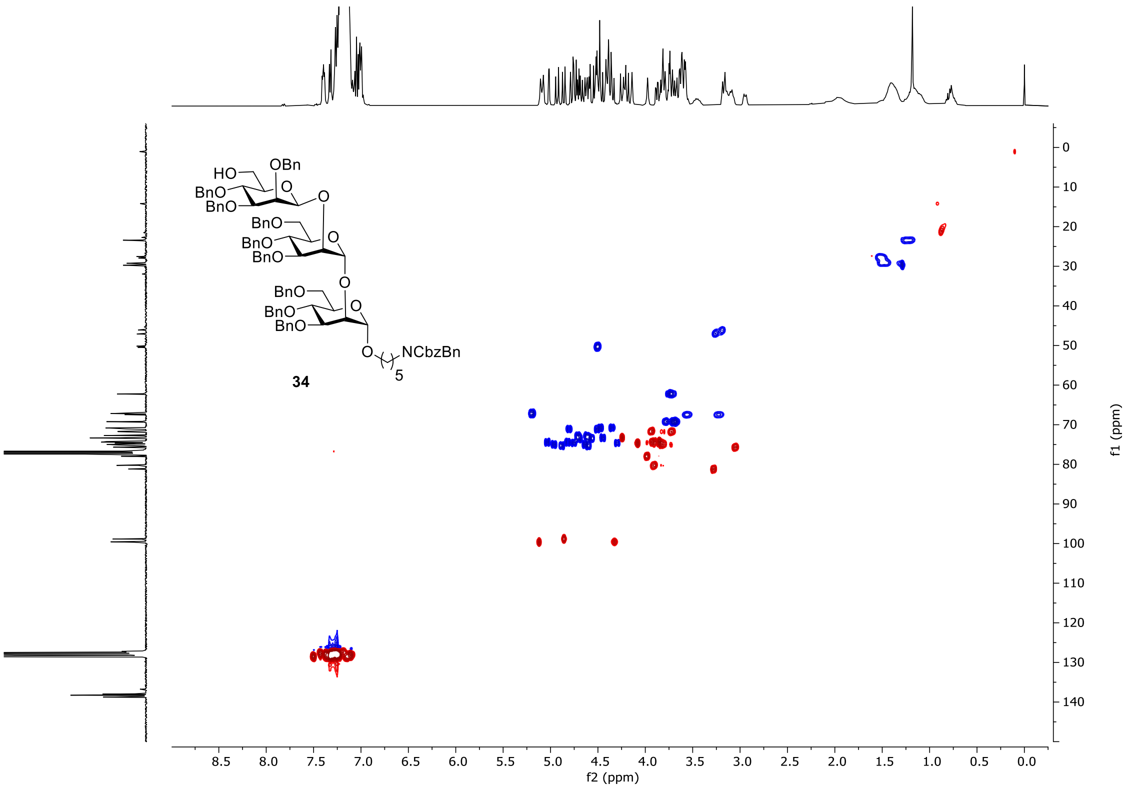

^{1}H-^{13}C Coupled HSQC NMR (400 MHz, $CDCl_3$)

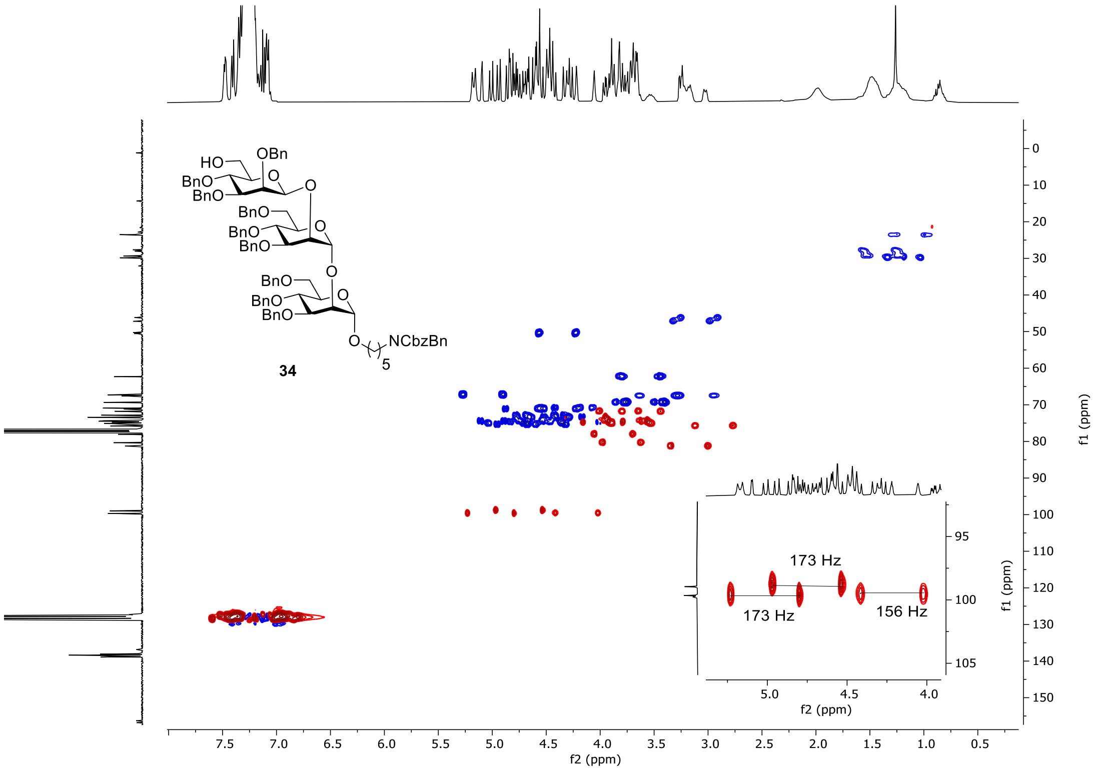

^{1}H NMR (700 MHz, D_2O)

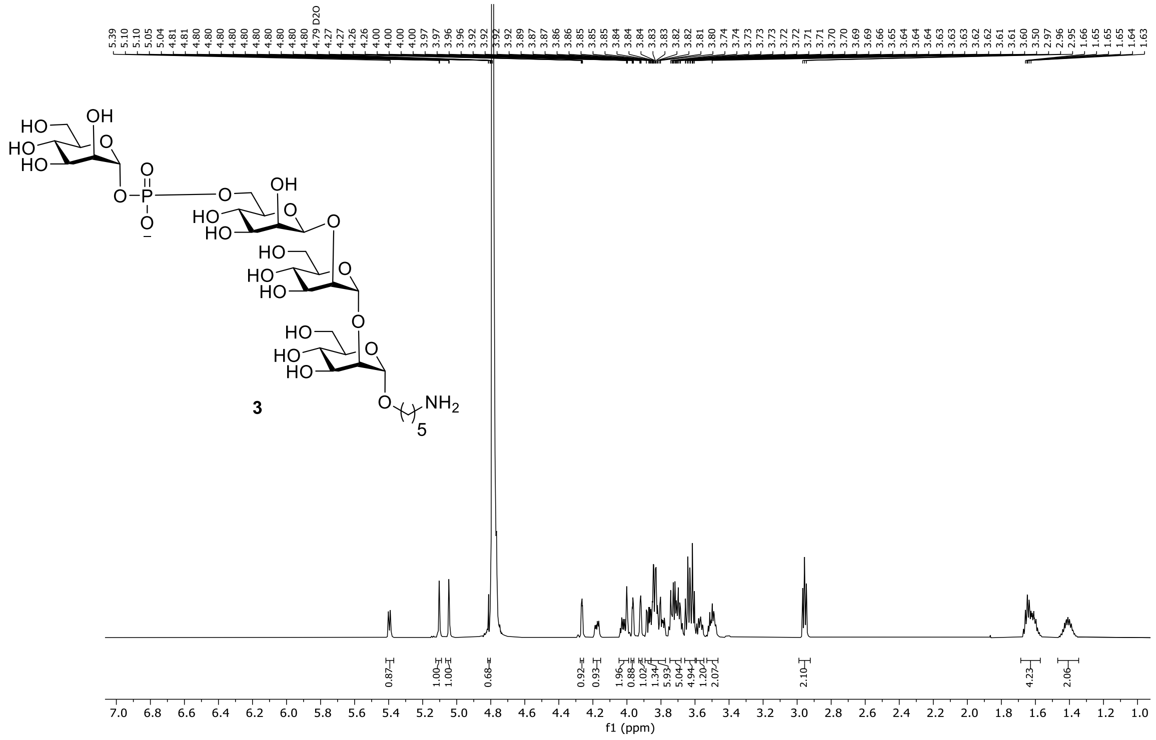

^{13}C NMR (176 MHz, D_2O)

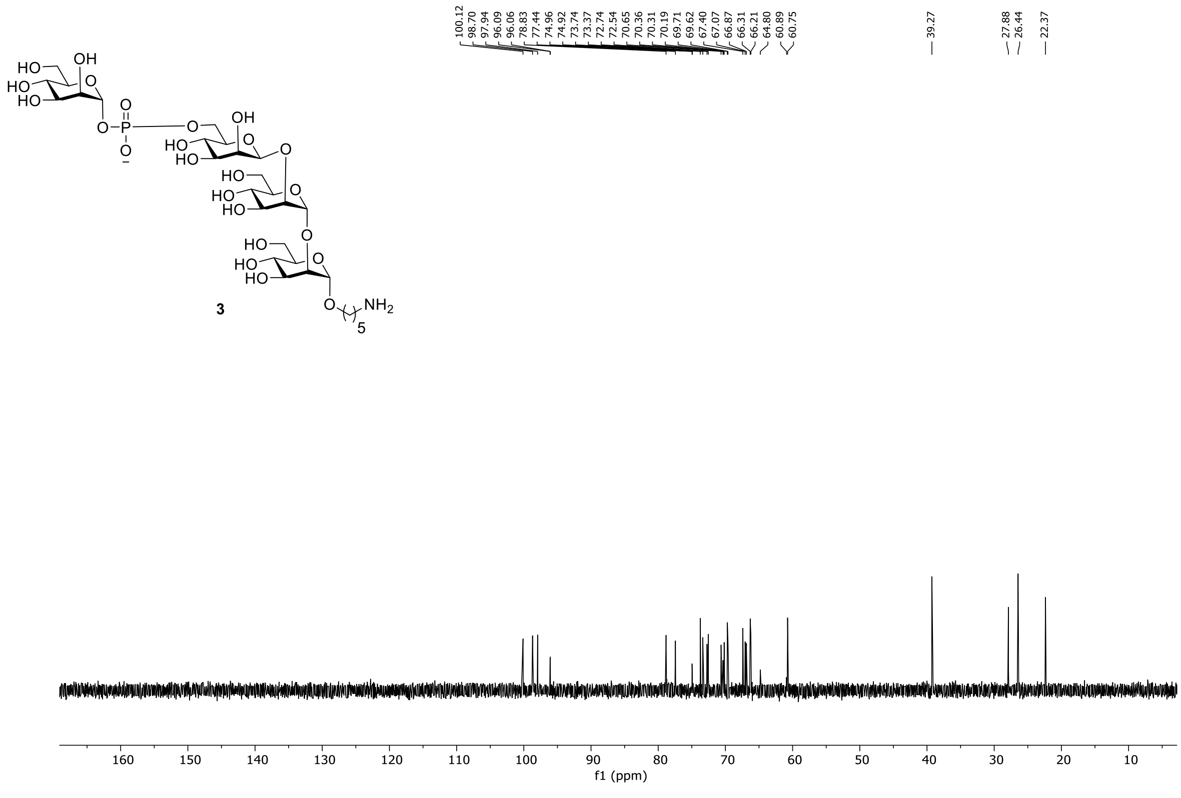

^{1}H-^{1}H COSY NMR (700 MHz, D_2O)

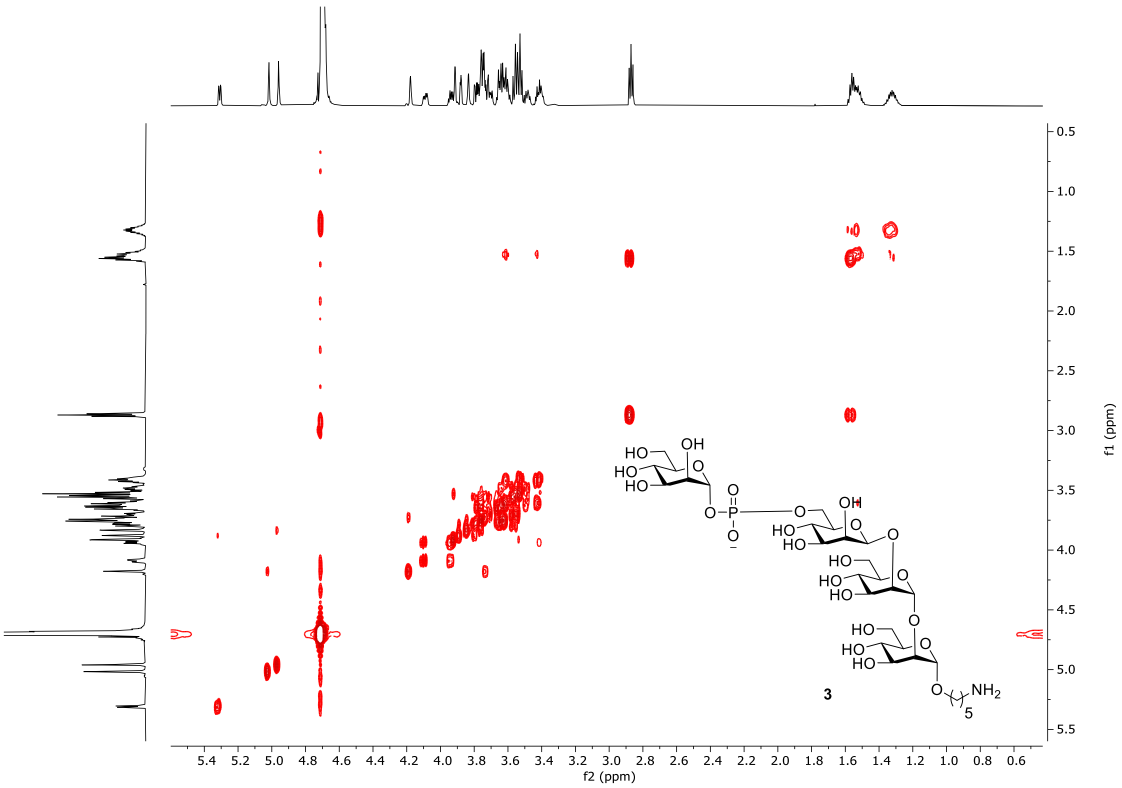

^{1}H-^{13}C HSQC NMR (700 MHz, D_2O)

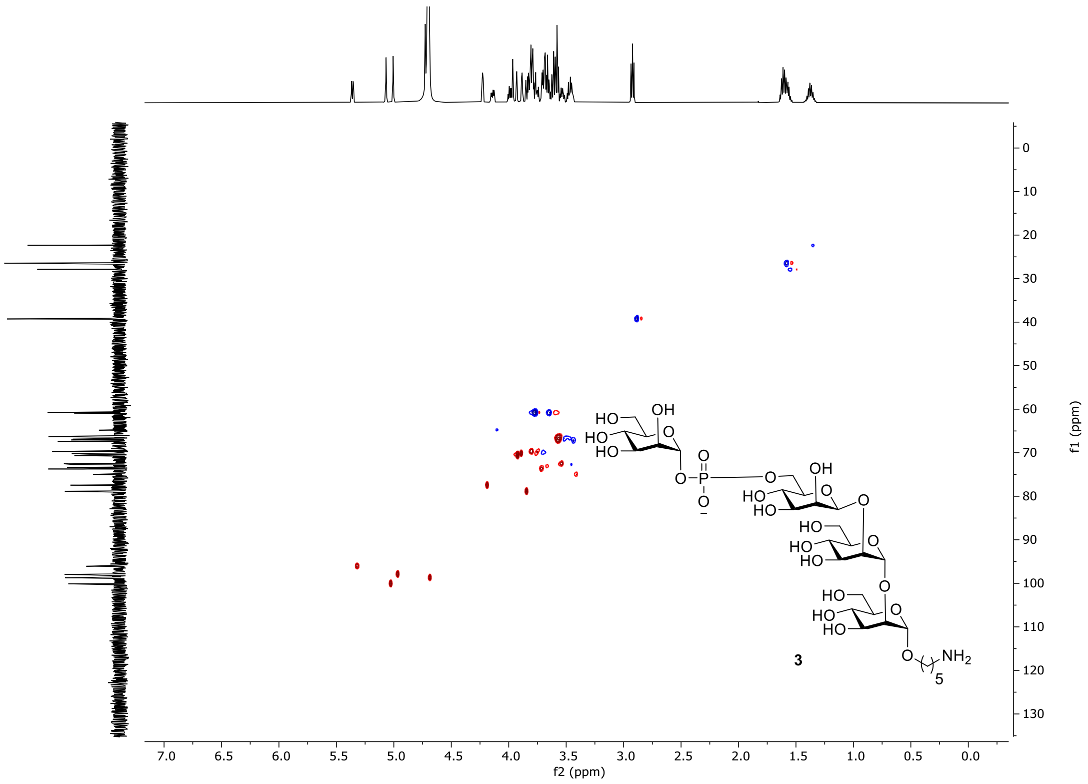

^{1}H-^{13}C Coupled HSQC NMR (700 MHz, D_2O)

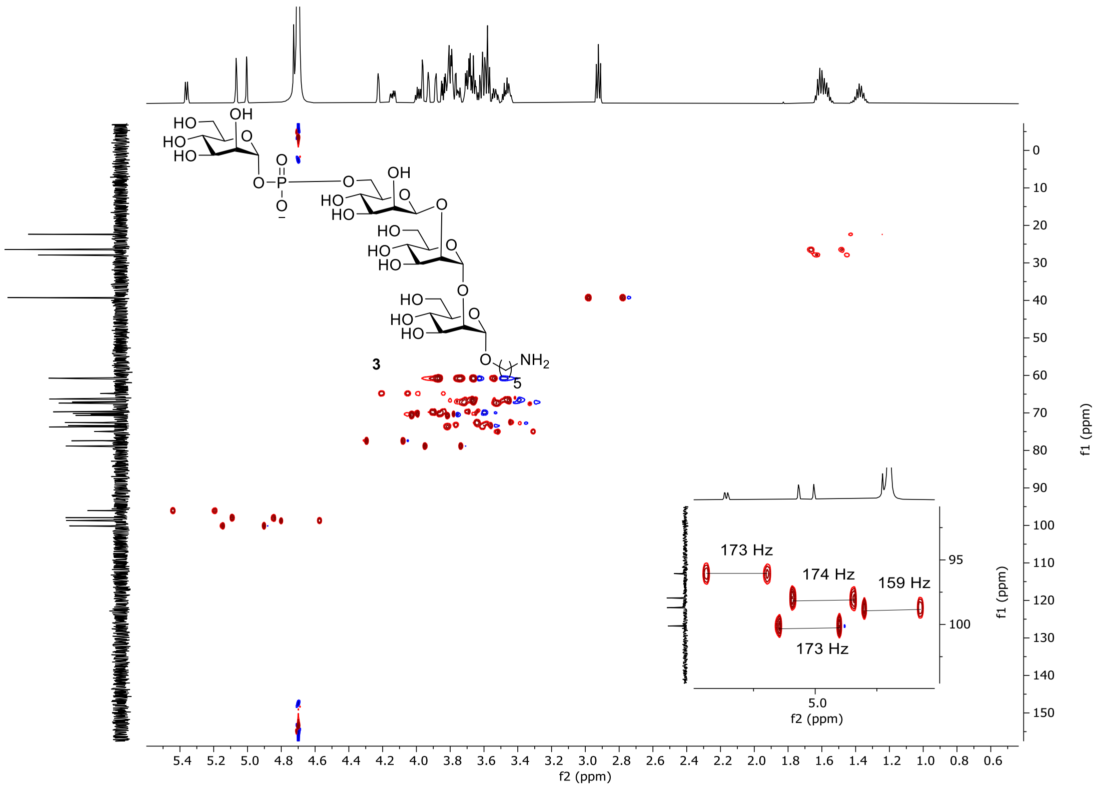

^{31}P NMR (243 MHz, D_2O)

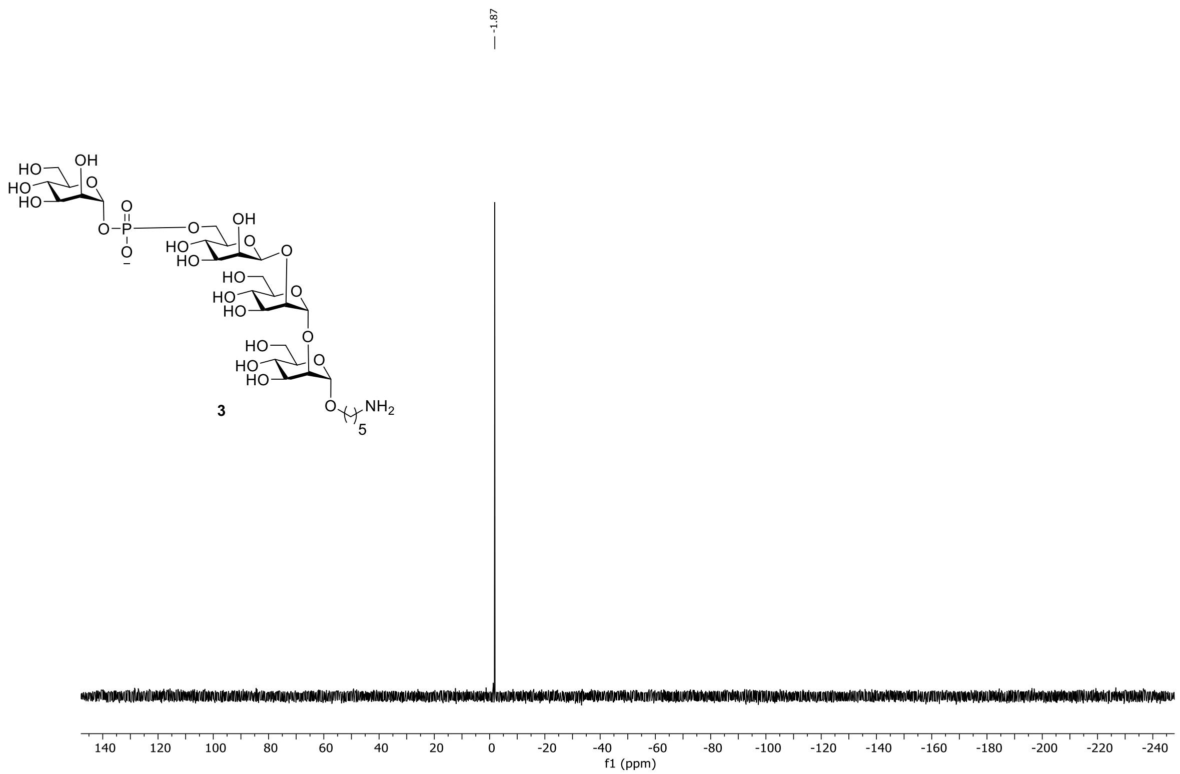

^{1}H NMR (700 MHz, $CDCl_3$)

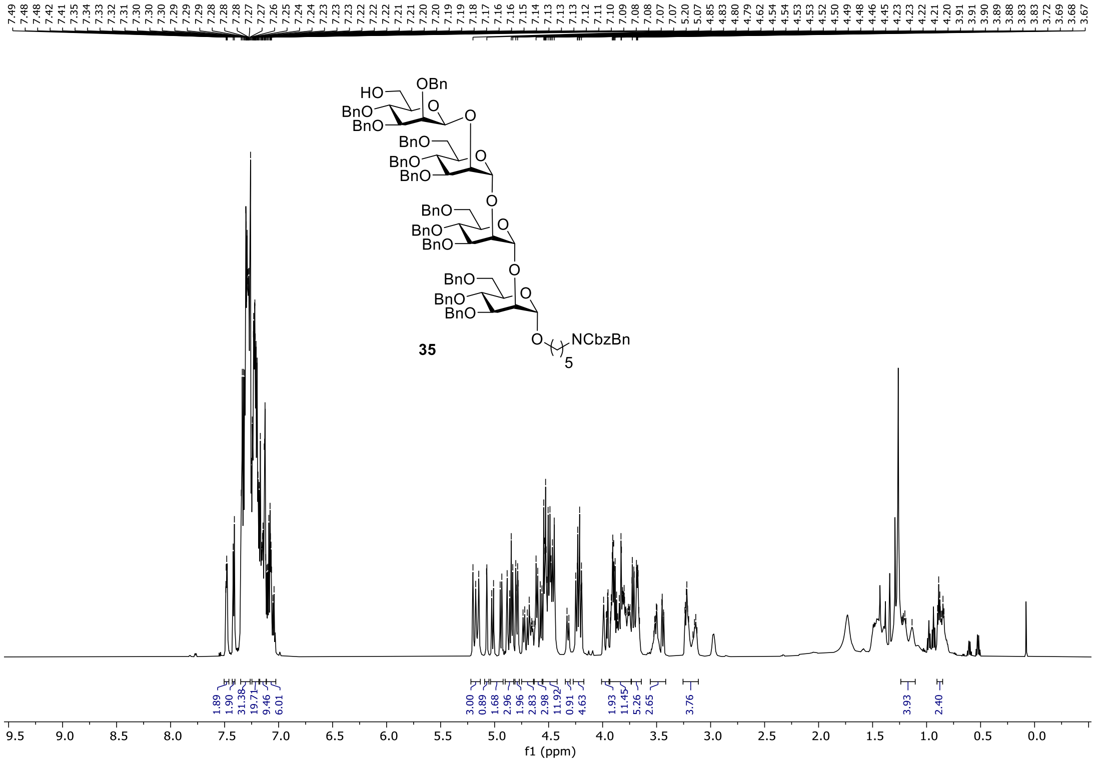

^{13}C NMR (176MHz, $CDCl_3$)

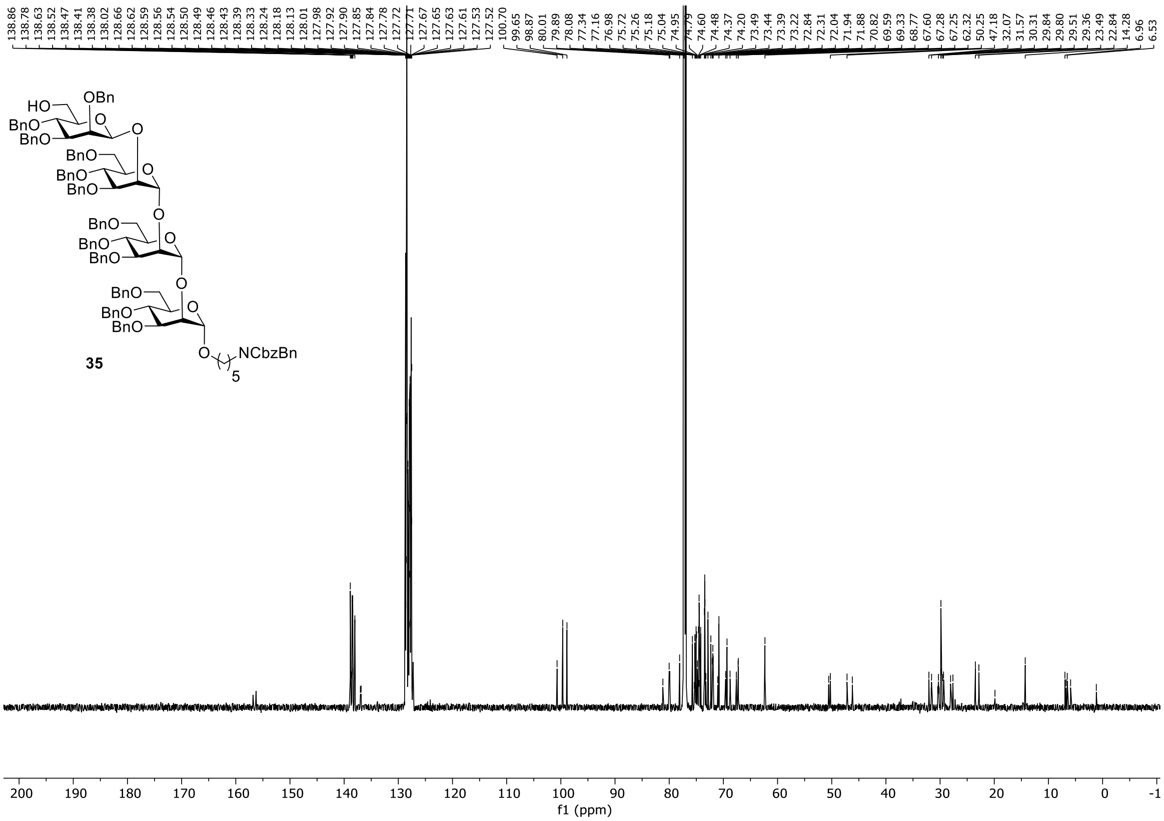

^{1}H-^{1}H COSY NMR (700 MHz, $CDCl_3$)

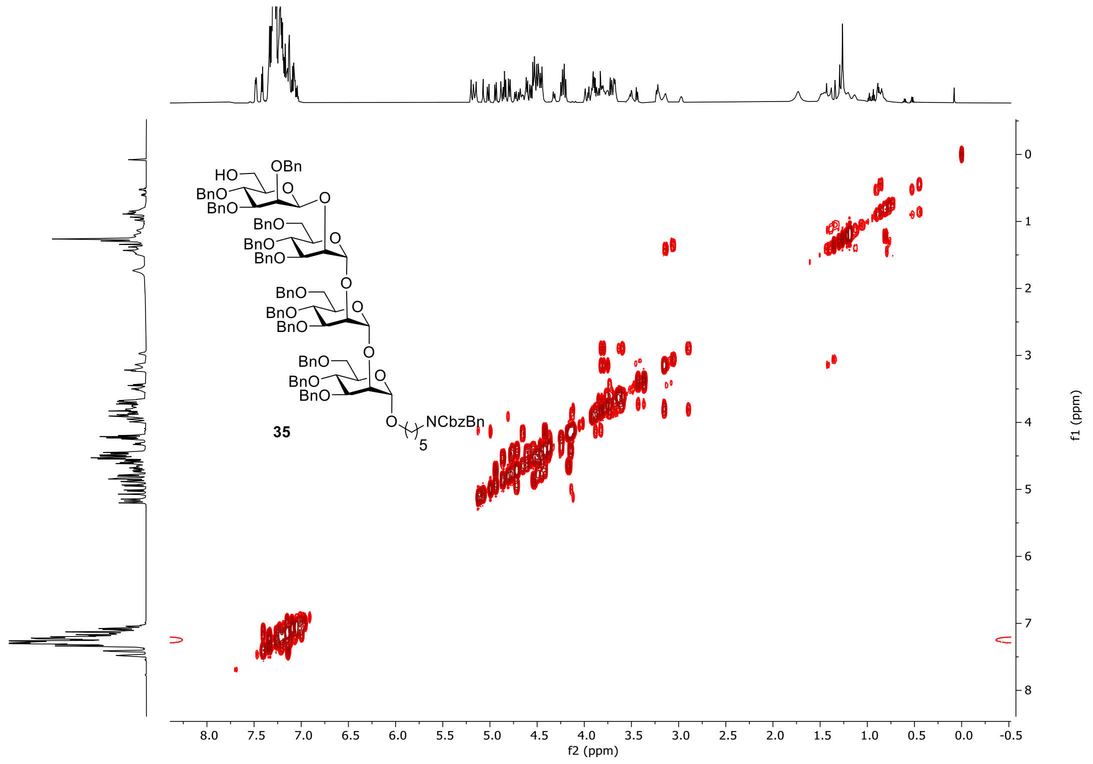

^{1}H-^{13}C HSQC NMR (700 MHz, $CDCl_3$)

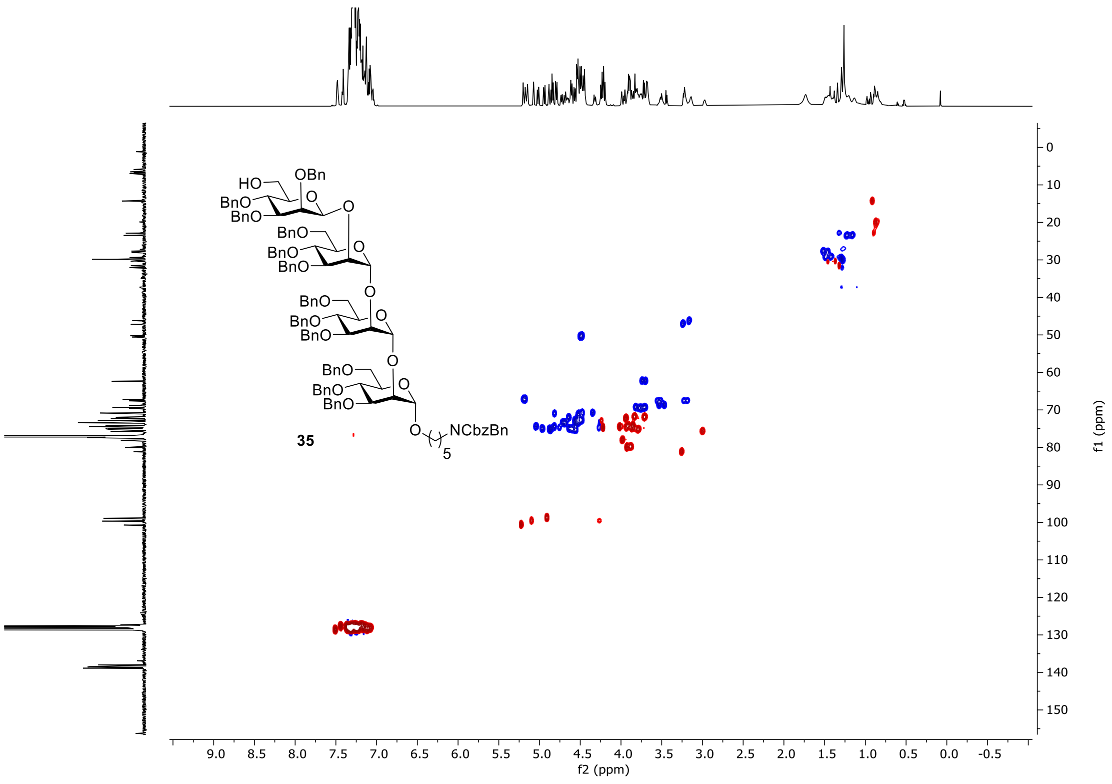

^1H-^{13}C Coupled HSQC NMR (700 MHz, $CDCl_3$)

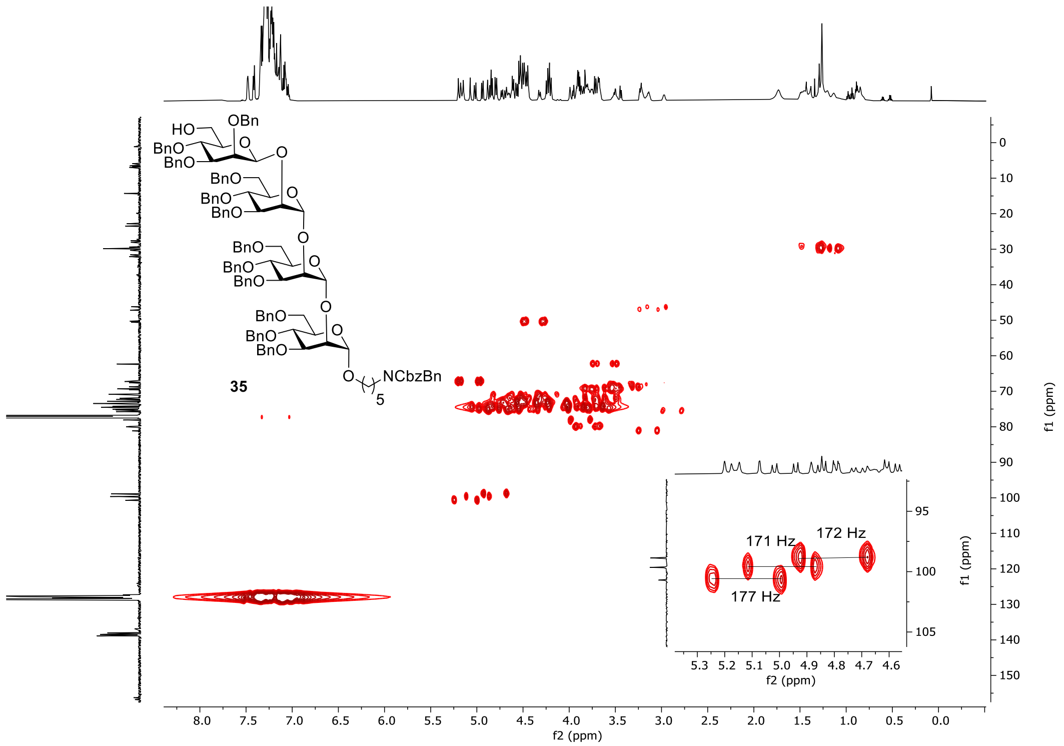

^1H NMR (700 MHz, D_2O)

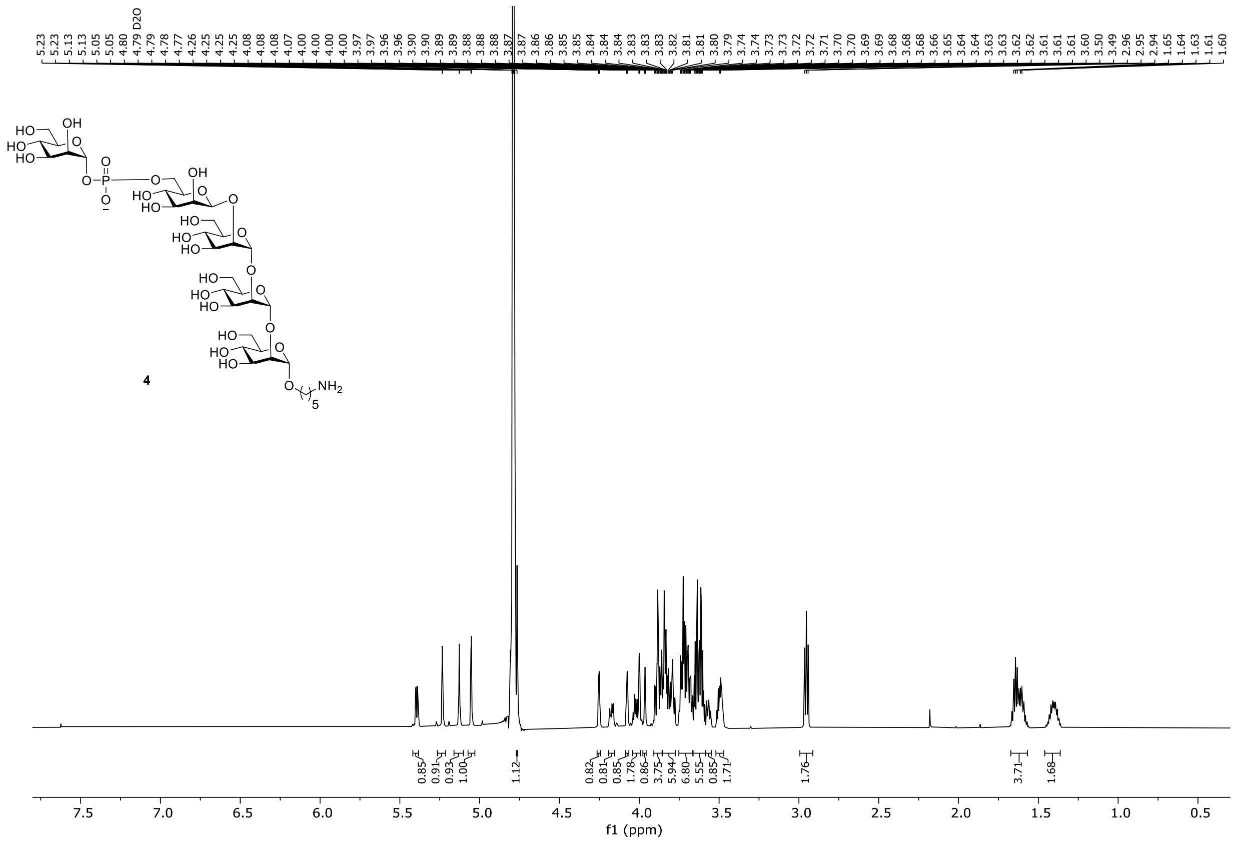

^{13}C NMR (176 MHz, D_2O)

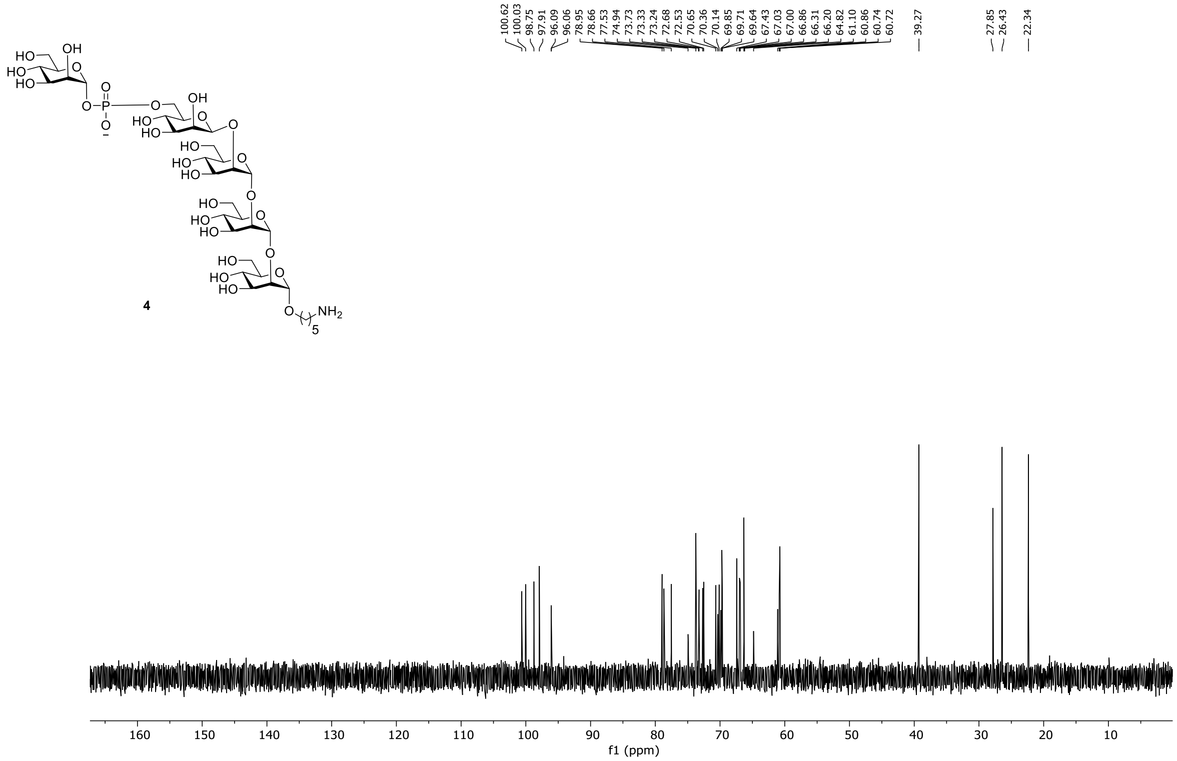

^{1}H-^{1}H COSY NMR (700 MHz, D_2O)

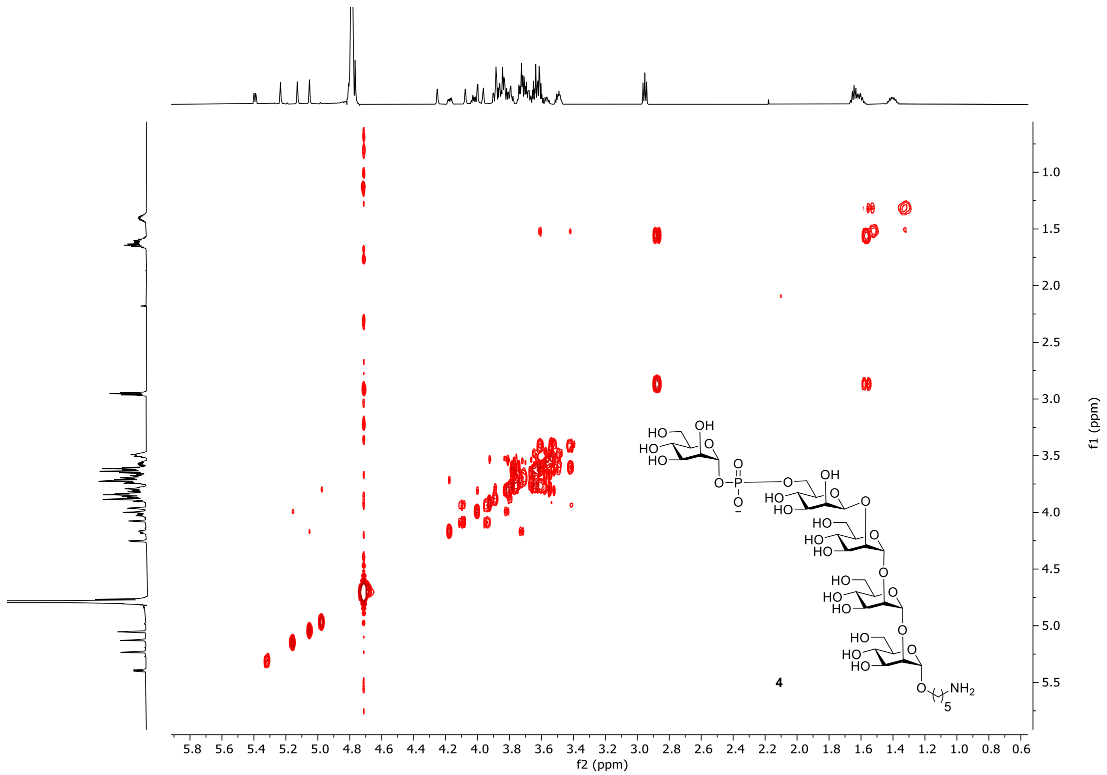

^1H-^{13}C HSQC NMR (700 MHz, D_2O)

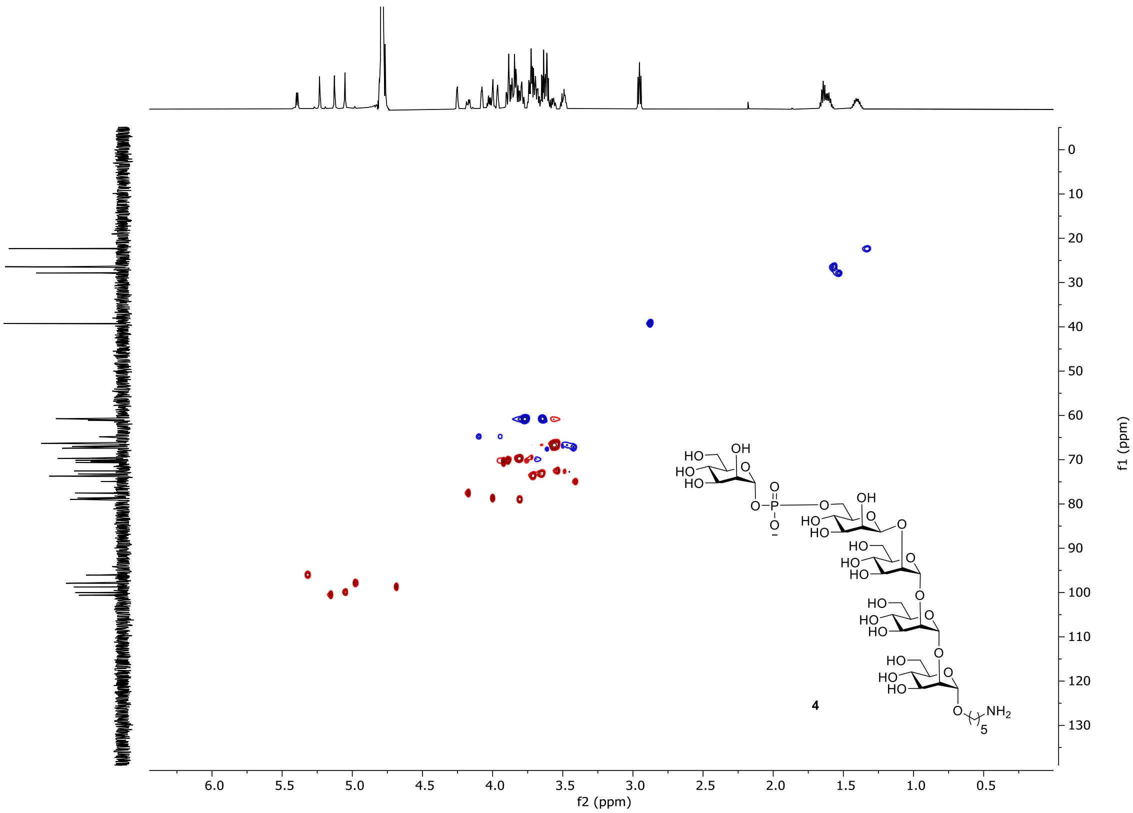

^1H-^{13}C Coupled HSQC NMR (700 MHz, D_2O)

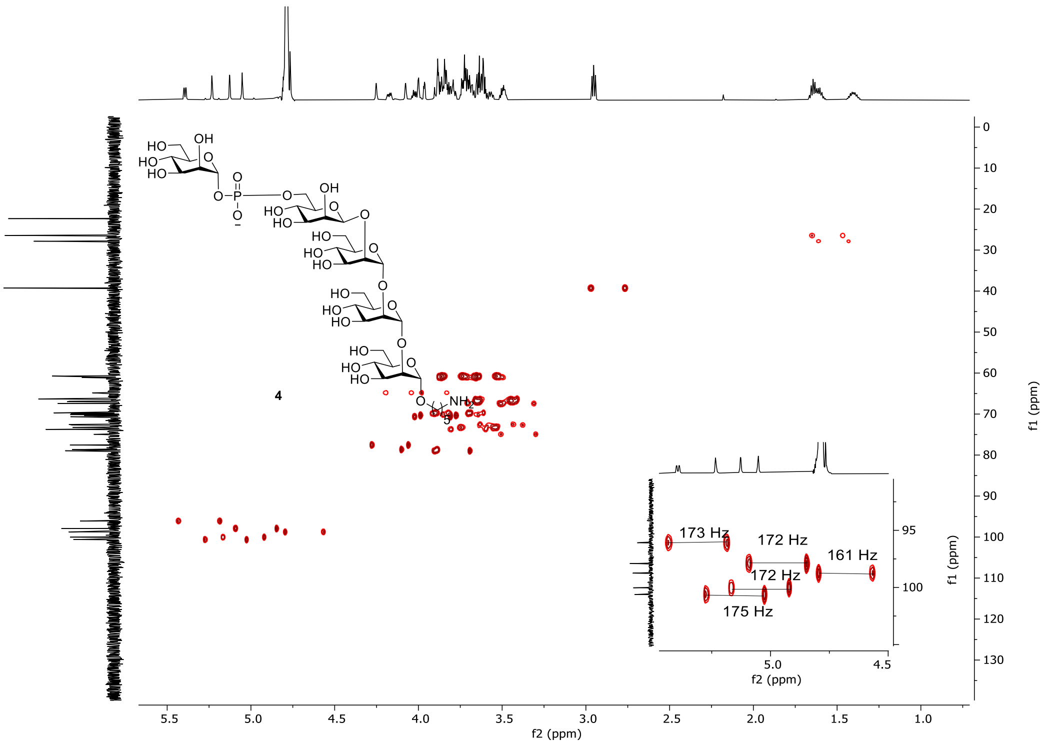

^{31}P NMR (243 MHz, $CDCl_3$)

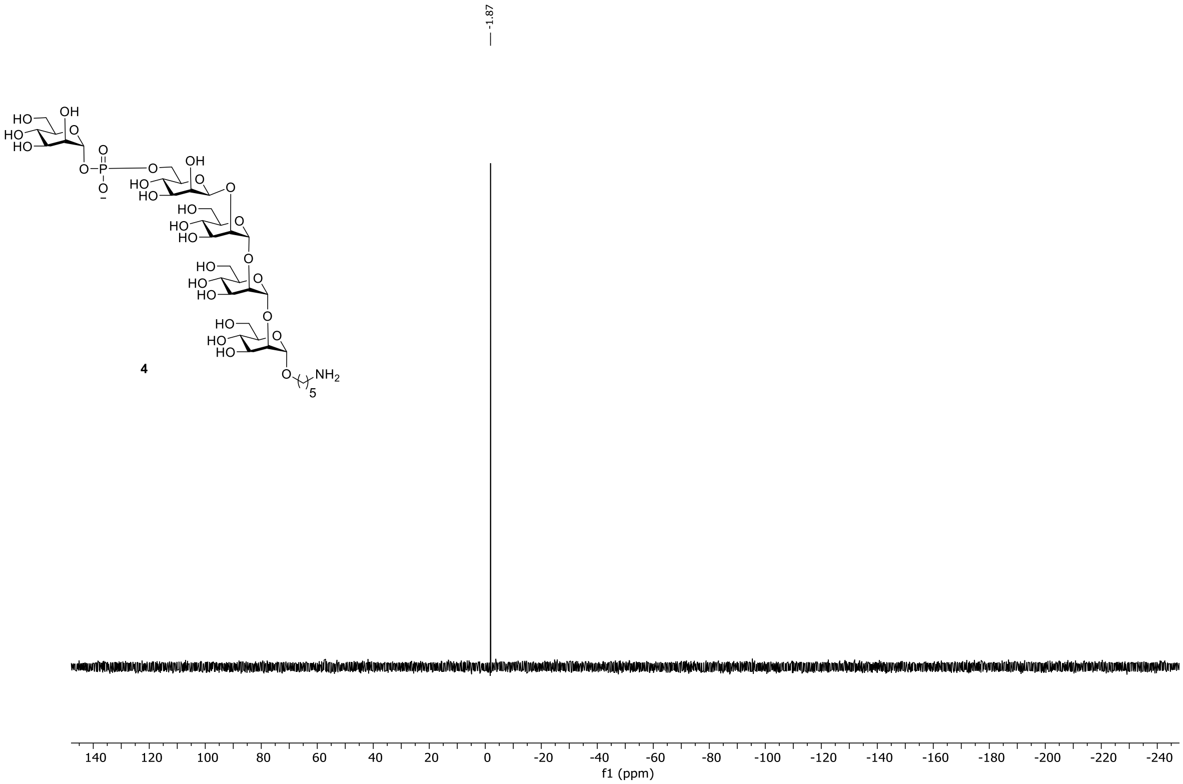

4

References

1. M. Bruno, S. Kersten, J. M. Bain, M. Jaeger, D. Rosati, M. D. Kruppa, D. W. Lowman, P. J. Rice, B. Graves, Z. Ma, Y. N. Jiao, A. Chowdhary, G. Renieris, F. L. van de Veerdonk, B.-J. Kullberg, E. J. Giamarellos-Bourboulis, A. Hoischen, N. A. R. Gow, A. J. P. Brown, J. F. Meis, D. L. Williams and M. G. Netea, *Nature Microbiology*, 2020, **5**, 1516-1531.
